# Mechanistic Insight into the Thermal Ring Opening of the Dewar Isomer of 1,2-Dihydro-1,2-azaborinines

**DOI:** 10.1021/jacsau.5c00923

**Published:** 2025-09-19

**Authors:** Sonja M. Biebl, Paul Ziemann, Markus Ströbele, Holger F. Bettinger

**Affiliations:** † Institut Für Organische Chemie, 9188 Eberhard Karls Universität Tübingen, Auf der Morgenstelle 18, 72076 Tübingen, Germany; ‡ Institut Für Anorganische Chemie, Eberhard Karls Universität Tübingen, Auf der Morgenstelle 18, 72076 Tübingen, Germany

**Keywords:** 1,2-dihydro-1,2-azaborinine, molecular solar thermal energy storage, MOST, photoisomerization, Dewar isomer, thermal ring opening, Hammett analysis

## Abstract

In contrast to the multichannel photochemistry of benzene, 1,2-dihydro-1,2-azaborinines generally undergo a selective electrocyclic ring-closure reaction, yielding the corresponding Dewar (2-aza-3-borabicyclo[2.2.0]­hex-5-ene) isomers. The Dewar isomers react back to the dihydroazaborinines in unimolecular reactions with long half-lives at room temperature, making this isomer pair of relevance as a molecular solar thermal (MOST) energy storage system. A systematic investigation of the influence of C-backbone functionalization on the photoisomerization or the thermal ring opening has not yet been conducted. We report here a study of the late-stage C3 functionalization of 1,2-dihydroazaborinines by cross-coupling and discuss the electronic impact of aryl substitution at C3 on the properties of 1,2-dihydro-1,2-azaborinines itself, as well as on the photoreaction and the thermal back reaction employing a Hammett analysis for the latter. 3-Aryl-substituted dihydroazaborinines react selectively and reversibly in almost quantitative fashion to the Dewar isomer. The Woodward–Hoffmann forbidden thermal ring-opening reaction proceeds by interaction with the adjacent boron center involving a three-center–two-electron interaction and is accelerated by both electron-withdrawing and electron-donating groups. Through the targeted selection of C3 substituents, both the electronic absorption characteristics of the dihydroazaborinine and the half-life of its Dewar isomer can be addressed.

## Graphical abstract

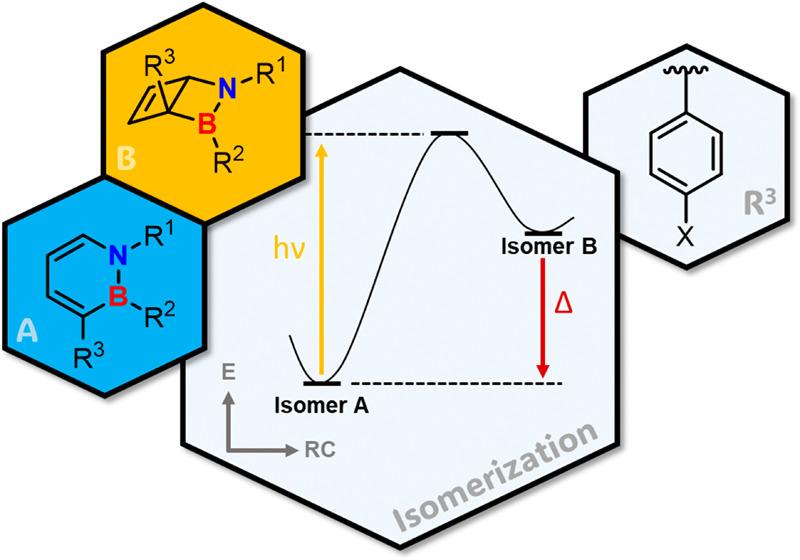

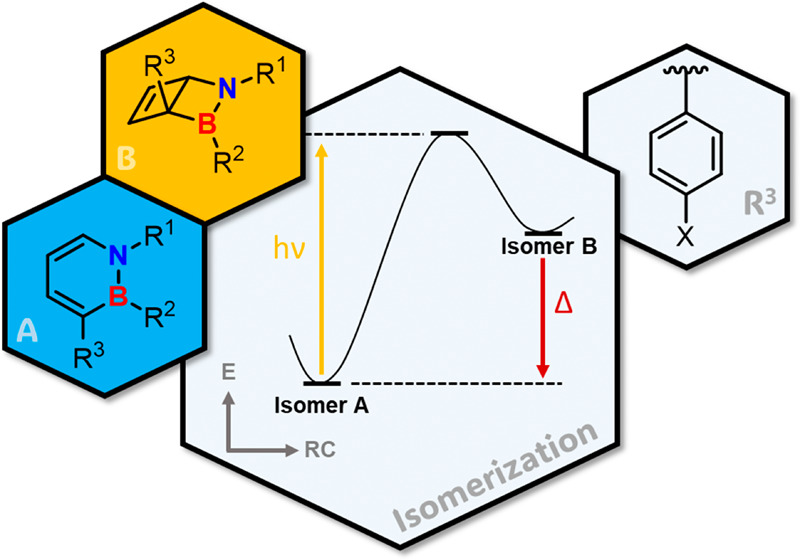

## Introduction

The replacement of selected CC units with their isoelectronic and isosteric BN units in π-conjugated organic compounds, particularly in polycyclic aromatic hydrocarbons (PAHs), has proven to be a successful strategy for the synthesis of novel organic–inorganic hybrid materials.^1–7^ Already for one of the simplest monocyclic BN-containing heterocycles, 1,2-dihydro-1,2-azaborinines, outstanding properties are evident.^8,9^ Upon exposure to light, representatives of this substance class undergo a selective electrocyclic photoisomerization to the corresponding Dewar species.^10–13^ More recently, Ozaki et al. demonstrated a selective photoisomerization to the benzvalene isomer through aryl substitution at the C5 position of the dihydroazaborinine backbone.^14^ While the parent Dewar isomer requires stabilization employing cryogenic conditions, sterically demanding substituents allowed for kinetic stabilization.^11^ Furthermore, it has been demonstrated that electrocyclic ring opening of the Dewar isomer to the dihydroazaborinine can proceed both thermally in a first-order reaction with a high activation energy of 27 ± 1.2 kcal/mol and catalytically.^11,12^ These examples already illustrate a clear dependence of photoisomerization,^15,16^ as well as thermal^11^ and catalytic^12^ ring-opening behavior, on the substituents of the heterocycle. Similar molecular photoswitches, which are capable of isomerizing between two states upon irradiation, have found applications in various fields such as molecular electronics, photopharmacology and, when the two isomers differ significantly in energy, molecular solar thermal (MOST) systems.^17^ A deeper understanding of the effects of substituents on dihydroazaborinines and their deliberate application could open up a wide range of potential applications for this substance class.

To enable a targeted application of dihydroazaborinines, insights into the structure–property relationship of photoisomerization and thermal ring-opening processes are essential. However, the corresponding studies are rare, and available insights were derived mainly from theoretical investigations. The available computational data of the photoreaction point toward a highly efficient process,^18–20^ in agreement with the finding that the isomerization also readily proceeds at 4 K in solid Ne.^10^ The work of Ozaki et al. indicates that additional aryl groups on C5 can result in a follow-up photoreaction of the Dewar into the benzvalene isomer.^14^ For the thermal back reaction of the parent 1,2-dihydro-1,2-azaborinine, Bettinger and Hauler identified a stepwise process using multiconfiguration SCF (CASSCF) and coupled cluster theory at the CCSD­(T) level.^21^ This mechanism is more favorable compared to the conventional con- or disrotatory pathways.^21^ In contrast, bulky substituents at the boron and the nitrogen heteroatoms lead to a concerted mechanism of the thermal cycloreversion.^11,22^

For the investigation of structure–property relationships of dihydroazaborinines in the context of MOST, efficient late-stage functionalization is deemed important for the introduction of substituents of variable electronic character. Existing literature protocols for the late-stage functionalization of the dihydroazaborinine backbone predominately focus on the reactive boron atom, where, C-,^11,23–26^ O-,^27^ S-,^28^ and N-nucleophiles^28^ can be introduced readily (Figure 1a). Additionally, a broad scope of aryl and alkenyl groups can be implemented via Stille^29^ or Heck^30^ couplings. For the electron-rich counterpart, nitrogen, reactions with different organic^31^ or inorganic^32^ electrophiles after deprotonation are established. Additionally, Liu et al. successfully performed Buchwald–Hartwig coupling on four substrates.^31^ The C3 position has the highest electron density in the carbon backbone and can be functionalized by simple electrophilic aromatic substitution reactions, e.g., bromination,^33,34^ while Friedel–Crafts acylation is selective toward C5.^33,35^ Further utilization of the brominated compound was introduced by Brown and Liu through Negishi coupling.^36,37^ Except for the already mentioned Friedel–Crafts acylation, modifications at other positions of the carbon backbone (C4–C6) have thus far been achieved exclusively by direct electrophilic C–H borylation (see Figure 1a).^14,38,39^ Here, high regioselectivity for the C5 position across a broad range of dihydroazaborinines and aryl halides is evident.^14^ The protocol by Liu et al. targeting C6 demonstrates regioselectivity in the presence of an unsubstituted nitrogen,^38^ whereas regioselective late-stage functionalization of the C4 position has not yet been achieved.^39^ Multifunctionalized dihydroazaborinines are accessible, e.g., using protocols developed by Braunschweig,^40^ Dong,^41^ or Liu et al.^42^

**Figure 1:**
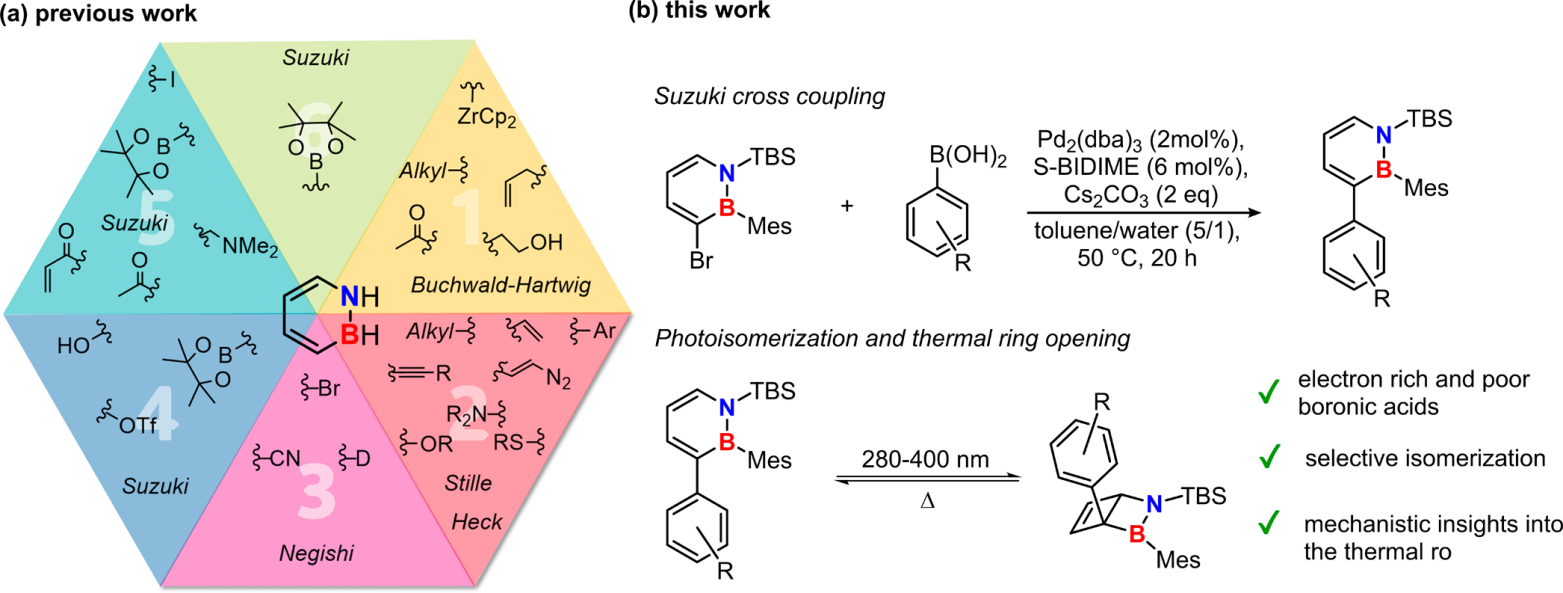
a) Examples for previously published synthetic methods which allow for a late-stage functionalization of different positions of the dihydroazaborinine framework. (b) Our investigations on C3 aryl-substituted dihydroazaborinines.

In the present work, we determine the influence of substitution at the C3-position (see Figure 1b) as this is one site at which the C–C bond undergoes cleavage during the thermal ring opening. In terms of the catalytic ring opening, Richter et al. identified this rehybridization at C3 as part of the rate-determining step by measuring a secondary kinetic isotope effect of 1.10 ± 0.02.^12^ We examined a series of C3 aryl-substituted compounds and found that the nature of the substituent affects the UV–Vis absorption of dihydroazaborinines as well as the rate and course of thermal ring opening (see Figure 1b). This provides the first mechanistic study of this orbital symmetric forbidden thermal reaction. Such investigations are essential for advancing the mechanistic understanding of the isomerization process, thus improving property predictions. Ultimately, this could facilitate a rational design of dihydroazaborinines that are ideally suited for applications in MOST systems.

## Results and Discussion

### Synthesis

Dihydroazaborinine **1** was synthesized based on previously published literature procedures (for details, see Supporting Information).^11,43^ The bromination of the C3 position is achieved with elemental bromine and was reported earlier for three different dihydroazaborinine derivatives (Scheme 1a).^34,36,44^ Furthermore, we successfully expanded the halogenation to additional carbon positions and to chlorination. These species themselves represent valuable synthons for the functionalization of dihydroazaborinines. In this study, however, we limit their application to competition experiments designed to probe the selectivity of the catalytic system developed herein. The two- and trifold bromination can be achieved with elemental bromine as well and leads to a mixture of four different brominated dihydroazaborinines **2–5**, that could be separated by column and size exclusion chromatography (Scheme 1b).

**Scheme 1:**
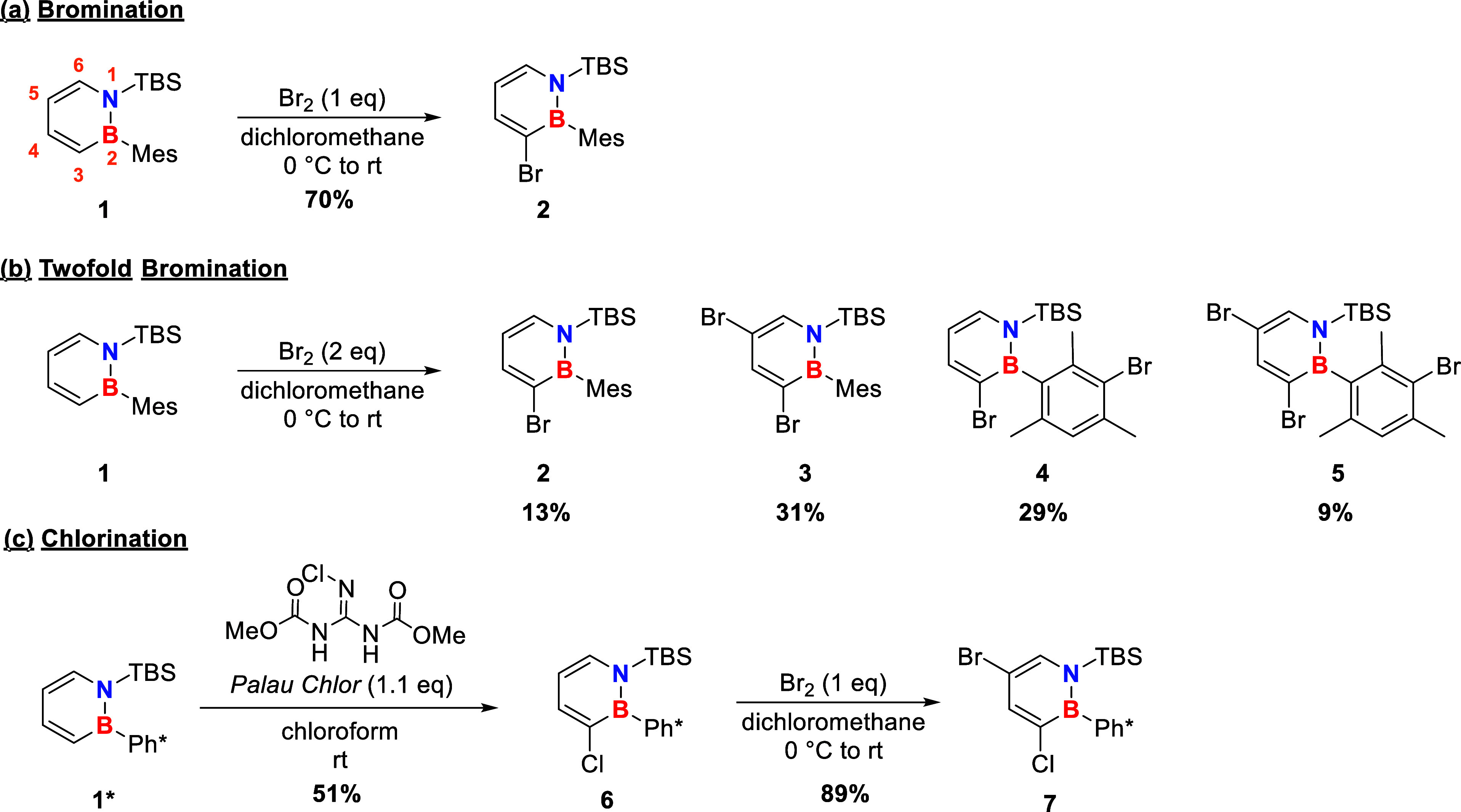
Synthesis of the Brominated Dihydroazaborinines **2–4** and the Chlorinated Species 6 Starting from the Literature-Known Compound **1**. Ph*: Pentamethylphenyl

The brominated carbon positions could be identified by NMR and proven via single-crystal diffraction (Figure 2). The C–Br bond length of **2–5** ranges from 1.884(3) to 1.912(2) Å, without showing any trend that extends over the four dihydroazaborinines. Molecular packing in the solid state is characterized by intermolecular interactions between the silyl protection group on nitrogen and the mesityl fragment shielding the boron atom for all products **2–5**. This interplay is not affected by bromination of the mesityl ring.

**Figure 2:**
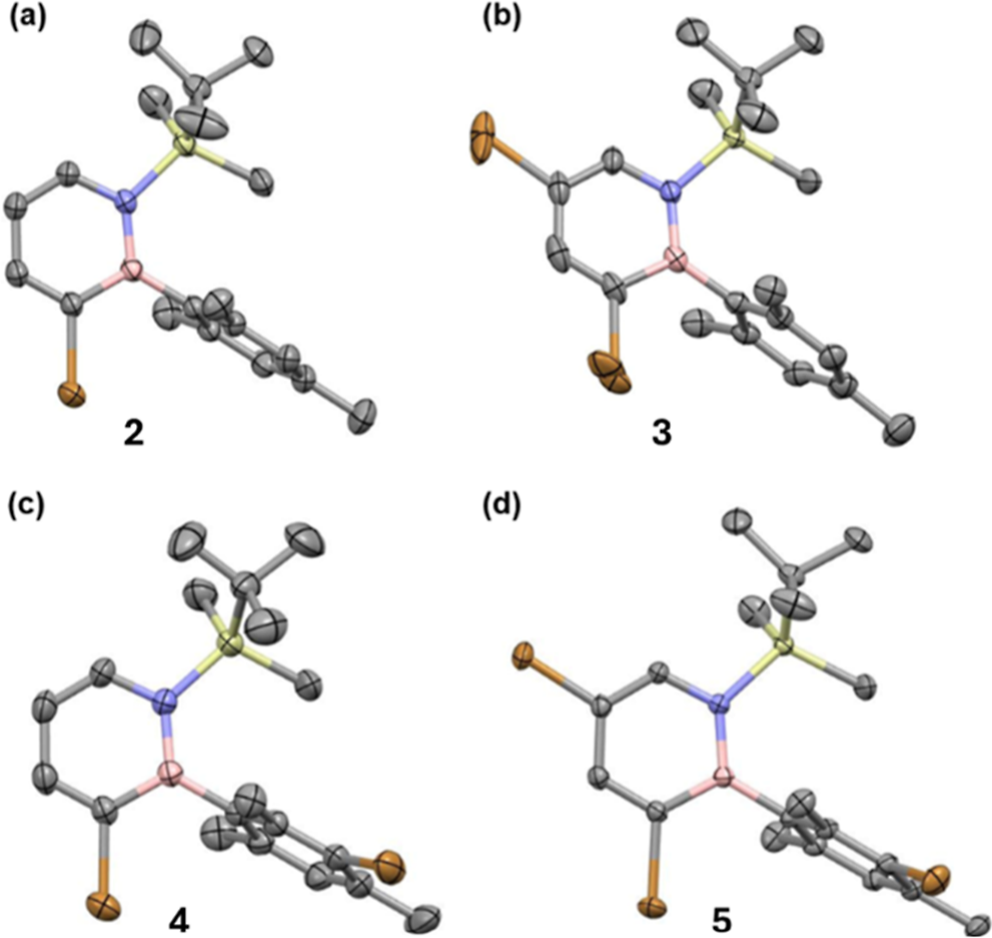
Molecular structures in the solid state of the different brominated dihydroazaborinines synthesized. Hydrogen atoms are omitted for clarity. Thermal ellipsoids are drawn at the 50% probability level. Lengths of C–Br and B–N bonds in Å: **2** (CCDC 2290772): Br1–C3 1.909(1), N1–B1 1.444(2); **3** (CCDC 2341277): Br1–C5 1.958(4), Br2–C3 1.965(3), N1–B1 1.448(2); **4** (CCDC 2350121): Br1–C3 1.912(2), Br2–C14 1.903(2), N1–B1 1.447(3); **5** (CCDC 2348307): Br1–C5 1.891(1), Br2–C3 1.902(1), Br3–C14 1.908(1), N1–B1 1.446(2).

For the chlorination of the C3 position, previously published variations using *N*-chlorosuccinimide were not successful with the employed dihydroazaborinine 1* (the pentamethylphenyl ring was used instead of mesityl to avoid its bromination in the synthesis of compound **7**).^34^ This applies to various experiments using different solvents, reaction temperatures, and times. Ultimately, the reaction was successfully carried out with guanidine-based Palau Chlor.^45^

With these halogenated compounds in hand, our objective was to establish a Suzuki cross coupling protocol in which these species were used as reactants. Given the widespread commercial availability of boronic acids, their increased tolerance toward functional groups, and their enhanced stability and diminished toxicity compared to organozinc compounds, we preferred the Suzuki approach over the literature-known Negishi coupling.^37^ For the optimization of the catalytic system, dihydroazaborinine **2** (1 equiv) and phenyl boronic acid (1.3 equiv) were chosen as the model reaction. As given in Table 1, catalytic systems commonly employed for all-carbon analogues proved ineffective under the applied conditions (entry 1; for more information, see Supporting Information).^46^ The same is true for a variety of catalytic systems published for N-heterocyclic aryl halides (entry 2; for more information, see Supporting Information).^47,48^ Selected coupling strategies designed for sterically hindered substrates were likewise unsuccessful (entry 3).^49^ However, the Suzuki cross coupling protocol for 2,1-BN-naphthalene by Song and co-workers was applicable with minor modifications.^50^ The dihydroazaborinine system appeared to be more sensitive with respect to the base. Also, the slight variations of the ligand described by Song et al.^50^ were not possible in our case.

**Table 1:**
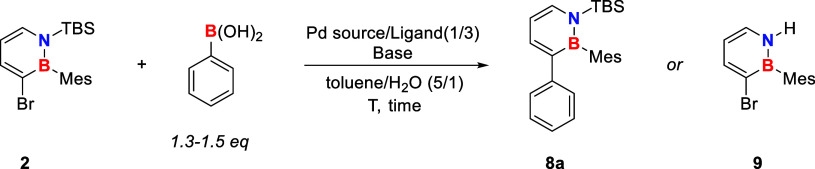

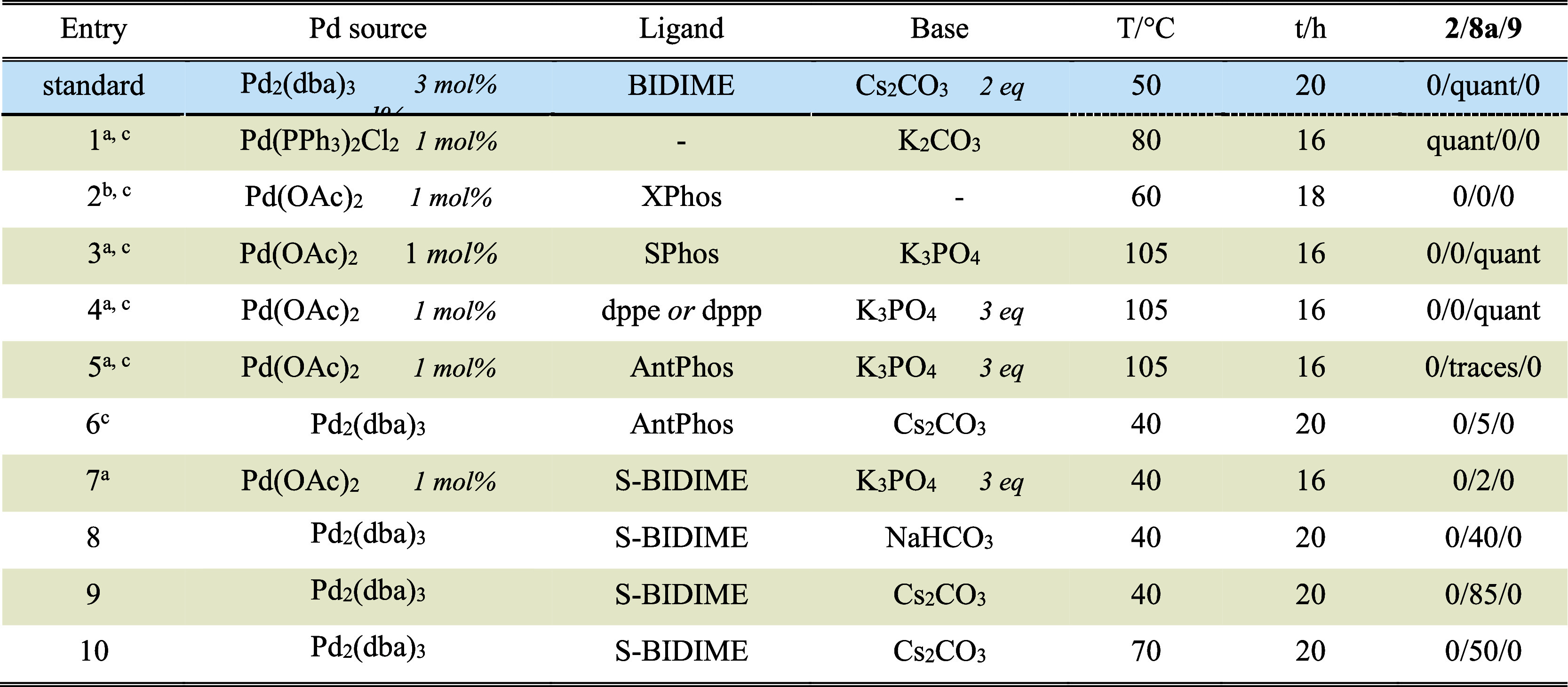
Screening and Optimization of the Catalytic System for the Suzuki Cross Coupling of C3 Brominated Dihydroazaborinines

a Toluene was used as the solvent.

b MeCN/H_2_O (3/2) was used as the solvent.

c A 1/2 ratio of the catalyst/ligand was used.

When diphenylphosphinoalkyl ligands in combination with palladium­(II)­acetate as a palladium source and potassium phosphate as a base are used, the cleavage of the silyl protection group is observed in a catalytic fashion (Table 1, entry 4).

Having identified a suitable catalytic system, we optimized other reaction parameters. A 1/3 ratio between the catalyst and ligand proved to be optimal, analogous to the protocol by Song et al.^50^ Increasing the temperature slightly (50 °C) offered reduced reaction times. However, further increases led to diminished yields (entry 10). A black precipitate in these screening attempts points to a faster decomposition of the catalytically active species at these higher temperatures.

### Substrate Scope—Boronic Acid

With these optimized conditions given, the substrate scope and functional group tolerance with respect to the boronic acid was investigated (Scheme 2). The system was compatible with boronic acids bearing electron-donating groups, including tertiary amines, alkoxy, and methylthiol, yielding the corresponding cross coupling products in high GC-FID yields (Scheme 2). Boronic acids with electron-withdrawing substituents were also tolerated. However, increased reaction time was required to reach a clean, reliable, and maximal conversion with the strongly electron-withdrawing trifluoromethyl substituent. Substrates with even stronger electron-withdrawing groups, e.g., nitroso, cyano, or methylsulfoxide (**8k**), were not converted successfully with the described catalytic system. The latter (**8k**) could still be obtained via oxidation of the corresponding thioether **8d** (Scheme 3).

**Scheme 2:**
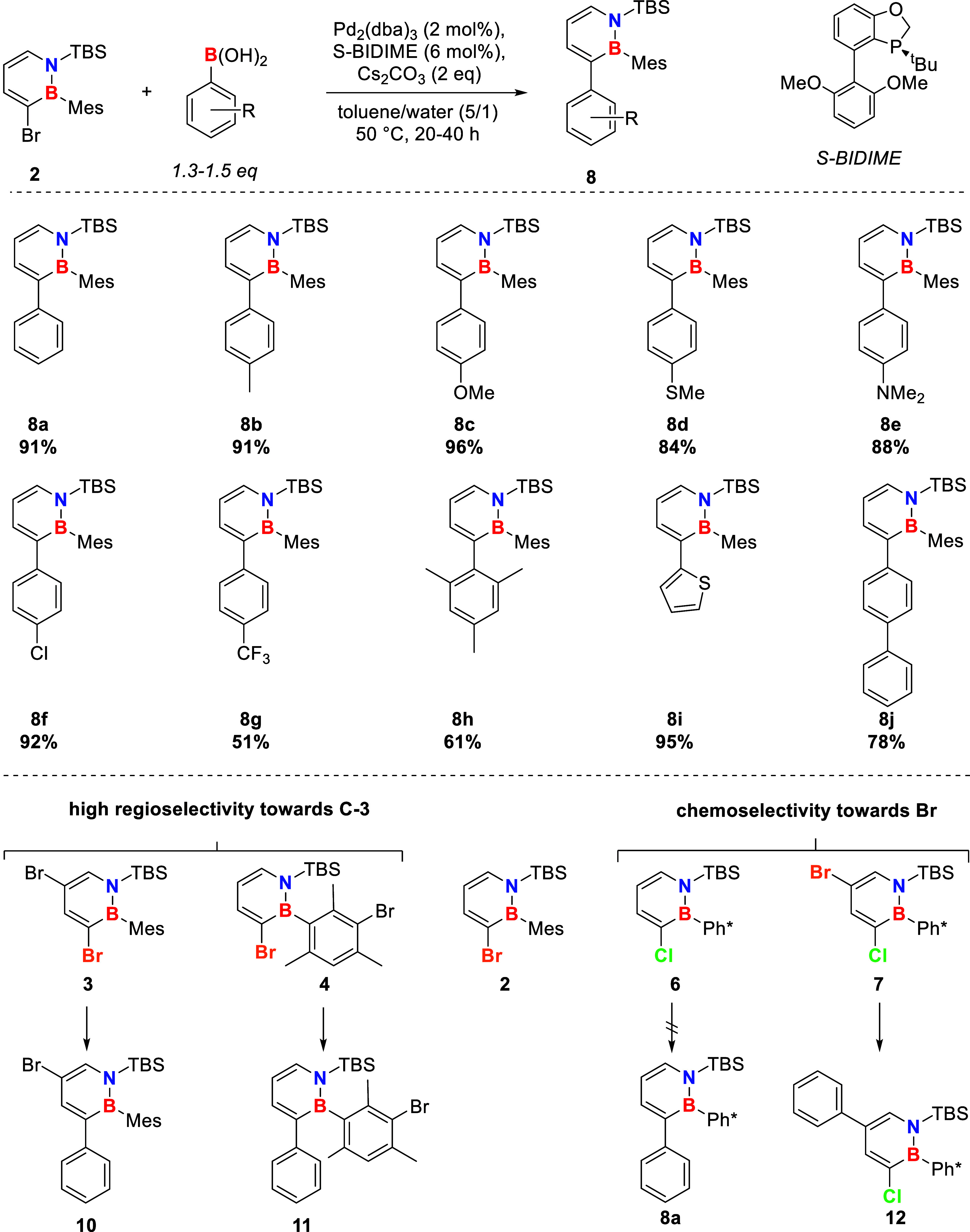
Substrate Scope with Respect to the Boronic Acid, GC-FID Conversion, and Yield for the Optimized Reaction Conditions. A Crystal Structure of 8j Can Be Found in the Supporting Information (CCDC 2336783). Regio- and Chemoselectivity of the Catalytic System toward Different Brominated and Chlorinated Dihydroazaborinines (Bottom)

**Scheme 3:**
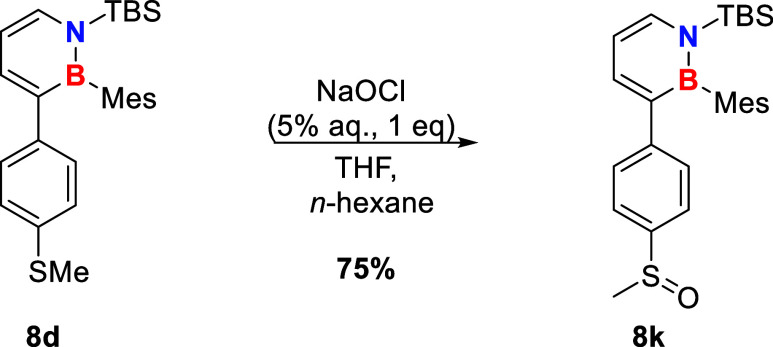
Synthesis of Dihydroazaborinine **8k** via Selective Oxidation of **8d**

Even boronic acids bearing heteroaromatic components (**8i**) or sterically demanding groups (**8h**) were tolerated in good to excellent yields. Upscaling experiments for **8c** also provided high isolated yields (for more information, see Supporting Information).

### Competition Experiments

Aiming for a better understanding of the reactivity of the dihydroazaborinine system in the cross-coupling reaction, competition experiments were conducted. In terms of regioselectivity, the more electrophilic C3 position is preferred for the two-fold brominated species **3** and **4**, despite the sterically demanding mesityl substituent at the adjacent boron position. In case of the C3 chlorinated compound **6**, the reagents were recovered in approximately quantitative yield demonstrating a high chemoselectivity of the catalytic system (Scheme 2). If the chlorine at C3 is accompanied by a bromine atom on C5 (**7**), a coupling to C5 can be achieved with high chemoselectivity. To improve the reaction efficiency and increase yields, a reoptimization of the catalytic system was necessary (for more information, see Supporting Information).

### UV–Vis Absorption

Considering electronic effects of the *para* substituent of the attached aryl ring on the absorption spectra (Figure 3), the absorption onset as well as the maximum absorption wavelength shift bathochromically as the electronic effect of the substituent becomes stronger (Table 2).

**Figure 3:**
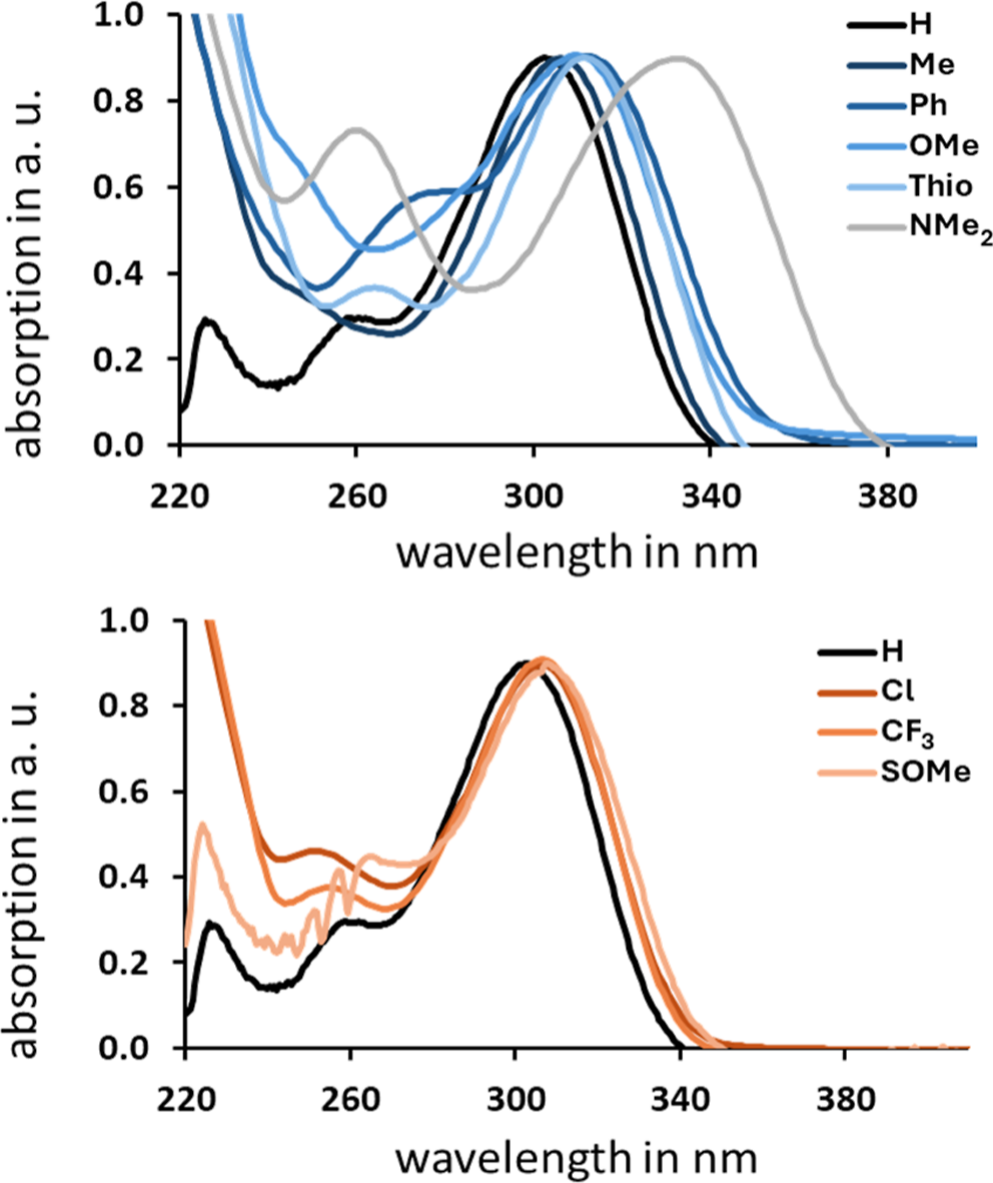
UV–Vis absorption spectra for the C3 arylated dihydroazaborinines synthesized using the Suzuki cross coupling protocol described in Scheme 2. The legend gives the *para* substituent of the aryl ring attached to C3, except for the thiophene substituent, which is directly linked to the dihydroazaborinine C3. The absorption maxima are normalized to 0.9 au.

**Table 2:** Absorption Maximum λ_max_ (in nm) of the Different C3 *p*-X-C_6_H_4_-Substituted Dihydroazaborinines in Cyclohexane Solution. The First Row Gives the *Para* Substituent of the Aryl Ring Attached to C3, except for Thiophene (**8i**), Which Is Directly Linked to the Dihydroazaborinine C3

X	**8a** H	**8b** Me	**8c** OMe	**8j** Ph	**8i**	**8e** NMe_2_	**8f** Cl	**8g** CF_3_	**8k** SOMe
λ_max_	302	306	310	312	312	333	306	310	309

In all examples studied, this appeared to be independent of whether the effect is electron donating or withdrawing. In most cases, the absorption spectrum changes just slightly, only for the most electron-donating (NMe_2_, **8e**) example a more significant shift occurs.

TD-B3LYP­(6-311+G­(d,p)) UV–Vis spectra are in good agreement with the experimental data, both qualitatively and quantitatively (see Supporting Information for λ_max,calc_). An analysis of the frontier orbitals of the investigated dihydroazaborinines reveals that, in particular, for compound **8e**, the HOMO–LUMO transition, which represents the longest-wavelength transition, involves charge transfer from the C3 substituents to the dihydroazaborinine core. In contrast, no such transition is observed for **8a**, and it is less pronounced for other derivatives (for the frontier orbitals of **8a**, **8c**, **8e**, and **8k**, see Supporting Information).

### Isomerization

Irradiations (280–400 nm) of 0.05 to 0.1 M solutions of the different dihydroazaborinines **8** in cyclohexane led to the exclusive formation of the corresponding Dewar isomers (for details, see Supporting Information). Notably, full conversion was achieved within remarkably short irradiation times of only 1–2 min.

The recorded NMR spectra of the photoproducts are in excellent agreement with those of previously reported Dewar isomers of dihydroazaborinines.^11,12^ Neither during the photoisomerization to the Dewar isomer nor upon continued irradiation do any additional signals appear in the NMR spectra beyond those attributable to the dihydroazaborinine and its Dewar isomer. Computations on the photoisomerization of the parent compound as well as substituted derivatives conclude that these processes occur through S_1_ states.^16,18–20^ In view of the analogous photochemistry of the dihydroazaborinines **8**, they likely follow the same mechanism.

Subsequent heating of the Dewar isomer recovered the dihydroazaborinines. Kinetic studies conducted by monitoring the thermal back-reaction via proton NMR spectroscopy at four different temperatures at least two times demonstrated first-order kinetics in all cases (Figure 4). These findings are consistent with previously published data on related dihydroazaborinines.^11,22^

**Figure 4:**
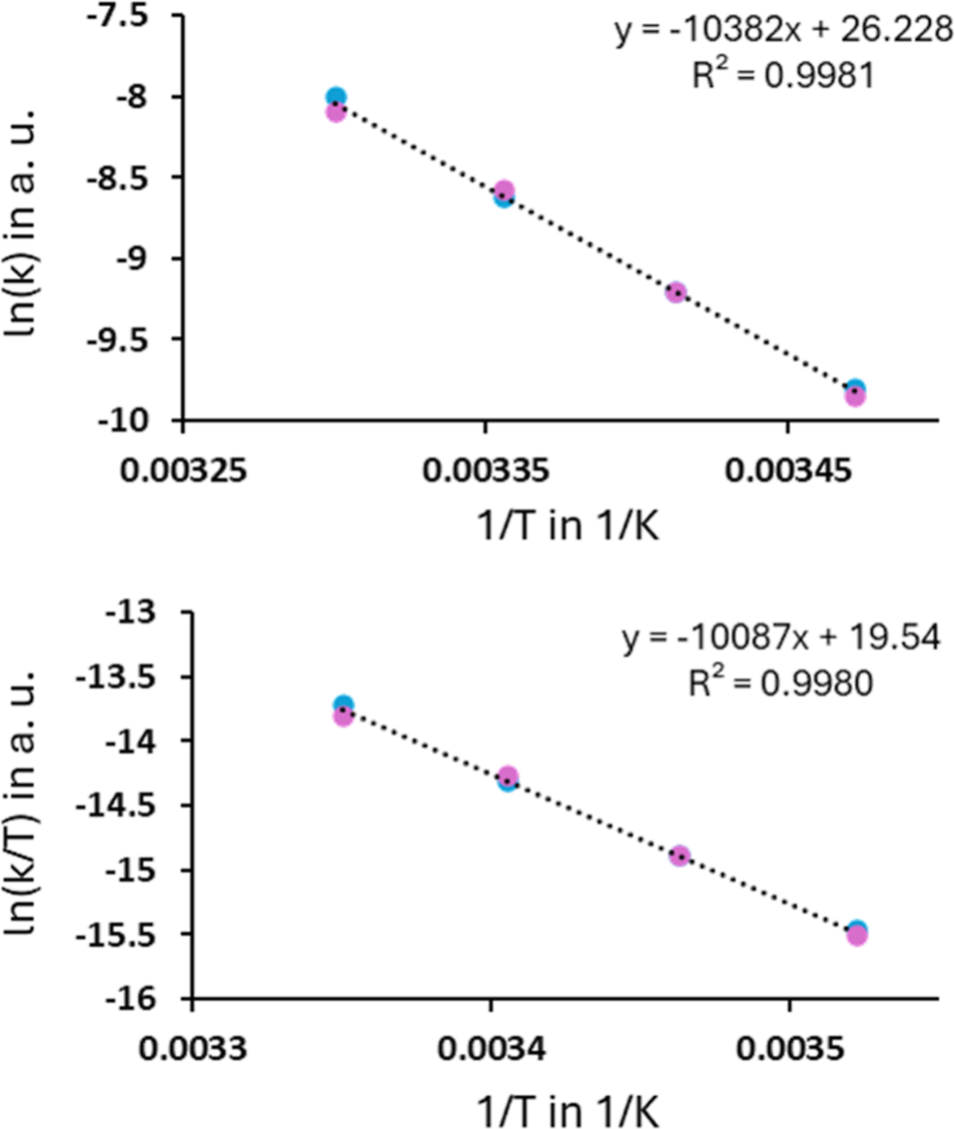
Arrhenius (top) and Eyring plot (bottom) for ring opening of the Dewar isomer of**8e**. The kinetics of the thermal ring opening were monitored by NMR spectroscopy at 288 K, 293 K, 298 K, and 303 K. Each temperature was measured twice. The unit of the rate constants used is s^–1^, as the reaction is first order.

Depending on the electronic effect of the substituent on C3, the lifetimes of the Dewar isomers change significantly, ranging from two days to a few hours at room temperature (Table 3). In comparison, the lifetime of the unsubstituted C3 analog is slightly below half a year at room temperature.^11,12^ A plausible explanation for this drastic decrease in lifetime could be a destabilization of the Dewar isomer due to substituent effects at C3 as discussed below. The activation barriers and lifetimes were derived via Arrhenius as well as Eyring treatments (Table 3).

**Table 3:** Arrhenius Activation Energy (*E*_A_) in kcal/mol, Reaction Rate (Extrapolated to 25 °C) in s^–1^ 10^–6^, and Lifetime (at 25 °C) in *d* of the Considered Dihydroazaborinines Were Calculated Based on the Arrhenius Plot. An Itemization into Enthalpy and Entropy Renders Possible via an Eyring Treatment of the Data. Δ*H* ^‡^ and Δ*G* ^‡^ Are Given in kcal/mol, Δ*S* ^‡^ Is Given in kcal/(mol K)

		**8a** H	**8b** Me	**8c** OMe	**8j** Ph	**8i**	**8e** NMe_2_	**8f** Cl	**8g** CF_3_	**8k** SOMe
Arrhenius	*E*_A_	22.93	22.10	22.65	23.47	21.60	20.63	21.06	22.40	21.32
	k25 °C	2.08	4.31	12.01	4.63	71.86	126.23	3.73	2.74	6.73
	*t*_1/2, 25 °C_	3.9	1.9	0.7	1.7	0.1	0.06	2.2	2.9	1.19
Eyring	Δ*H* ^‡^	22.27	21.46	22.03	22.85	21.00	20.04	20.43	21.76	20.73
	Δ*S* ^‡^	–0.009	–0.010	–0.006	–0.006	–0.006	–0.008	–0.014	–0.010	–0.006
	Δ*G* ^‡^	24.97	24.54	23.94	24.50	22.88	22.54	24.63	24.81	22.45

To gain more insights into the mechanism of the cycloreversion based on this trend, a Hammett analysis was carried out. Very good correlations were obtained for the electron-donating substituents using σ^+^ and for the electron-withdrawing substituents against σ^–^, although only a limited amount of data points is available for the latter (Figure 5).

**Figure 5:**
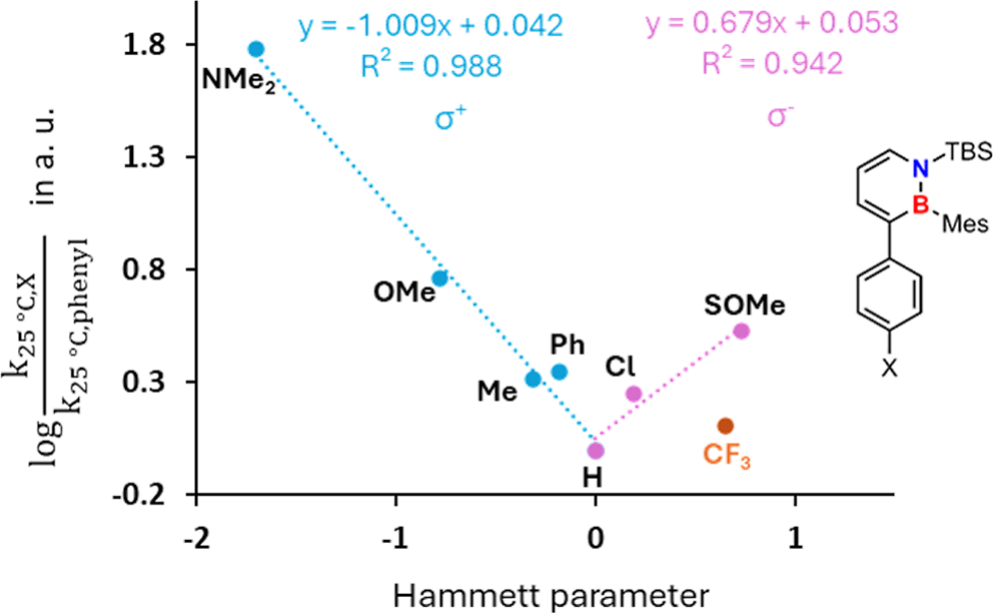
Hammett plot for the electron-rich dihydroazaborinines against σ^+^ and the electron-poor coupling products toward σ^–^. The annotation at the individual points refers to the position marked with an X in the dihydroazaborinine on the right.

For the latter, the trifluoromethyl derivative does not fit the slope and appears to have a significantly lower electron-withdrawing character than expected. This result was highly reproducible, as every temperature of the kinetic analysis was measured three times. As the Hammett reference systems (σ^+^ and σ^–^) consider structures with direct resonance between the reaction site and the substituent, the deviation of the CF_3_ system seems to be a consequence of the inductive nature of its electronic effect. All other examples we considered rather have a mesomeric effect or a weaker inductive effect than the CF_3_ group.^51^ Due to the pronounced inductive effect of the CF_3_ group, which appears not to be sufficiently transmitted across the π-system to the reaction center, its influence is overestimated by approximately a factor of 2.

The Hammett analysis evidently shows a change in slope between electron-rich and electron-poor species. Frequently found explanations for such behavior are a change in the rate-determining step,^52,53^ a change in mechanism,^54^ or changing overall contributions.^55,56^ A reasonable correlation can also be obtained using Creary parameters, as pointed out by a reviewer. However, computations did not provide evidence of the involvement of diradical intermediates (see Supporting Information for details).

We performed explorative computations using the B3LYP/6-311+G­(d,p) method (see Supporting Information for details) and identified a thermal ring-opening mechanism that occurs stepwise through two transition states and involves a shallow intermediate. This finding parallels the ring opening of parent dihydroazaborinine studied by Bettinger and Hauler,^21^ while for 1-(*tert*-butyldimethylsilyl)-2-mesityl-1,2-dihydro-1,2-azaborinine **1**, the C3 unsubstituted analog of the compounds **8a–k**, a concerted mechanism was found.^11^ In these studies, the B3LYP functional was demonstrated to be suitable for calculations of dihydroazaborinines, exhibiting only marginal deviations from CCSD­(T) calculations and experimental data.^10,11,21^ Toward the transition state, the C3 atom rotates significantly outward. The opposite C6 atom, however, does not move in the orbitally allowed conrotatory motion toward the center of the molecule. Instead, it only undergoes a very slight outward movement.^11^

The ring opening of **8a** can be described by a similar motion. Approaching the first transition state (**8a**_**TS1**_) and the intermediate state (**8a**_**IM**_), the central C3–C6 bond of the Dewar isomer breaks. This is accompanied by planarization of C3 into the plane of the prospective dihydroazaborinine ring, resulting in a B–C6–C5−C4 dihedral angle of 18.6° and a C3–C6 distance of 2.409 Å in **8a**_**IM**_. The C6–B distance is reduced by 0.194 Å (**8a**_**TS1**_) or 0.327 Å (**8a**_**IM**_) relative to that of the Dewar isomer (**8a**_***Dewar***_) (see Figure 6), leaving room for an interpretation as a B–C3–C6 three-center-two-electron (3c2e) bond. Such coordination is supported by natural bond orbital (NBO) analysis for **8a**_**IM**_ that identifies a three-center bond orbital among these atoms.

**Figure 6:**
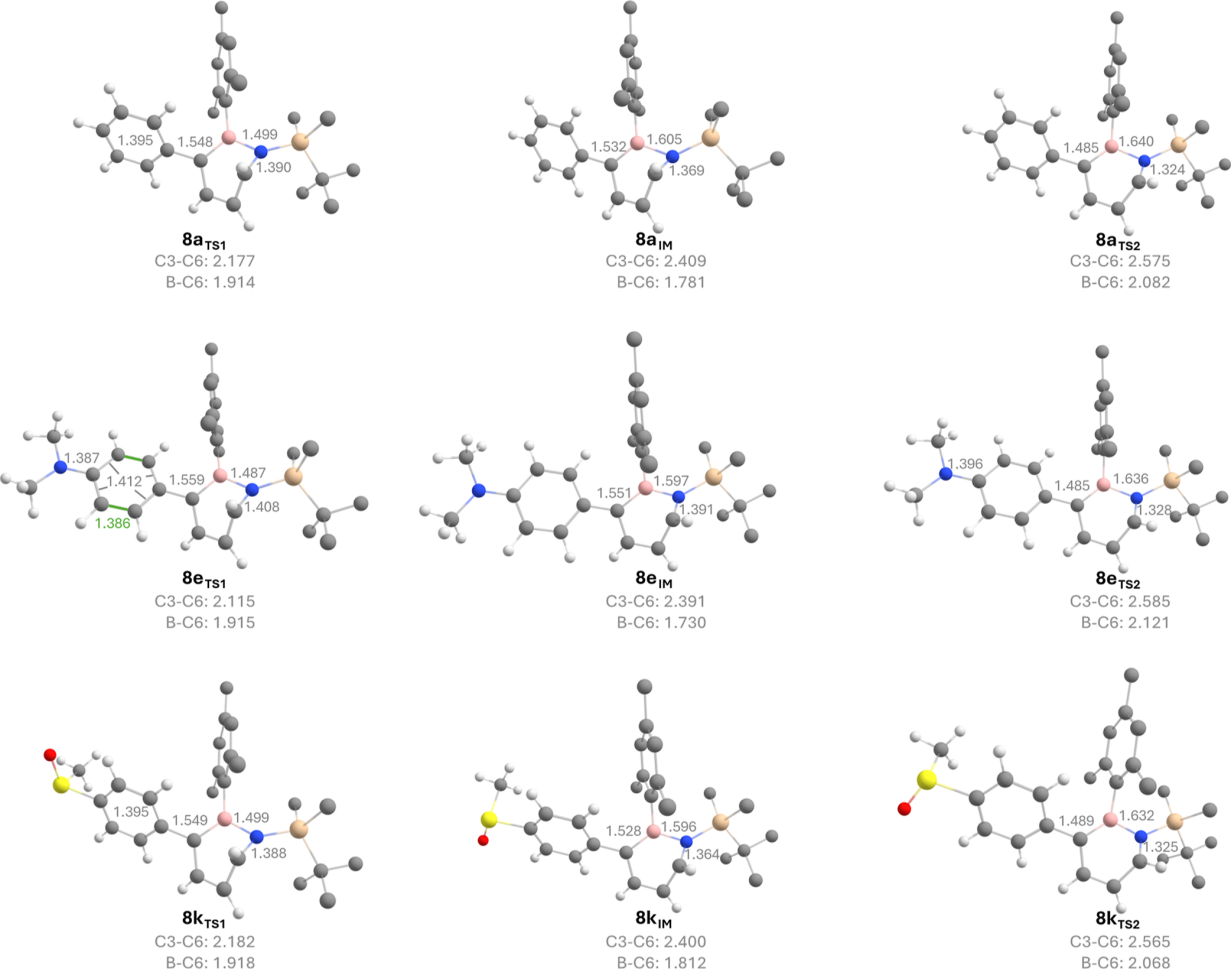
Geometries of IM, TS1, and TS2 computed at the B3LYP/6-311+G­(d,p) level of theory. C3–B, B–N, N–C6, C3–C6, and B–C6 distances are given in Å. For geometries of the dihydroazaborinines **8a**, **8e**, and **8k** as well as their Dewar isomers, see Supporting Information.

In contrast, the NBO analysis describes a direct π bond between C3 and C6 in **8a**_**TS1**_ and a lone vacancy (LV) located at the boron (see Table 4). In the second transition state **8a**_**TS2**_, the C6 atom is slightly rotated outward leading to an increased C3–C6 distance of 2.575 Å and a C3–C4–C5-C6 dihedral reduced by 3.6° compared to the intermediate (C3–C4–C5-C6 dihedral: 4.1°). Additionally, the B–C3 bond is shortened to 1.324 Å. Taking into consideration electron-donating (*p*-NMe_2_, **8e**) or -withdrawing (*p*-SOMe, **8k**) substituents in the *para* position of the C3 aryl substituent provided a similar picture (see Figure 6).

**Table 4:** APT Charges at C3 in the Course of the Thermal Ring Opening for the Dewar Isomers of **8a**, **8e**, and **8k** (Left). Occupancy of the B–C3–C6 3c2e Bond and Main Contributor to this Bond with Contribution in Percentage

		Dewar	TS1	IM	TS2	Aza			B–C3–C6 bonds		B–C3–C6 LV	
APT charge	NMe_2_	–0.2	0.1	0.7	–0.2	–0.2	occupancy	TS1	1.578	C3–C6 π	0.396	B
								IM	1.544	C6–B π	0.854	C3
	H	–0.3	–0.2	0.1	–0.4	–0.3		TS1	1.535	C3–C6 π	0.426	B
								IM	1.825	2e3c		
	SOMe	–0.3	–0.3	–0.01	–0.4	–0.3		TS1	1.532	C3–C6 π	0.424	B
								IM	1.818	2e3c		

The *p*-NMe_2_ substituent results in a lowering of the energy of **8e**_**TS1**_ by 4.4 kcal/mol and of **8e**_**IM**_ by 5.18 kcal/mol compared to that of **8a** (Figure 7). For the *p*-SOMe electron-withdrawing group (**8k**), a negligible change (0.5 kcal/mol) in the relative energy levels of **8k**_**TS1**_ and **8k**_**IM**_ is computed in relation to **8a**. Independent of the additional *para* electron-donating or -withdrawing group, the second transition state remains highest in energy (see Figure 7). The energy levels of these transition states are 27.3 kcal/mol (**8a**_**TS2**_), 24.9 kcal/mol (**8e**_**TS2**_), and 25.7 kcal/mol (**8k**_**TS2**_). This trend is in good agreement with the experimentally determined Gibbs free energies of activation, and the absolute ΔG^‡^ values differ from the experimental ones by approximately 1.4–2.4 kcal/mol (see Table 3).

**Figure 7:**
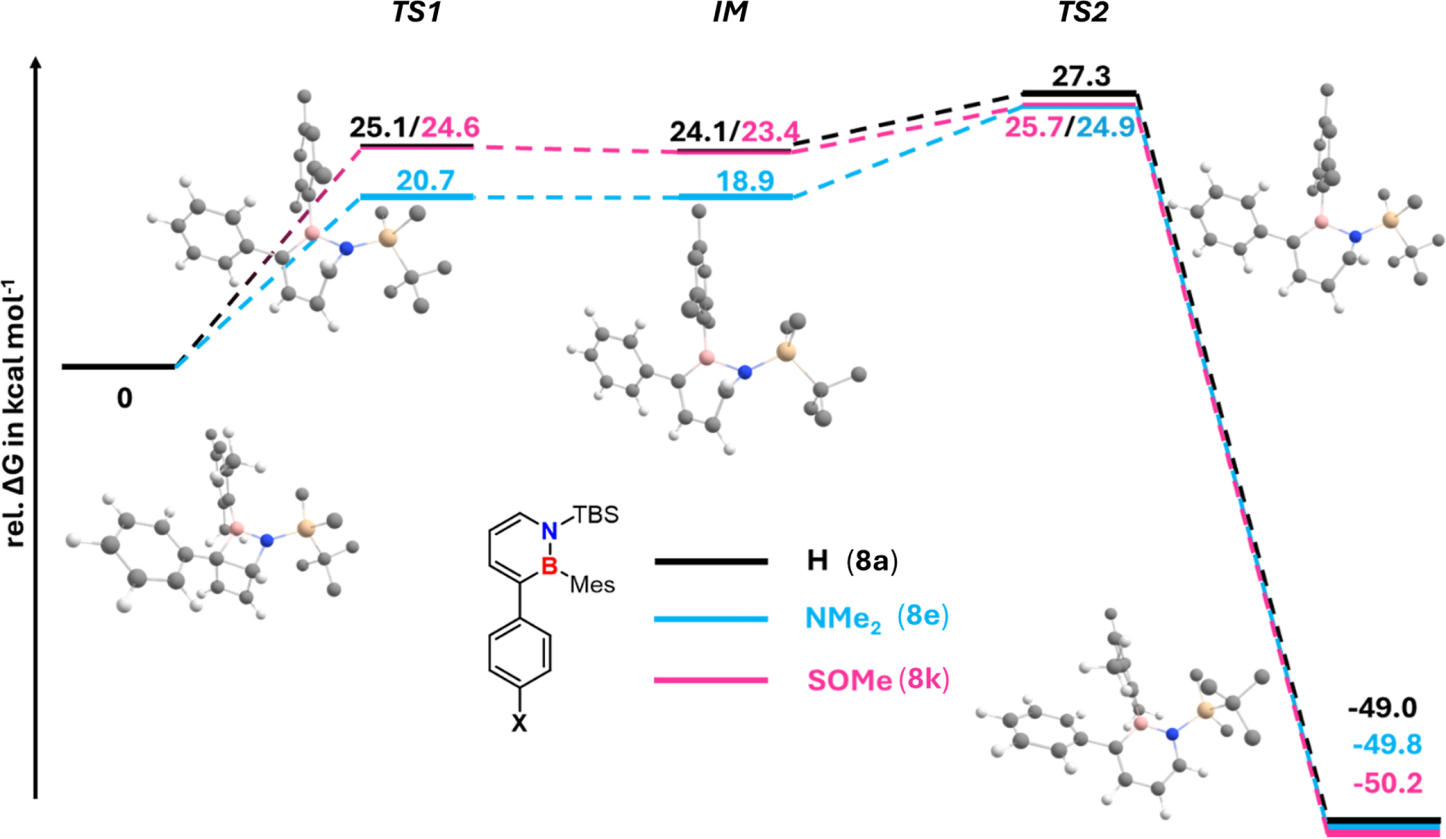
Gibbs energy (at 298 K) diagram for the thermal ring-opening reaction calculated for **8a** (black), **8e** (blue), and **8k** (pink) at the B3LYP/6-311+G­(d,p) level of theory. Energies are given in kcal/mol relative to the corresponding Dewar isomer.

Following Kozuch et al., we identified the intermediate as part of the rate-determining zone in the transformation of the Dewar isomers to the dihydroazaborinines.^57–59^ This is because its formation is the elementary step with the highest energy barrier to be crossed.^57^ Hence, we focus on the variation of computed atomic polar tensor (APT) charges toward the formation of the intermediate. In the case of the electron-donating *p*-NMe_2_ substituent (**8e**), positive charge is built up at the C3 position toward the intermediate. This charge accumulation is contrary to the inherent polarity of the molecule that is induced by the B–N bond. Instead of the B–C3–C6 3c2e bond, a π C6–B bond with an occupancy of 0.854 e^–^ is formed in **8e**_**IM**_. In the intermediate (**8e**_**IM**_), this leads to a lone vacancy at C3 with an occupancy of 0.854 e^–^, which is not observed in the case of the 3c2e interaction in **8a**_**IM**_ and **8k**_**IM**_ (see Table 4). The distinctly pronounced positive charge at C3 appears to be efficiently stabilized by the EDG at this position.

In contrast, the electron-withdrawing substituent *p*-SOMe (**8k**) preserves an approximately constant negative charge at C3 during the initial step of the thermal ring opening to the first transition state (see Table 4) in accordance with the small sensitivity parameter ρ = 0.679.

The C3–C6 π coordination and B–C3–C6 3c2e coordination are present in **8k**_**TS1**_ and **8k**_**IM**_, respectively. In the second transition state, all molecules have a negatively polarized C3 and the NBO analysis points to a partial B–C3 double bond. This π-bond has the highest occupancy for the EDG (**8e**_**TS2**_: 1.595 e^–^), followed by **8a**_**TS2**_ (1.569 e^–^) and the EWG (**8k**_**TS2**_: 1.554 e^–^). Based on the Lewis structures derived from the NBO analysis and the APT charges at different stages of the reaction, we suggest a change in the electronic structure of the intermediate as illustrated in Scheme 4. EDG stabilizes the resonance form with positive charge on C3 in agreement with a dominant C6–B coordination and the lone vacancy at C3 in the NBO. In contrast, EWG stabilizes the negative charge on C3. In this way, both EDG and EWG accelerate the ring opening, in agreement with the Hammett analysis (Figure 4).

**Scheme 4:**
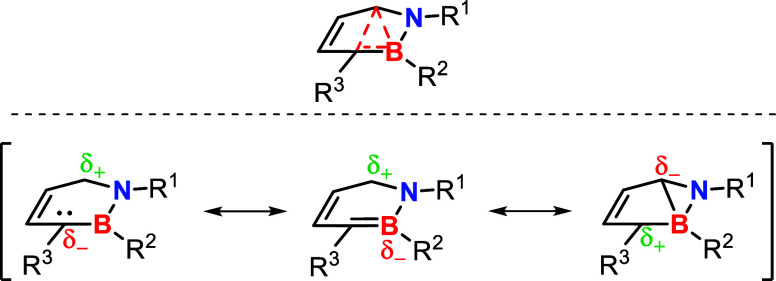
Different Mesomeric Representations of the 3c2e Bond That Evolves toward the Intermediate of the Thermal Ring-Opening Reaction

As the Hammett plot already indicated a correlation using reference systems where a positive (σ^+^) or negative (σ^–^) charge is built up, we suppose that the Hammett analysis reveals how the opposed charges at C3 become pronounced, depending on the electronic effect of the substituent, and thus change the electronic mechanism of the reaction.

## Conclusion

We developed a Suzuki cross coupling protocol for brominated dihydroazaborinines, which tolerates electron rich and poor, as well as sterically demanding boronic acids adjacent to the sterically demanding boron-bound mesityl group. GC-FID and upscaling experiments prove very good yields of the reactions. Depending on the electronic nature of the *p*-X-aryl substituent, a bathochromic shift of the absorption spectrum occurs in line with an increasing electronic effect of *p*-X-aryl. The absorption maxima and onsets of all investigated dihydroazaborinines remain in the UV range. All 11 synthesized dihydroazaborinines isomerize exclusively to the Dewar isomer upon UV-irradiation. Moreover, they cleanly undergo a thermal back reaction. NMR kinetic experiments revealed a relation between the activation barrier of this electrocyclic ring opening and the electronic effect of the C3 *p*-X-Ar substituent. The reaction is accelerated by EDGs and EWGs. Through a Hammett analysis and quantum chemical calculations, this behavior can be attributed to the switching of polarity of C3 in the rate-determining state, the intermediate, and a resulting change in the electronic mechanism of the reaction. In the broader context, the work shows that the storage time of the dihydroazaborinine MOST system can be addressed by judicious choice of the electronic effect of the C3 substituent.

## Supplementary Material



## Notes

Experimental details, NMR, MS and UV–vis spectral data, kinetic analysis data for the thermal ring opening, substituent parameters, computational details, Cartesian coordinates, GC-FID calibrations, and crystal structure data (PDF)

## Abbreviations

EWG: electron-withdrawing group
EDG: electron-donating group
APT: atomic polar tensor
IM: intermediate
TS1: first transition state
TS2: second transition state.
