# Supplementary material for: Mechanistic Insight into the Thermal Ring Opening of the Dewar Isomer of 1,2-Dihydro-1,2-azaborinines
Source: JACS Au. 2025 Sep 19;5(10):5006–16. doi: 10.1021/jacsau.5c00923 (PMC12569699; doi:10.1021/jacsau.5c00923)
Supplement: Supplementary file 1 [file au5c00923_si_001.pdf]

Supporting Information

Mechanistic insights into the thermal ring opening of the Dewar isomer of 1,2-Dihydro-1,2-azaborinines

Sonja M. Biebl,[a] Paul Ziemann,[a] Markus Ströbele,[b] Holger F. Bettinger[a,*]

[a] Institut für Organische Chemie, Eberhard Karls Universität Tübingen, Auf der Morgenstelle 18, 72076 Tübingen, Germany, E-Mail: holger.bettinger@uni-tuebingen.de

[b] Institut für Anorganische Chemie, Eberhard Karls Universität Tübingen, Auf der Morgenstelle 18, 72076 Tübingen, Germany

Table of Contents

1. Methods 4

2. Analytics 4

3. Synthesis 6

3-Bromo-1-(*tert*-butyldimethylsilyl)-2-mesityl-1,2-dihydro-1,2-azaborinine 6

Optimization of the catalytic system 11

Substratescope (boronic acid) 12

100 µmol experiment (8a-8j) 13

Upsacaling experiment: 1-(*tert*-Butyldimethylsilyl)-2-mesityl-3-(4-methoxyphenyl)-1,2-dihydro-1,2-azaborinine (8c) 54

Synthesis of two and trifold brominated dihydroazaborinines 2-5 55

Oxidation of 1-(*tert*-Butyldimethylsilyl)-2-mesityl-3-(4-methylthiophenyl)-1,2-dihydro-1,2-azaborinine (8d) to 1-(*tert*-Butyldimethylsilyl)-2-mesityl-3-(4-methlsulfoxidphenyl)-1,2-dihydro-1,2-azaborinine (8k) 68

Synthesis of pentamethylphenyl lithium (14) 72

Synthesis of 1-(*tert*-butyldimethylsilyl)-2-pentamethylphenyl-1,2-dihydro-1,2-azaborinine (11) 74

Synthesis of 3-Chloro-1-(*tert*-butyldimethylsilyl)-2-pentamethylphenyl-1,2-dihydro-1,2-azaborinine (6) 78

Synthesis of 5-bromo-3-chloro-1-(*tert*-butyldimethylsilyl)-2-mesityl-1,2-dihydro-1,2-azaborinine (8) 86

Synthesis of 3-chloro-1-(*tert*-butyldimethylsilyl)-2-pentamethylphenyl-5-phenyl-1,2-dihydro-1,2-azaborinine (9) – Route A 90

Synthesis of 3-chloro-1-(*tert*-butyldimethylsilyl)-2-pentamethylphenyl-5-phenyl-1,2-dihydro-1,2-azaborinine (9) – Route B 90

4. Irradiation experiments 95

NMR data of $8a_{Dewar}$ 96

NMR data of $8b_{Dewar}$ 100

NMR data of $8c_{Dewar}$ 104

NMR data of $8e_{Dewar}$ 108

NMR data of $8f_{Dewar}$ 112

NMR data of $8g_{Dewar}$ 116

NMR data of $8h_{Dewar}$ 120

NMR data of $8j_{Dewar}$ 124

NMR data of $8k_{Dewar}$ 128

5. Kinetic experiments 131

Sample preparation 131

Experimental details 131

Time dependence (1. order kinetic) 139

Hammett and Creary Parameters ... 140
Creary Plot ... 140
7. Crystal structures ... 142
9. GC-FID Calibrations ... 158
8. Computations ... 162
Relative energies of the optimized structures (B3LYP/6-311+G(d,p)) ... 162
APT charges ... 163
3c2e bond ... 164
Frontier orbitals of 8a, 8c, 8e and 8k ... 166
Absorption maxima ... 169
Coordinates 8a ... 170
IRC 8a ... 180
Coordinates 8e ... 181
IRC 8e ... 191
Coordinates of 8k ... 192
IRC 8k ... 202
References ... 203

1. Methods

Unless otherwise noted, all experiments were carried out under inert conditions using Schlenk technique with argon or nitrogen as the protective gas or in a glove box (UNIlab Pro, MBraun). Glassware was dried before use by heating. Commercial triethylamine (water content ~ 0.2%) was refluxed over KOH for two hours and then distilled before use. The remaining chemicals used were employed as received from the manufacturer without further purification. Anhydrous solvents were obtained from Thermo Fisher Scientific Inc., Acros Organics B.V. B.A., Sigma-Aldrich or, in the case of dichloromethane, diethyl ether, n-hexane, tetrahydrofuran and toluene, from an SPS-800 solvent drying system by the manufacturer MBraun.

2. Analytics

NMR Spectroscopy: The acquisition of ^{1}H, ^{13}C-{^{1}H}, and ^{11}B-{1H} NMR spectra were performed on a Bruker Advance III HD 400 MHz instrument (^{1}H spectra) at 101 MHz (^{13}C-{^{1}H} spectra), and 128 MHz (^{11}B-{1H} spectra). Complementarily, spectra of these nuclei were recorded on a Bruker Advance III HD 300 MHz NanoBay at 300 MHz (^{1}H spectra), 76 MHz (^{13}C-{^{1}H} spectra), and 96 MHz (^{11}B-{1H} spectra). For high-temperature NMR measurements (kinetic experiments), a Bruker Avance III HDX 600 spectrometer was employed with a measuring frequency of 600 MHz (^{1}H spectra), 151 MHz (^{13}C-{^{1}H} spectra) or 192 MHz (^{11}B-{1H} spectra) and on a Bruker Avance III HDX 700 with a measuring frequency of 700 MHz (^{1}H spectra) or 176 MHz (^{13}C-{^{1}H} spectra). All obtained NMR spectra were referenced to the solvent peak. Deuterated dichloromethane (5.32 ppm), benzene (7.16 ppm), chloroform (7.26 ppm), cyclohexane (1.38 ppm), or tetrachloroethane (5.91 ppm) from Sigma-Aldrich or Deutero were used for referencing.

MPLC Chromatography: Column chromatographic purifications were performed using the puriFlash 430 in combination with pre-packed silica gel columns (particle size: 30 µm) from Interchim. Detection was carried out using a puriFlash One Series UV detector (DAD 200-600 nm) from the same manufacturer.

All solvents used were of HPLC-grade purity. Solvent mixtures are indicated as V/V ratio.

GPC Chromatography: The size exclusion chromatography was conducted on a Recycling Preparative HPLC and GPC (LaboACE LC-7080 Plus II) device and a JAIGEL-2.5 HR Plus column both from Japan Analytical Industry Co.

UV/VIS Spectroscopy: For determining the absorption maxima of the presented 1,2-substituted 1,2-dihydro-1,2-azaborinines, a Lambda 1050 UV/VIS/NIR spectrometer (Perkin Elmer) with a 3D WB detection module was used, operated with the accompanying UV Win Lab software (Version 2.0.2). The wavelength range covered was from 250 nm to 850 nm. All solvents used had a cut-off wavelength below 250 nm and were water-free.

Irradiation experiments: As light source an Osram HBO-500-W/2 high pressure mercury lamp in an Oriel housing with quartz optics and a dichroic mirror (280 – 400 nm) was applied. No additional filters were used.

The samples for the kinetic studies prepared in quartz glas J.-Young-NMR tubes as the reaction vessel.

X-Ray Crystallography: Crystaly suitable for X-Ray diffraction were grown by vapour diffusion with *n*-hexane and dichloromethane at room temperature. Single crystals were selected, coated with Parabar 10312 and fixed on a microloop.

Data were collected on a XtaLAB Synergy, Dualflex, HyPix diffractometer using ω scans with Cu K_{α} radiation. The crystal was kept at a steady temperature during data collection. The diffraction pattern was indexed and the total number of runs and images was based on the strategy calculation from the program CrysAlisPro 1.171.42.49, which was also used for refining the unit cell. The structure was solved with the **ShelXT** 2018/2 solution program{Sheldrick, 2008 #143;Dittrich, 2011 #143}[1] using dual methods and by using **Olex2** 1.5-ac5-024 as the graphical interface.[2] The model was refined with **olex2.refine** 1.5-ac5-024 using full matrix least squares minimisation on F^2.[3]

GC-FID analysis: The measurement was carried on an Agilent 7820A system with an Agilent 19091J-431 column (30 m × 320 µm × 0.25 µm), utilizing H_2 as the carrier gas. The programs used heated from 50 °C to 280 °C within 15 min or from 50 °C to 300 °C in 36 min.

Mass spectrometry: High resolution mass spectra were recorded on a HR-ESI/APCI-TOF device (maXis 4G, Bruker) or a HR-ESI-Orbitrap-MS (Q exactive HF, Thermo Scientific) for ESI ionization. The sample was dissolved in dichloromethane and injection was carried out using a syringe pump. For ASAP ionization a Exactivem Plus Orbitrap MS from Thermo Scientific was used. The sample was dissolved in dichloromethane.

3. Synthesis

1-(*tert*-Butyldimethylsilyl)-2-mesityl-1,2-dihydro-1,2-azaborinine (**1**) was synthesized according to Richter *et al.*[4]

3-Bromo-1-(*tert*-butyldimethylsilyl)-2-mesityl-1,2-dihydro-1,2-azaborinine

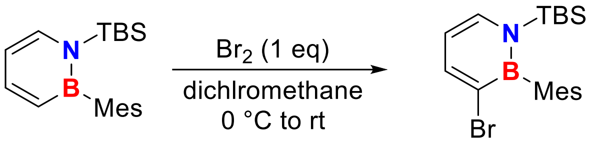

1 **2**

The synthesis is based on a procedure by Lamm *et al.*[5] 1-(*tert*-butyldimethylsilyl)-2-mesityl-1,2-dihydro-1,2-azaborinine (**1**) (1 g, 3.2 mmol, 1 eq) was solved in dichloromethane (10 mL) and cooled to 0 °C. A solution of bromine (0.09 mL, 3.5 mmol, 1.1 eq) in dichloromethane (10 mL) was added dropwise over 1 h. The reaction mixture was stirred for 30 minutes at 0 °C. The solution was allowed to reach room temperature and stirred for another 30 minutes, before a saturated solution of Na_2S0_3 (20 mL) was added. The aqueous layer was extracted three times with *n*-hexane (15 mL) and the combined organic layers were dried over $MgSO_4$. After removing of the solvent, the crude product was purified by column chromatography (silica, *n*-hexane/dichloromethane gradient). The product was obtained as colorless solid/crystals (0.8774 g, 70%).

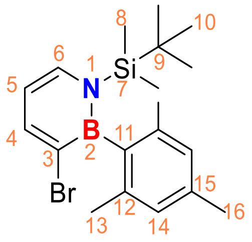

2

$C_{19}H_{29}BBrNSi$ (390.25 g/mol)

^{1}H-NMR (400 MHz, **CD_2Cl_2**): δ = 7.89 (dd, $^3J_{HH}$ = 7.18 Hz, $^4J_{HH}$ = 0.86 Hz, 1H, H-4), 7.48 (dd, $^3J_{HH}$ = 6.78 Hz, $^4J_{HH}$ = 0.86 Hz, 1H, H-6), 6.80 (s, 2H, H-14), 6.31 (ps. t, J = 6.89 Hz, 1H, H-5), 2.29 (s, 3H, H-16), 2.06 (s, 6H, H-13), 0.92 (s, 9 H, H-10), -0.01 (s, 6H, H-8) ppm.

^{13}C-{^{1}H}-NMR (100 MHz, **CD_2Cl_2**): δ = 144.8 (C4), 139.4 (C12/C15), 138.7 (C6), 137.4(), 127.2 (C14), 111.8 (C5), 27.6 (C10), 22.9 (C13), 21.4 (C16), 19.5 (C9), -3.0 (C8) ppm.

^{11}B-{^{1}H}-NMR (128 MHz, **CD_2Cl_2**): δ = 39.5 ppm.

HR-MS (APCI): m/z calc. For [M+H]+ 254.20776, found 254.20730.

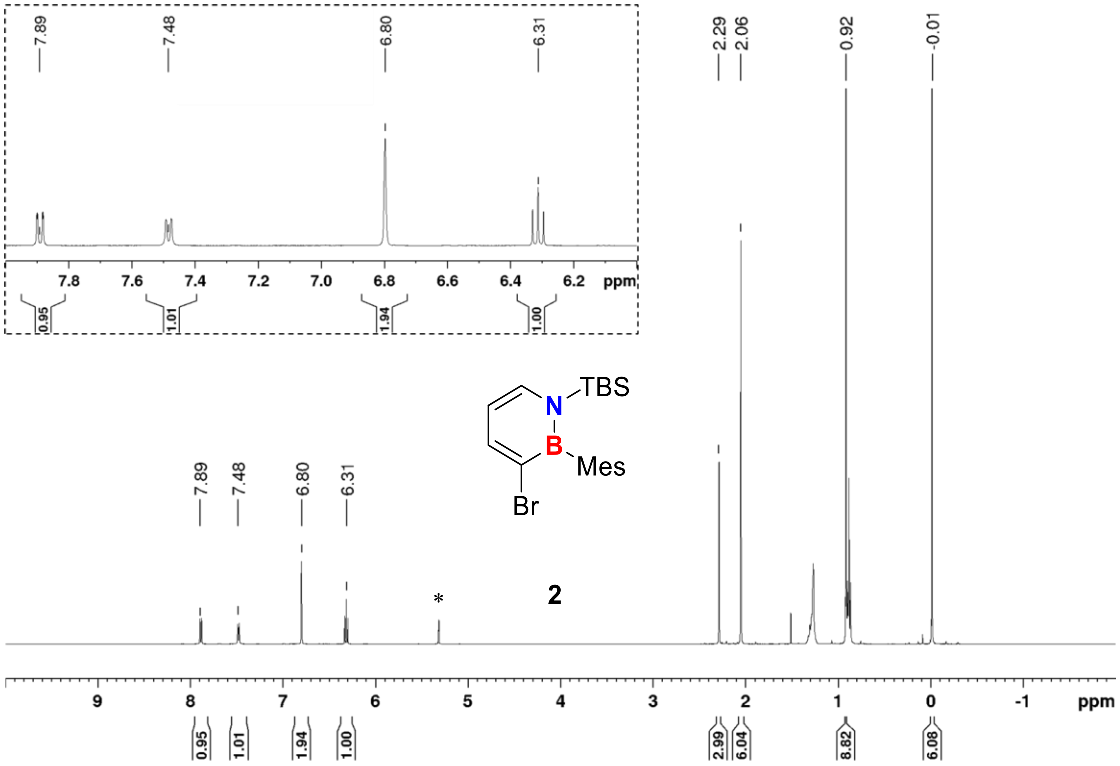

Figure S1. ^{1}H-NMR spectrum of compound **2** in CD_2Cl_2 measured at a 400 MHz spectrometer. The enlarged section shows the region between 6 and 8 ppm for a better visibility of the aromatic signals. The solvent signal is marked with an asterisk.

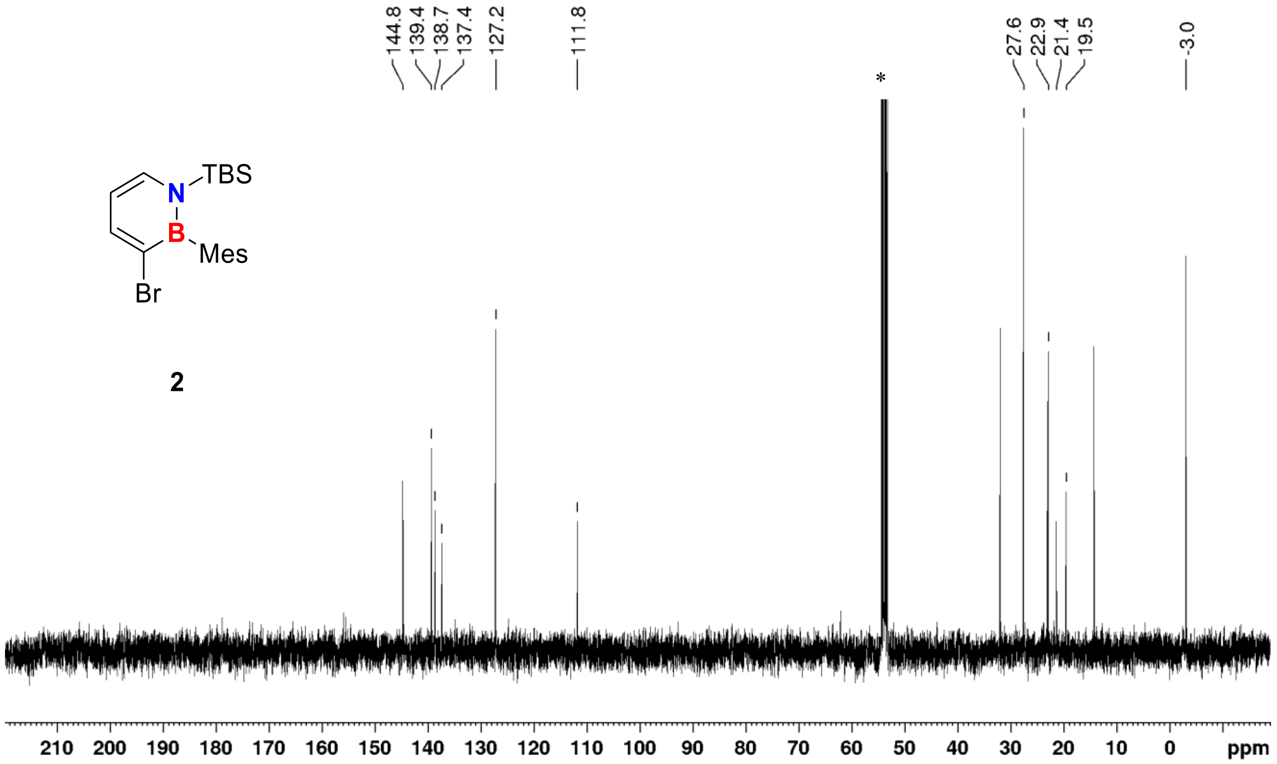

Figure S2. ^{13}C-{^{1}H}-NMR spectrum of compound **2** in CD_2Cl_2 measured at a 400 MHz spectrometer. The solvent signal is marked with an asterisk.

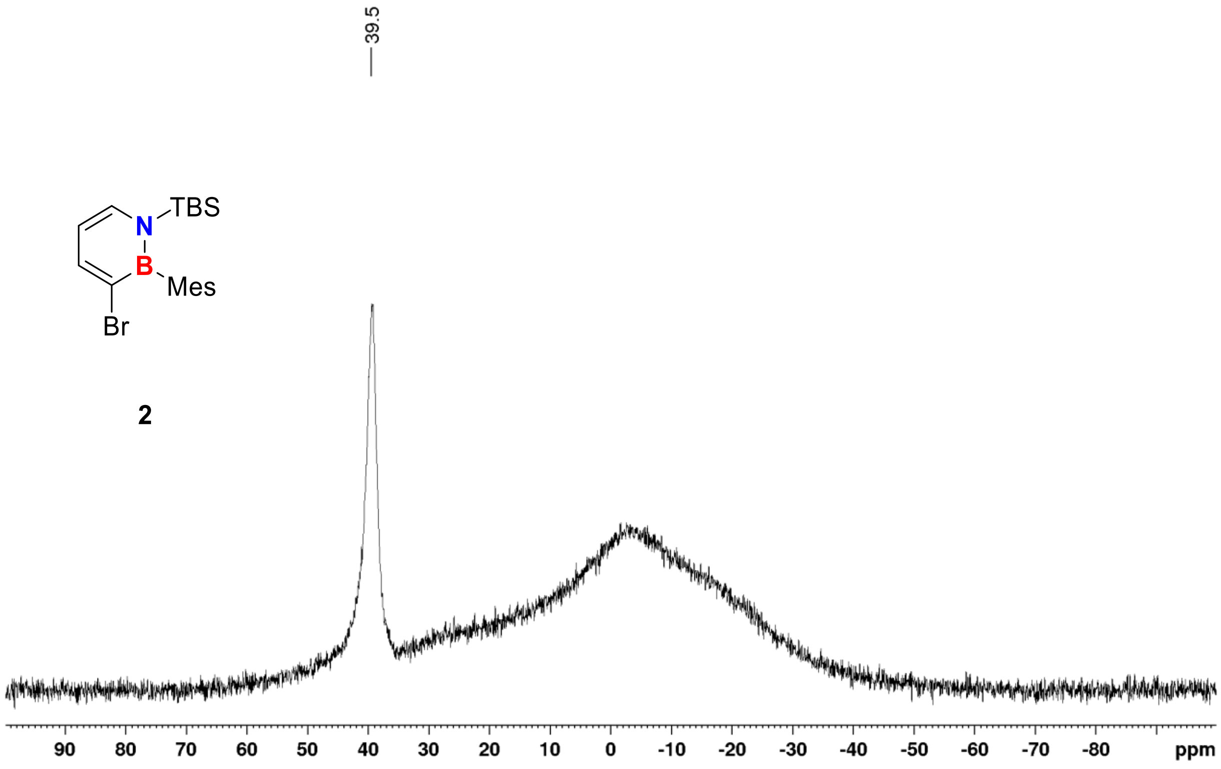

Figure S3. ^{11}B-{^{1}H}-NMR spectrum of compound **2** in CD_2Cl_2 measured at a 400 MHz spectrometer. The broad signal between -30 and 30 ppm) is due to the borosilicate glass of the NMR tube.

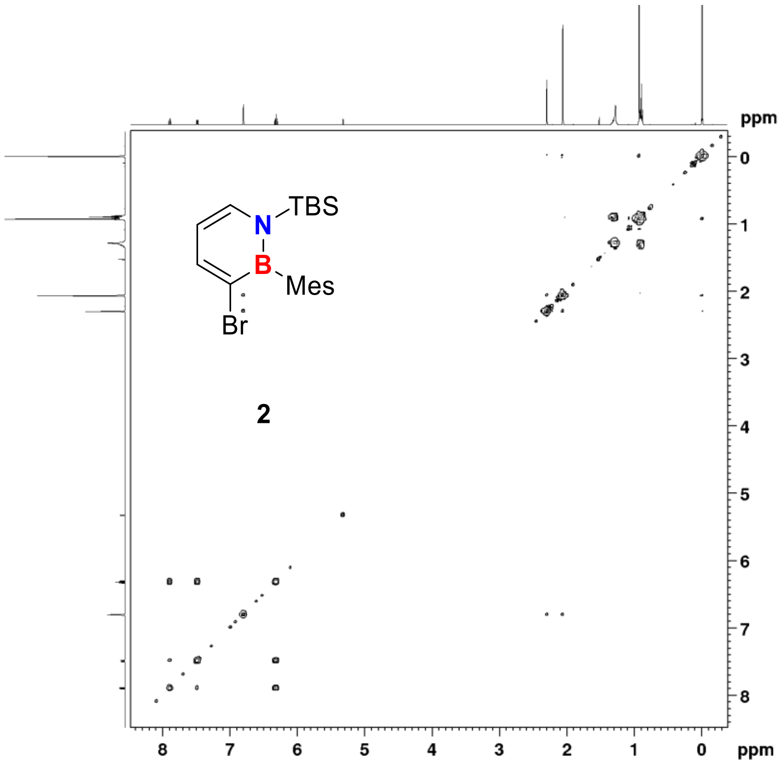

Figure S4. ^{1}H-^{1}H-COSY-NMR spectrum of compound **2** in CD_2Cl_2 measured at a 400 MHz spectrometer.

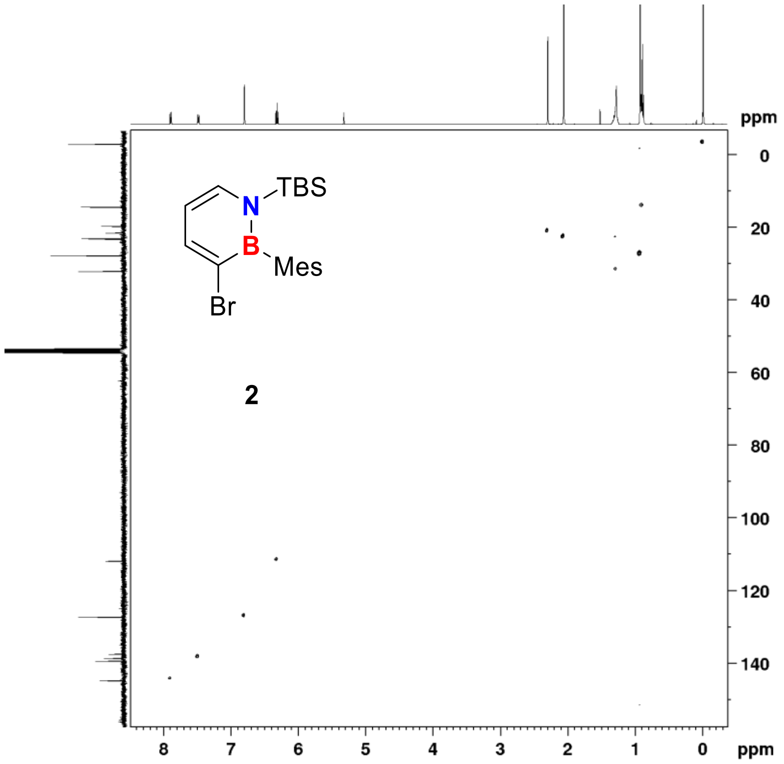

Figure S5. ^{1}H-^{13}C-HSQC-NMR spectrum of compound **2** in CD_2Cl_2 measured at a 400 MHz spectrometer.

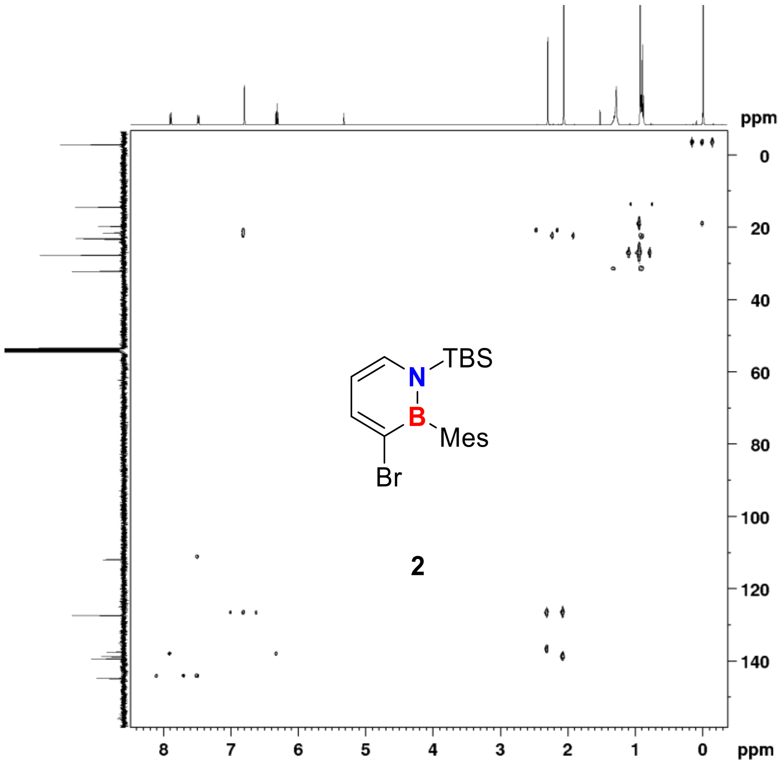

Figure S6. ^{1}H-^{13}C-HMBC-NMR spectrum of compound **2** in CD_2Cl_2 measured at a 400 MHz spectrometer.

Optimization of the catalytic system

For the optimization of the catalytic system dihydoazaborinine **2** (1 eq) and phenyl boronic acid (1.3 eq) were chosen as the model reaction.

Table S1. Screening and optimization of the catalytic system for the Suzuki cross coupling of C-3 brominated dihydroazaborinines.

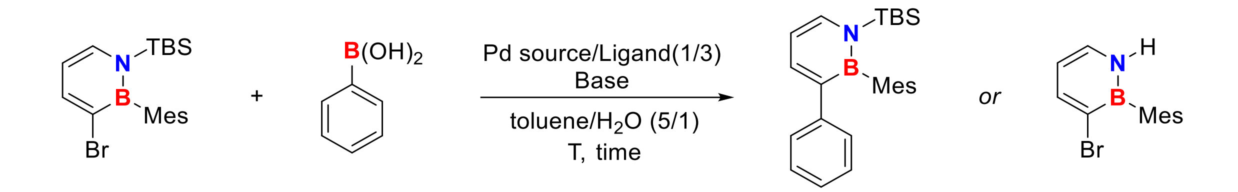

1.3-1.5 eq

2 **8a** **9**

Entry		Pd source		Ligand	Base		T/°C	t/h	**2/8a/9**
standard	[6]	$Pd_2(dba)_3$	*3 mol%*	BIDIME	Cs_2CO_3	*2 eq*	50	20	0/quant/0
1[a, c]	[7]	$Pd(PPh_3)_2Cl_2$	*1 mol%*	-	K_2CO_3		80	16	quant/0/0
2[b, c]	[8, 9]	$Pd(OAc)_2$	*1 mol%*	XPhos	-		60	18	0/0/0
3[a, c]	[10]	$Pd(OAc)_2$	*1 mol%*	SPhos	K_3PO_4		105	16	0/0/quant
4[a, c]	[10]	$Pd(OAc)_2$	*1 mol%*	dppe *or* dppp	K_3PO_4	*3 eq*	105	16	0/0/quant
5[a, c]	[10]	$Pd(OAc)_2$	*1 mol%*	dppb	K_3PO_4	*3 eq*	105	16	0/0/50
6[d]	[11]	$Pd(PCy_3)G2$	*5 mol%*	-	-		60	18	quant/0/0
8d	[11]	PdXPhosG2	*5 mol%*	-	-		60	18	quant/0/0
8[d]	[11]	$Pd(P^tBu_3)G2$	*5 mol%*	-	-		60	18	quant/0/0
9[a]	[12]	$Pd(P^tBu_3)G2$	*4 mol%*	-	K_2CO_3	*3 eq*	60	18	0/0/0
10[e]	[12]	$Pd(P^tBu_3)G2$	*4 mol%*	-	Cs_2CO_3	*3 eq*	60	16	0/0/0
11[a,]	[10]	$Pd(OAc)_2$	*1 mol%*	AntPhos	K_3PO_4	*3 eq*	105	16	0/traces/
12[c]	[10]	$Pd_2(dba)_3$		AntPhos	Cs_2CO_3		40	20	0/5/0
13[a]	[10]	$Pd(OAc)_2$	*1 mol%*	S-BIDIME	K_3PO_4	*3 eq*	40	16	0/2/0
14	[6]	$Pd_2(dba)_3$		S-BIDIME	$NaHCO_3$		40	20	0/40/0
15	[6]	$Pd_2(dba)_3$		S-BIDIME	Cs_2CO_3		40	20	0/85/0
16	[6]	$Pd_2(dba)_3$		S-BIDIME	Cs_2CO_3		70	20	0/50/0

[a] only toluene was used as the solvent; [b] MeCN/H_2O (3/2) was used as the solvent; [c] a 1/2 ratio of catalyst/ligand was used; [d] THF was used as the solvent; [e] Dioxane/ H_2O (3/2) was used as the solvent

Substratescope (boronic acid)

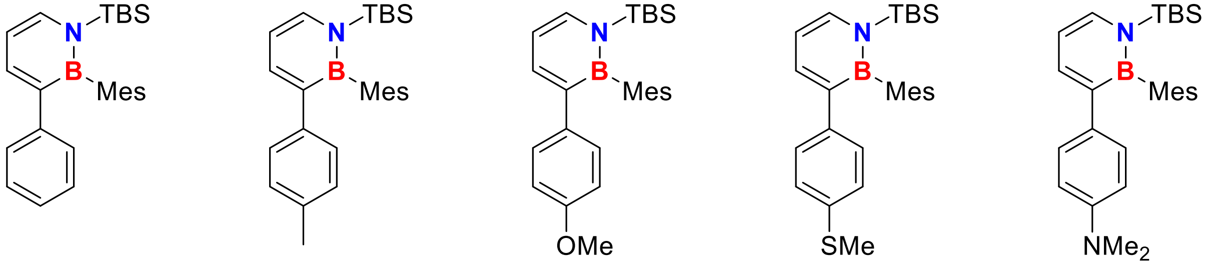

8a **8b** **8c** **8d** **8e**

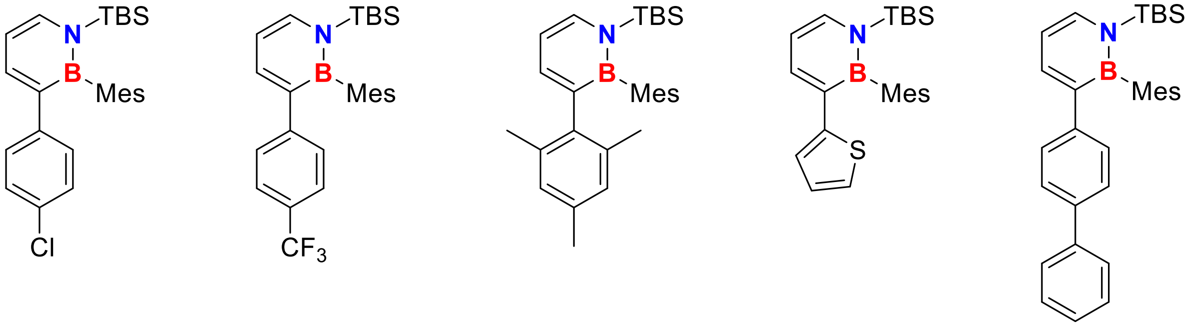

8f **8g** **8h** **8i** **8j**

Figure S7. Scope of C3-functionalized dihydroazaborinines synthesizes *via* Suzuki cross coupling of compound **2** with different boronic acids.

100 µmol experiment (8a-8j)

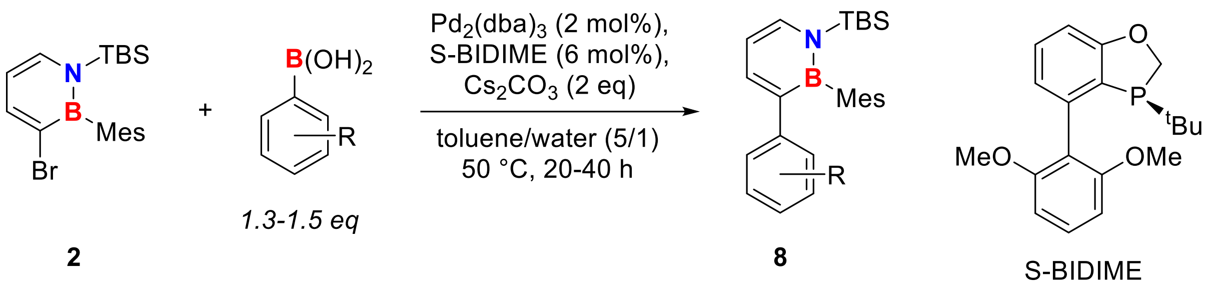

The synthesis was carried out according to a procedure by Yang *et al*. with modifications.[6]

3-Bromo-1-(*tert*-butyldimethylsilyl)-2-mesityl-1,2-dihydro-1,2-azaborinine (0.2977 g, 0.76 mmol, 1 eq), 4-methoxyphenylboronic acid (0.150 g, 1 mmol, 1.3 eq), dry Cs_2CO_3 (0.495 g, 1.5 mmol, 2 eq), $Pd_2(dba)_3$ (14 mg, 0.015 mmol, 2 mol%) and S-BIDIME (12.6 mg, 0.038 mmol, 6 mol%) were solved in dry, degassed toluene (12 mL) and degassed water (2.5 mL). The reaction mixture was allowed to stir for 16 h at 50 °C. The reaction was allowed to cool to room temperature and pentadecane was added with a *Hamilton* syringe as internal standard. The conversion was determined *via* GC-FID.

For the GC-FID calibration and the NMR and HR-mass characterization all compounds **8** were isolated according to the following procedure. Distilled water (2 mL) was added and the aqueous layer was extracted three times with *n*-hexane (2 mL). The combined organic layers were dried over $MgSO_4$. After removing of the solvent, the crude product was purified by column chromatography (silica, n-hexane/dichloromethane gradient). The product was obtained as colorless solid in the case of **8a-8d** and **8f** to **8j**. Compound **8e** was a pale yellow solid.

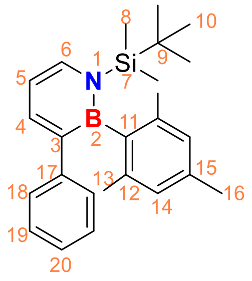

8a

$C_{25}H_{34}BNSi$ (387.26 g/mol)

GC-FID: t_r = 12.964 min, conversion = quant.

Isolated yield: 91%, average of two runs on a 100 µmol scale (85 % and 97%).

^{1}H-NMR (600 MHz, **C_6D_{12}**): δ = 7.43 (dd, $^3J_{HH}$ = 6.74 Hz, $^4J_{HH}$ = 1.12 Hz, 1H, H-4), 7.40 (dd, $^3J_{HH}$ = 6.74 Hz, $^4J_{HH}$ = 1.12 Hz, 1H, H-6), 6.86 (m, 5H, H-18, H-19, H-20), 6.59 (s, 1H, H-14), 6.40 (ps. t, $^3J_{HH}$ = 6.74 Hz, 1H, H-5), 2.18 (s, 3H, H-16), 1.92 (s, 6H, H-13), 0.92 (s, 9H, H-10), 0.00 ()s, 6H, H-8 ppm.

^{13}C-{^{1}H}-NMR (151 MHz, **C_6D_{12}**): δ = 146.2 (C3), 141.9 (C4), 140.9 (br. C11), 139.7 (C12), 137.7 (C15), 137.0 (C6), 128.6 (C18), 127.7 (C19), 127.6 (C14), 125.5 (C20), 112.2 (C5), 28.0 (C10), 23.4 (C13), 21.5 (C16), 19.9 (C9), -2.8 (C8) ppm.

^{11}B-{^{1}H}-NMR (192 MHz, **C_6D_{12}**): δ = 40.3 ppm.

HR-MS (APCI): m/z calc. For [M+H]+ 388.26311, found 388.26236.

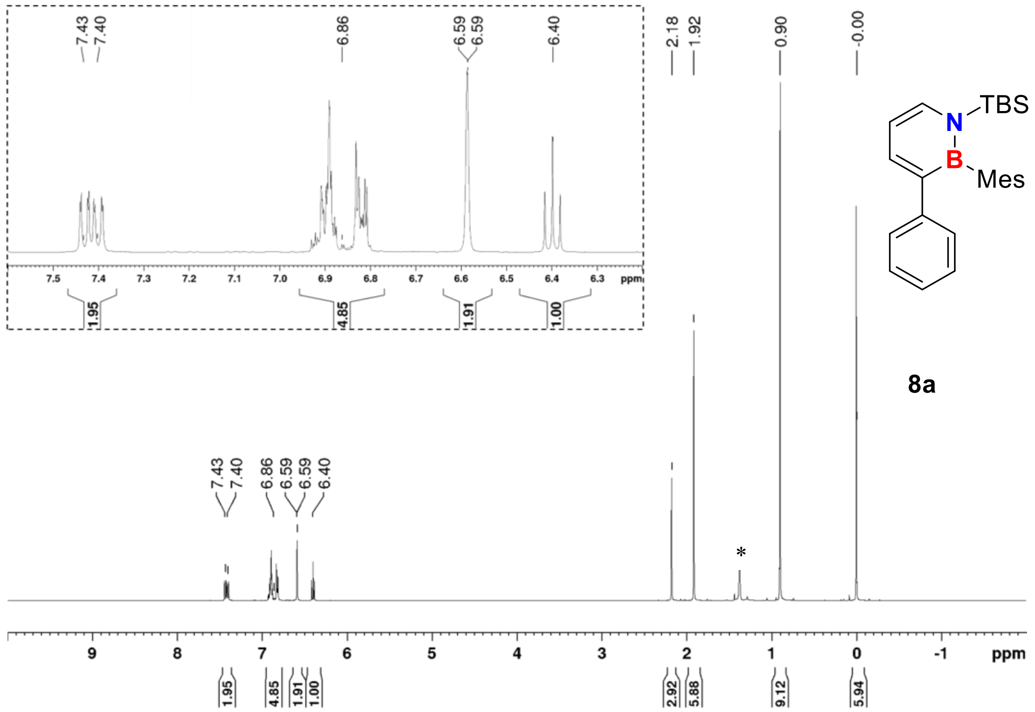

Figure S8. ^{1}H-NMR spectrum of compound **8a** in C_6D_{12} measured at a 600 MHz spectrometer. The enlarged section shows the region between 6.2 and 7.6 ppm for a better visibility of the aromatic signals. The solvent signal is marked with an asterisk.

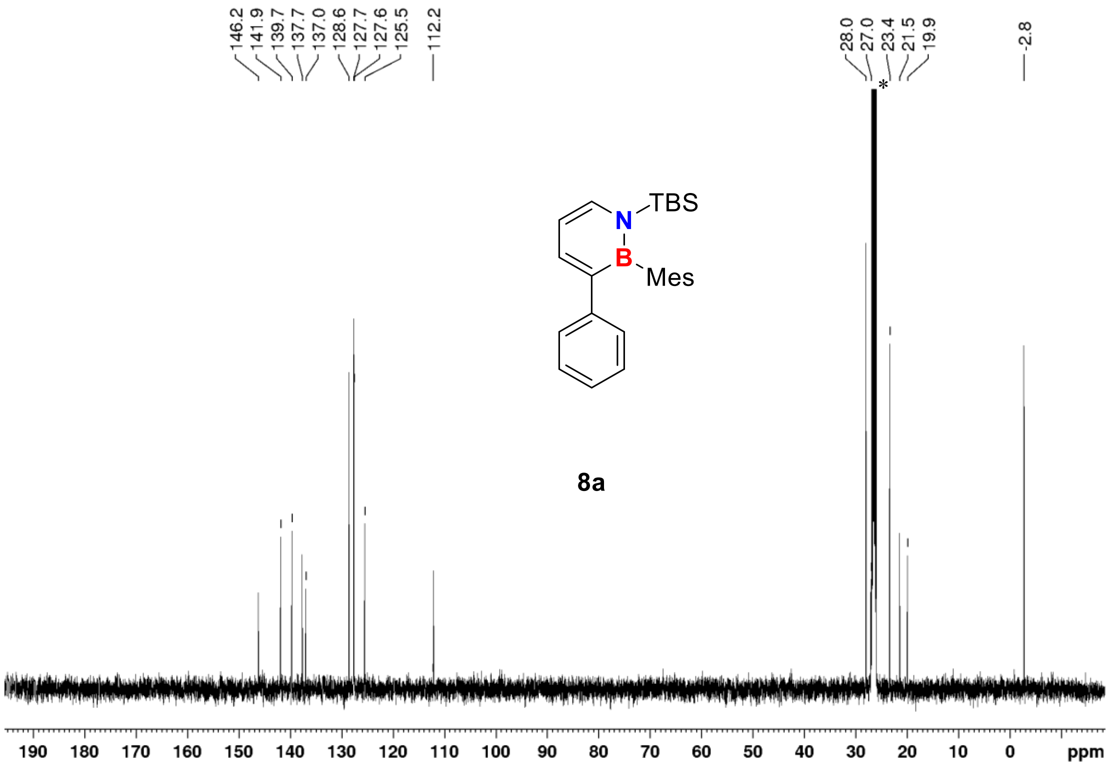

Figure S9. ^{13}C-{^{1}H}-NMR spectrum of compound **8a** in C_6D_{12} measured at a 600 MHz spectrometer. The solvent signal is marked with an asterisk.

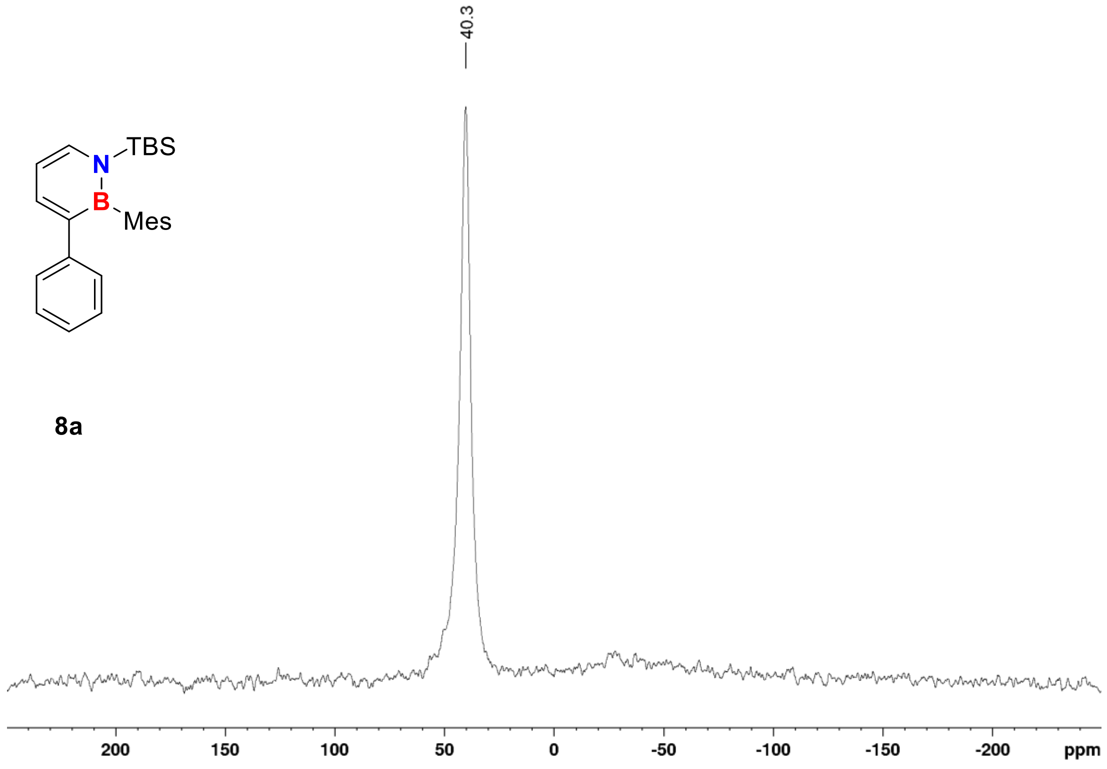

Figure S10. ^{11}B-{^{1}H}-NMR spectrum of compound **8a** in C_6D_{12} measured at a 600 MHz spectrometer.

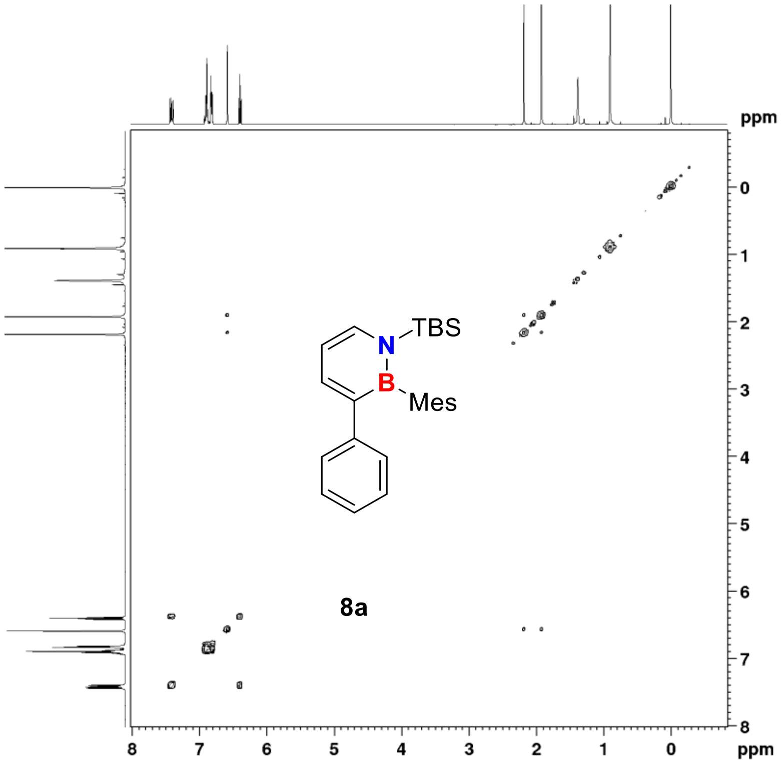

Figure S11. ^{1}H-^{1}H-COSY-NMR spectrum of compound **8a** in C_6D_{12} measured at a 600 MHz spectrometer.

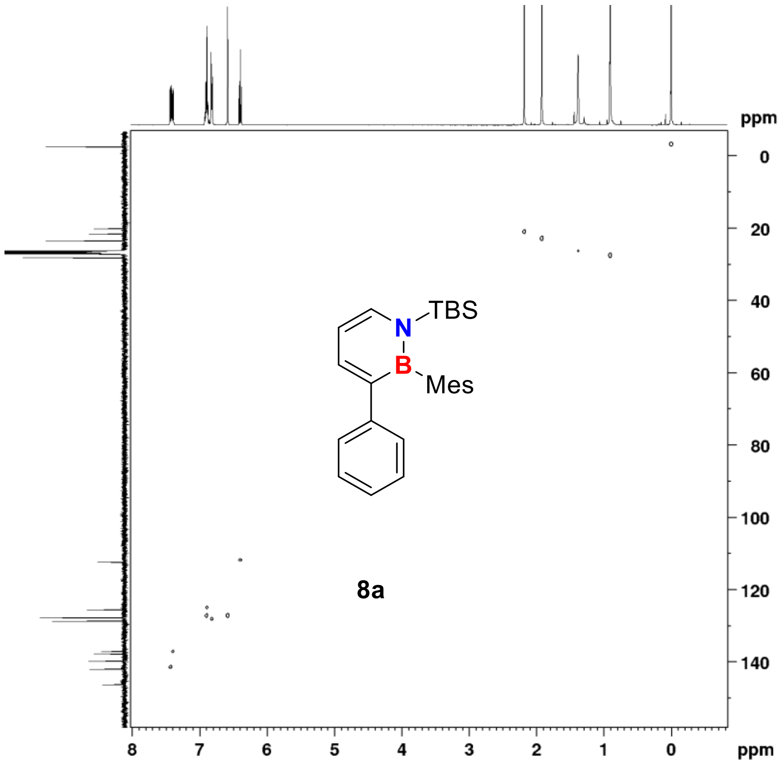

Figure S12. ^{1}H-^{13}C-HSQC-NMR spectrum of compound **8a** in C_6D_{12} measured at a 600 MHz spectrometer.

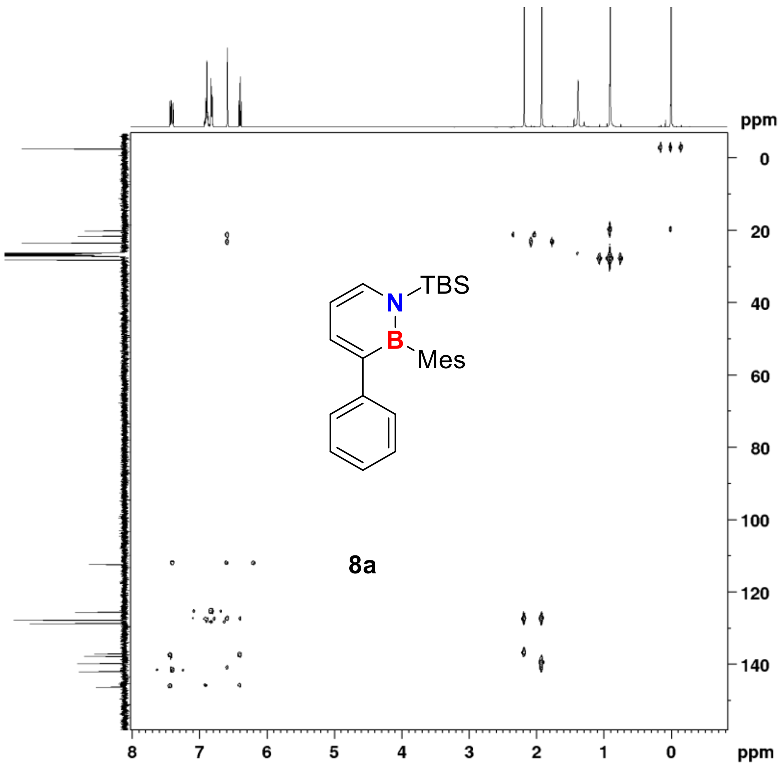

Figure S13. ^{1}H-^{13}C-HMBC-NMR spectrum of compound **8a** in C_6D_{12} measured at a 600 MHz spectrometer.

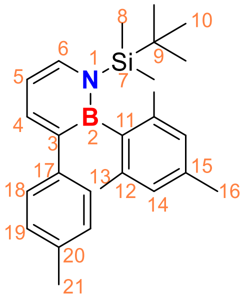

8b

$C_{26}H_{36}BNSi$ (401.48 g/mol)

GC-FID: t_r = 13.376 min, conversion = 98 %.

Isolated yield: 91%, average of two runs on a 100 µmol scale (91 % and 92%).

^{1}H-NMR (700 MHz, **C_6D_{12}**): δ = 7.40 (dd, $^{3}J_{HH}$ = 6.70 Hz, $^{4}J_{HH}$ = 1.00 Hz, 1H, H-4), 7.38 (dd, $^{3}J_{HH}$ = 6.91 Hz, $^{4}J_{HH}$ = 1.00 Hz, 1H, H-6), 6.73 (m, 4H, H-18/H-19), 6.59 (s, 2H, H-14), 6.38 (ps. t, 1H, H-5), 2.19 (s, 3H, H-16), 2.15 (s, 3H, H-21), 1.92 (H-13), 0.90 (H-10), -0.01 (H-8) ppm.

^{13}C-{^{1}H}-NMR (176 MHz, **C_6D_{12}**): δ = 145.8 (C3), 143.4 (C20), 141.7 (C11), 141.3 (C4), 139.7 (C17), 137.4 (C6), 136.9 (), 134.4 (), 128.5 (C18), 128.5 (C19) 127.6 (C14), 112.2 (C5), 28.0 (C10), 23.4 (C13), 21.5 (C16), 21.2 (C21), 19.9 (C9), -2.89 (C8) ppm.

^{11}B-{^{1}H}-NMR (128 MHz, **C_6D_6**): δ = 40.2 ppm.

HR-MS (APCI): m/z calc. For [M+H]+ 402.27878, found 402.27840.

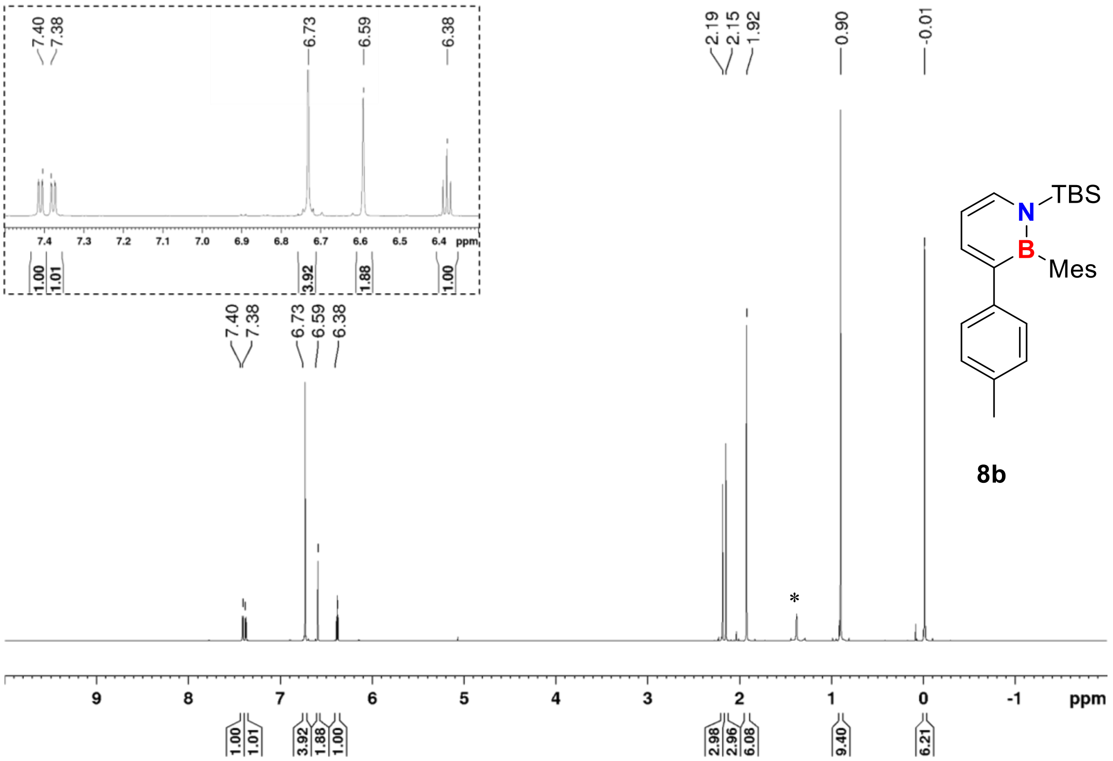

Figure S14. ^{1}H-NMR spectrum of compound **8b** in C_6D_{12} measured at a 700 MHz spectrometer. The enlarged section shows the region between 6.3 and 7.5 ppm for a better visibility of the aromatic signals. The solvent signal is marked with an asterisk.

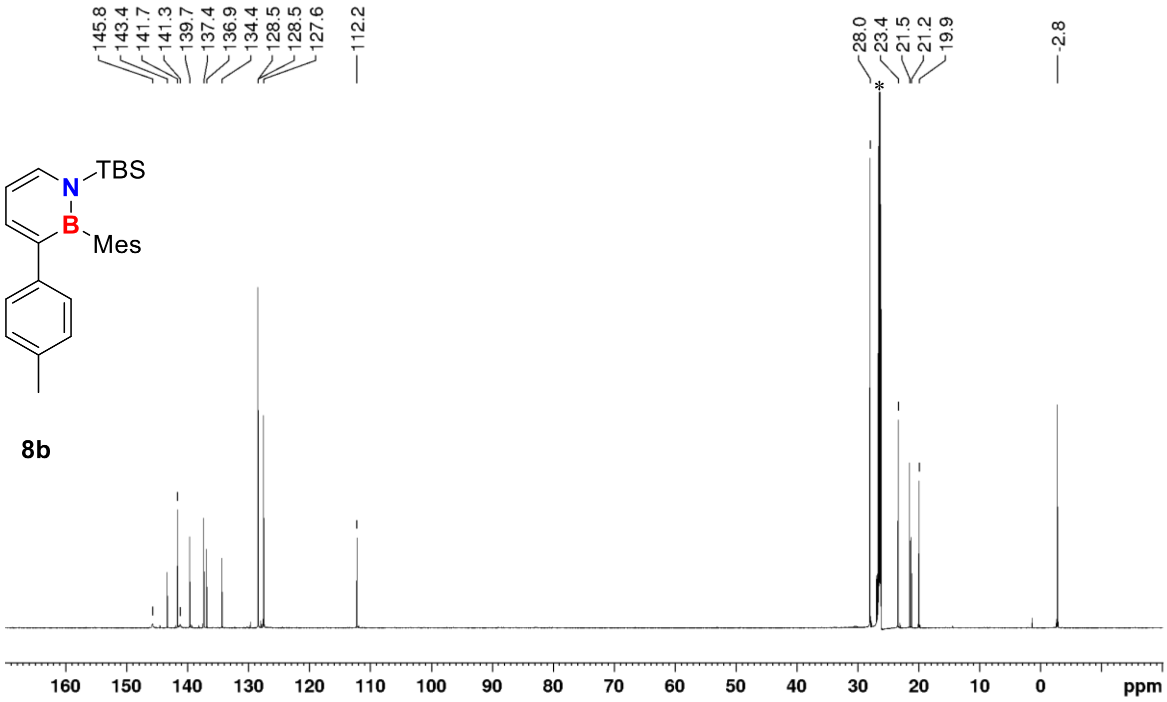

Figure S15. ^{13}C-{^{1}H}-NMR spectrum of compound **8b** in C_6D_{12} measured at a 700 MHz spectrometer. The solvent signal is marked with an asterisk.

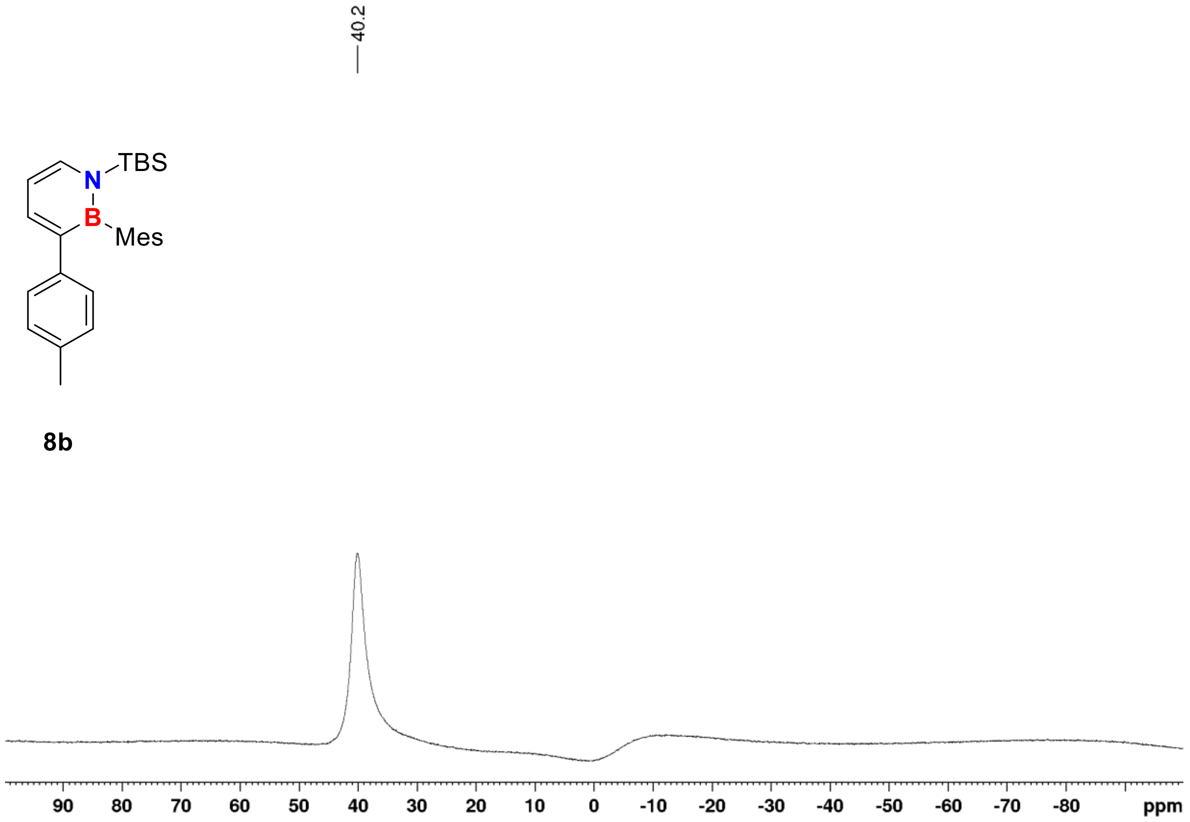

Figure S16. ^{11}B-{^{1}H}-NMR spectrum of compound **8b** in C_6D_{12} measured at a 400 MHz spectrometer.

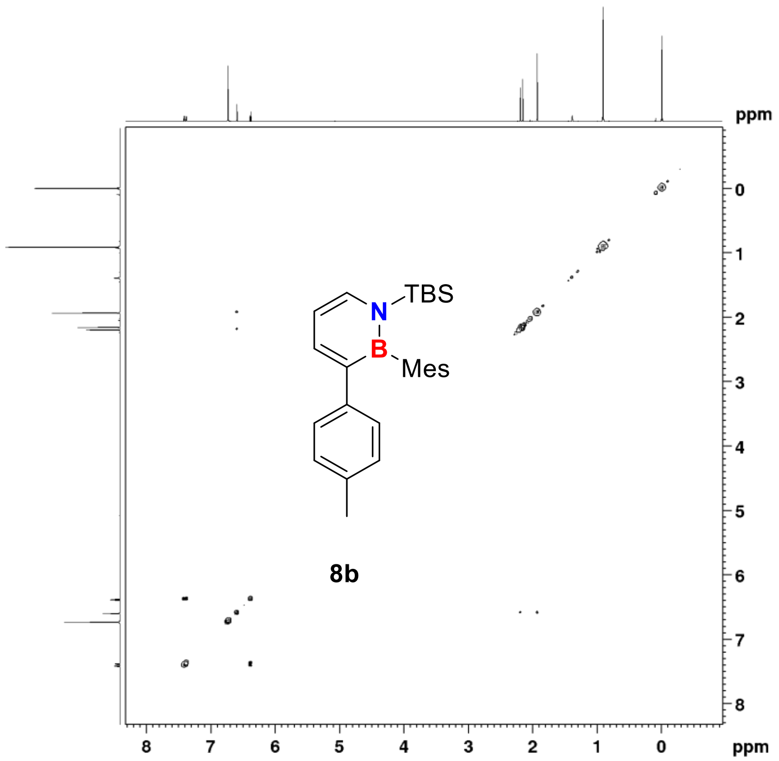

Figure S17. ^{1}H-^{1}H-COSY-NMR spectrum of compound **8b** in C_6D_{12} measured at a 700 MHz spectrometer.

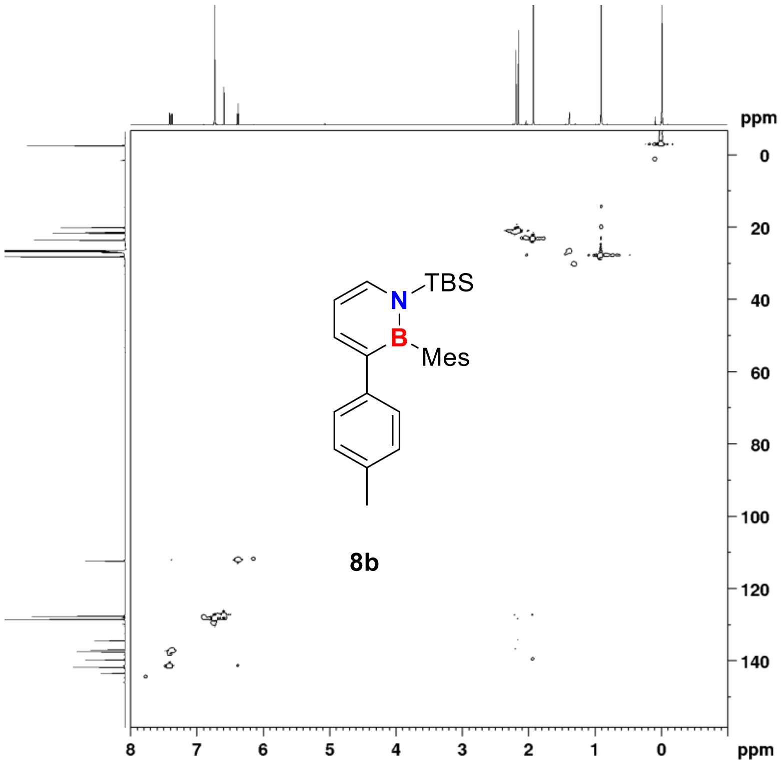

Figure S18. ^{1}H-^{13}C-HSQC-NMR spectrum of compound **8b** in C_6D_{12} measured at a 700 MHz spectrometer.

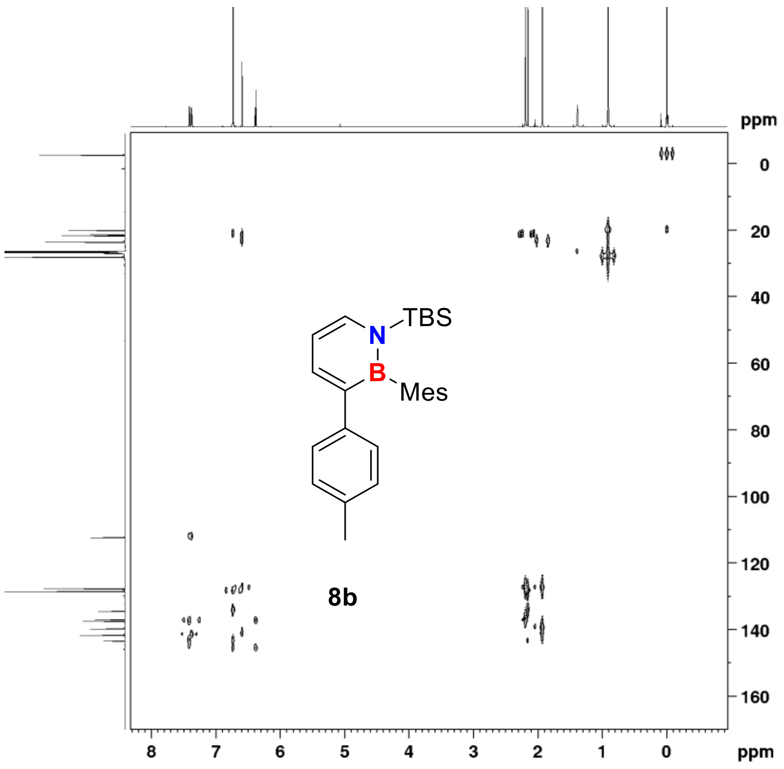

Figure S19. ^{1}H-^{13}C-HMBC-NMR spectrum of compound **8b** in C_6D_{12} measured at a 700 MHz spectrometer.

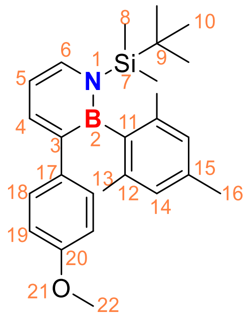

8c

$C_{26}H_{36}BNOSi$ (417.48 g/mol)

GC-FID: t_r = 43.564 min, conversion = 90 %.

Isolated yield: 96%, average of two runs on a 100 µmol scale (98 % and 95%).

^{1}H-NMR (400 MHz, **CD_2Cl_2**): δ = 7.46 (ps. t, 2H, H-4/ H-6), 6.83 (d, $^{3}J_{HH}$ = 8.81 Hz, 2H, H-19), 6.67 (s, 2H, H-14), 6.61 (ps. t, 1H, H-5), 6.49 (d, $^{3}J_{HH}$ = 8.81 Hz, 2H, H-18), 3.70 (s, 3H, H-22), 2.24 (s, 3H, H-16), 1.96 (s, 6H, H-13), 0.91 (s, 9H, H-10), -0.01 (s, 6H, H-8) ppm.

^{13}C-{^{1}H}-NMR (151 MHz, **C_6D_{12}**): δ = 158.5 (C20), 145.4 (C3), 141.2 (C4), 139.6 (C11), 138.5 (C12), 137.0 (C6), 136.8 (C15), 129.3 (C19), 128.3 (C17), 127.5 (C14), 113.1 (C18), 112.2 (C5), 54.5 (C22), 27.9 (C10), 23.3 (C13), 21.4 (C16), 19.9 (C9), 1.3 (*grease*), -2.8 (C8) ppm.

^{11}B-{^{1}H}-NMR (128 MHz, **CD_2Cl_2**): δ = 39.8 ppm.

HR-MS (APCI): m/z calc. For [M+H]+ 418.27299, found 418.27370.

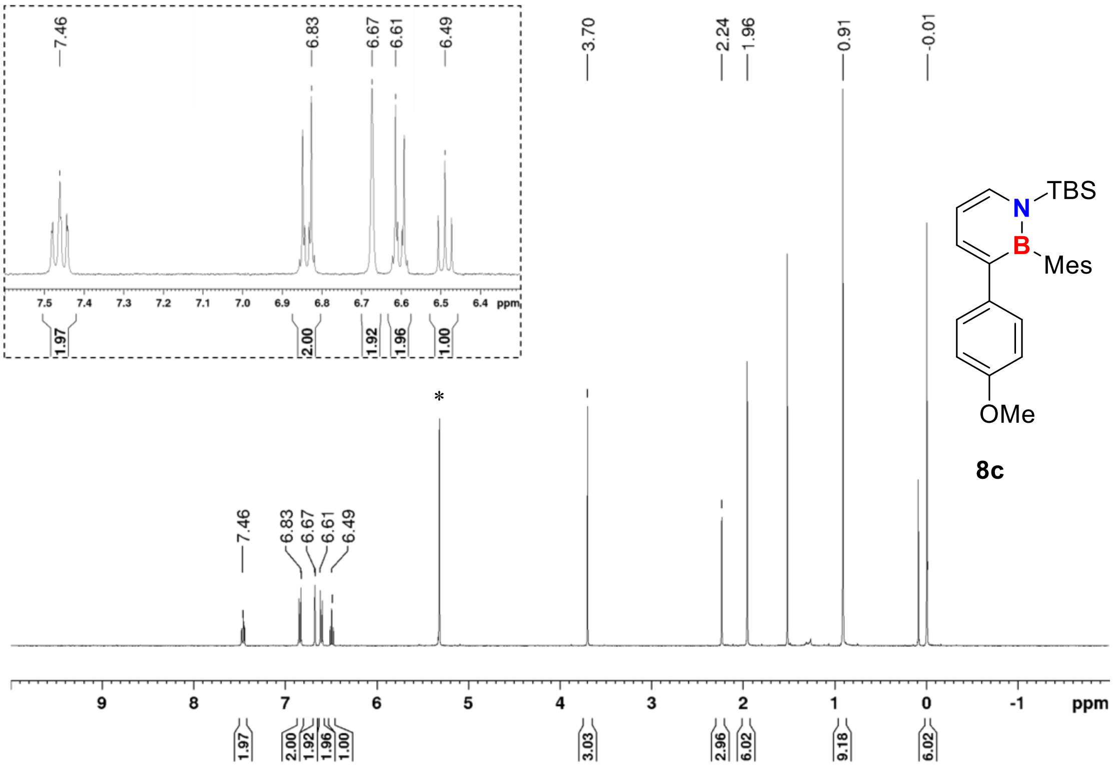

Figure S20. ^{1}H-NMR spectrum of compound **8c** in CD_2Cl_2 measured at a 400 MHz spectrometer. The enlarged section shows the region between 6.3 and 7.6 ppm for a better visibility of the aromatic signals. The solvent signal is marked with an asterisk.

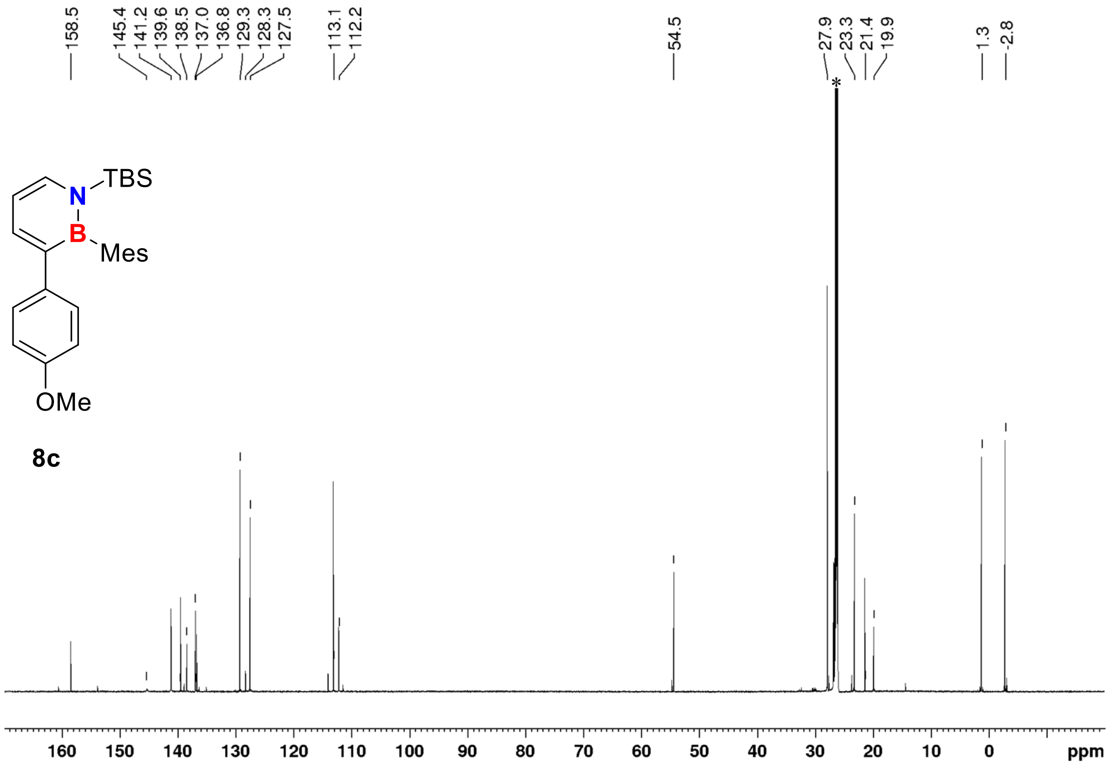

Figure S21. ^{13}C-{^{1}H}-NMR spectrum of compound **8c** in C_6D_{12} measured at a 600 MHz spectrometer. The solvent signal is marked with an asterisk.

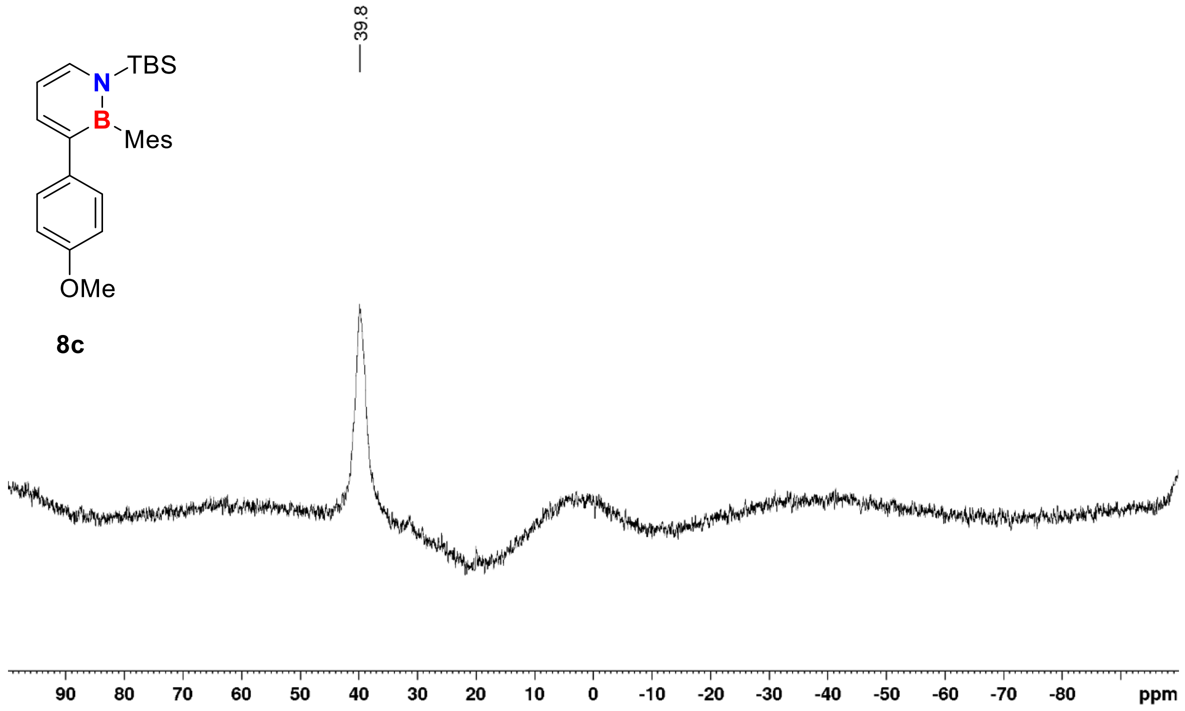

Figure S22. ^{11}B-{^{1}H}-NMR spectrum of compound **8c** in CD_2Cl_2 measured at a 400 MHz spectrometer.

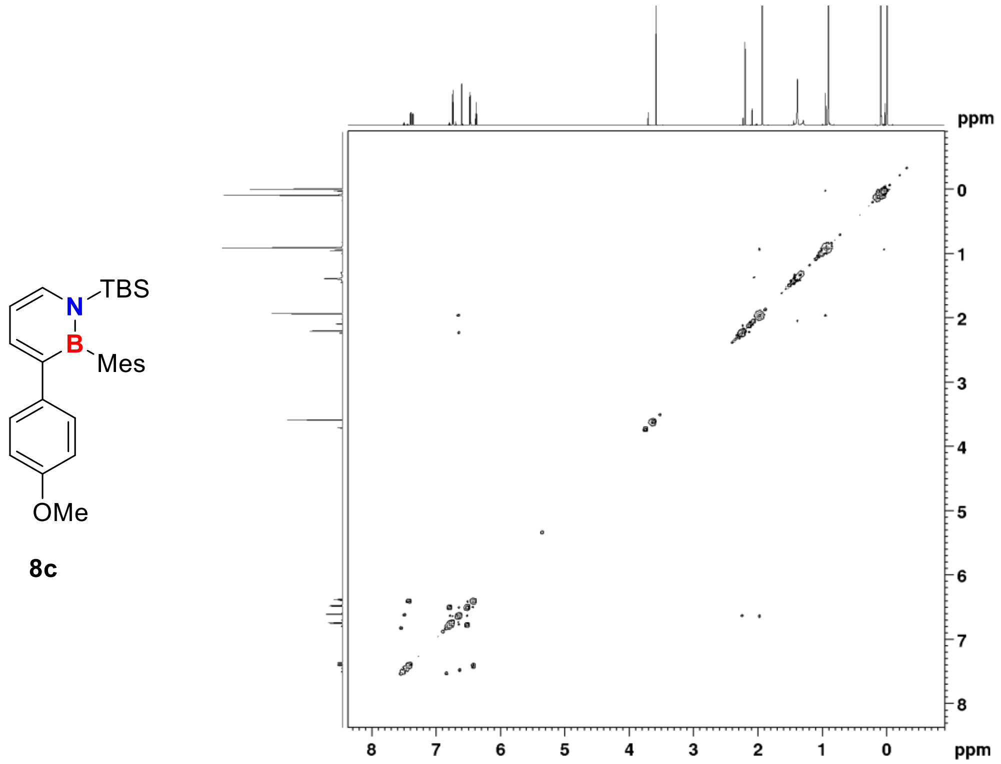

Figure S23. ^{1}H-^{1}H-COSY-NMR spectrum of compound **8c** in CD_2Cl_2 measured at a 600 MHz spectrometer.

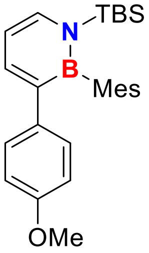

8c

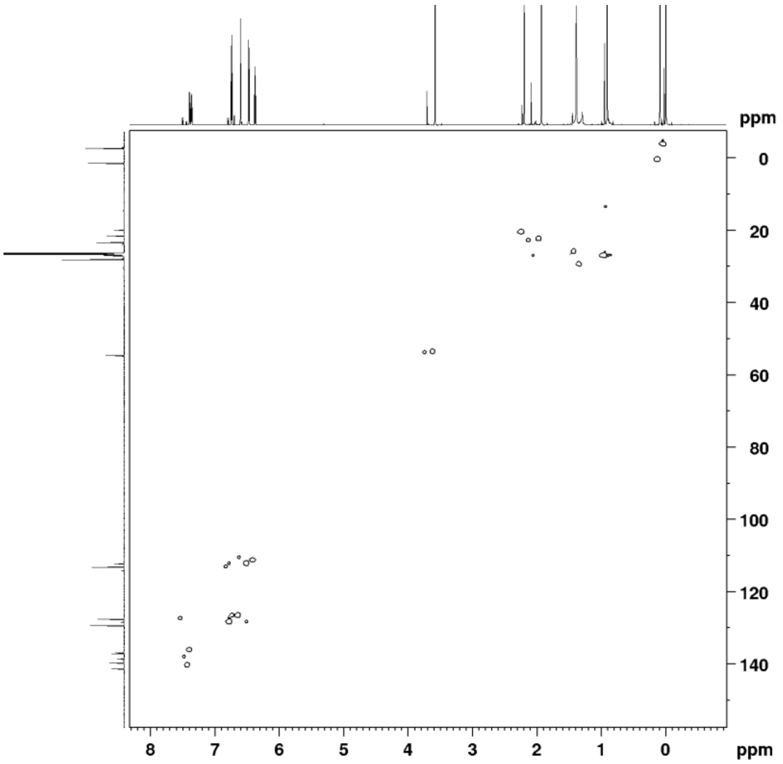

Figure S24. ^{1}H-^{13}C-HSQC-NMR spectrum of compound **8c** in CD_2Cl_2 measured at a 600 MHz spectrometer.

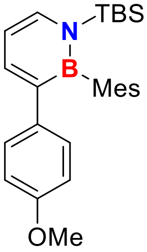

8c

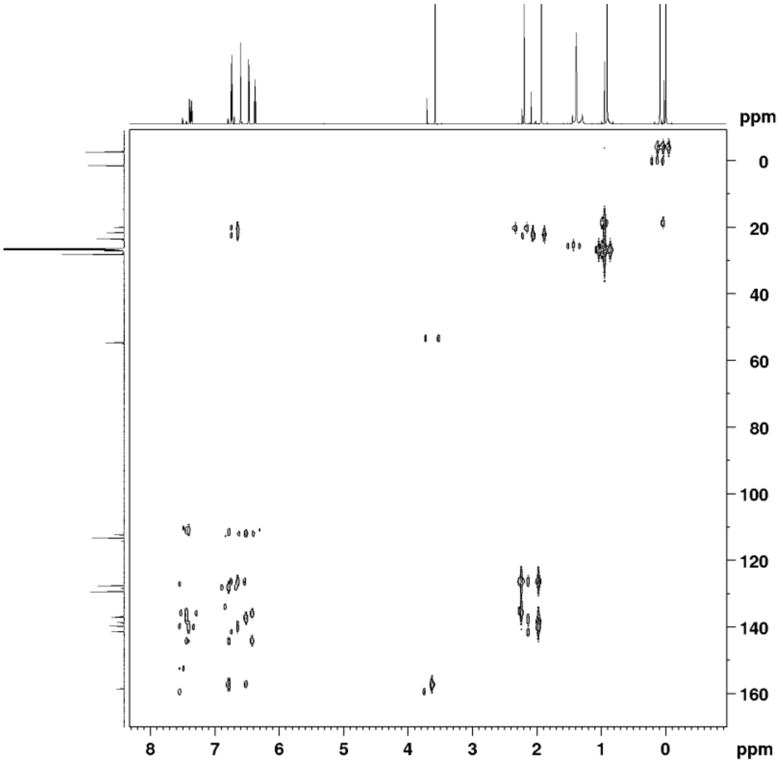

Figure S25. ^{1}H-^{13}C-HMBC-NMR spectrum of compound **8c** in CD_2Cl_2 measured at a 600 MHz spectrometer.

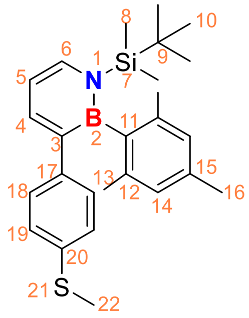

8d

$C_{26}H_{36}BNSSi$ (433.54 g/mol)

Isolated yield: 84%, average of two runs on a 100 µmol scale (76 %, 80% and 97%).

^{1}H-NMR (300 MHz, **CD_2Cl_2**): δ = 7.50 (ps. t, 2H, H-4/H-6), 6.94 (dm, $^{3}J_{HH}$ = 8.81 Hz, 2H, H-19), 6.87 (dm, $^{3}J_{HH}$ = 8.81 Hz, 2H, H-18), 6.69 (s, 2H, H-14), 6.51 (ps. t, 1H, H-3), 2.40 (s, 3H, H-22), 2.24 (s, 3 H, H-16), 1.97 (s, 3H, H-13), 0.92 (s, 9H, H-10), 0.00 (s, 6H, H-8) ppm.

^{13}C-{^{1}H}-NMR (151 MHz, **CD_2Cl_2**): δ = 143.4 (C17), 141.8 (C6), 141.0 (br. C11), 139.5 (C12), 138.5 (C4), 138.1 (br. C3), 137.0 (C15), 134.9 (C20), 128.9 (C19), 127.2 (C14), 125.9 (C18), 111.8 (C5), 30.1 (*n-hexane*), 27.7 (C10), 23.3 (C13), 21.3 (C16), 19.6 (C9), 15.9 (C22), 1.2 (*grease*), -2.9 (C8) ppm.

^{11}B-{^{1}H}-NMR (128 MHz, **CD_2Cl_2**): δ = 40.3 ppm.

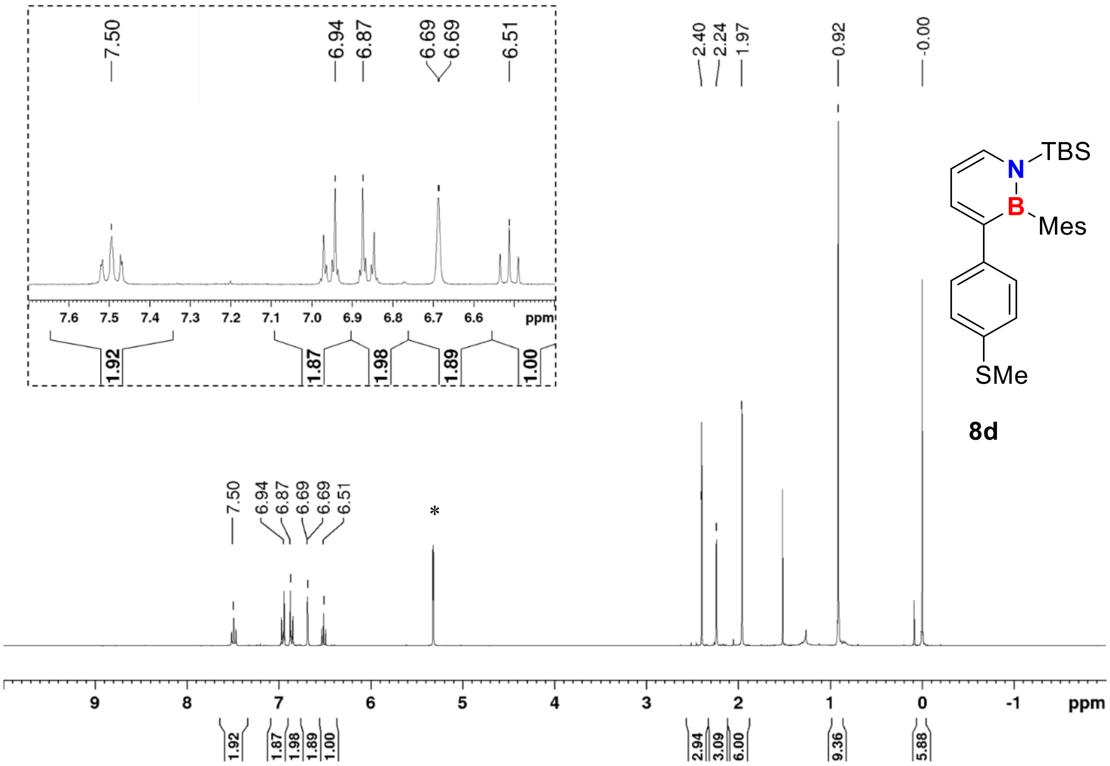

Figure S26. ^{1}H-NMR spectrum of compound **8d** in CD_2Cl_2 measured at a 400 MHz spectrometer. The enlarged section shows the region between 6.4 and 7.7 ppm for a better visibility of the aromatic signals. The solvent signal is marked with an asterisk.

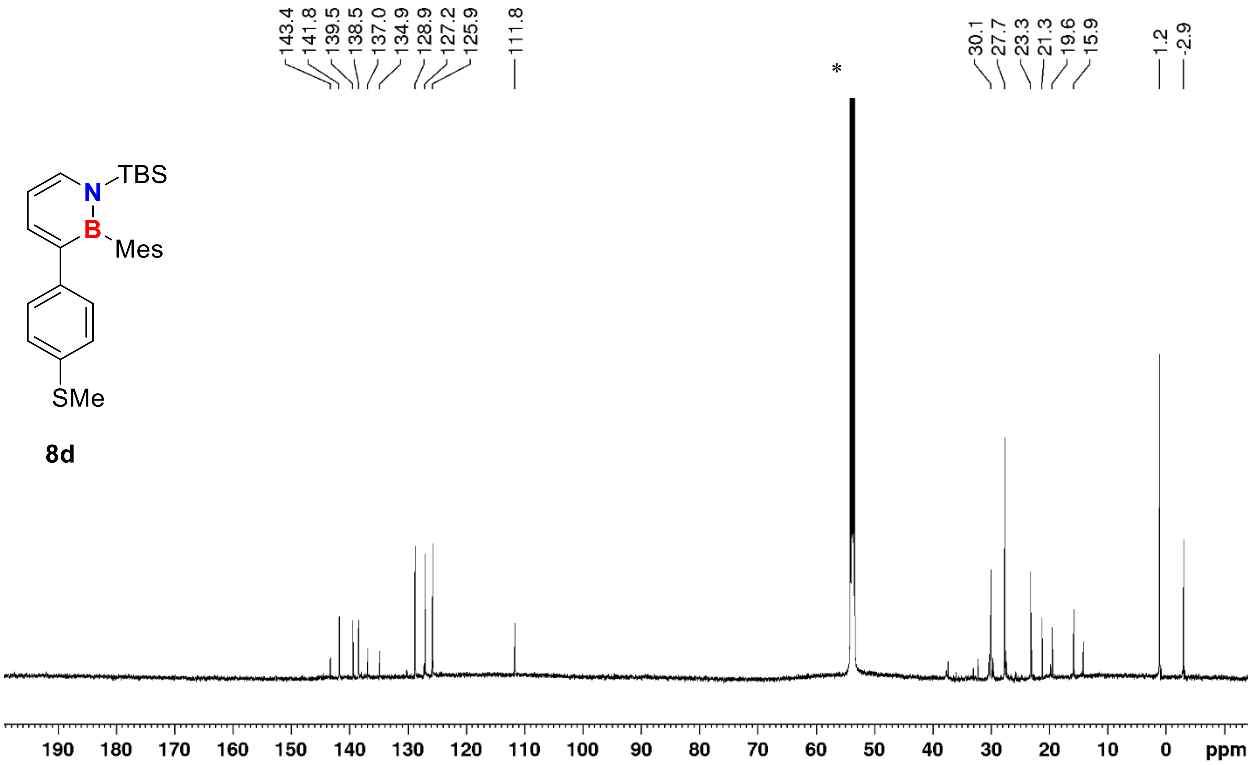

Figure S27. ^{13}C-{^{1}H}-NMR spectrum of compound **8d** in CD_2Cl_2 measured at a 600 MHz spectrometer. The solvent signal is marked with an asterisk.

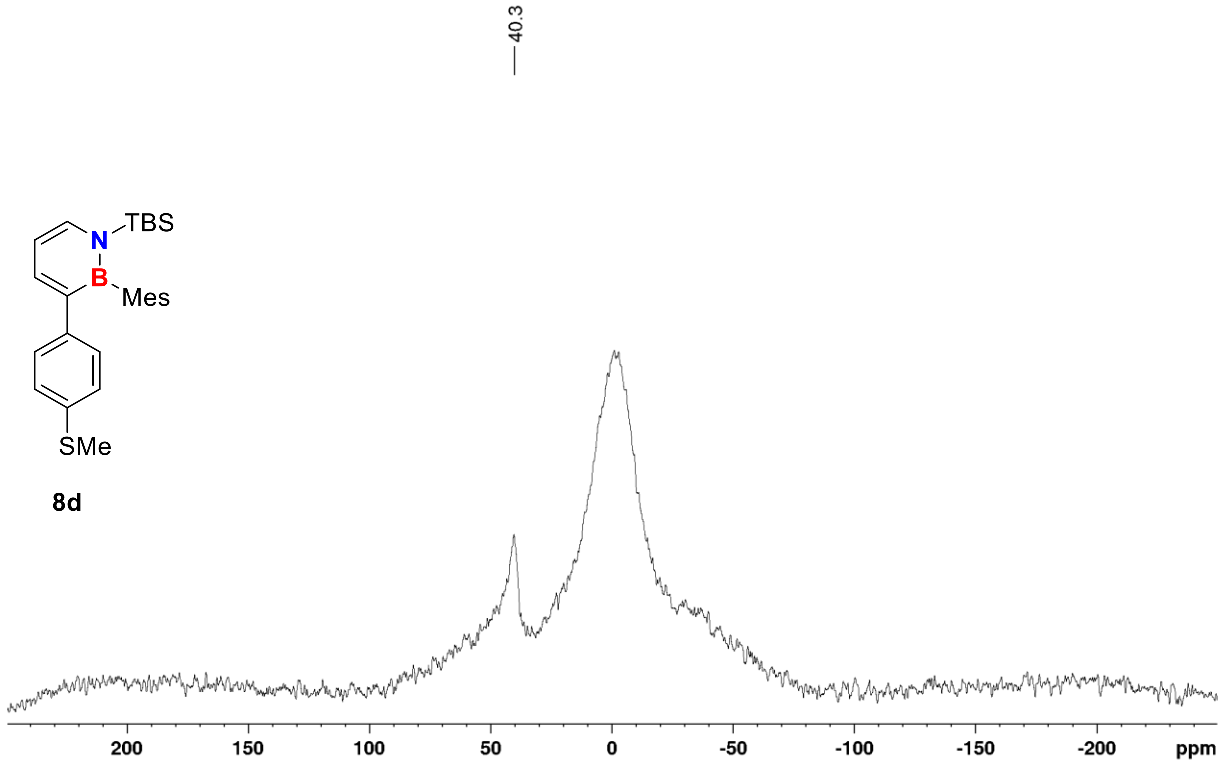

Figure S28. ^{11}B-{^{1}H}-NMR spectrum of compound **8d** in CD_2Cl_2 measured at a 300 MHz spectrometer. The broad signal between -30 and 30 ppm corresponds to the borosilicate glass of the NMR tube.

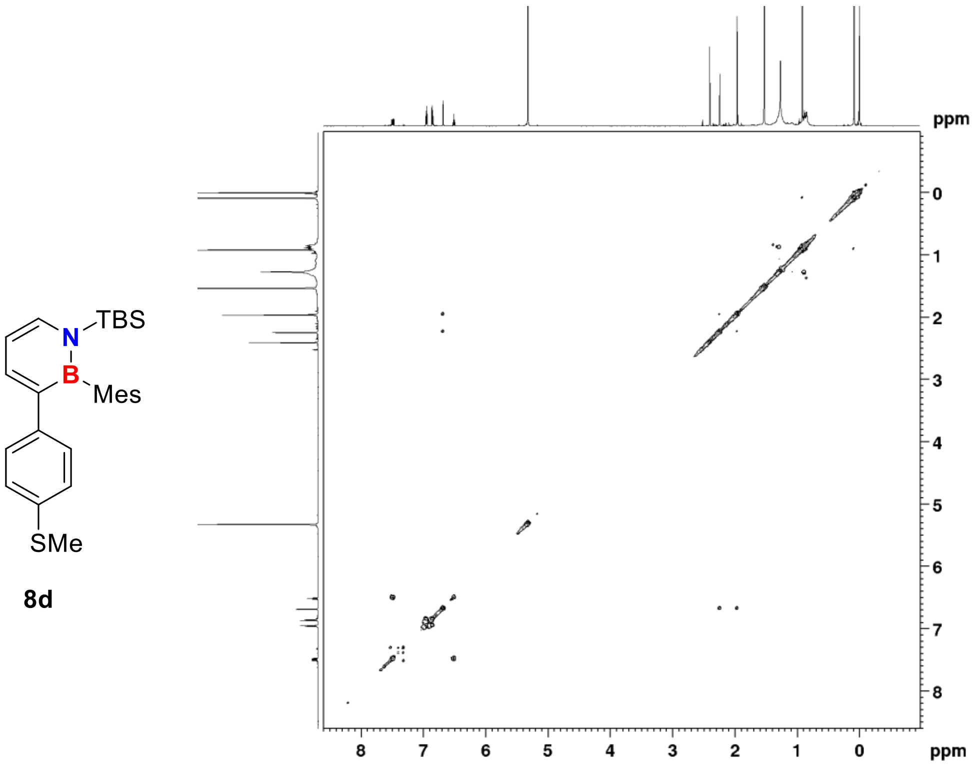

Figure S29. ^{1}H-^{1}H-COSY-NMR spectrum of compound **8d** in CD_2Cl_2 measured at a 600 MHz spectrometer.

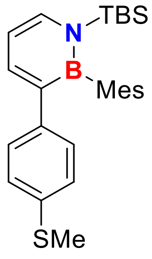

8d

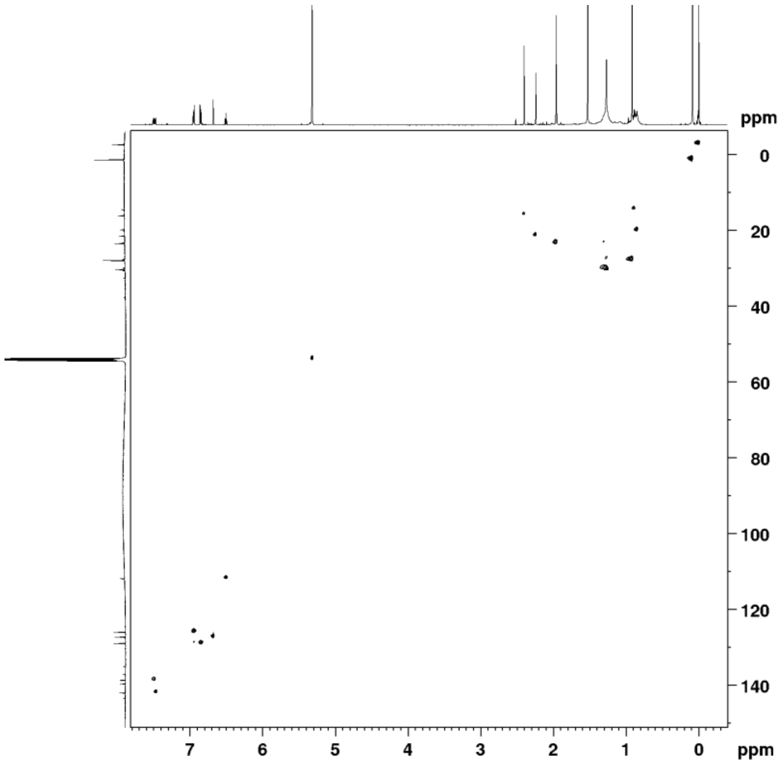

Figure S30. ^{1}H-^{13}C-HSQC-NMR spectrum of compound **8d** in CD_2Cl_2 measured at a 600 MHz spectrometer.

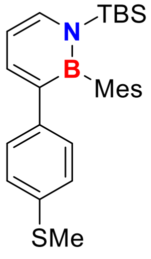

8d

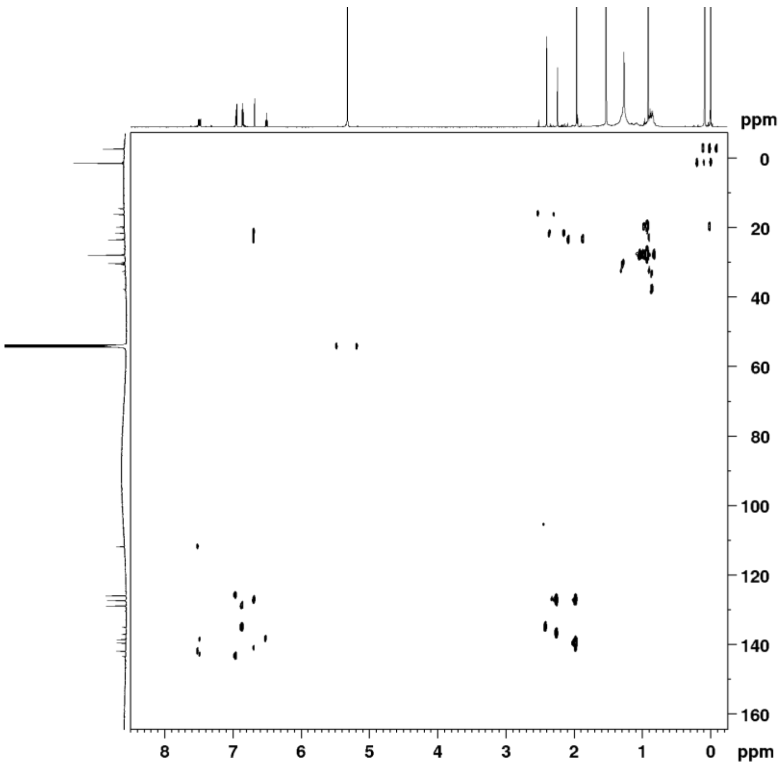

Figure S31. ^{1}H-^{13}C-HMBC-NMR spectrum of compound **8d** in CD_2Cl_2 measured at a 600 MHz spectrometer.

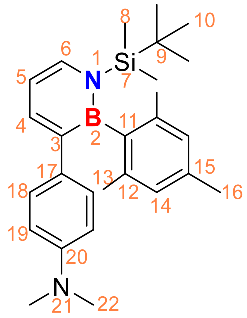

8e

$C_{28h39}BN_2Si$ (430.52 g/mol)

GC-FID: t_r = 11.308 min, conversion = 97 %.

Isolated yield: 88%, average of two runs on a 100 µmol scale (83 % and 93%).

^{1}H-NMR (600 MHz, **C_6D_{12}**): δ = 7.37 (dd, $^3J_{HH}$ = 6.69 Hz, $^4J_{HH}$ = 1.11 Hz, 2H, H-4), 7.33 (dd, $^3J_{HH}$ = 6.76 Hz, $^4J_{HH}$ = 1.11 Hz, 2H, H-6), 6.71 (dm, $^3J_{HH}$ = 9.01 Hz, 2H, H-19), 6.60 (s, 2H, H-14), 6.36 (ps.t, 1H, H-5), 6.34 (dm $^3J_{HH}$ = 9.01 Hz, ,2H, H-18), 2.75 (s, 6H, H-22), 2.19 (s, 3H, H-16), 1.94 (s, 6H, H-13), 0.90 (s, 9H, H-10), -0.02 (s, 6H, H-8) ppm.

^{13}C-{^{1}H}-NMR (151 MHz, **C_6D_{12}**): δ = 149.1 (C20), 145.8 (C3), 141.7 (C11), 140.7 (C4), 139.8 (C12), 136.7 (C15), 136.5 (C6), 135.1 (C17), 129.1 (C19), 127.5 (C14), 112.6 (C18), 112.4 (C5), 40.8 (C22), 28.1 (C10), 23.3 (C13), 21.5 (C16), 19.9 (C9), -2.7 (C8) ppm.

^{11}B-{^{1}H}-NMR (192 MHz, **C_6D_{12}**): δ = 40.1 ppm.

HR-MS (APCI): m/z calc. For [M+H]+ 431.30535, found 431.30490.

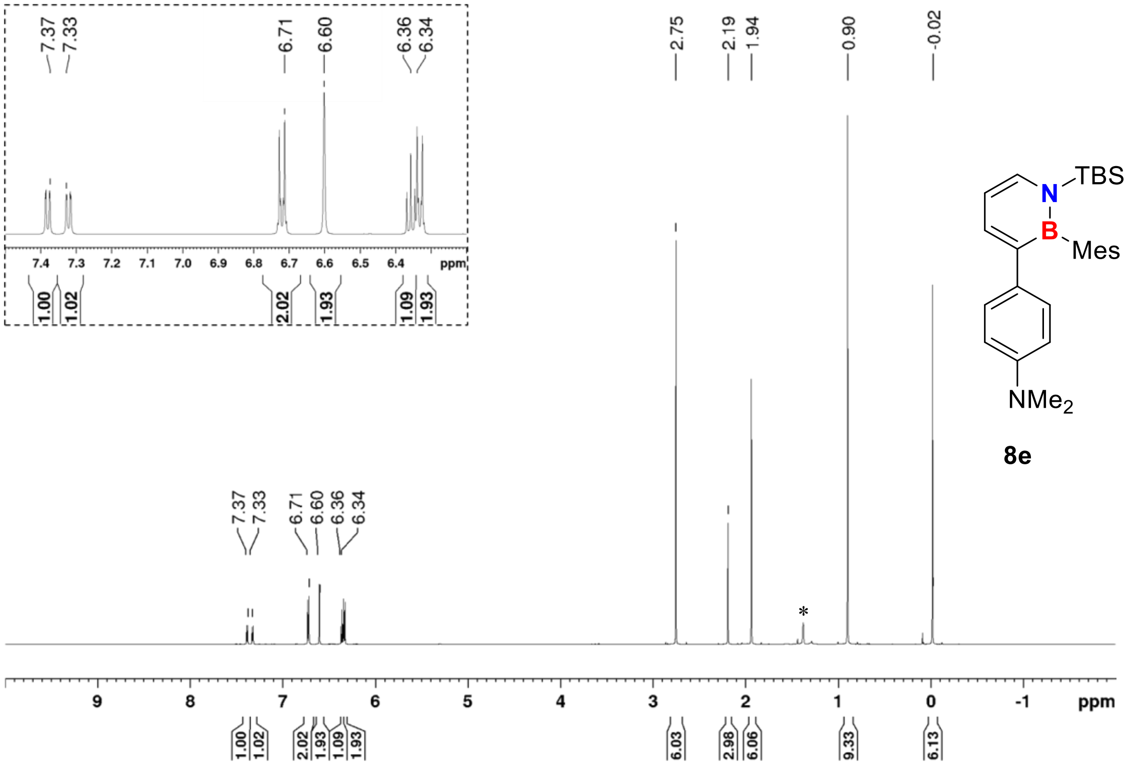

Figure S32. ^{1}H-NMR spectrum of compound **8e** in C_6D_{12} measured at a 600 MHz spectrometer. The enlarged section shows the region between 6.2 and 7.5 ppm for a better visibility of the aromatic signals. The solvent signal is marked with an asterisk.

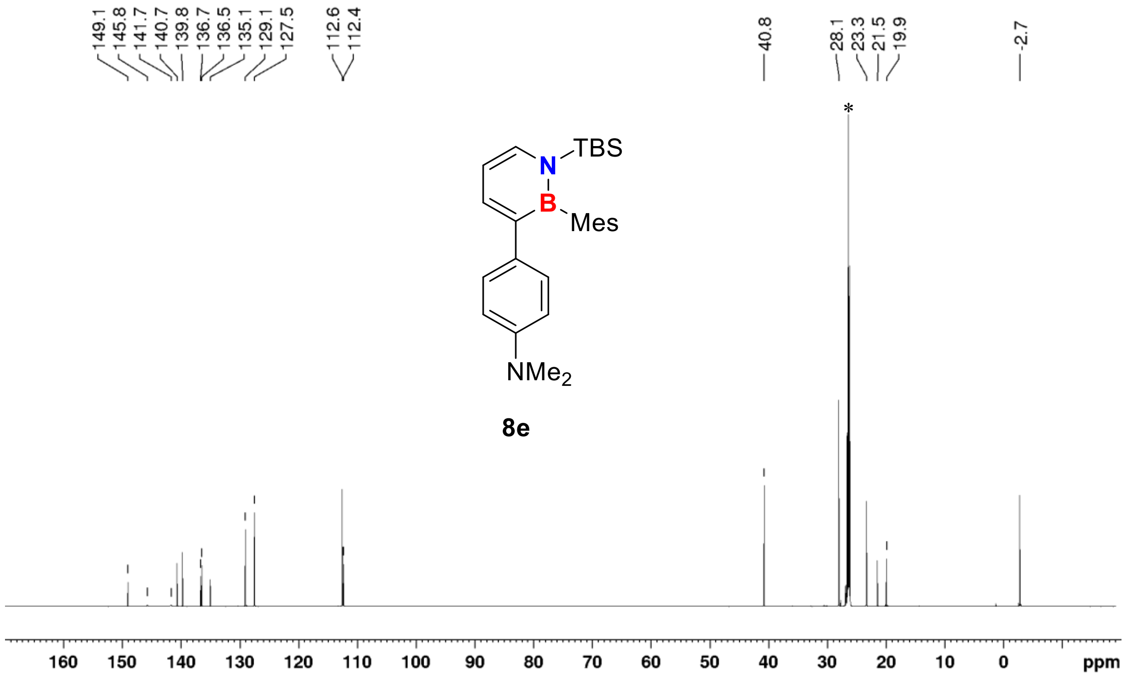

Figure S33. ^{13}C-{^{1}H}-NMR spectrum of compound **8e** in C_6D_{12} measured at a 600 MHz spectrometer. The solvent signal is marked with an asterisk.

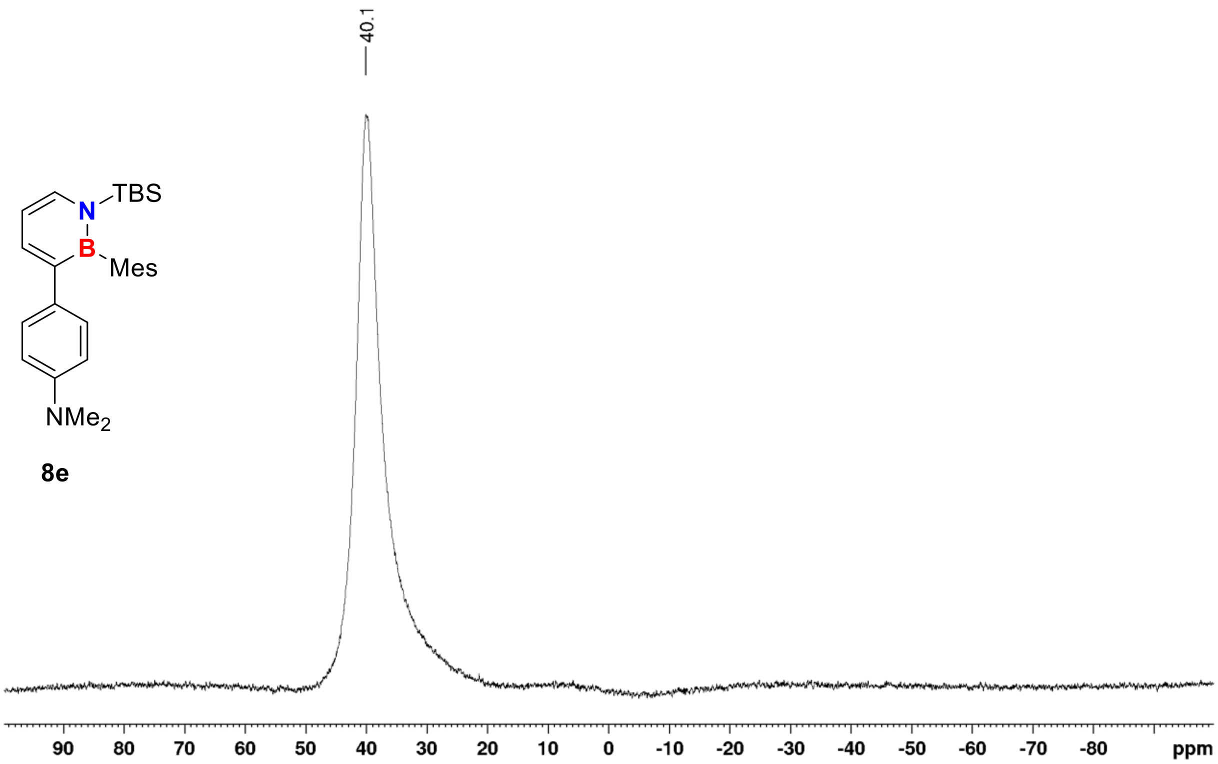

Figure S34. ^{11}B-{^{1}H}-NMR spectrum of compound **8e** in C_6D_{12} measured at a 600 MHz spectrometer.

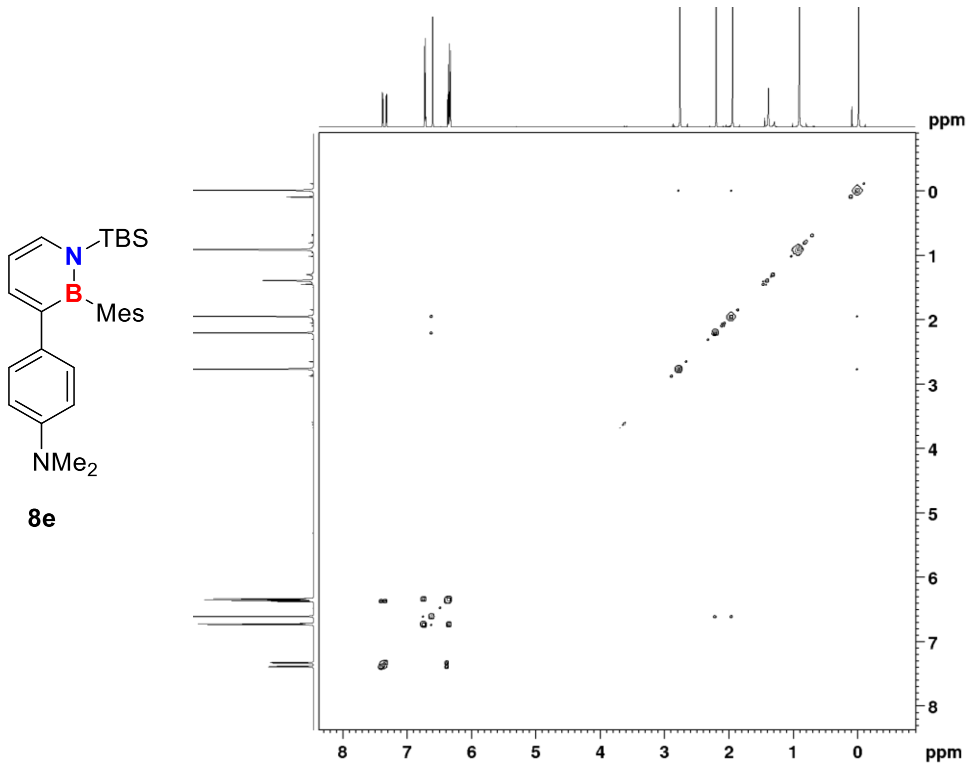

Figure S35. ^{1}H-^{1}H-COSY-NMR spectrum of compound **8e** in C_6D_{12} measured at a 600 MHz spectrometer.

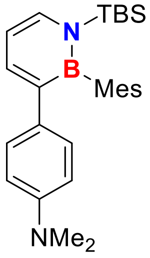

8e

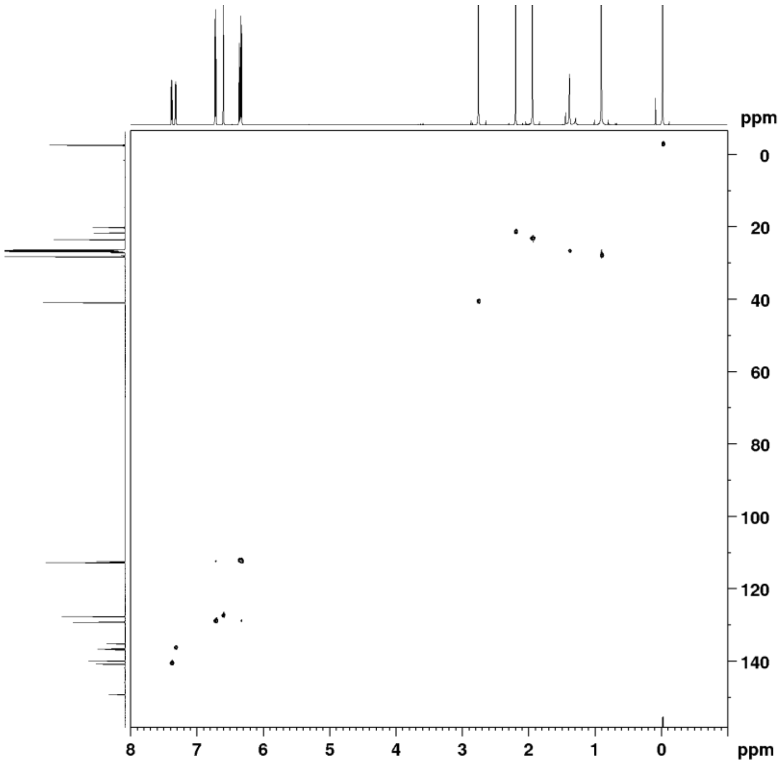

Figure S36. ^{1}H-^{13}C-HSQC-NMR spectrum of compound **8e** in C_6D_{12} measured at a 600 MHz spectrometer.

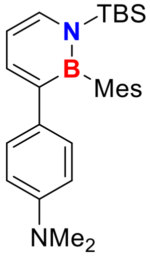

8e

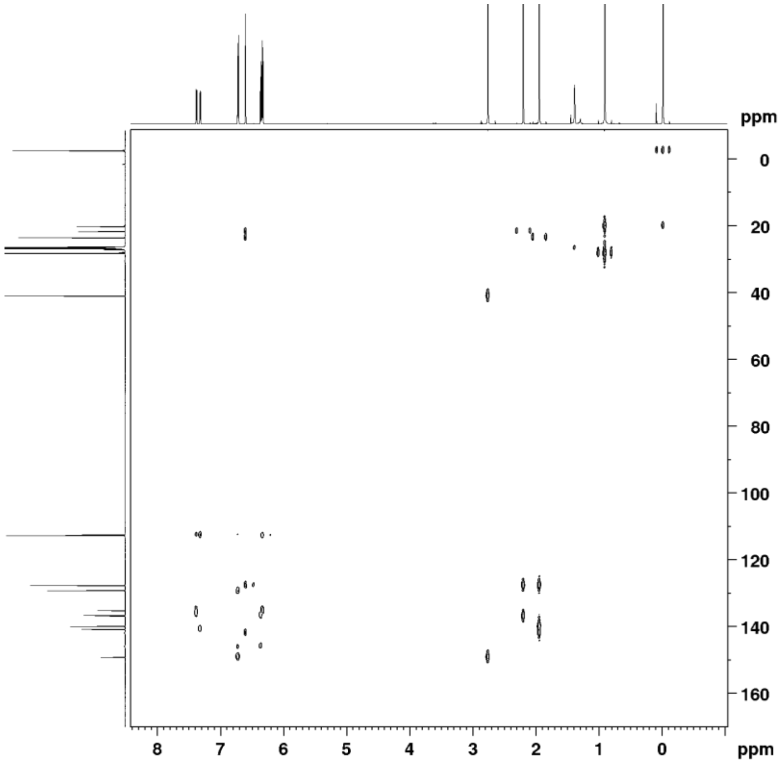

Figure S37. ^{1}H-^{13}C-HMBC-NMR spectrum of compound **8e** in C_6D_{12} measured at a 600 MHz spectrometer.

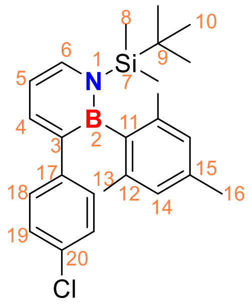

8f

$C_{25}H_{33}BClNSi$ (421.89 g/mol)

GC-FID: t_r = 14.309 min, conversion = quant.

Isolated yield: 92%, average of two runs on a 100 µmol scale (91 % and 93%).

^{1}H-NMR (600 MHz, **$CDCl_3$**): δ = 7.53 (dd, $^3J_{HH}$ = 6.74 Hz, $^4J_{HH}$ = 1.06 Hz, 1H, H-4), 7.42 (dd, $^3J_{HH}$ = 6.83 Hz, $^4J_{HH}$ = 1.06 Hz, 1H, H-6), 7.06 (dm, $^3J_{HH}$ = 8.51 Hz, 2H, H-19), 6.98 (dm, $^3J_{HH}$ = 8.51 Hz, 2H, H-18), 6.76 (s, 2H, H-14), 6.42 (ps. t, 1H, H-5), 2.24 (s, 3H, H-16), 2.10 (s, 6H, H-13), 0.86 (s, 9H, H-10), 0.00 (s, 6H, H-8) ppm.

^{13}C-{^{1}H}-NMR (151 MHz, **C_6D_{12}**): δ = 144.6 (C20), 144.4 (C11), 142.0 (C4), 140.8 (C3), 139.6 (C12), 138.2 (C6), 137.4 (C15), 132.3 (C17), 129.7 (C18), 128.0 (C19), 127.8 (C14), 112.2 (C5), 28.0 (C10), 23.3 (C13), 21.5 (C16), 19.9 (C9), -2.8 (C8) ppm.

^{11}B-{^{1}H}-NMR (192 MHz, **$CDCl_3$**): δ = 40.0 ppm.

HR-MS (APCI): m/z calc. For [M+H]+ 422.22413, found 422.22344.

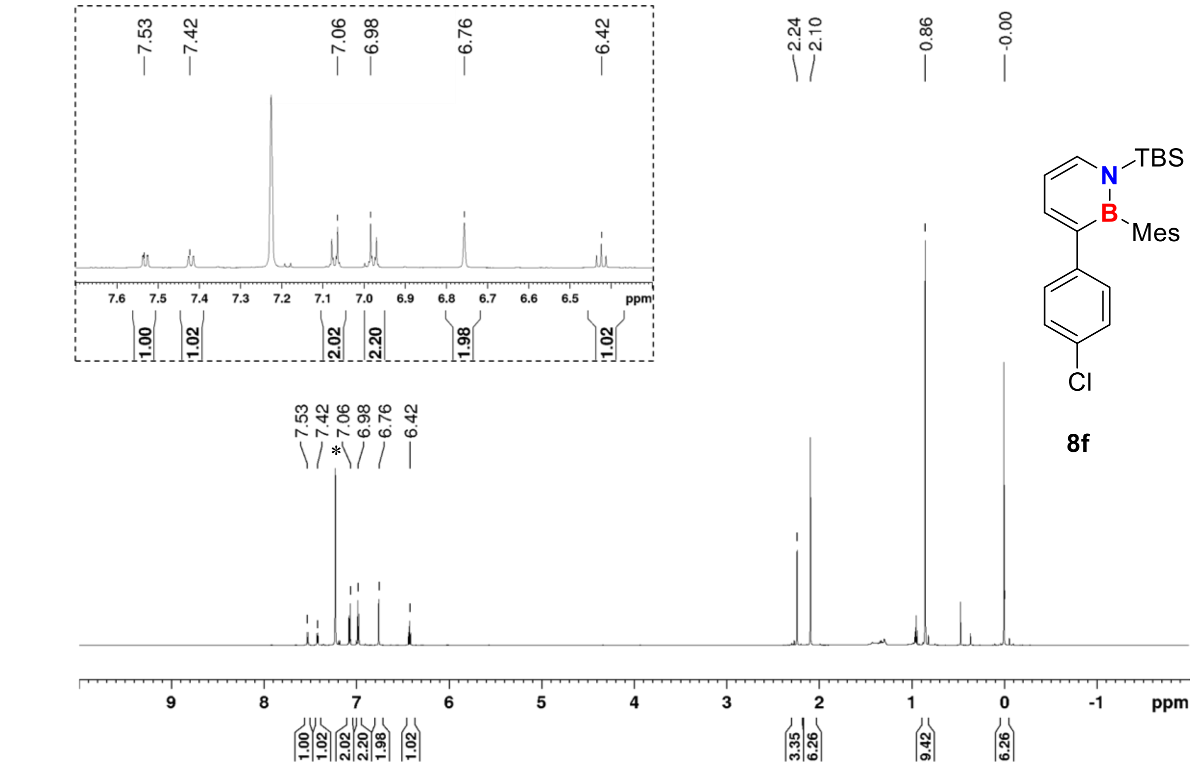

Figure S38. ^{1}H-NMR spectrum of compound **8f** in $CDCl_3$ measured at a 600 MHz spectrometer. The enlarged section shows the region between 6.3 and 7.7 ppm for a better visibility of the aromatic signals. The solvent signal is marked with an asterisk.

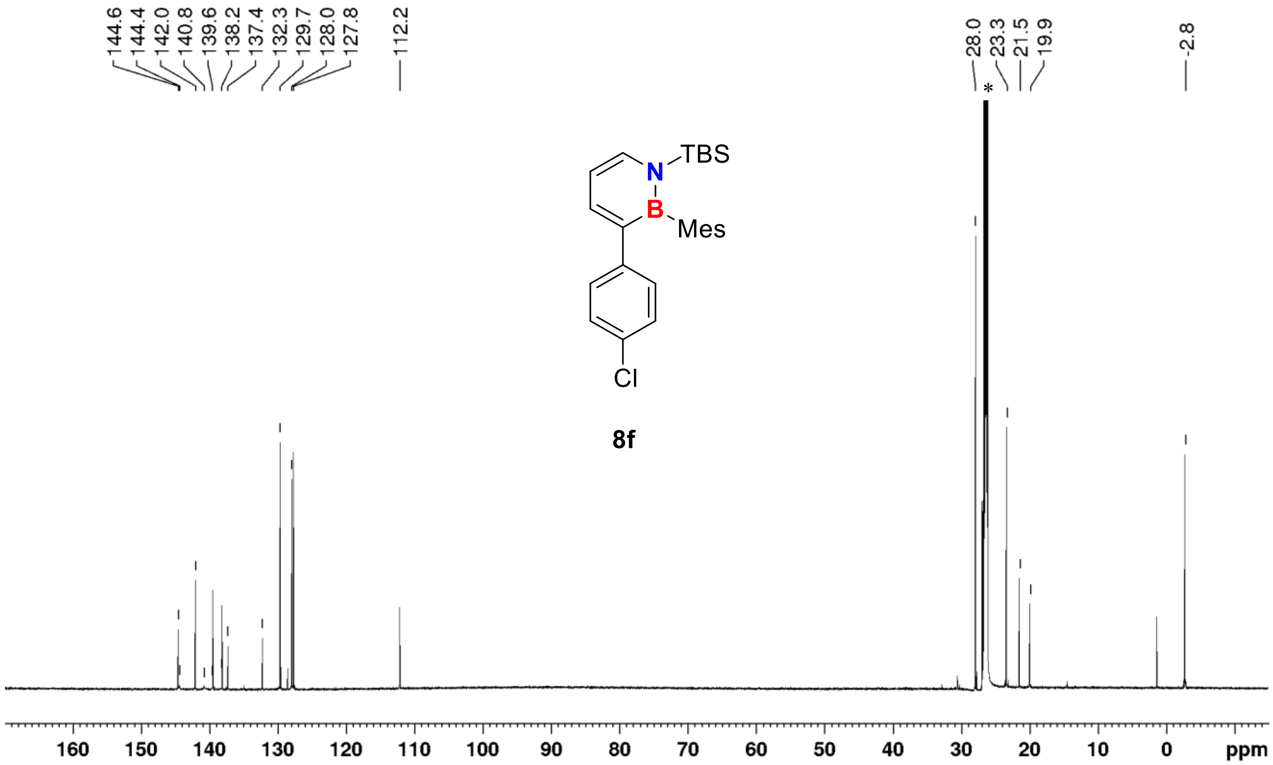

Figure S39. ^{13}C-{^{1}H}-NMR spectrum of compound **8f** in C_6D_{12} measured at a 600 MHz spectrometer. The solvent signal is marked with an asterisk.

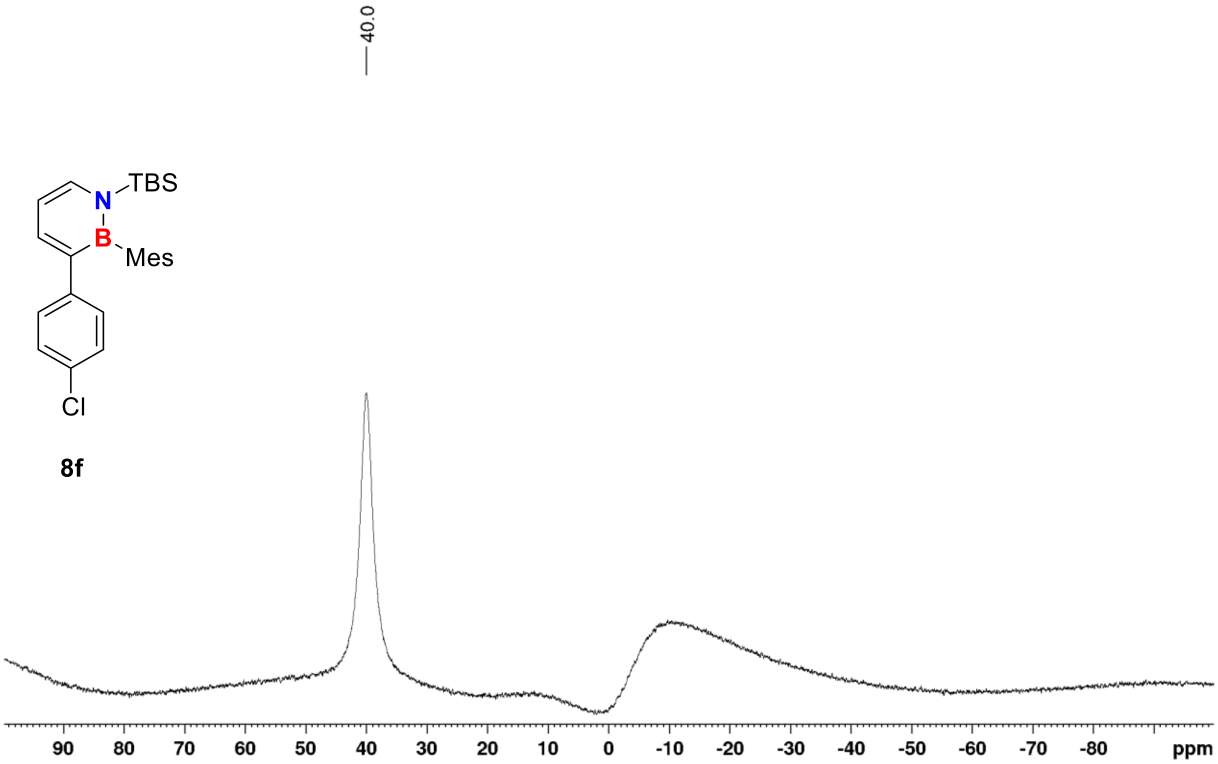

Figure S40. ^{11}B-{^{1}H}-NMR spectrum of compound **8f** in $CDCl_3$ measured at a 600 MHz spectrometer. The broad signal between 0 and 30 ppm corresponds to the borosilicate glass of the NMR tube.

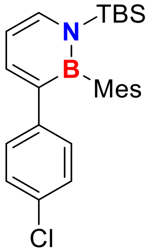

8f

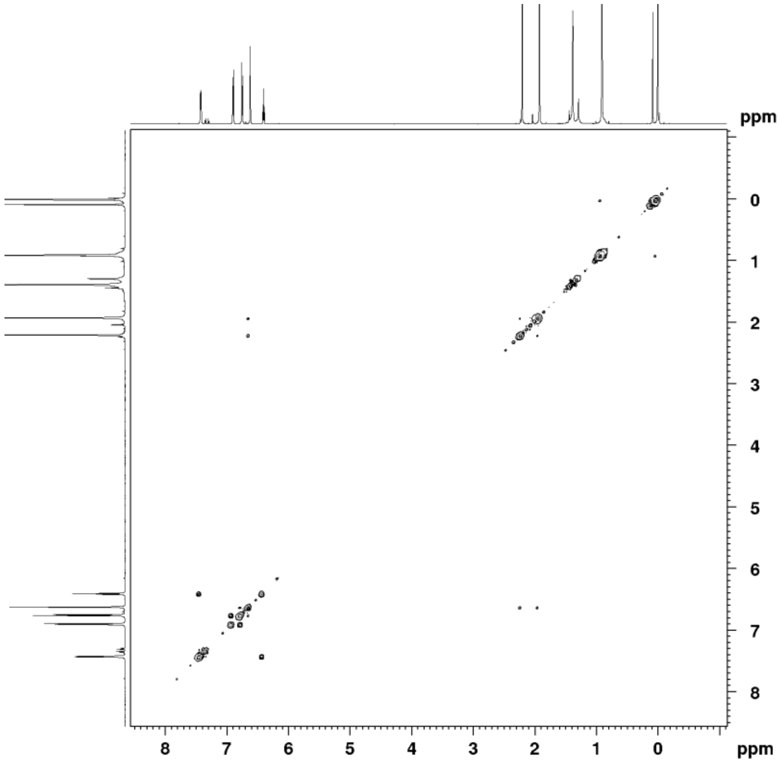

Figure S41. ^{1}H-^{1}H-COSY-NMR spectrum of compound **8f** in C_6D_{12} measured at a 600 MHz spectrometer.

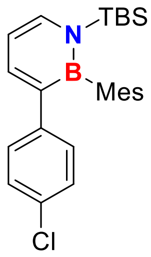

8f

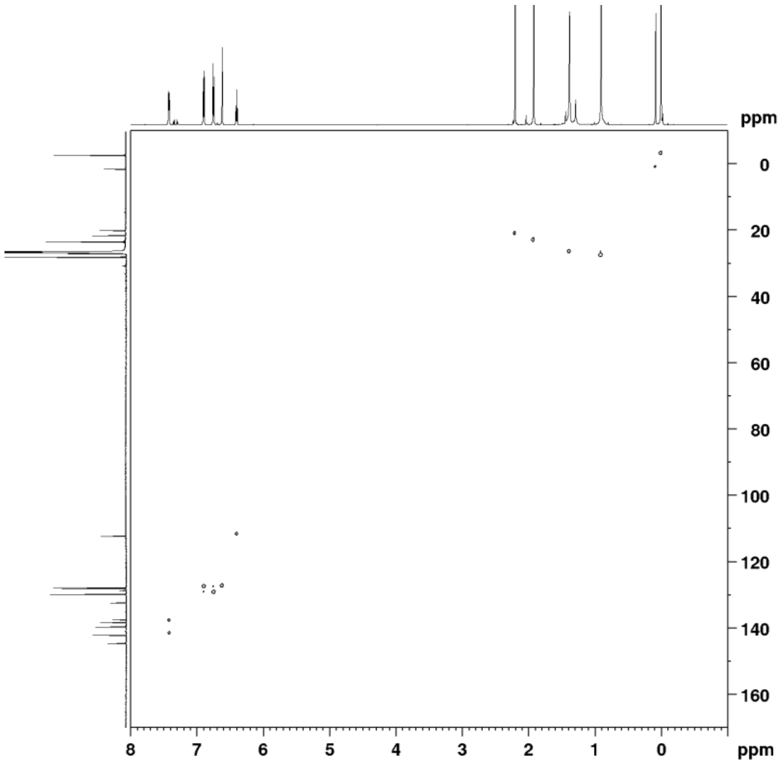

Figure S42. ^{1}H-^{13}C-HSQC-NMR spectrum of compound **8f** in C_6D_{12} measured at a 600 MHz spectrometer.

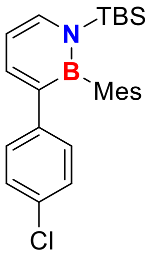

8f

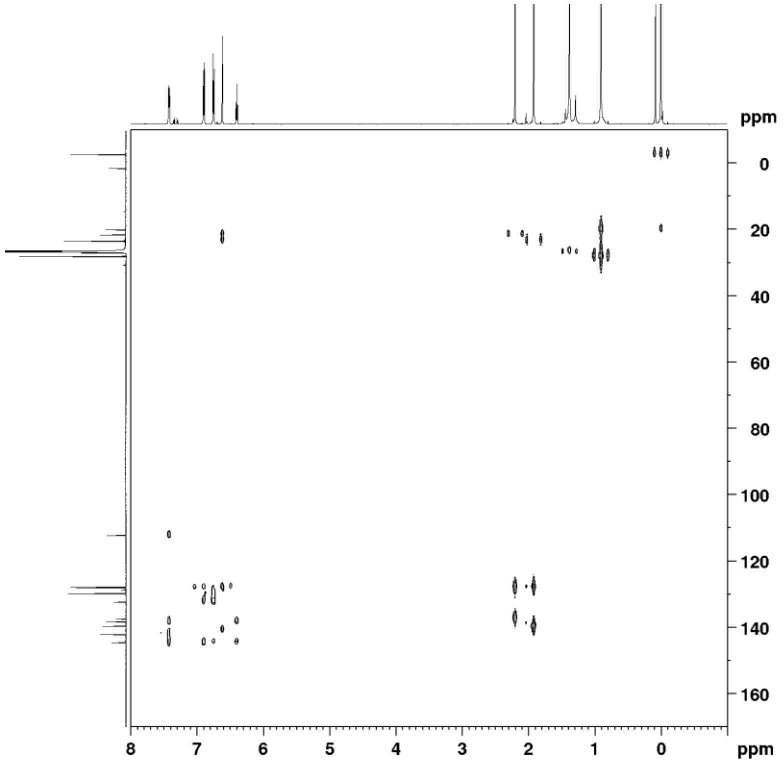

Figure S43. ^{1}H-^{13}C-HMBC-NMR spectrum of compound **8f** in C_6D_{12} measured at a 600 MHz spectrometer.

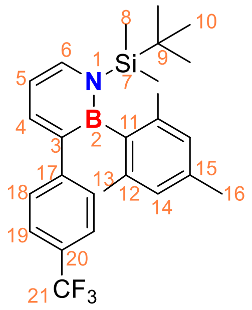

8g

$C_{26}H_{33}BF_3NSi$ (455.45 g/mol)

GC-FID: t_r = 12.555 min, conversion = 62 %.

Isolated yield: 51%, average of two runs on a 100 µmol scale (48 % and 54%).

^{1}H-NMR (600 MHz, **C_6D_{12}**): δ = 7.47 (ps. dd, 2H, H-4/H-6), 7.20 (d, $^3J_{HH}$ = 8.14 Hz, 2H, H-19), 6.94 (d, $^3J_{HH}$ = 8.14 Hz, 2H, H-18), 6.63 (s, 2H , H-14), 6.44 (ps. t, 1H, H-5), 2.20 (s, 3H, H-16), 1.92 (s, 6H, H-13), 0.91 (s, 9H, H-10), 0.01 (s, 6H, H-8) ppm.

^{13}C-{^{1}H}-NMR (151 MHz, **C_6D_{12}**): δ = 149.9 (C21), 144.1 (C3), 142.6 (C4), 140.5 (C11), 139.5 (C12), 138.9 (C6), 137.6 (C15), 128.7 (C18), 127.8 (C14), 124.7 (C19), 112.2 (C5), 27.9 (C10), 23.3 (C13), 21.4 (C16), 19.9 (C9), -2.8 (C8) ppm.

^{11}B-{^{1}H}-NMR (192 MHz, **C_6D_{12}**): δ = 40.1 ppm.

HR-MS (APCI): m/z calc. For [M+H]+ 456.25051, found 456.24966.

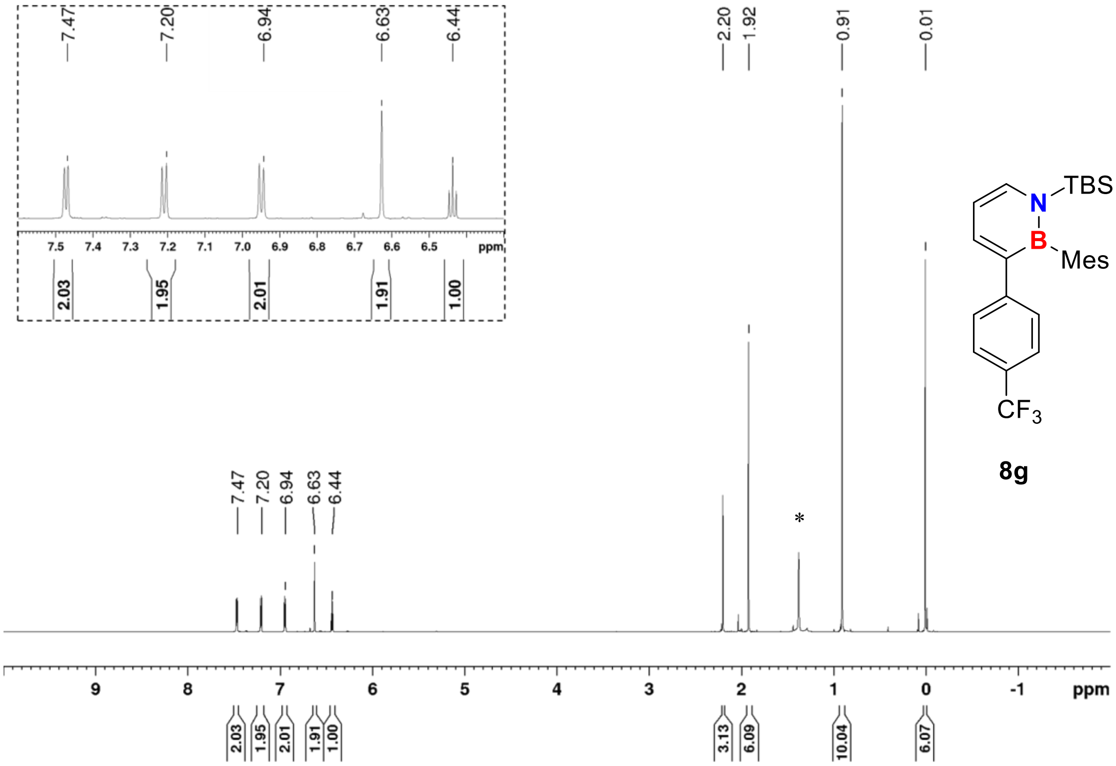

Figure S44. ^{1}H-NMR spectrum of compound **8g** in C_6D_{12} measured at a 600 MHz spectrometer. The enlarged section shows the region between 6.3 and 7.6 ppm for a better visibility of the aromatic signals. The solvent signal is marked with an asterisk.

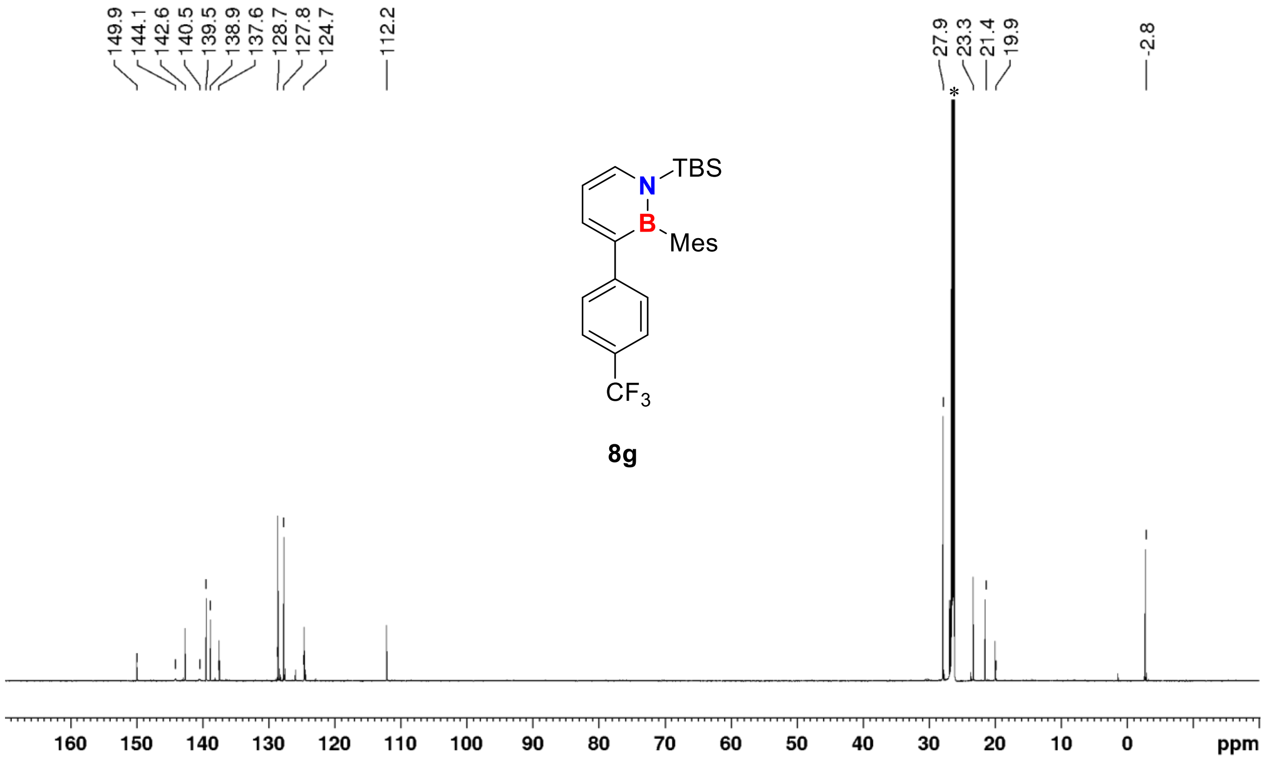

Figure S45. ^{13}C-{^{1}H}-NMR spectrum of compound **8g** in C_6D_{12} measured at a 600 MHz spectrometer. The solvent signal is marked with an asterisk.

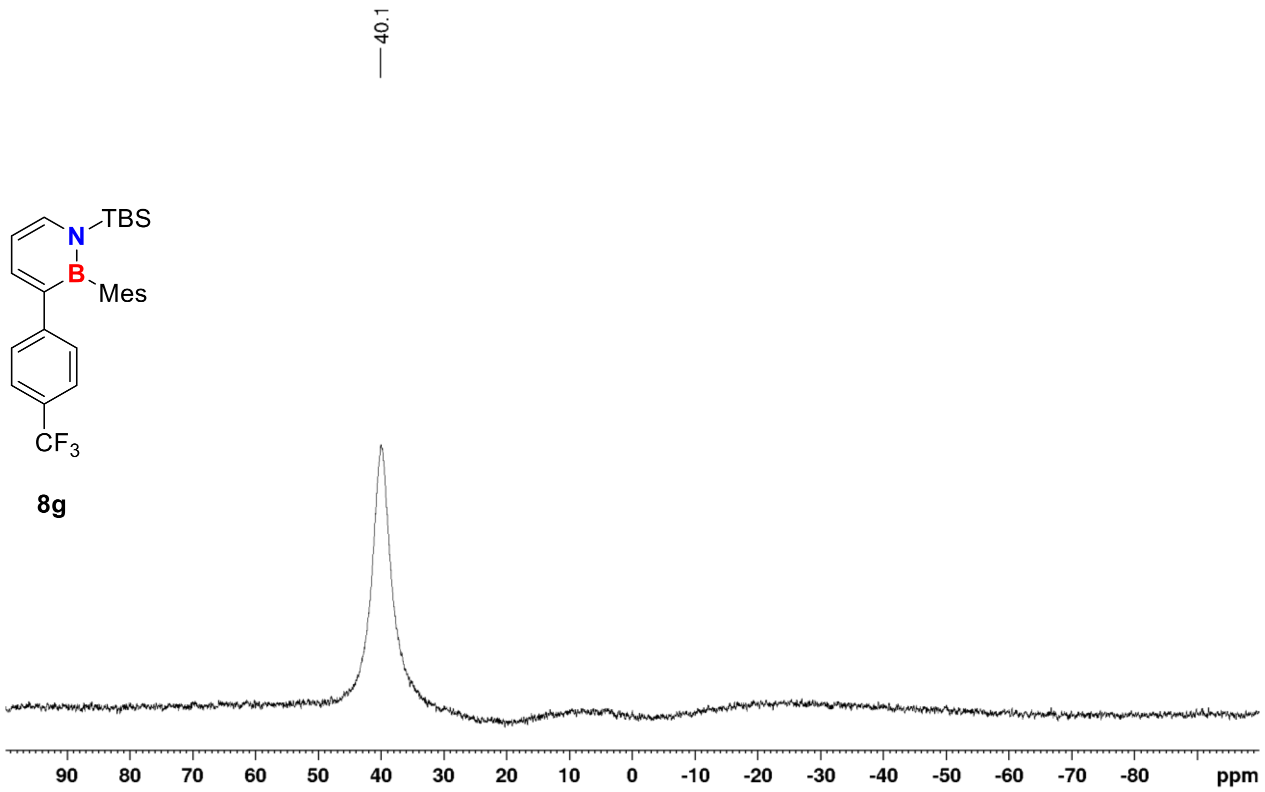

Figure S46. ^{11}B-{^{1}H}-NMR spectrum of compound **8g** in C_6D_{12} measured at a 600 MHz spectrometer.

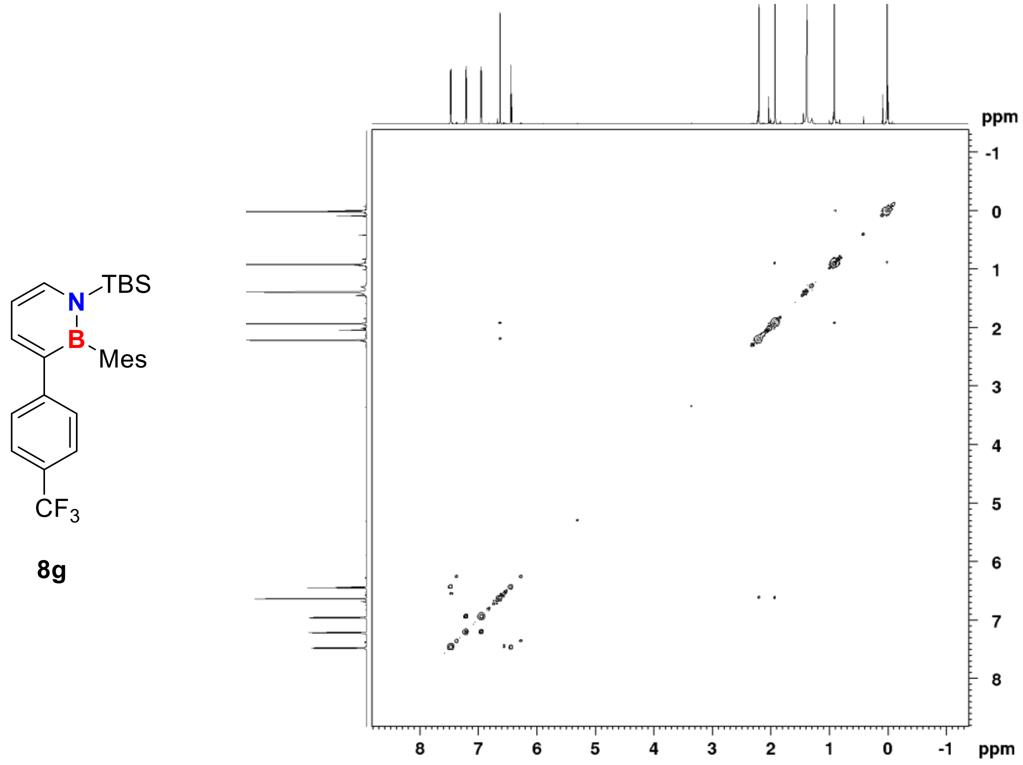

Figure S47. ^{1}H-^{1}H-COSY-NMR spectrum of compound **8g** in C_6D_{12} measured at a 600 MHz spectrometer.

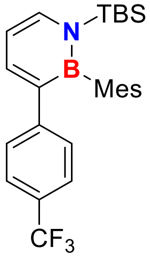

8g

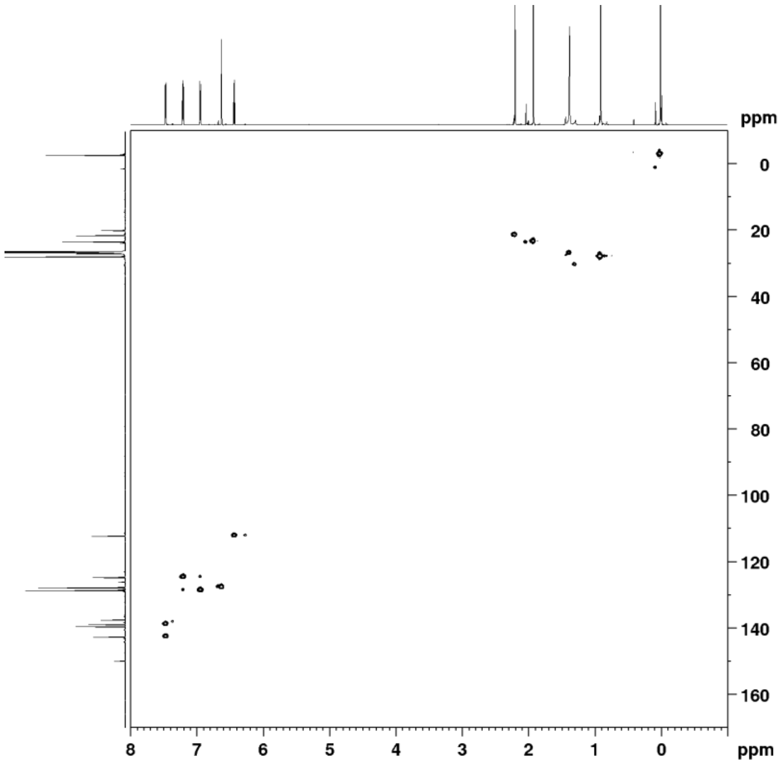

Figure S48. ^{1}H-^{13}C-HSQC-NMR spectrum of compound **8g** in C_6D_{12} measured at a 600 MHz spectrometer.

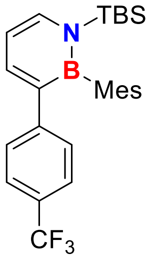

8g

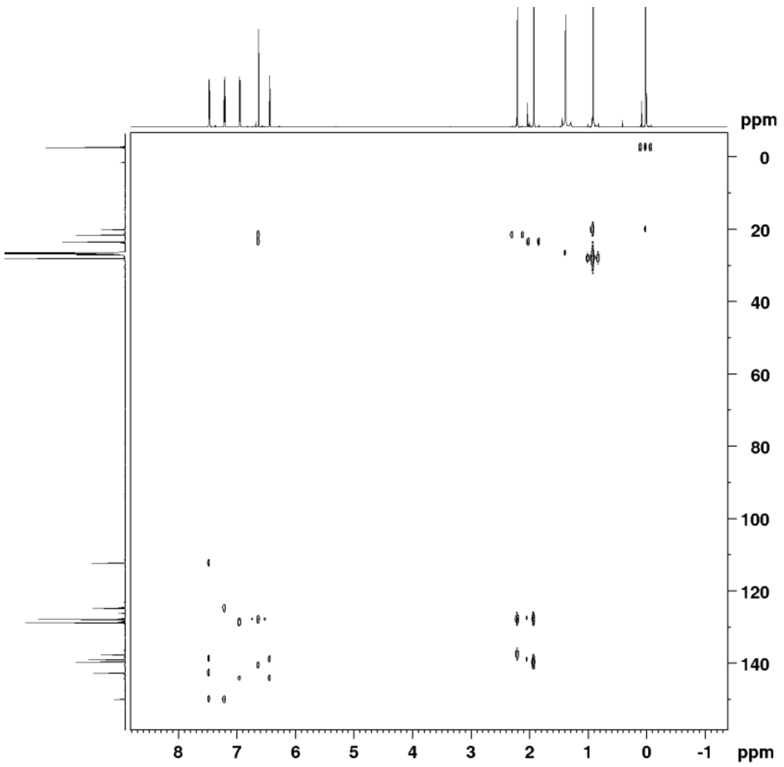

Figure S49. ^{1}H-^{13}C-HMBC-NMR spectrum of compound **8g** in C_6D_{12} measured at a 600 MHz spectrometer.

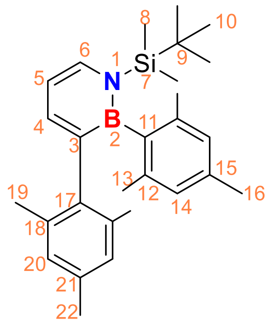

8h

$C_{28}H_{40}BNSi$ (429.53 g/mol)

GC-FID: t_r = 42.513 min, conversion = 73 %.

Isolated yield: 61%, average of two runs on a 100 µmol scale (59 % and 63%).

^{1}H-NMR (700 MHz, **C_6D_{12}**): δ = 7.40 (dd, $^3J_{HH}$ = 6.63 Hz, 1H, H-4), 7.12 (dd, $^3J_{HH}$ = 6.99 Hz, 1H, H-6), 6.57 (s, 2H, H-20), 6.50 (s, 2H, H-14), 6.37 (ps. t, 1H, H-5), 2.13 (s, 6H, H-16/H-22), 1.88 (s, 6H, H-13), 1.79 (s, 6H, H-19), 0.85 (s, 9H, H-10), -0.01 (s, 6H, H-8) ppm.

^{13}C-{^{1}H}-NMR (151 MHz, **C_6D_{12}**): δ = 146.0 (C3), 143.4 (C6), 142.0 (C17), 140.7 (C11), 140.3 (C12), 136.9 (C15), 136.9 (C4), 135.6 (C18), 134.5 (C21), 128.4 (C20), 127.7 (C14), 111.4 (C5), 27.7 (C10), 23.5 (C13), 21.4 (C19), 21.4 (C19), 21.1 (C16/C22), 19.8 (C9), -3.4 (C8) ppm.

^{11}B-{^{1}H}-NMR (192 MHz, **C_6D_{12}**): δ = 42.0 ppm.

HR-MS (APCI): m/z calc. For [M+H]+ 430.31011, found 430.30966.

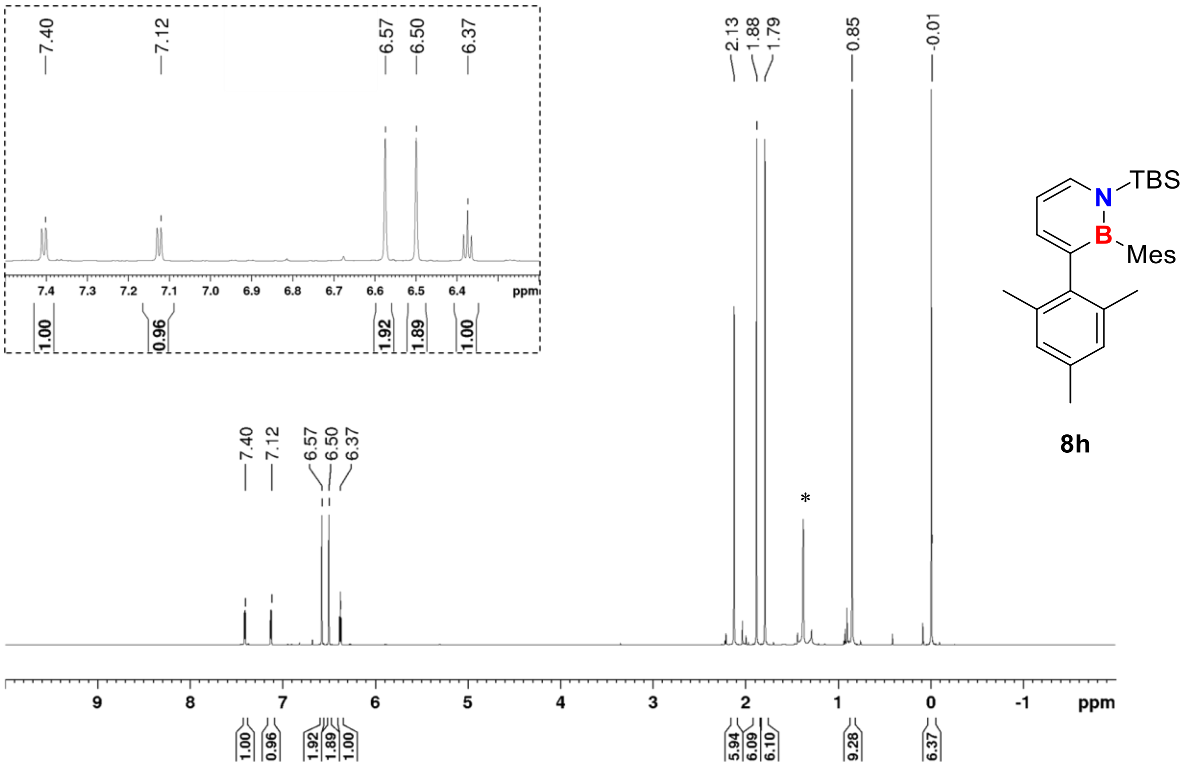

Figure S50. ^{1}H-NMR spectrum of compound **8h** in C_6D_{12} measured at a 700 MHz spectrometer. The enlarged section shows the region between 6.2 and 7.5 ppm for a better visibility of the aromatic signals. The solvent signal is marked with an asterisk.

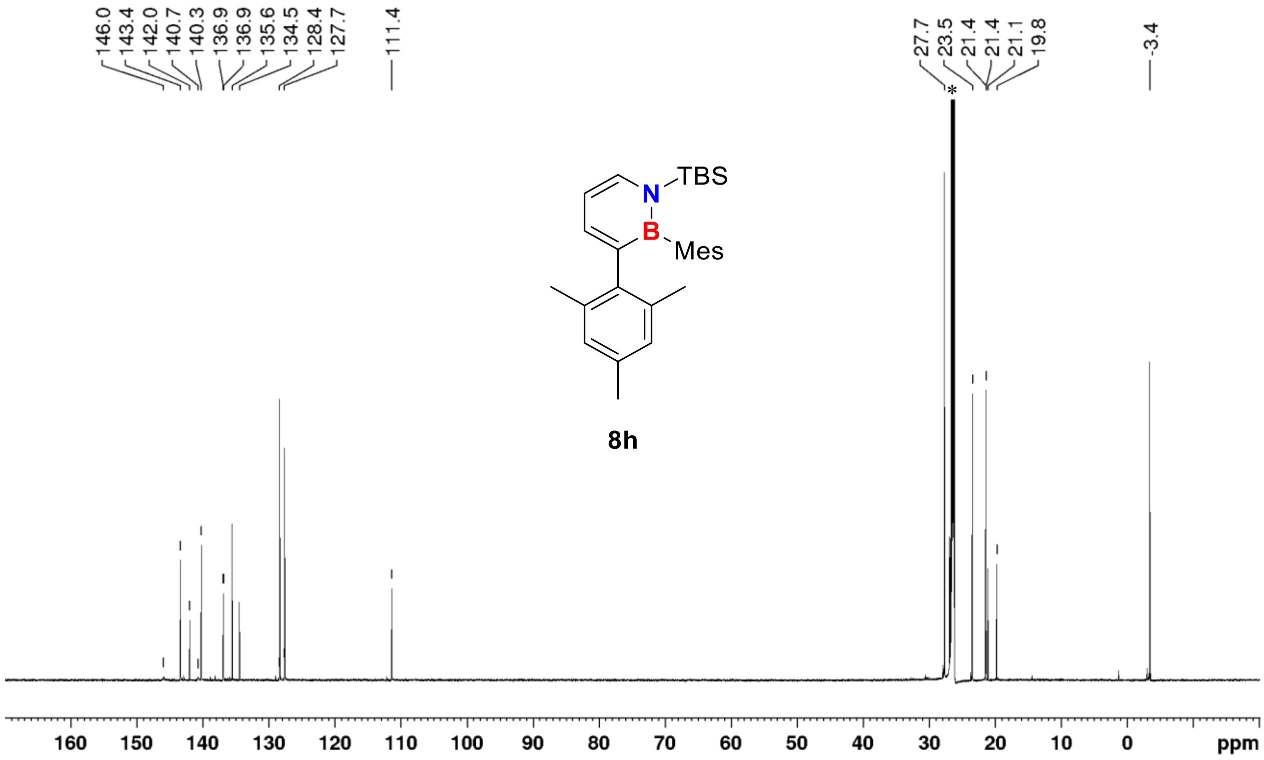

Figure S51. ^{13}C-{^{1}H}-NMR spectrum of compound **8h** in C_6D_{12} measured at a 600 MHz spectrometer. The solvent signal is marked with an asterisk.

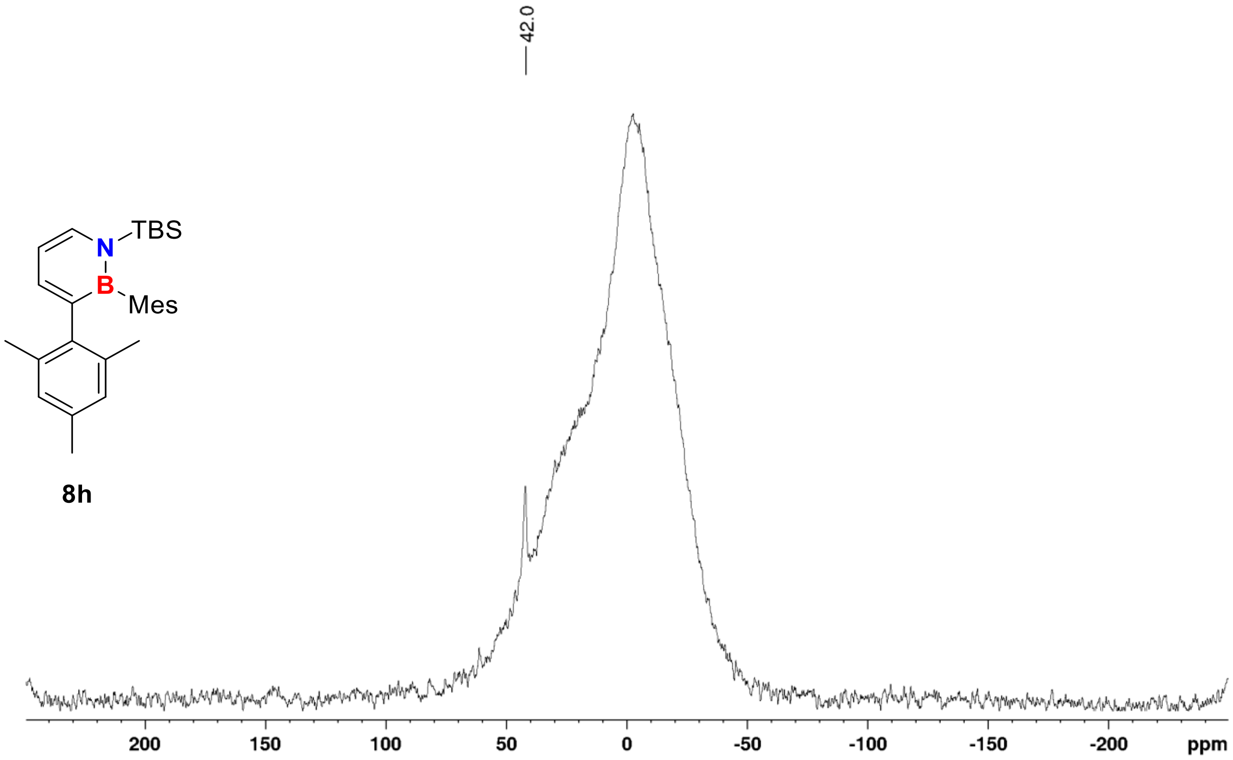

8h

Figure S52. ^{11}B-{^{1}H}-NMR spectrum of compound **8h** in C_6D_{12} measured at a 600 MHz spectrometer. The broad signal between -50 and 50 ppm corresponds to the borosilicate glass of the NMR tube.

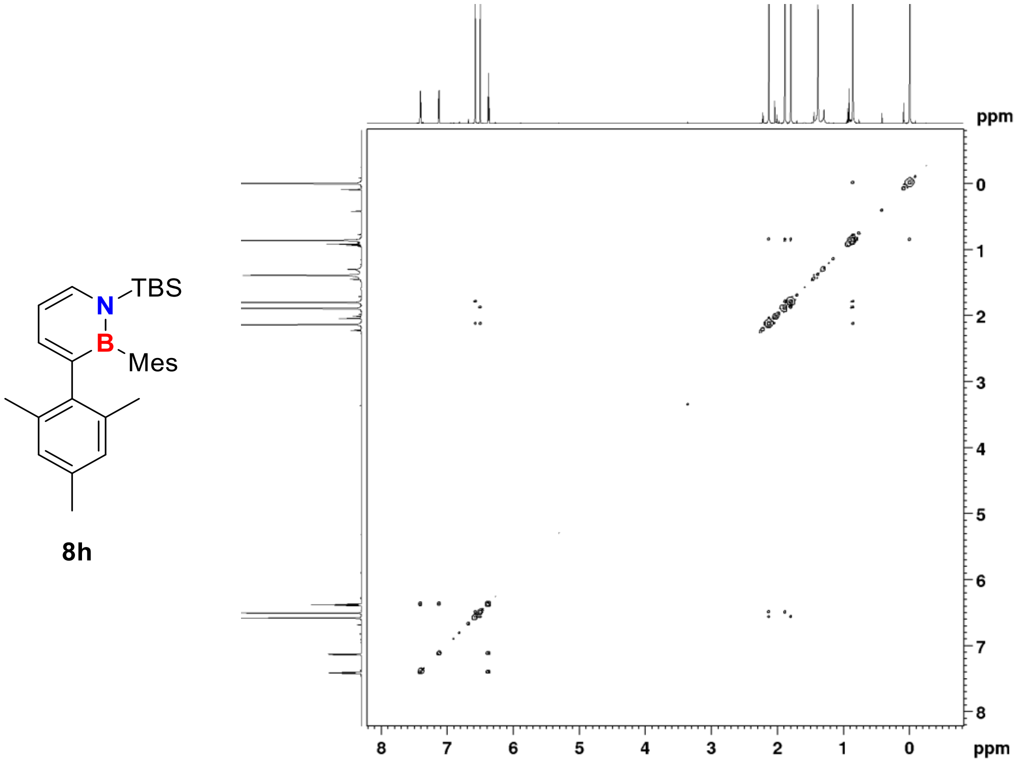

8h

Figure S53. ^{1}H-^{1}H-COSY-NMR spectrum of compound **8h** in C_6D_{12} measured at a 600 MHz spectrometer.

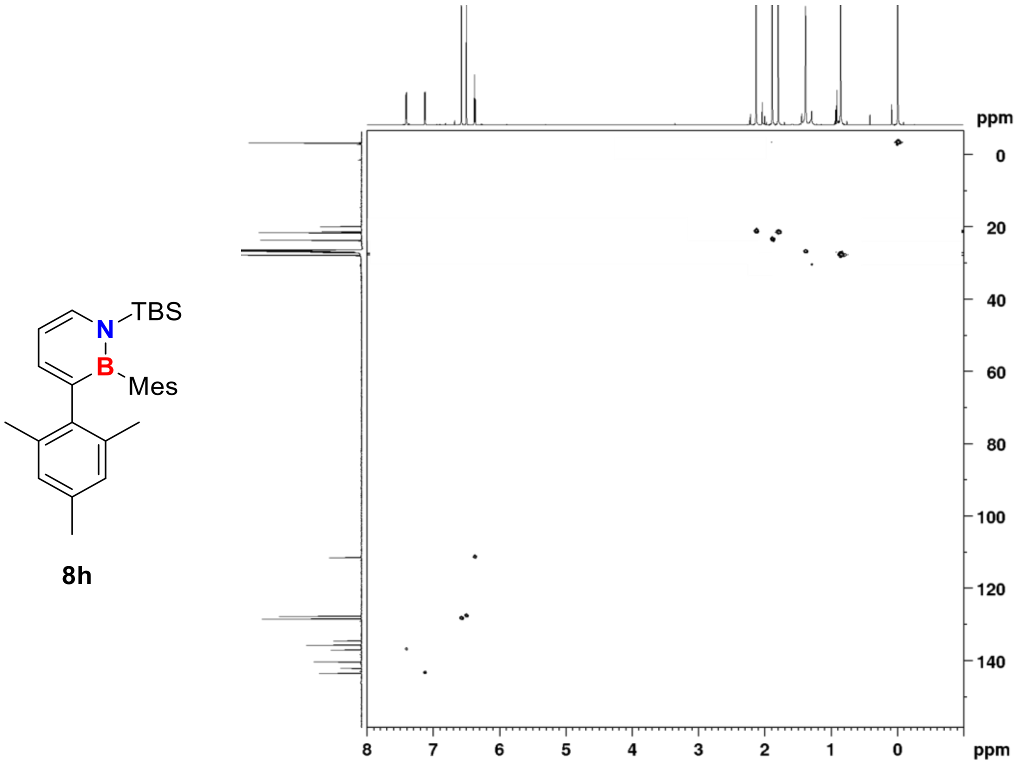

Figure S54. ^{1}H-^{13}C-HSQC-NMR spectrum of compound **8h** in C_6D_{12} measured at a 600 MHz spectrometer.

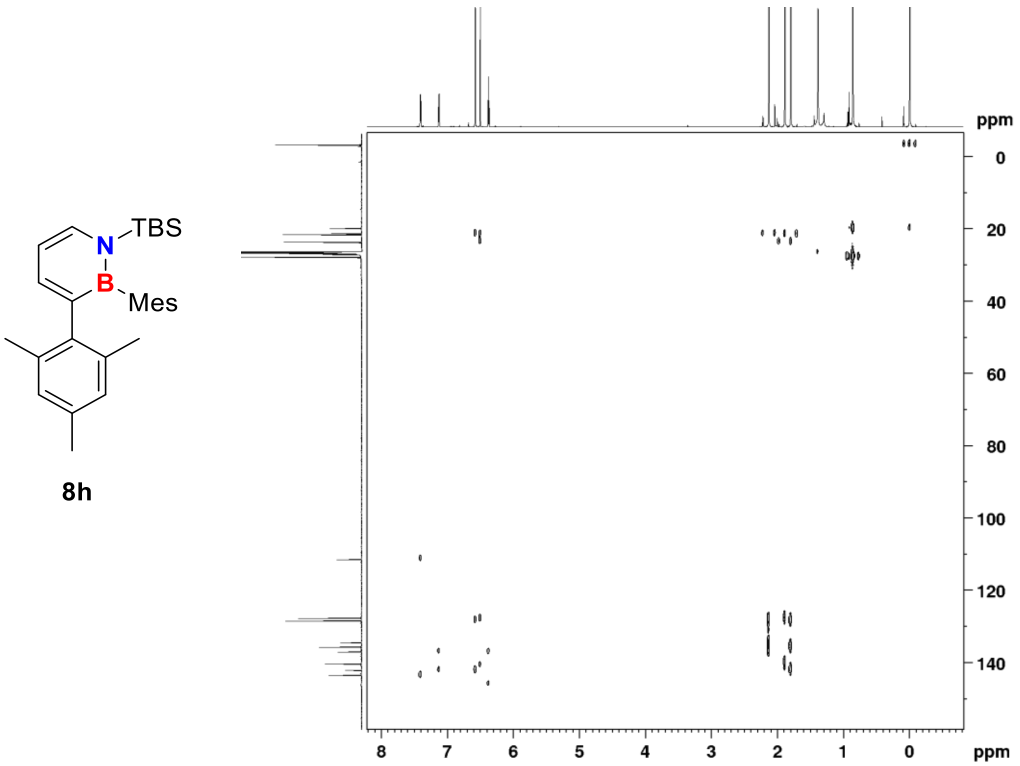

Figure S55. ^{1}H-^{13}C-HMBC-NMR spectrum of compound **8h** in C_6D_{12} measured at a 600 MHz spectrometer.

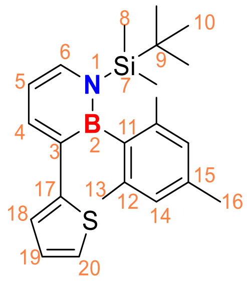

8i

$C_{23}H_{32}BNSSi$ (393.47 g/mol)

GC-FID: t_r = 41,095 min, conversion = quant.

Isolated yield: 95%

^{1}H-NMR (600 MHz, **C_6D_{12}**): δ = 7.59 (dd, $^3J_{HH}$ = 6.86 Hz, $^4J_{HH}$ = 1.10 Hz, 1H, H-4), 7,36 (dd, $^3J_{HH}$ = 6.81 Hz, $^4J_{HH}$ = 1.10 Hz, 1H, H-6), 6. 84 (dd, $^3J_{HH}$ = 5.11 Hz, $^3J_{HH}$ = 3.11 Hz, 1H, H-20), 6.71 (dd, $^3J_{HH}$ = 5.11 Hz, $^4J_{HH}$ = 1.30 Hz, 1H, H-21), 6.67 (s, 2H, H-14), 6.37 (dd, $^3J_{HH}$ = 3.11 Hz, $^4J_{HH}$ = 1.30 Hz, 1H, H-19), 6.37(ps. t, 1H, H-5), 2.24 (s, 3H, H-16), 1.94 (s, 6H, H-13), 0.91 (s, 9H, H-10), -0.01 (s, 6H, H-8) ppm.

^{13}C-{^{1}H}-NMR (151 MHz, **C_6D_{12}**): δ = 145.9 (C17), 141.7 (C11), 140.5 (C4), 139.8 (C12), 139.2 (C3), 137.4 (C6), 137.4 (C15), 127.9 (C21), 127.8 (C14), 123.5 (C20), 120.2 (C19), 112.1 (C5), 32.8 (*n-hexane*), 30.2 (*n-hexane*), 28.0 (C10), 23.5, 23.1 (C16), 21.5 (C13), 19.9 (C9), 14.4 (*n-hexane*), -2.7 (C8) ppm.

^{11}B-{^{1}H}-NMR (192 MHz, **C_6D_{12}**): δ = 40.1 ppm.

HR-MS (APCI): m/z calc. For [M+H]+ 394.21950, found 394.21935.

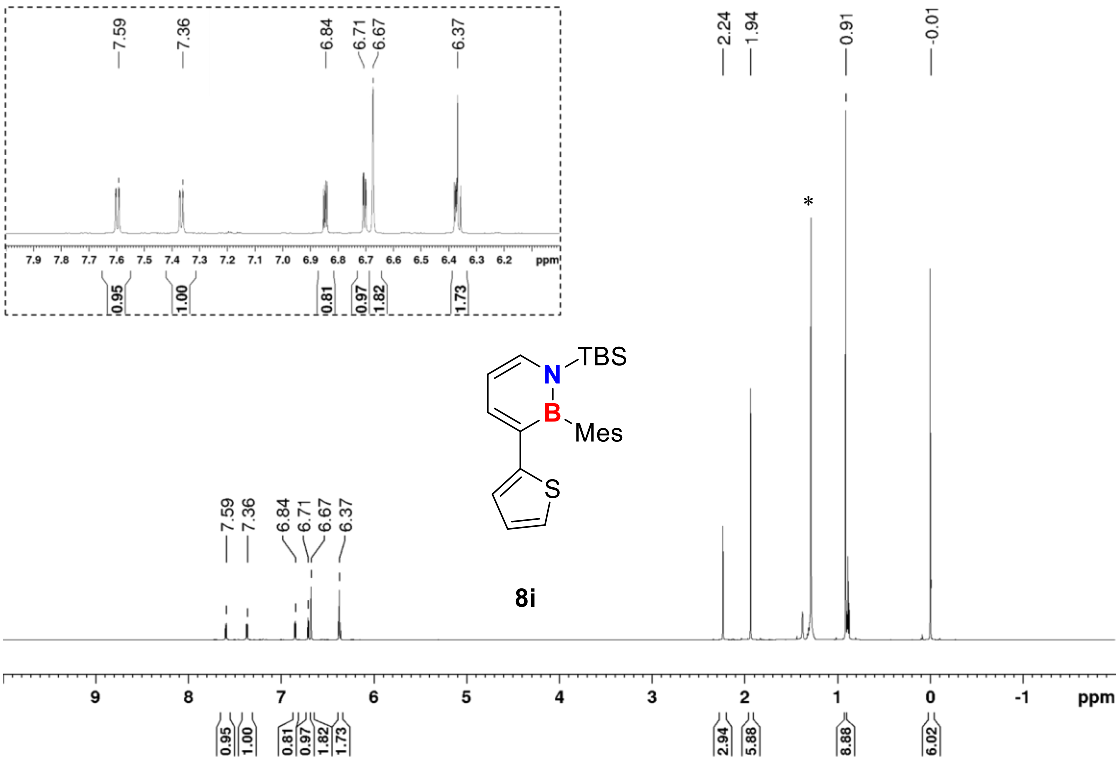

Figure S56. ^{1}H-NMR spectrum of compound **8i** in C_6D_{12} measured at a 600 MHz spectrometer. The enlarged section shows the region between 6 and 8 ppm for a better visibility of the aromatic signals. The solvent signal is marked with an asterisk.

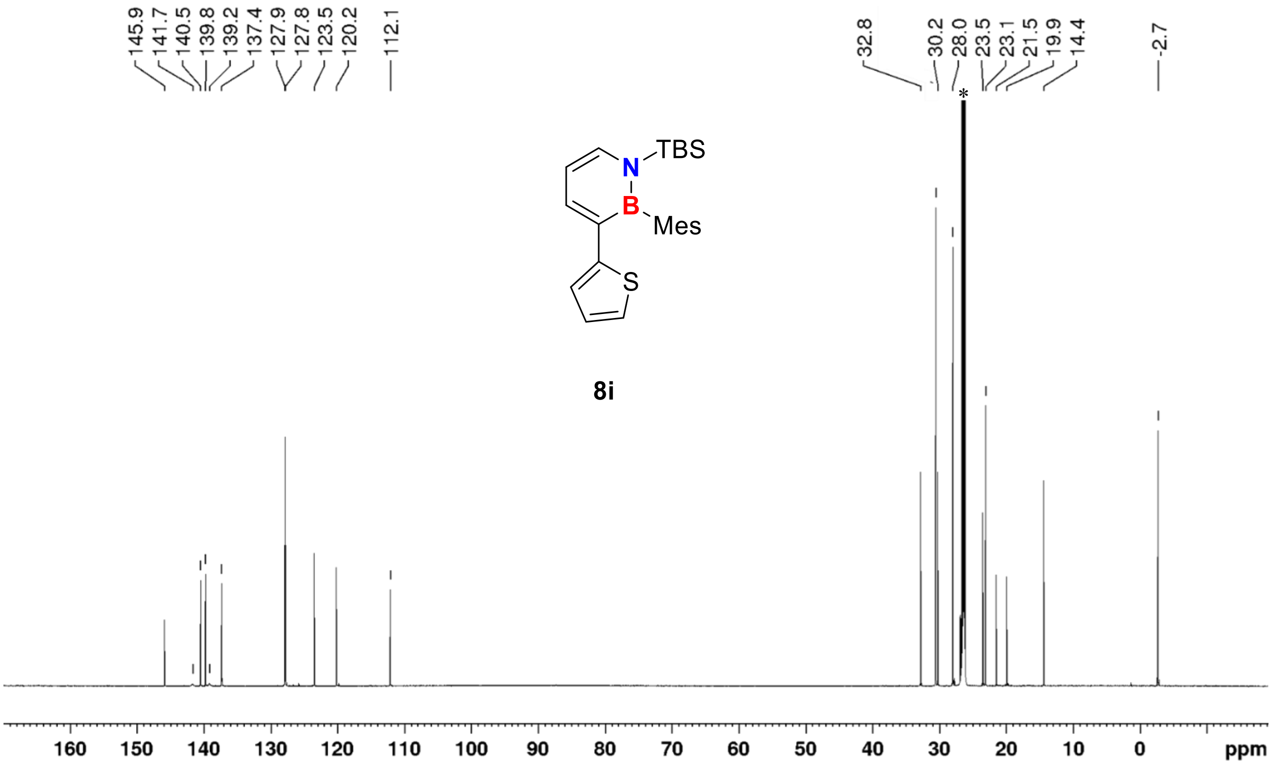

Figure S57. ^{13}C-{^{1}H}-NMR spectrum of compound **8i** in C_6D_{12} measured at a 600 MHz spectrometer. The solvent signal is marked with an asterisk.

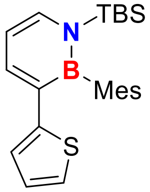

8i

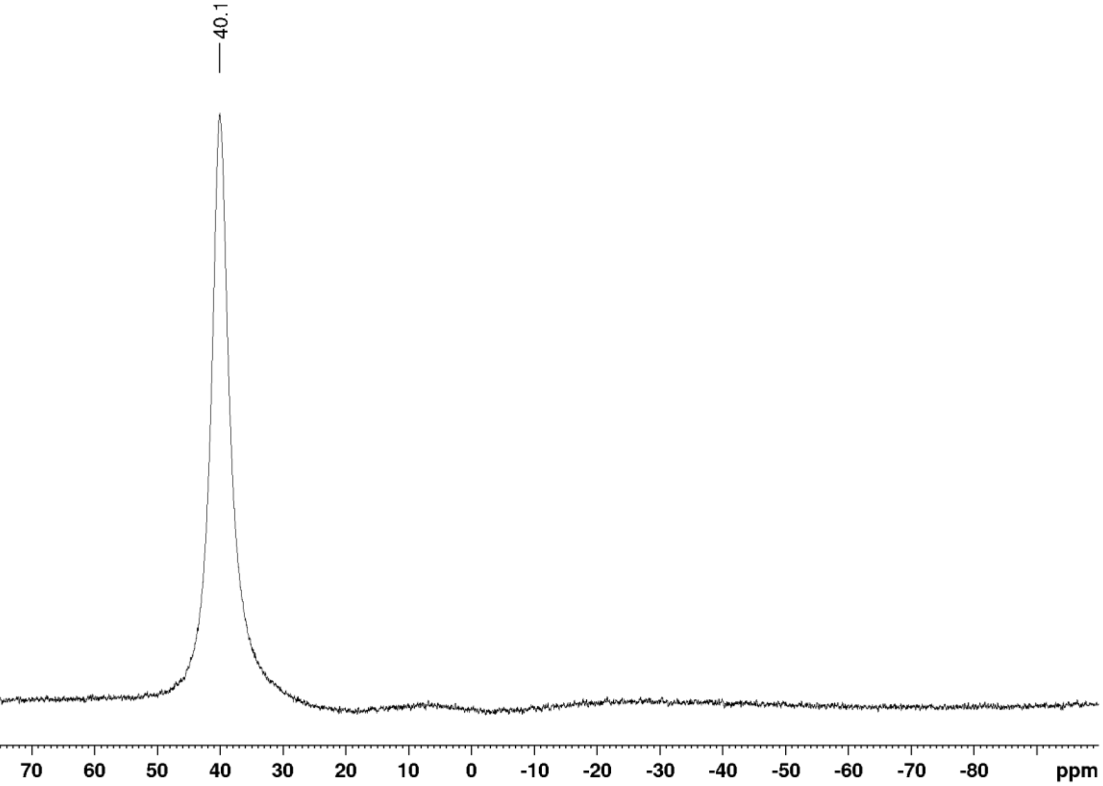

Figure S58. ^{11}B-{^{1}H}-NMR spectrum of compound **8i** in C_6D_{12} measured at a 600 MHz spectrometer.

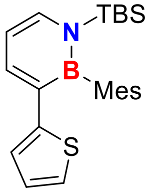

8i

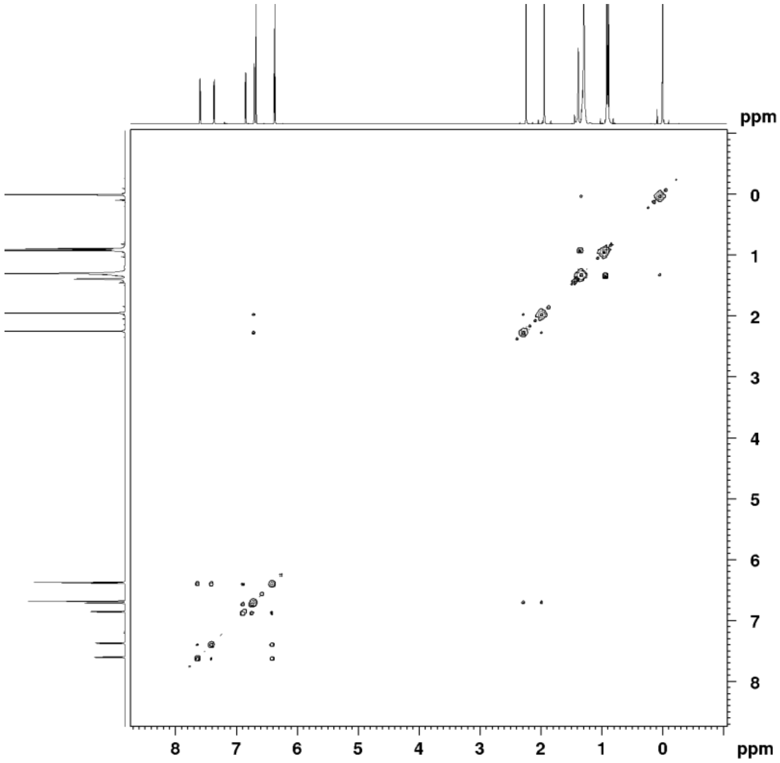

Figure S59. ^{1}H-^{1}H-COSY-NMR spectrum of compound **8i** in C_6D_{12} measured at a 600 MHz spectrometer.

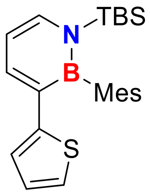

8i

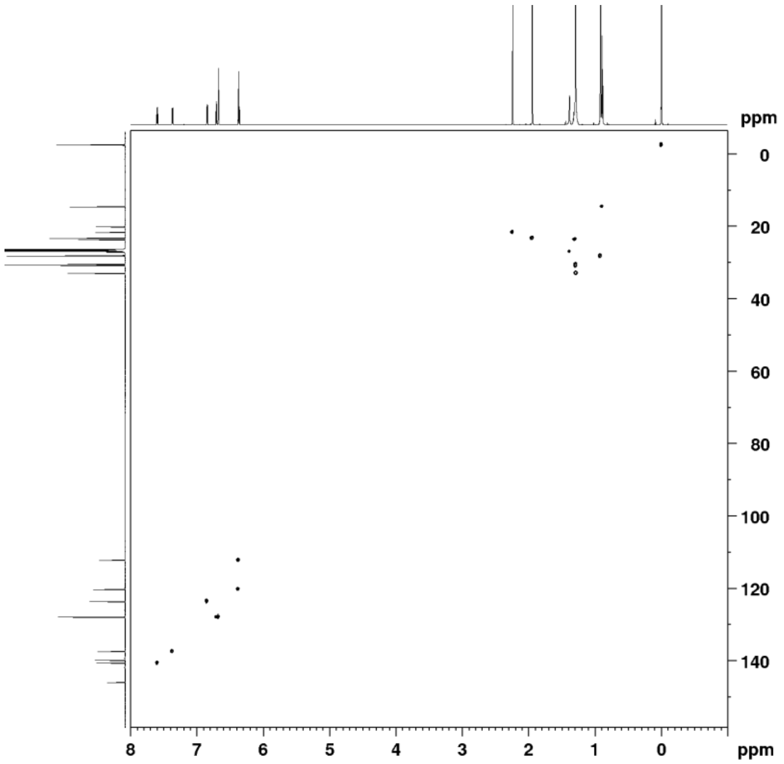

Figure S60. ^{1}H-^{13}C-HSQC-NMR spectrum of compound **8i** in C_6D_{12} measured at a 600 MHz spectrometer.

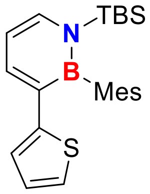

8i

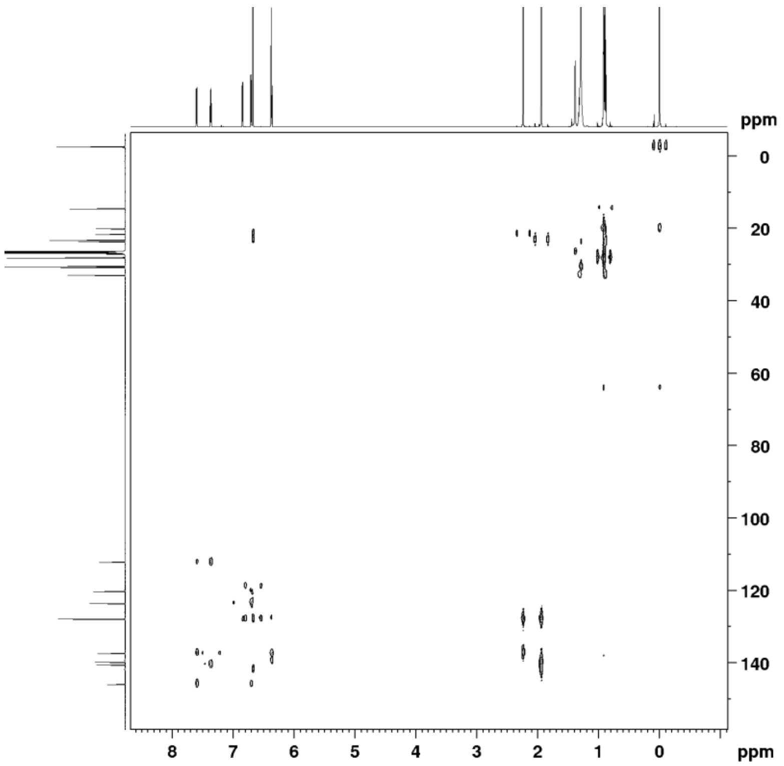

Figure S61. ^{1}H-^{13}C-HMBC-NMR spectrum of compound **8i** in C_6D_{12} measured at a 600 MHz spectrometer.

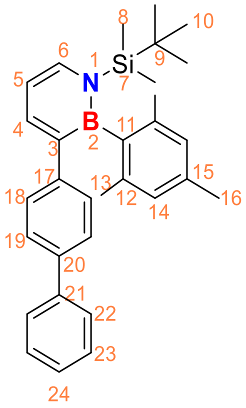

8j

$C_{31}H_{38}BNSi$ (463.55 g/mol)

GC-FID: t_r = 50.650 min, conversion = 88 %.

Isolated yield: 78%, average of two runs on a 100 µmol scale (76 % and 80%).

^{1}H-NMR (700 MHz, **C_6D_{12}**): δ = 7.50 (dd, $^{3}J_{HH}$ = 6.91 Hz, $^{4}J_{HH}$ = 0.95 Hz, 1H, H-4), 7.42 (dd, $^{3}J_{HH}$ = 6.87 Hz, $^{4}J_{HH}$ = 0.95 Hz, 1H, H-6), 7.93 (dm, $^{3}J_{HH}$ = 8.47 Hz, 2H, H-22), 7.22 (ps. t, 2H, H-23), 7.19 (dm, $^{3}J_{HH}$ = 8.26 Hz, 2H, H-19), 7.11 (ps.t, 1H, H-24), 6.91 (dm, $^{3}J_{HH}$ = 8.26 Hz, 2H, H-18), 6.62 (s, 2H, H-14), 6.42 (ps. t, 1H, H-5), 2,19 (s, 3H, H-16), 1.96 (s, 6H, H-13), 0.91 (s, 9H, H-10), 0.01 (s, 6H, H-8) ppm.

^{13}C-{^{1}H}-NMR (151 MHz, **C_6D_{12}**): δ = 145.2 (C17), 142.3 (C21), 141.9 (C4), 141.1 (C11), 139.7 (C12), 138.7 (C20), 137.8 (C6), 137.1 (C15), 129.0 (C18), 128.8 (C23), 127.7 (C14), 127.3 (C22), 126.9 (C24), 126.5 (C19), 112.3 (C5), 28.0 (C10), 23.4 (C13), 21.5 (C16), 19.9 (C9), -2.8 (C8) ppm.

^{11}B-{^{1}H}-NMR (192 MHz, **C_6D_{12}**): δ = 40.0 ppm.

HR-MS (APCI): m/z calc. For [M+H]+ 463.28669, found 463.28659.

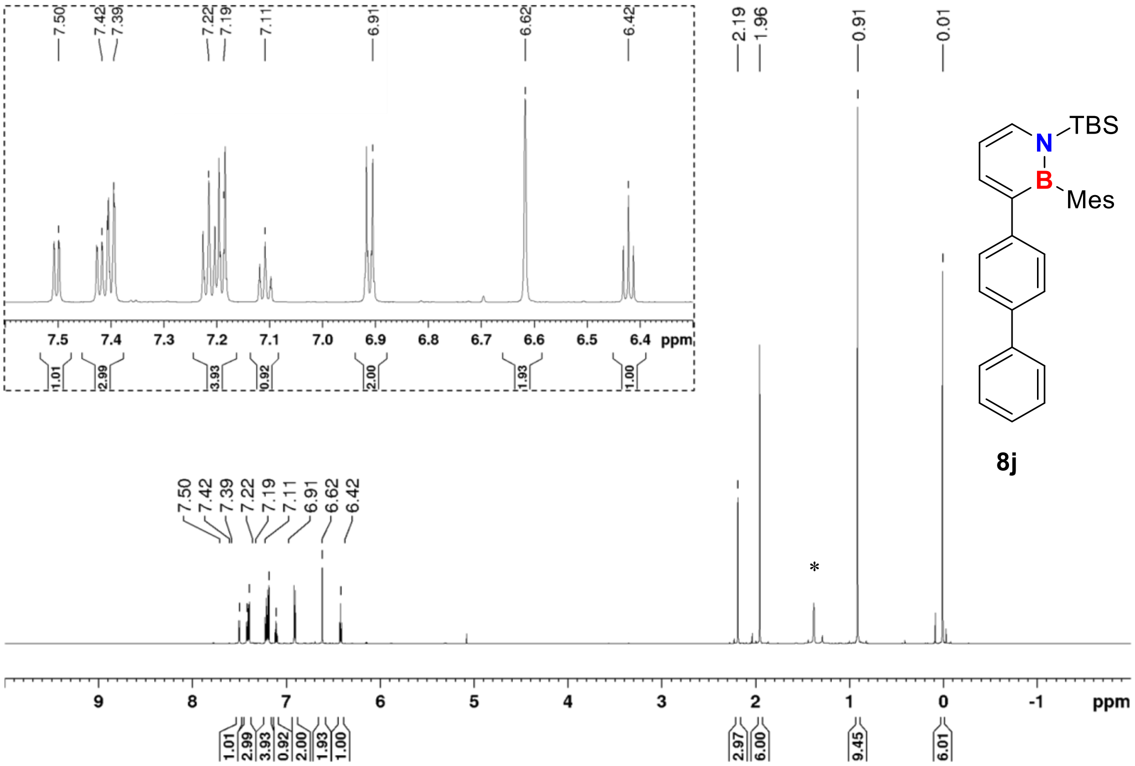

Figure S62. ^{1}H-NMR spectrum of compound **8j** in C_6D_{12} measured at a 700 MHz spectrometer. The enlarged section shows the region between 6.3 and 7.6 ppm for a better visibility of the aromatic signals. The solvent signal is marked with an asterisk.

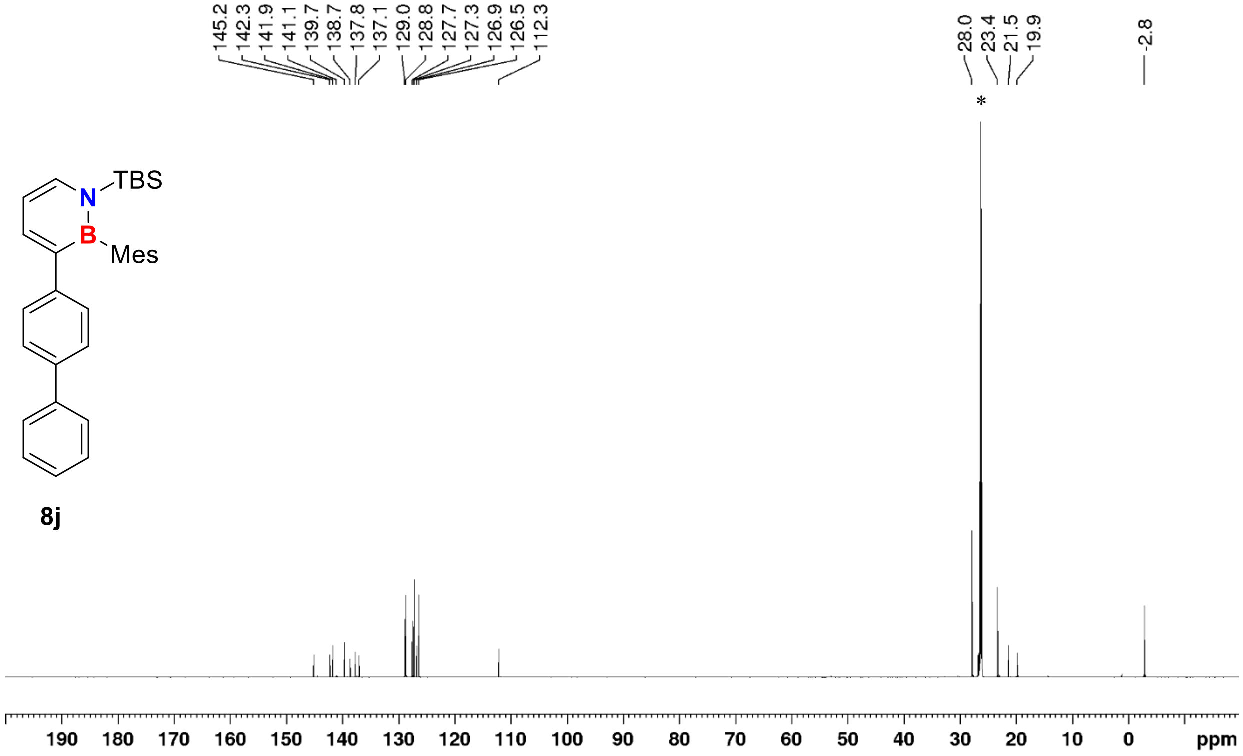

Figure S63. ^{13}C-{^{1}H}-NMR spectrum of compound **8j** in C_6D_{12} measured at a 600 MHz spectrometer. The solvent signal is marked with an asterisk.

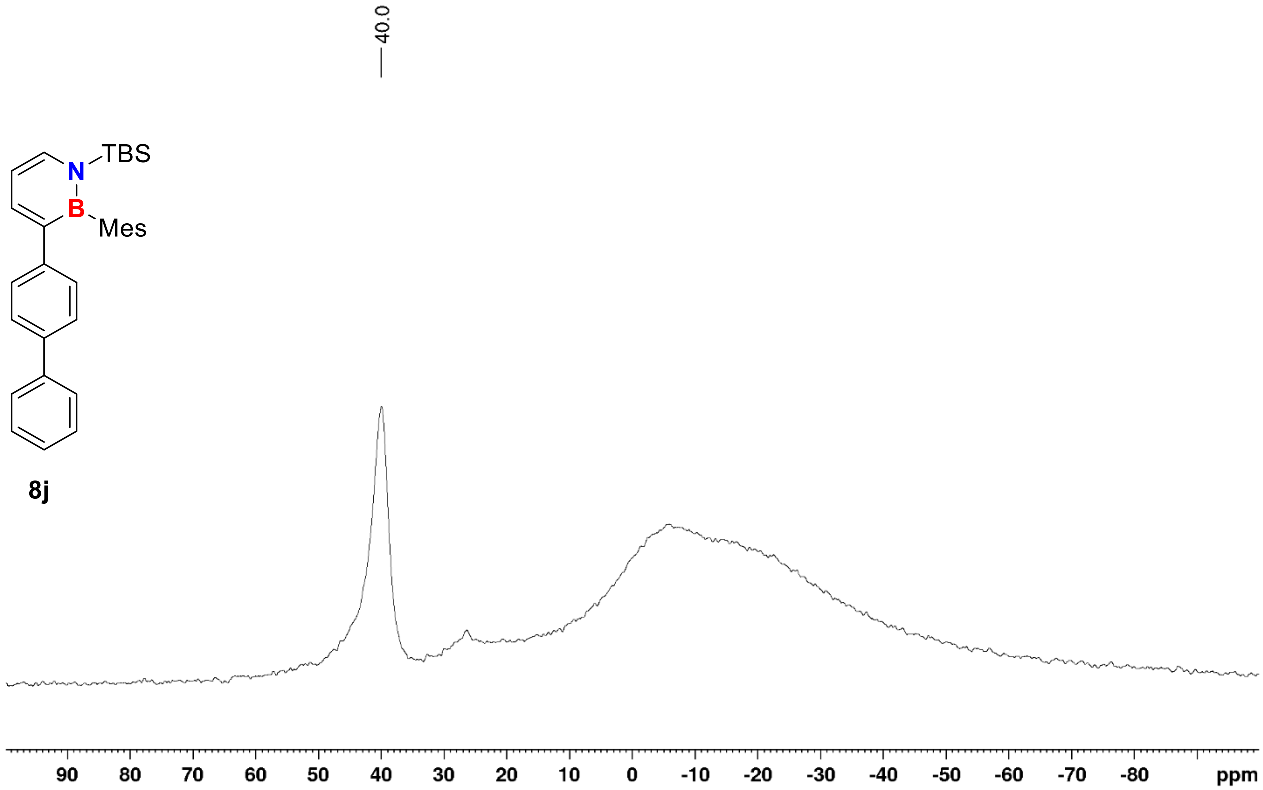

Figure S64. ^{11}B-{^{1}H}-NMR spectrum of compound **8j** in C_6D_{12} measured at a 600 MHz spectrometer. The broad signal between -30 and 30 ppm corresponds to the borosilicate glass of the NMR tube.

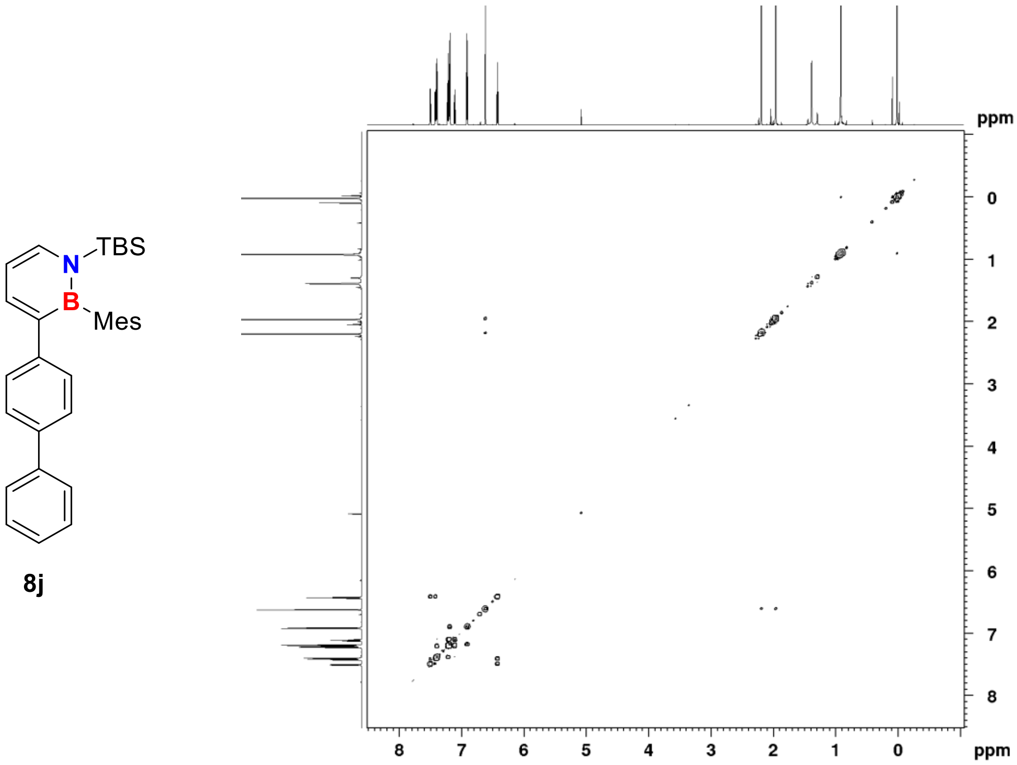

Figure S65. ^{1}H-^{1}H-COSY-NMR spectrum of compound **8j** in C_6D_{12} measured at a 600 MHz spectrometer.

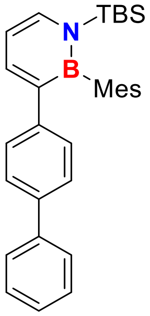

8j

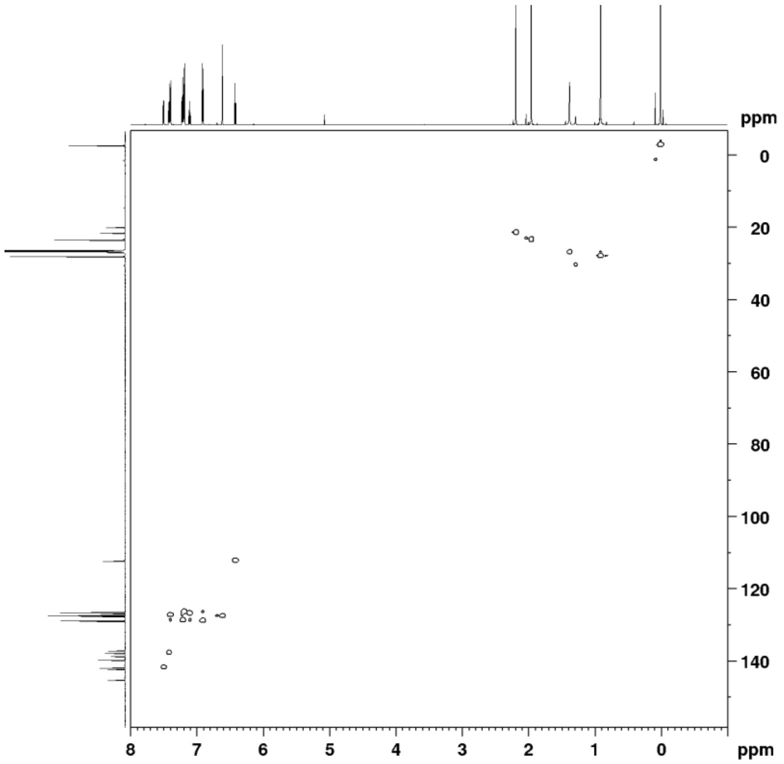

Figure S66. ^{1}H-^{13}C-HSQC-NMR spectrum of compound **8j** in C_6D_{12} measured at a 600 MHz spectrometer.

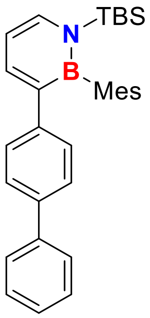

8j

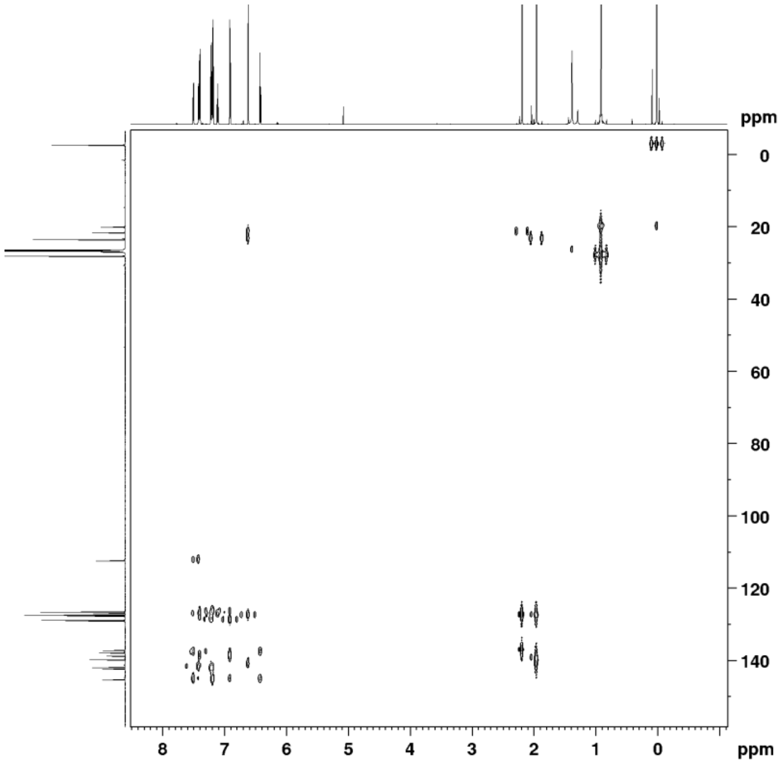

Figure S67. ^{1}H-^{13}C-HMBC-NMR spectrum of compound **8j** in C_6D_{12} measured at a 600 MHz spectrometer.

Upsacaling experiment: **1-(*tert*-Butyldimethylsilyl)-2-mesityl-3-(4-methoxyphenyl)-1,2-dihydro-1,2-azaborinine (8c)**

The synthesis was carried out according to a procedure by Yang *et al*. with modifications.[6]

3-Bromo-1-(*tert*-butyldimethylsilyl)-2-mesityl-1,2-dihydro-1,2-azaborinine (0.2977 g, 0.76 mmol, 1 eq), 4-methoxyphenylboronic acid (0.150 g, 1 mmol, 1.3 eq), dry Cs_2CO_3 (0.495 g, 1.5 mmol, 2 eq), $Pd_2(dba)_3$ (14 mg, 0.015 mmol, 2 mol%) and S-BIDIME (12.6 mg, 0.038 mmol, 6 mol%) were solved in dry, degassed toluene (12 mL) and degassed water (2.5 mL). The reaction mixture was allowed to stir for 16 h at 50 °C. Distilled water (10 mL) was added and the aqueous layer was extracted three times with n-hexane (10 mL). The combined organic layers were dried over $MgSO_4$. After removing of the solvent, the crude product was purified by column chromatography (silica, *n*-hexane/dichloromethane gradient). The product **8c** was obtained as colorless crystals (0.3062 g, 97%).

Synthesis of two and trifold brominated dihydroazaborinines 2-5

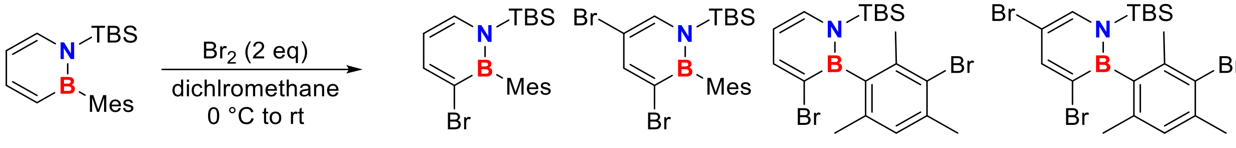

1-(*tert*-butyldimethylsilyl)-2-mesityl-1,2-dihydro-1,2-azaborinine (**1**) (1 g, 3.2 mmol, 1 eq) was solved in dichloromethane (10 mL) and cooled to 0 °C. A solution of bromine (0.18 mL, 7 mmol, 2.2 eq) in dichloromethane (10 mL) was added dropwise over 1 h. The reaction mixture was stirred for 30 minutes at 0 °C. The solution was allowed to reach room temperature and stirred for another 30 minutes, before a saturated solution of Na_2S0_3 (20 mL) was added. The aqueous layer was extracted three times with *n*-hexane (15 mL) and the combined organic layers were dried over $MgSO_4$. After removing of the solvent, the crude product was purified by column chromatography (silica, *n*-hexane/dichloromethane gradient). This yielded a pure fraction of dihydroazaborinine **3**, a mix of the dihydroazaborinines **2-3**, a mix of compounds **3** and **4** and a mixture of **3** and **5**. All mixtures were further purified *via* GPC size exclusion chromatography (*n*-hexane/dichloromethane 20/80). In this way, it was possible to obtain pure fractions of the different brominated dihydroazaborinines **3-5**. However, complete baseline separation could not be achieved for all compounds, which reduces the yield. Ultimately, the following yields were achieved: **2** (156.1 mg, 13 %), **3** (464.4 mg, 31%), **4** (431.5 mg,29%) and **5** (164.2 mg, 9%).

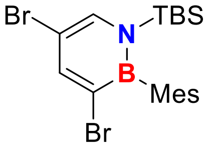

3

$C_{19}H_{28}BBr_2NSi$ (469.14 g/mol)

^{1}H-NMR (600 MHz, **CD_2Cl_2**): δ = 8.00 (d, $^3J_{HH}$ = 6.91 Hz, 1H, H-4), 7.61 (d, $^3J_{HH}$ = 6.91 Hz, 1H, H-6), 6.80 (s, 2H, H-14), 2.29 (s, 3H, H-16), 2.05 (s, 6H, H-13), 0.93 (s, 9H, H-10), 0.00 (s, 6H, H-8) ppm.

^{13}C-{^{1}H}-NMR (151 MHz, **CD_2Cl_2**): δ = 147.3 (C4), 139.4 (C12), 138.7 (C11), 138.5 (C6), 137.8 (C15), 132.7 (C3), 127.4 (C14), 104.7 (C5), 27.5 (C10), 22.9 (C13), 21.4 (C16), 19.5 (C9), -3.1 (C8) ppm.

^{11}B-{^{1}H}-NMR (192 MHz, **CD_2Cl_2**): δ = 39.0 ppm.

HR-MS (ESI): m/z calc. For [M+Na]+ 490.03430, found 490.03507.

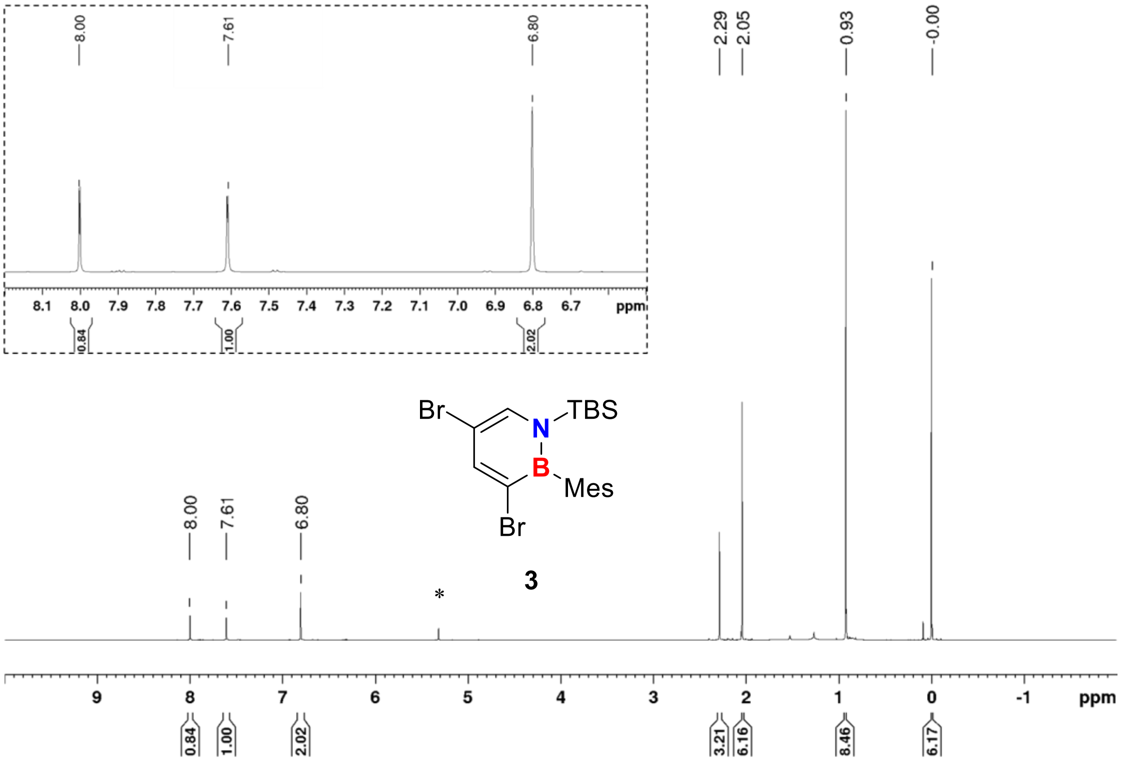

Figure S68. ^{1}H-NMR spectrum of compound **3** in CD_2Cl_2 measured at a 600 MHz spectrometer. The enlarged section shows the region between 6.5 and 8.2 ppm for a better visibility of the aromatic signals. The solvent signal is marked with an asterisk.

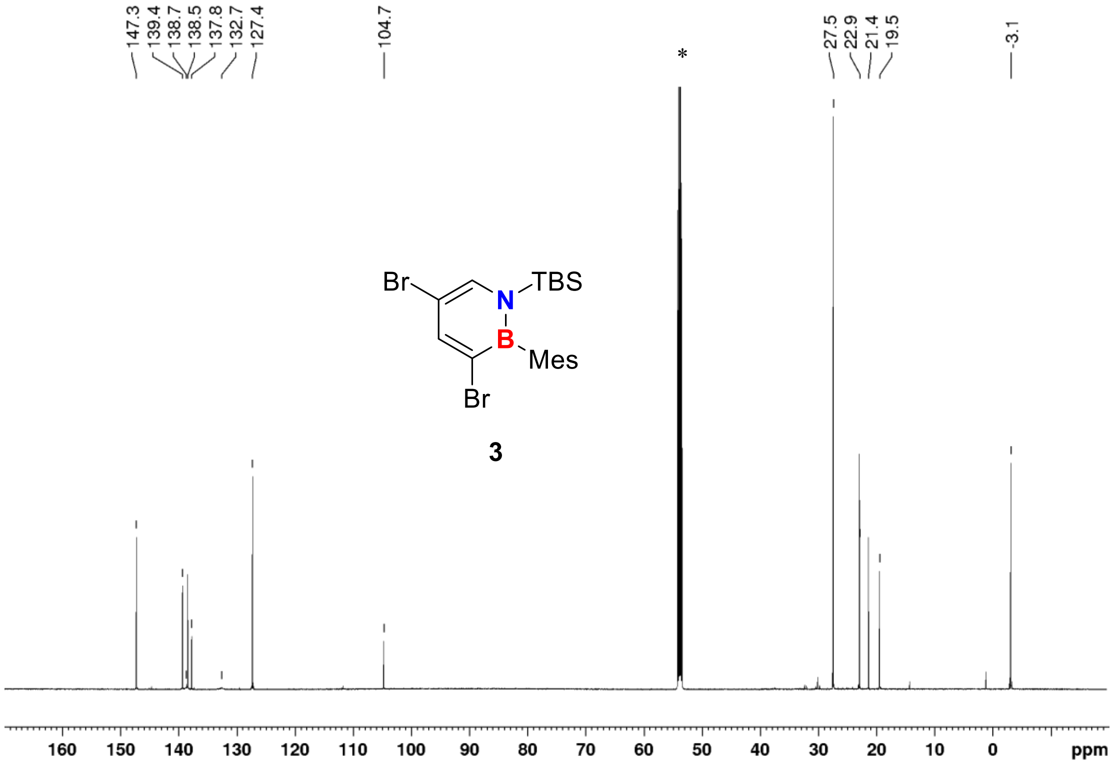

Figure S69. ^{13}C-{^{1}H}-NMR spectrum of compound **3** in CD_2Cl_2 measured at a 600 MHz spectrometer. The solvent signal is marked with an asterisk.

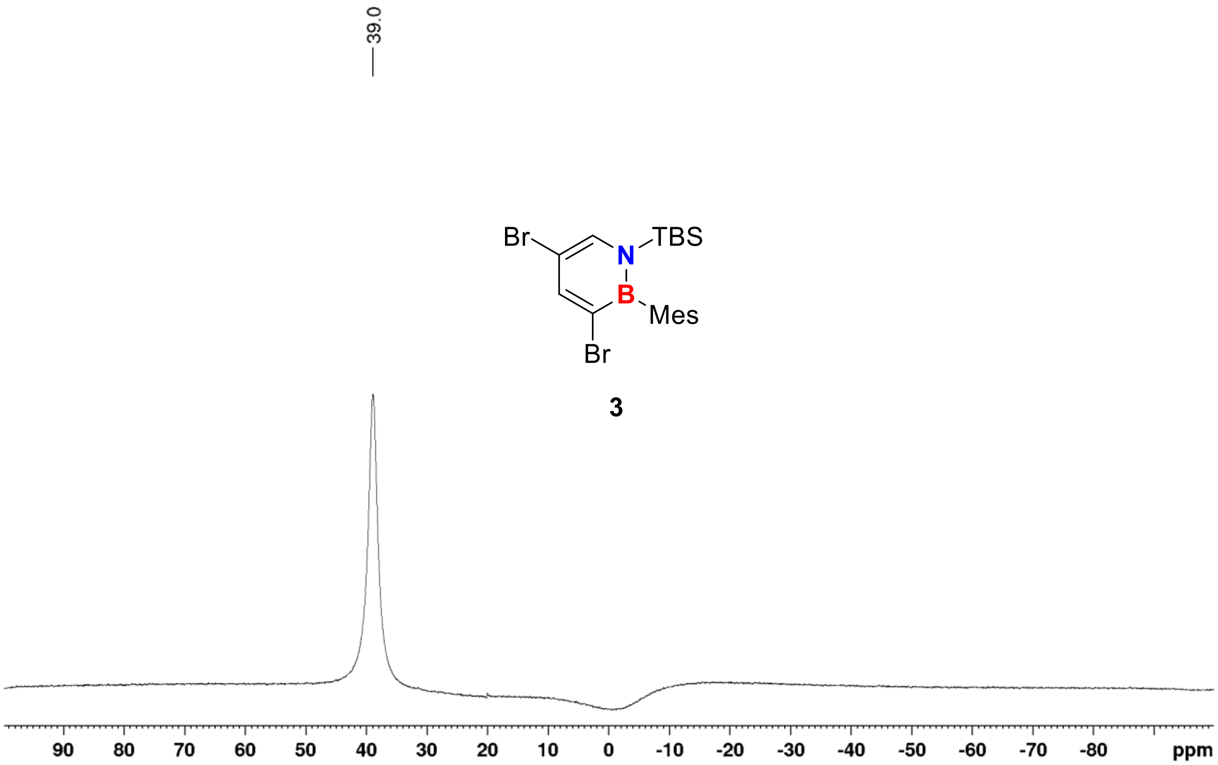

Figure S70. ^{11}B-{^{1}H}-NMR spectrum of compound **3** in CD_2Cl_2 measured at a 600 MHz spectrometer.

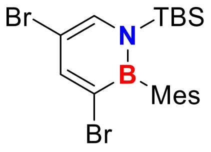

3

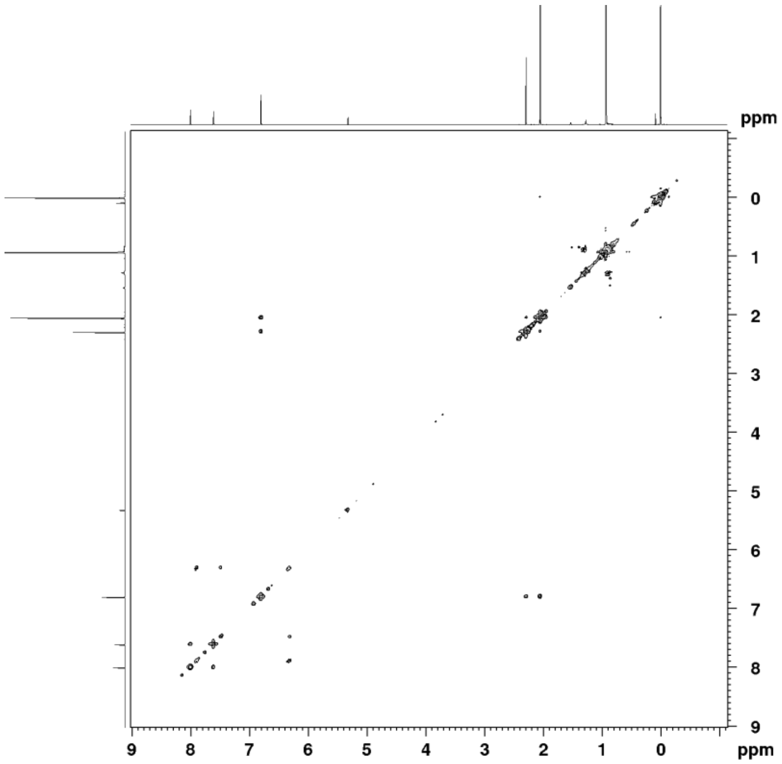

Figure S71. ^{1}H-^{1}H-COSY-NMR spectrum of compound **3** in CD_2Cl_2 measured at a 600 MHz spectrometer.

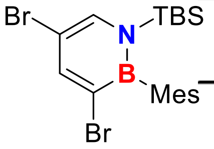

3

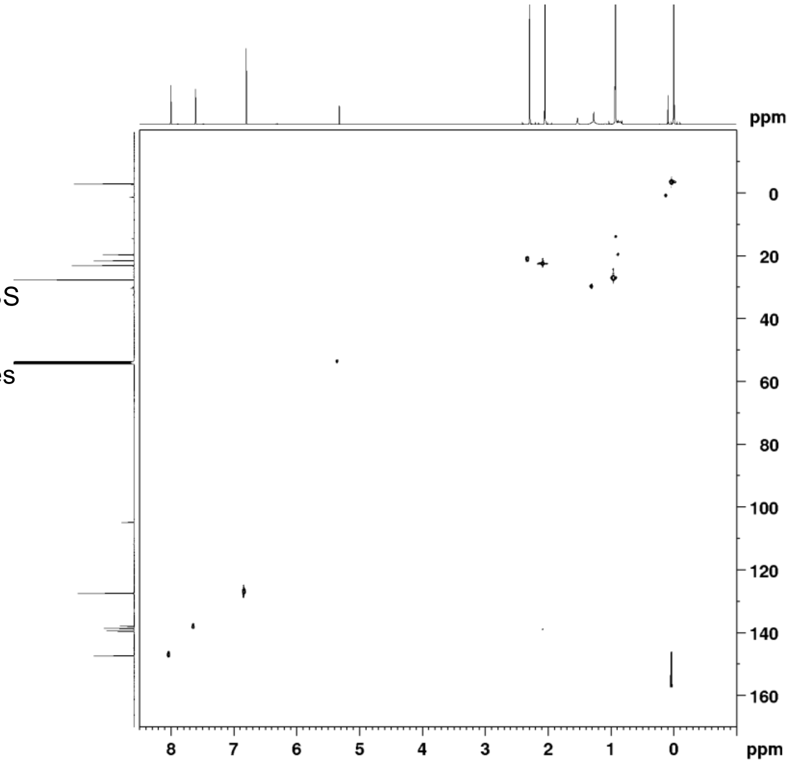

Figure S72. ^{1}H-^{13}C-HSQC-NMR spectrum of compound **3** in CD_2Cl_2 measured at a 600 MHz spectrometer.

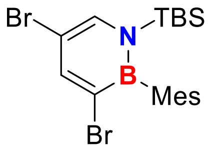

3

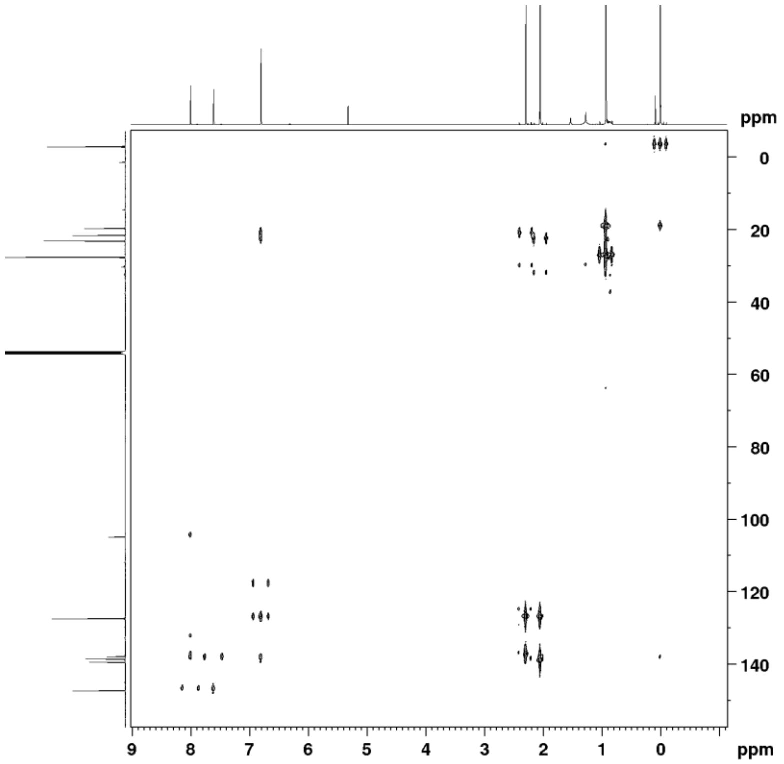

Figure S73. ^{1}H-^{13}C-HMBC-NMR spectrum of compound **3** in CD_2Cl_2 measured at a 600 MHz spectrometer.

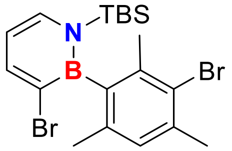

4

$C_{19}H_{28}BBr_2NSi$ (469.14 g/mol)

^{1}H-NMR (600 MHz, **CD_2Cl_2**): δ = 7.91 (dd, $^3J_{HH}$ = 7.23 Hz, $^4J_{HH}$ = 0.88 Hz, 1H, H-4), 7.48 (dd, $^3J_{HH}$ = 6.81 Hz, $^4J_{HH}$ = 0.88 Hz, 1H, H-6), 6.91 (s, 2H, H-17), 6.34 (ps.t, 1H, H-5), 2.40 (s, 3H, H-16), 2.20 (s, 3H, H-13), 2.01 (s, 3H, H-19), 0.91 (s, 9H, H-10), 0.03 (s, 3H, H-8), -0.06 (s, 3H, H-8) ppm.

^{13}C-{^{1}H}-NMR (151 MHz, **CD_2Cl_2**): δ = 145.1 (C4), 142.7 (C11), 138.9 (C12), 138.6 (C6), 138.3 (C18), 137.6 (C15), 131.8 (C3), 129.6 (C17), 125.3 (C14), 112.1 (C5), 27.5 (C10), 25.1 (C13), 24.1 (C16), 22.4 (C19), 19.5 (C9), -2.9 (C8) ppm.

^{11}B-{^{1}H}-NMR (192 MHz, **CD_2Cl_2**): δ = 38.6 ppm.

HR-MS (ESI): m/z calc. For [M+Na]+ 490.03430, found 490.03458.

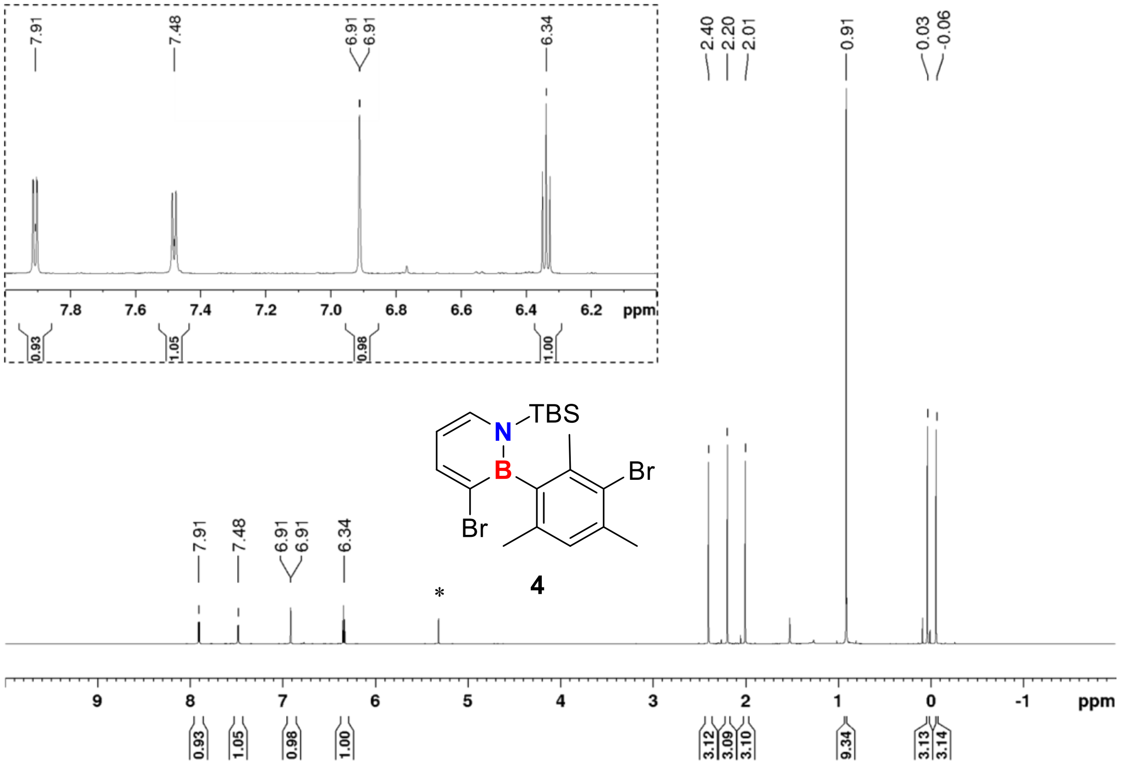

Figure S74. ^{1}H-NMR spectrum of compound **4** in CD_2Cl_2 measured at a 600 MHz spectrometer. The enlarged section shows the region between 5 and 8 ppm for a better visibility of the aromatic signals. The solvent signal is marked with an asterisk.

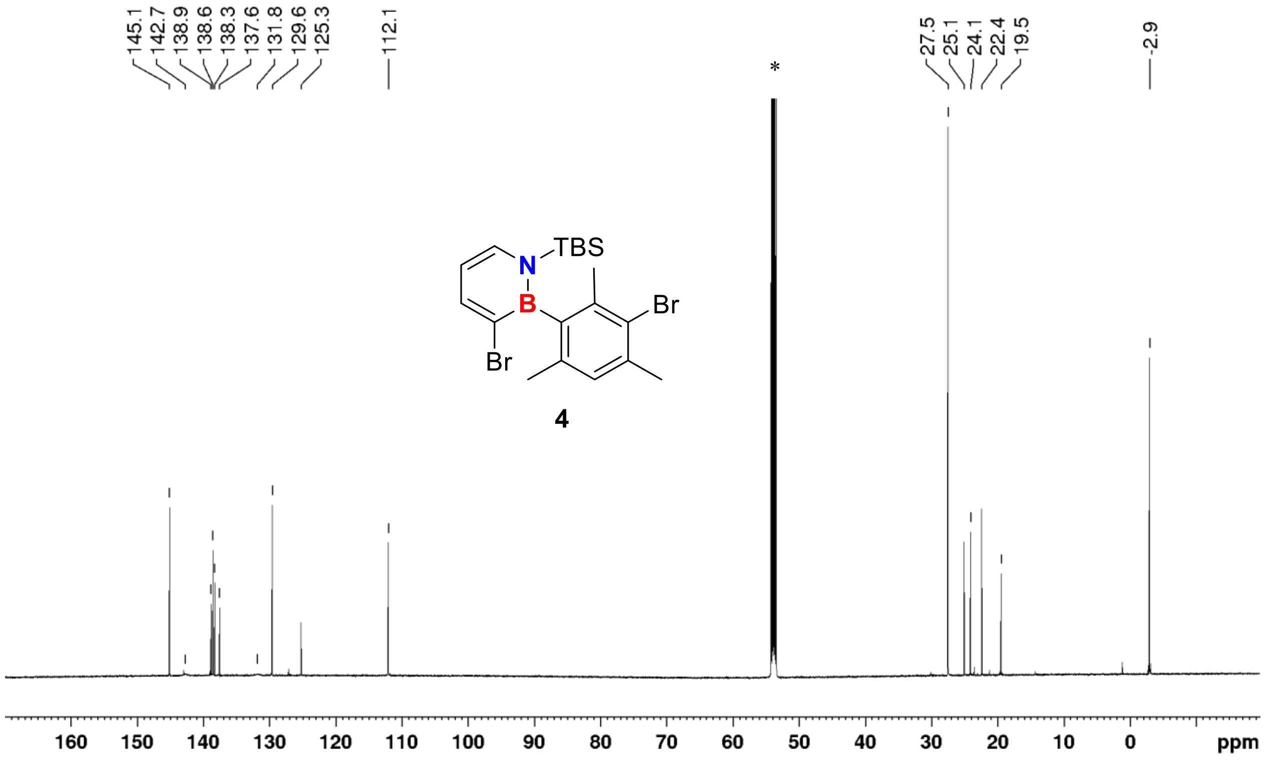

Figure S75. ^{13}C-{^{1}H}-NMR spectrum of compound **4** in CD_2Cl_2 measured at a 600 MHz spectrometer. The solvent signal is marked with an asterisk.

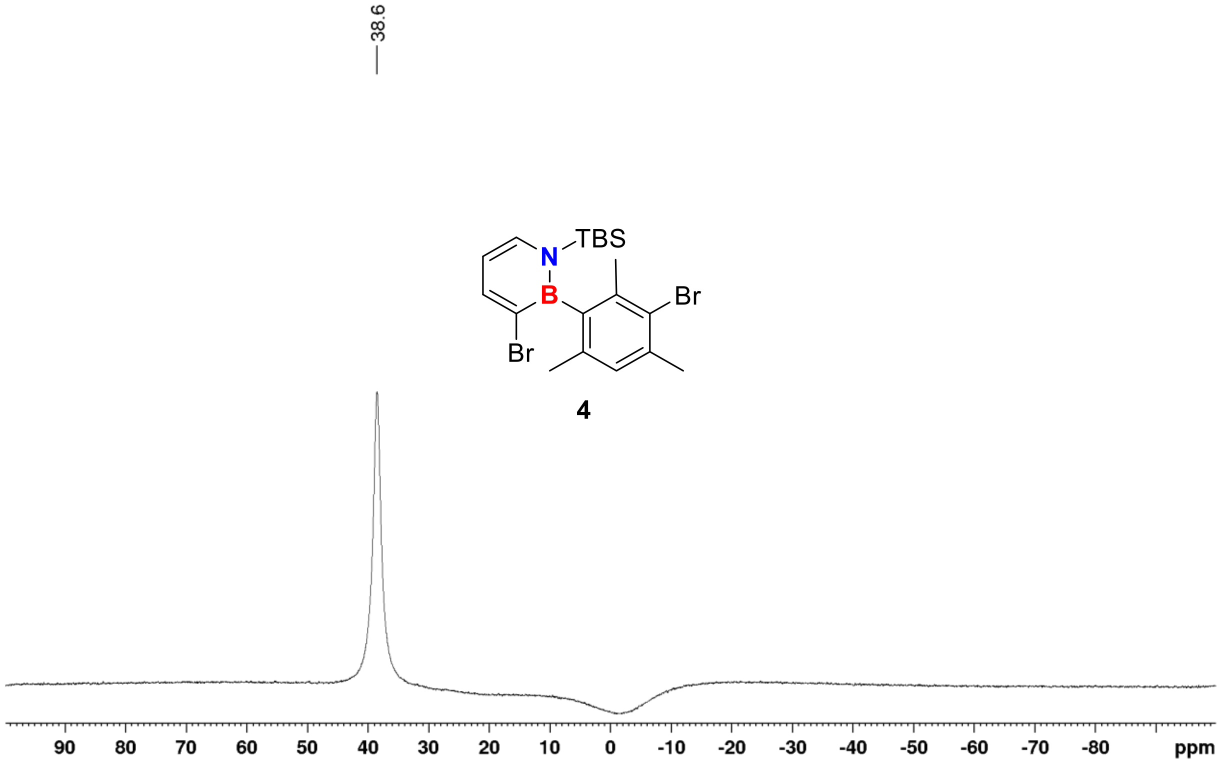

Figure S76. ^{11}B-{^{1}H}-NMR spectrum of compound **4** in CD_2Cl_2 measured at a 600 MHz spectrometer.

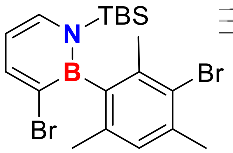

4

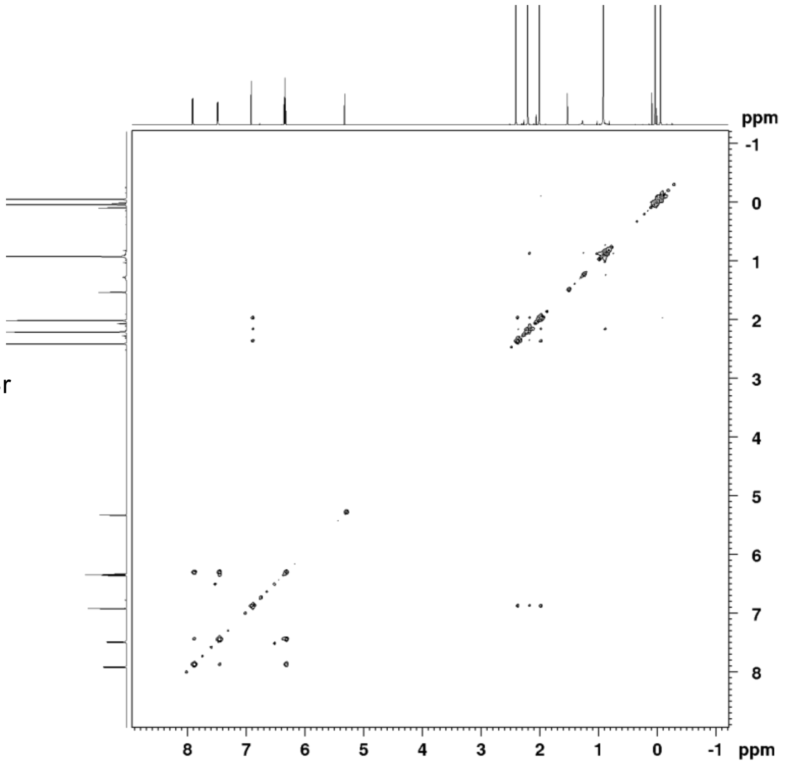

Figure S77. ^{1}H-^{1}H-COSY-NMR spectrum of compound **4** in CD_2Cl_2 measured at a 600 MHz spectrometer.

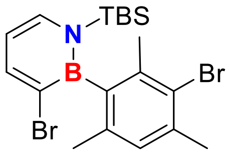

4

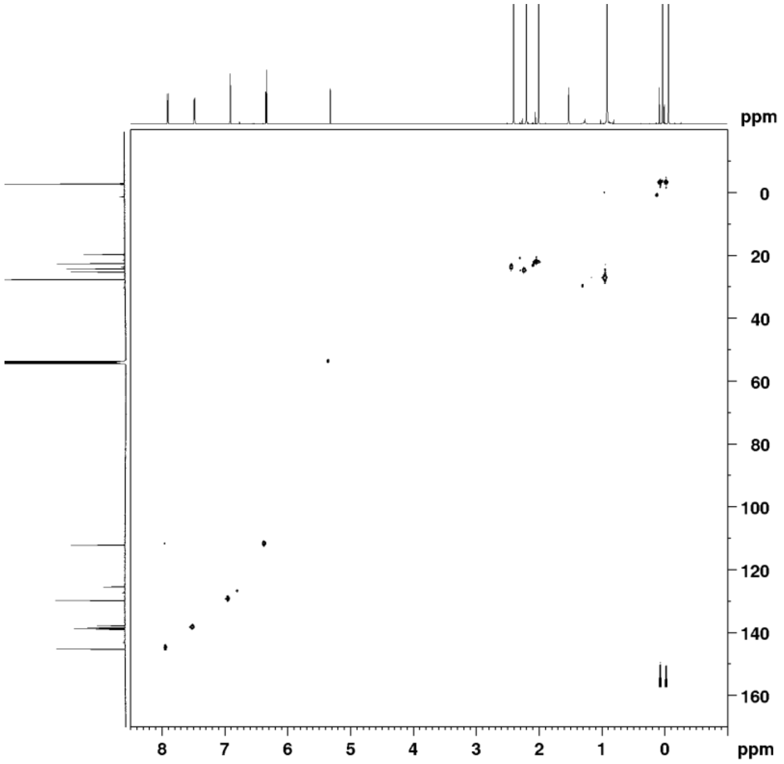

Figure S78. ^{1}H-^{13}C-HSQC-NMR spectrum of compound **4** in CD_2Cl_2 measured at a 600 MHz spectrometer.

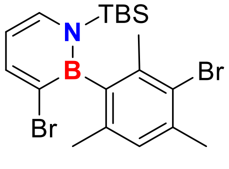

4

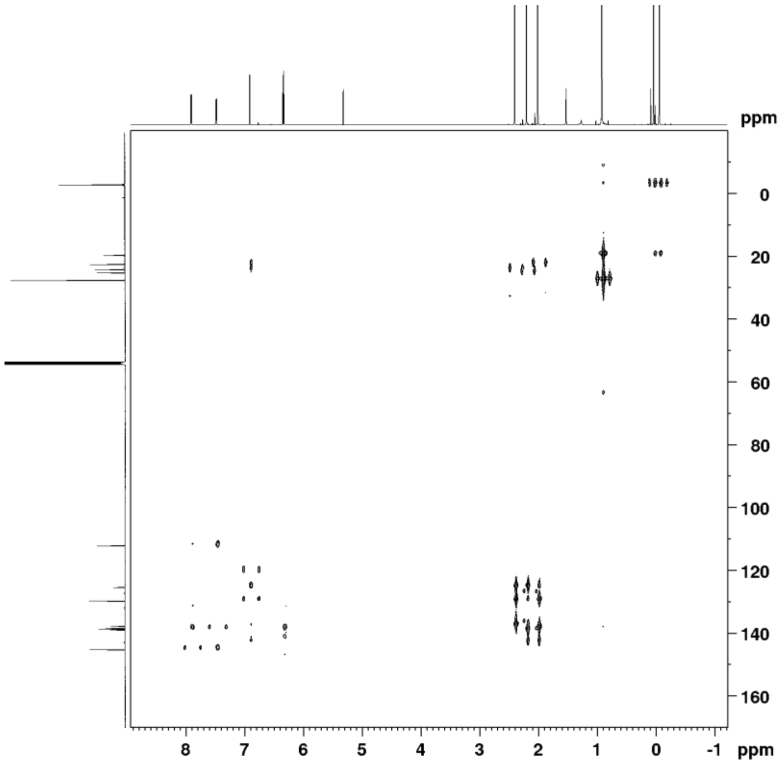

Figure S79. ^{1}H-^{13}C-HMBC-NMR spectrum of compound **4** in CD_2Cl_2 measured at a 600 MHz spectrometer.

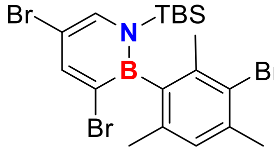

5

$C_{19}H_{28b}Br_3NSi$ (548.04 g/mol)

^{1}H-NMR (600 MHz, **CD_2Cl_2**): δ = 8.02 (d, $^4J_{HH}$ = 1.66 Hz, 1H, H-4), 7.61 (d, $^4J_{HH}$ = 1.66 Hz, 1H, H-6), 6.92 (s, 1H, H-17), 2.40 (s, 3H, H-16), 2.19 (s, 3H, H-13), 2.00 (s, 3H, H-19), 0.92 (s, 9H, H-10), 0.05 (s, 3H, H-8), -0.05 (s, 3H, H-8) ppm.

^{13}C-{^{1}H}-NMR (151 MHz, **CD_2Cl_2**): δ = 147.7 (C4), 141.5 (C11), 138.9 (C12), 138.4 (C6), 138.3 (C17), 138.0 (C15), 132.2 (C3), 129.7 (C17), 125.4 (C14), 105.0 (C5), 27.4 (C10), 25.1 (C13), 24.1 (C16), 22.4 (C19), 19.5 (C9), -3.0 (C8) ppm.

^{11}B-{^{1}H}-NMR (192 MHz, **CD_2Cl_2**): δ = 38.2 ppm.

HR-MS (ASAP): m/z calc. For [M]·+ 547.9599, found 547.9613.

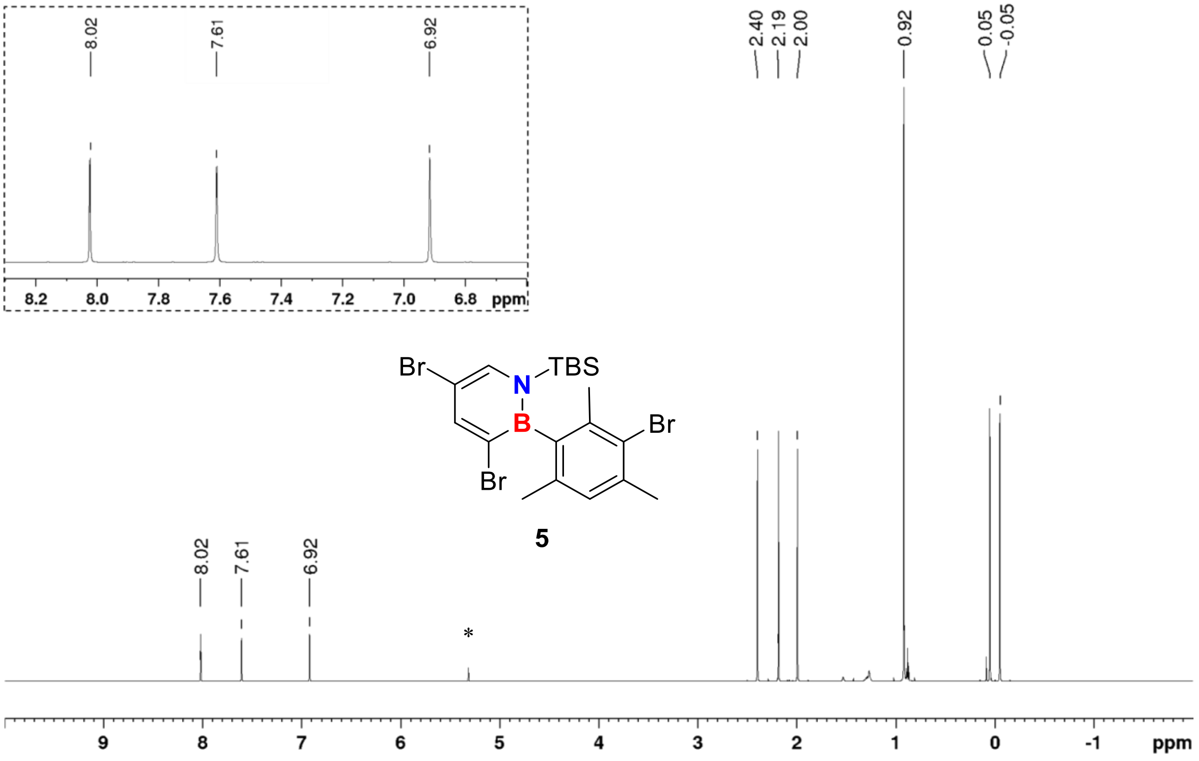

Figure S80. ^{1}H-NMR spectrum of compound **5** in CD_2Cl_2 measured at a 600 MHz spectrometer. The enlarged section shows the region between 6.5 and 8.2 ppm for a better visibility of the aromatic signals. The solvent signal is marked with an asterisk.

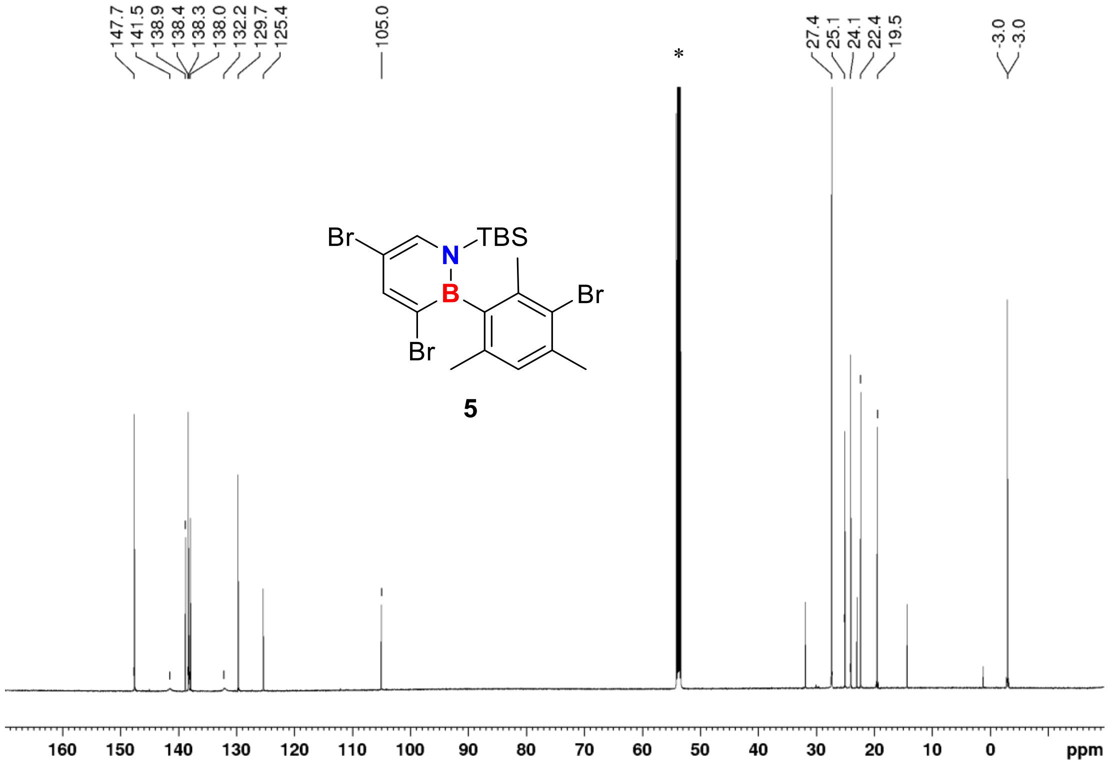

Figure S81. ^{13}C-{^{1}H}-NMR spectrum of compound **5** in CD_2Cl_2 measured at a 600 MHz spectrometer. The solvent signal is marked with an asterisk.

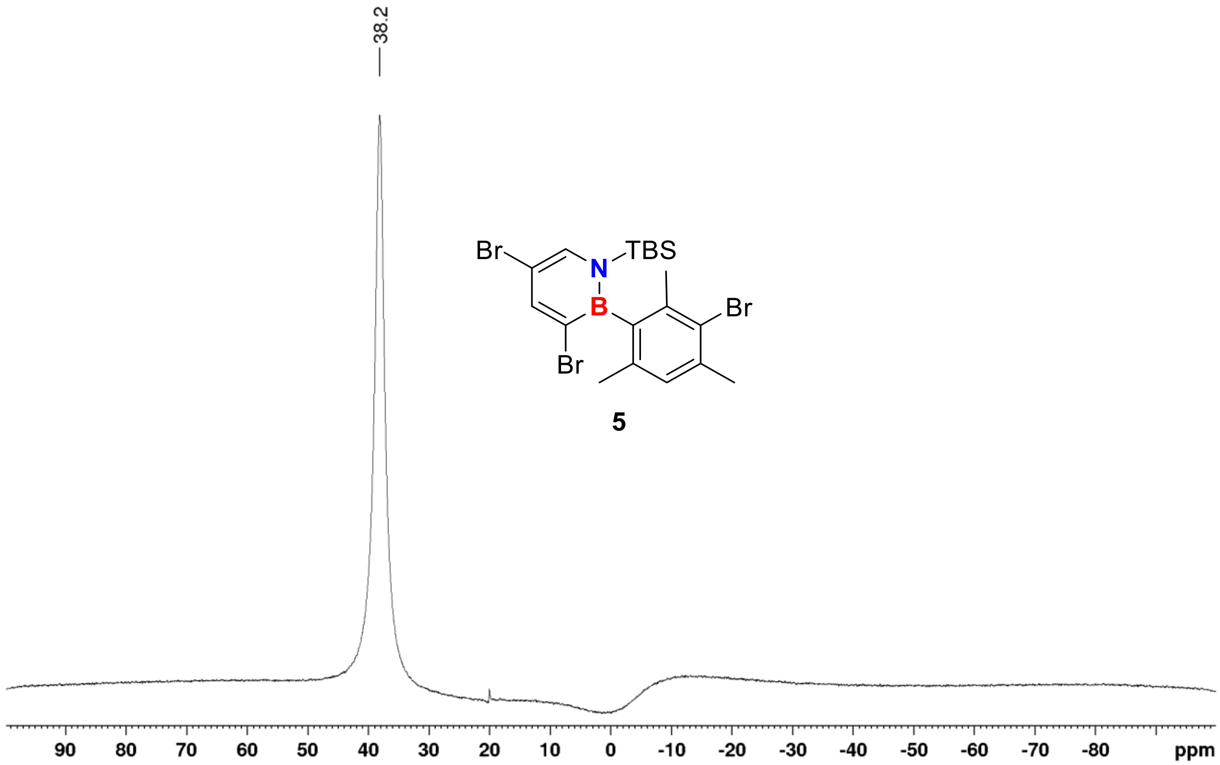

Figure S82. ^{11}B-{^{1}H}-NMR spectrum of compound **5** in CD_2Cl_2 measured at a 600 MHz spectrometer.

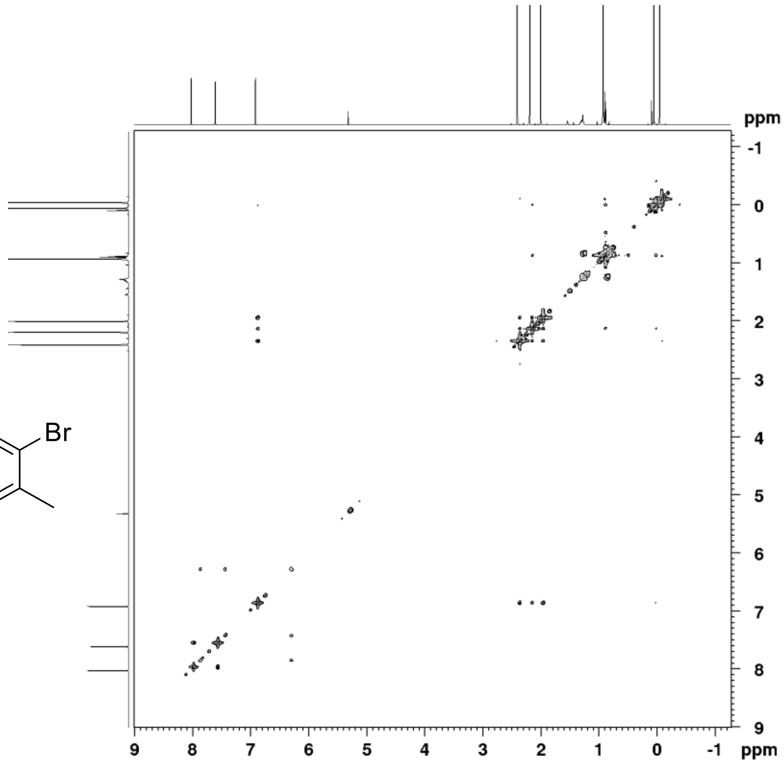

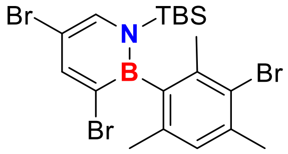

5

Figure S83. ^{1}H-^{1}H-COSY-NMR spectrum of compound **5** in CD_2Cl_2 measured at a 600 MHz spectrometer.

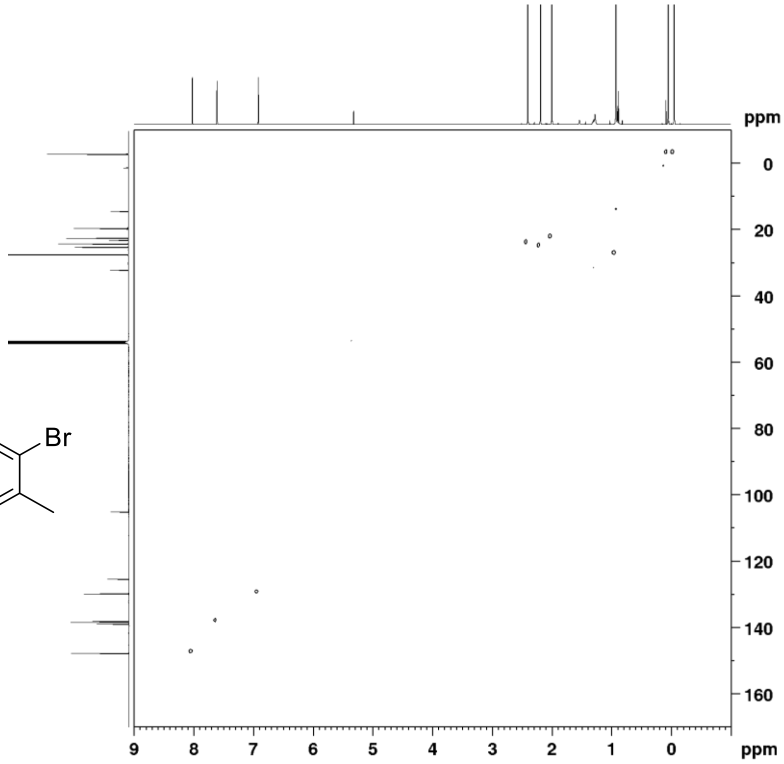

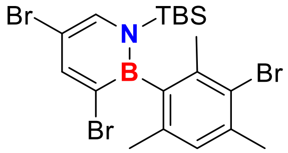

5

Figure S84. ^{1}H-^{13}C-HSQC-NMR spectrum of compound **5** in CD_2Cl_2 measured at a 600 MHz spectrometer.

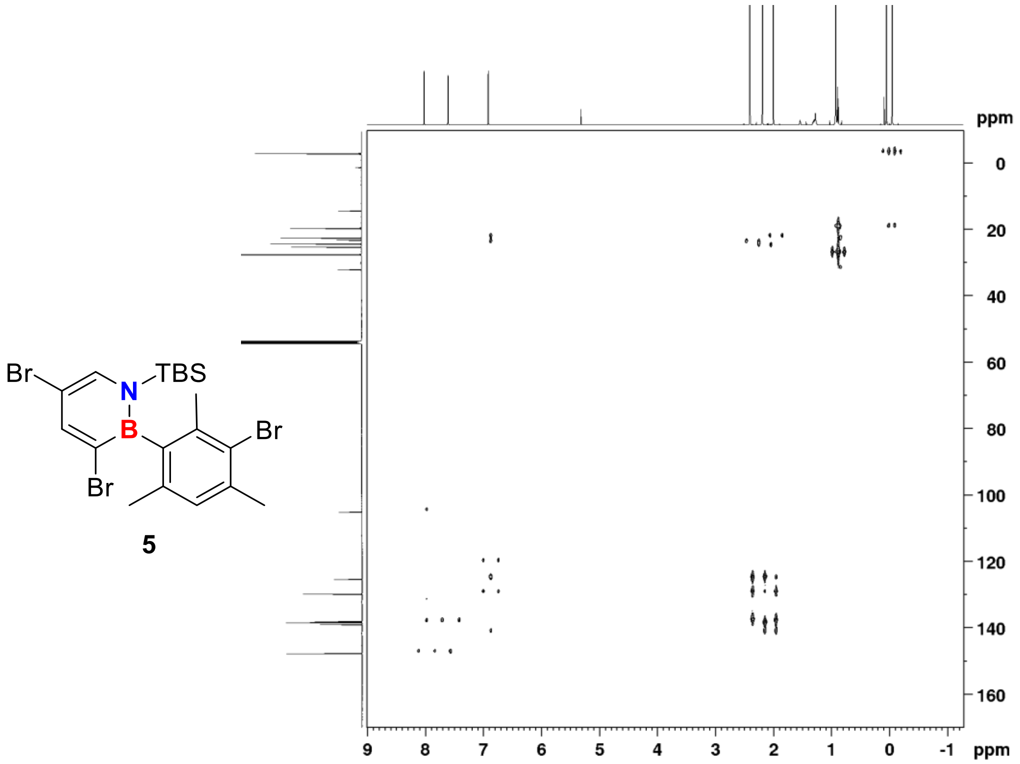

Figure S85. ^{1}H-^{13}C-HMBC-NMR spectrum of compound **5** in CD_2Cl_2 measured at a 600 MHz spectrometer.

Oxidation of 1-(*tert*-Butyldimethylsilyl)-2-mesityl-3-(4-methylthiophenyl)-1,2-dihydro-1,2-azaborinine (8d) to 1-(*tert*-Butyldimethylsilyl)-2-mesityl-3-(4-methlsulfoxidphenyl)-1,2-dihydro-1,2-azaborinine (8k)

The synthesis was carried out according to a procedure by Wood and Brown *et al*. with modifications.[13, 14]

Azaborinine 8d (9.7 mg, 22 µmol, 1 eq) is placed in a 3 mL screw cap vial and solved in a mixture of *n*-hexane (10 µL) and tetrahydrofuran (90 µL). Over the course of 5 minutes an aqueous solution of sodium hypochlorite (5% solution in water, 32 µL, 22 µmol, 1 eq) is added dropwise. The resulting solution was stirred for two hours at room temperature. The whole reaction mixture is transferred into a silica column without any interim aqueous workup (*n*-hex/ethylacetate gradient). The product was obtained as a colorless solid (7.4 mg, 75 %).

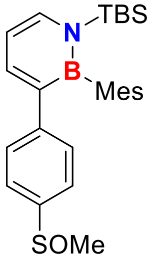

8k

$C_{26}H_{36}BNO_2SSi$ (449.54 g/mol)

^{1}H-NMR (400 MHz, **CD_2Cl_2**): δ = 7.55 (ps. t, 2H, H-4/H-6), 7.31 (dm, $^3J_{HH}$ = 8.48 Hz, 2H, H-19), 7.08 (dm, $^3J_{HH}$ = 8.48 Hz, 2H, H-18), 6.68 (s, 2H, H-14), 6.55 (ps. t, 1H, H-5), 0.61 (s, 3H, H-22), 2.23 (s, 3H, H-16), 1.96 (s, 3H, H-13), 1.94 (s, 3H, H-13), 0.91 (s, 9H, H-10), 0.02 (s, 3H, H-8), 0.01 (s, 6H, H-8) ppm.

^{13}C-{^{1}H}-NMR (101 MHz, **CD_2Cl_2**): δ = 149.5 (C17), 142.7 (C4), 142.6 (C20), 139.5 (C12), 139.4 (C6), 137.2 (C15), 129.2 (C18), 127.4 (C11), 127.3 (C14), 127.3 (C3), 123.0 (C19), 111.8 (C5), 44.2 (C22), 27.7 (C10), 23.3 (C13), 23.2 (C13), 21.3 (C16), 19.6 (C9), -2.9 (C8) ppm.

^{11}B-{^{1}H}-NMR (128 MHz, **CD_2Cl_2**): δ = 39.8 ppm.

HR-MS (ESI): m/z calc. For [M+H]+ 450.24527, found 450.24612.

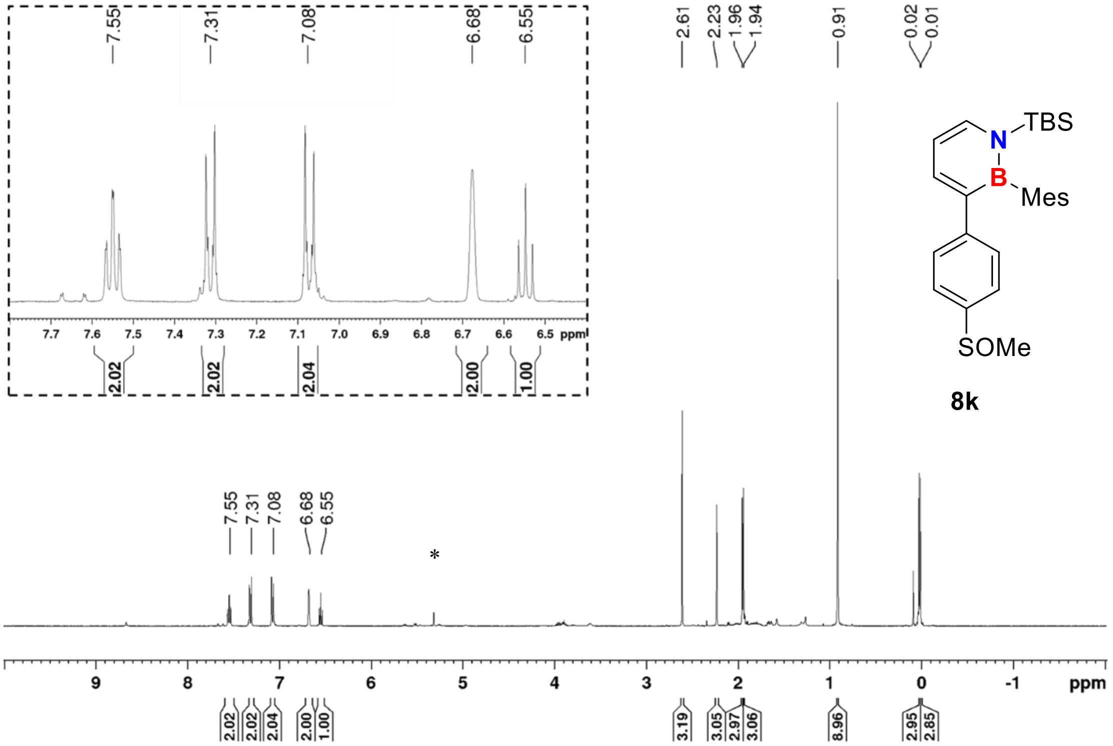

Figure S86. ^{1}H-NMR spectrum of compound **8k** in CD_2Cl_2 measured at a 400 MHz spectrometer. The enlarged section shows the region between 6.4 and 7.8 ppm for a better visibility of the aromatic signals. The solvent signal is marked with an asterisk.

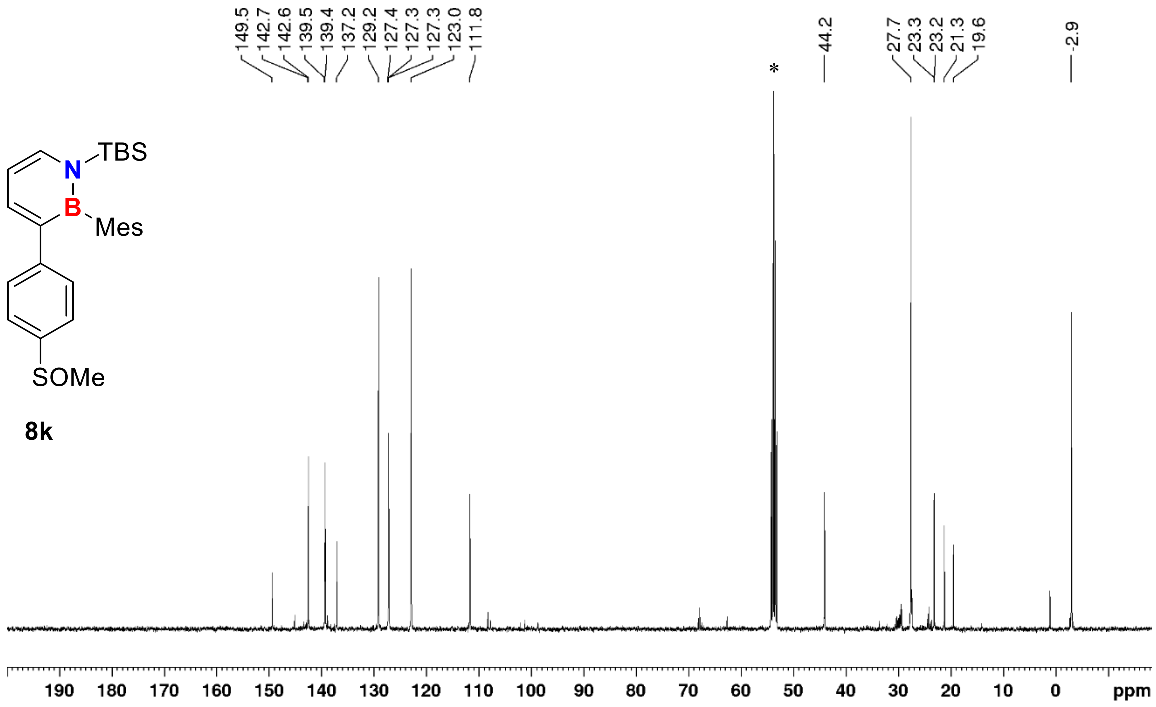

Figure S87. ^{13}C-{^{1}H}-NMR spectrum of compound **8k** in CD_2Cl_2 measured at a 400 MHz spectrometer. The solvent signal is marked with an asterisk.

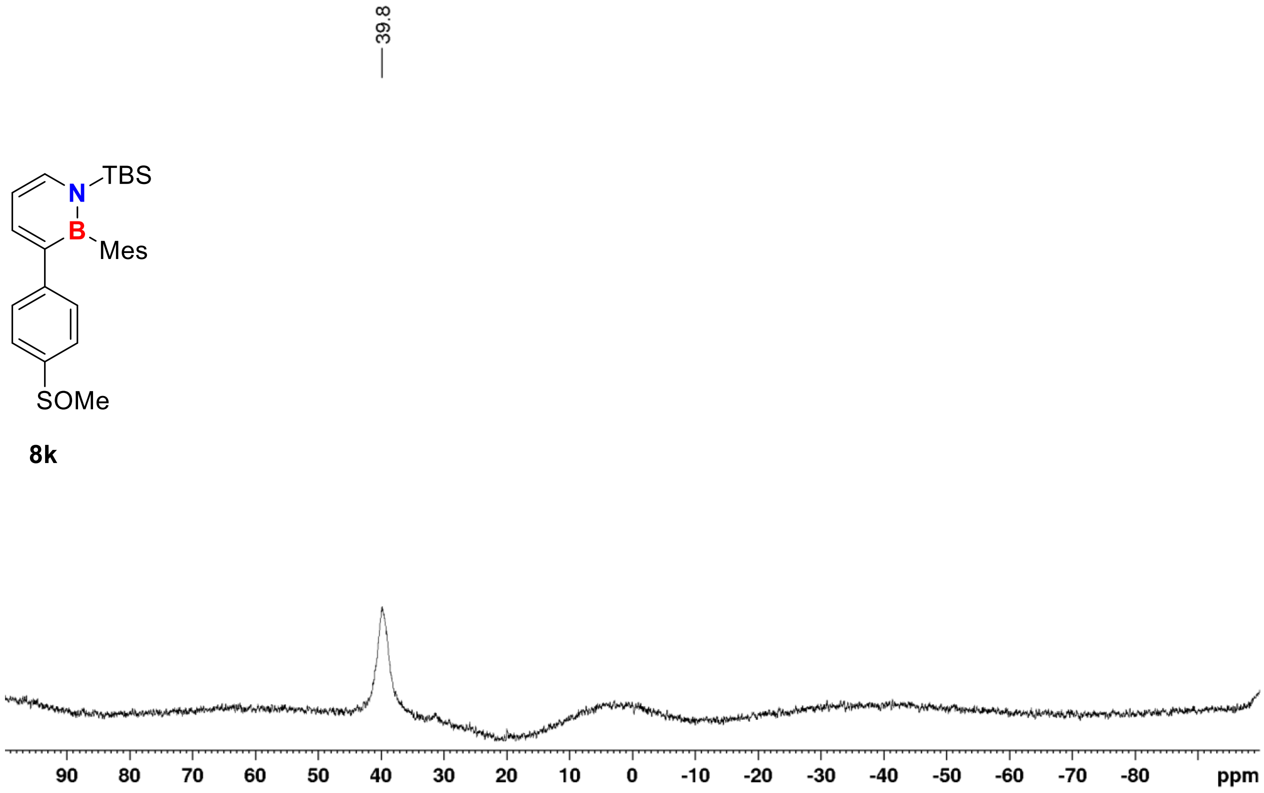

Figure S88. ^{11}B-{^{1}H}-NMR spectrum of compound **8k** in CD_2Cl_2 measured at a 400 MHz spectrometer.

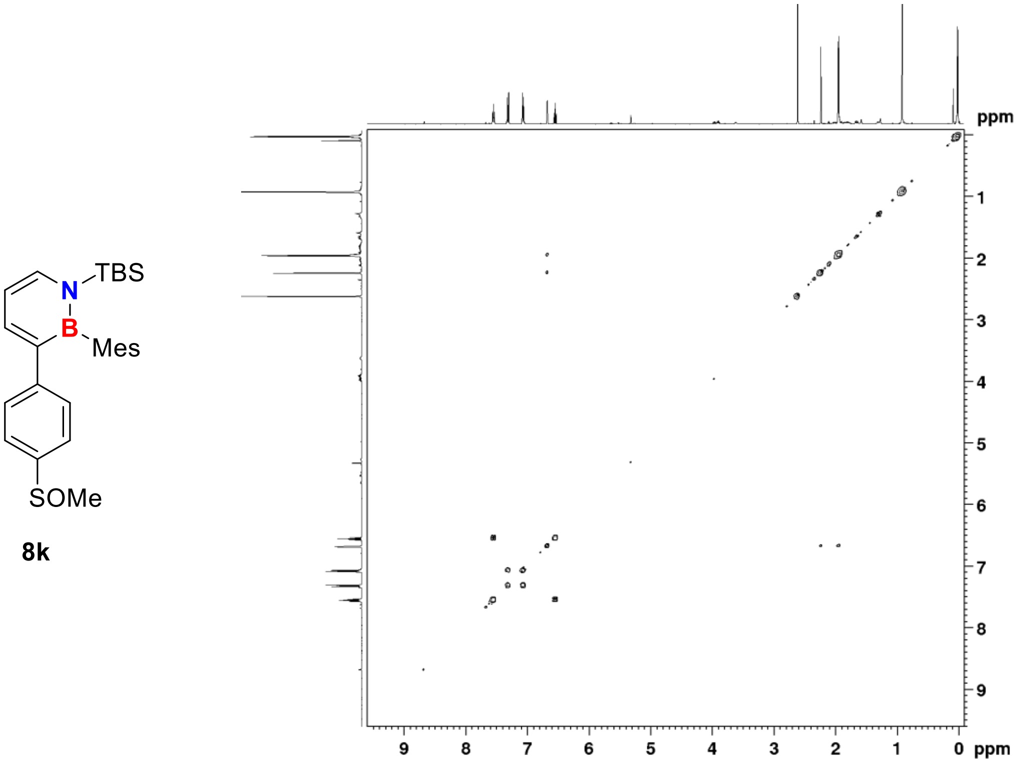

Figure S89. ^{1}H-^{1}H-COSY-NMR spectrum of compound **8k** in CD_2Cl_2 measured at a 400 MHz spectrometer.

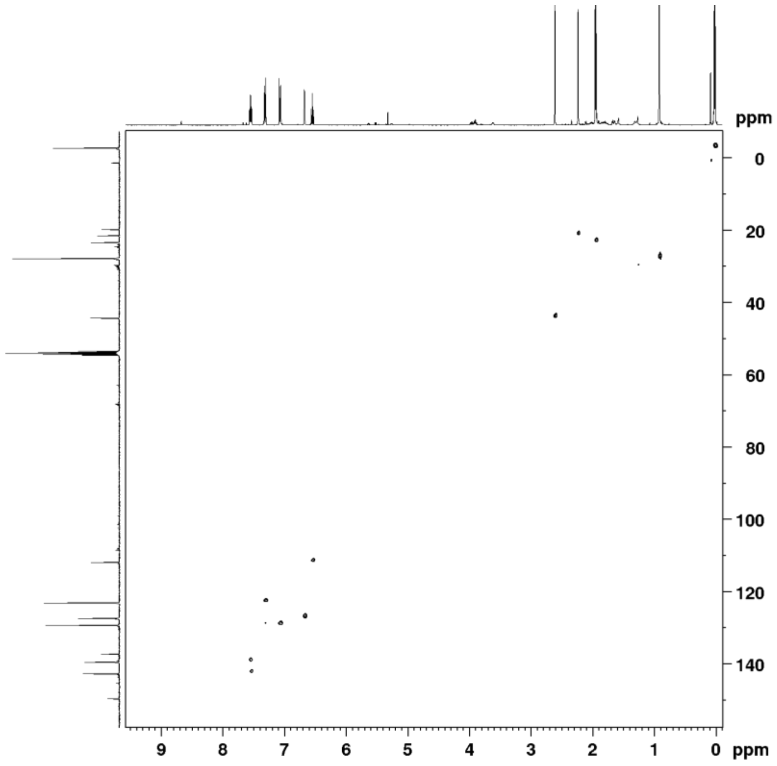

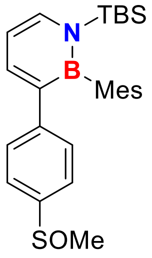

8k

Figure S90. ^{1}H-^{13}C-HSQC-NMR spectrum of compound **8k** in CD_2Cl_2 measured at a 400 MHz spectrometer.

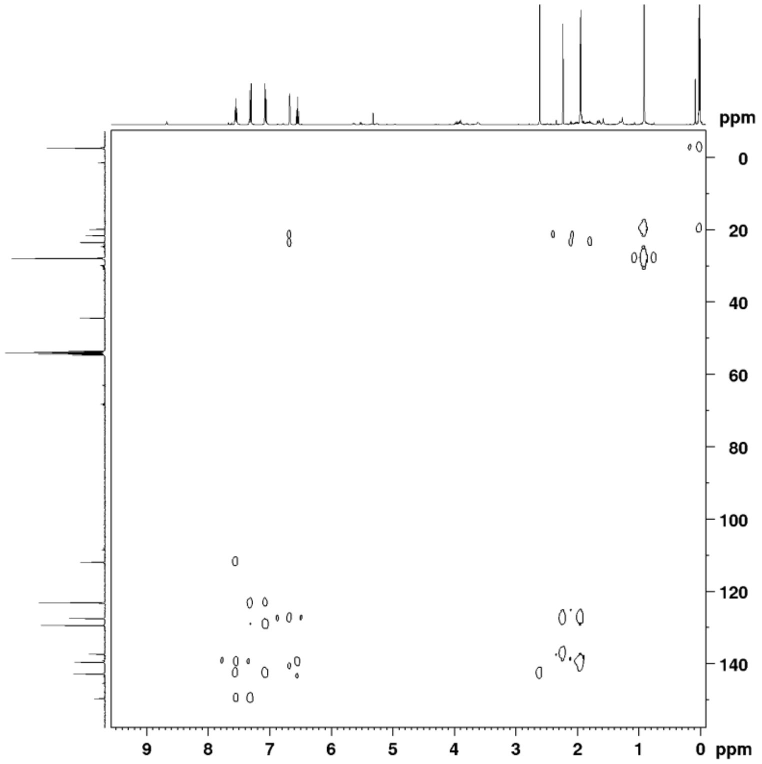

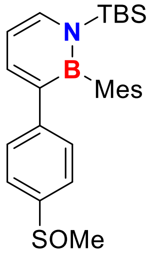

8k

Figure S91. ^{1}H-^{13}C-HMBC-NMR spectrum of compound **8k** in CD_2Cl_2 measured at a 400 MHz spectrometer.

Synthesis of pentamethylphenyl lithium (14)

Pentamethylphenyl bromide (5 g, 22 mmol, 1 eq) was solved in dry *n*-hexane (25 ml) and dry diethylether (10 mL). The mixture was cooled in a ice-salt bath and n-BuLi (1.6 in *n*-hexane, 15.1 mL, 24 mmol, 1.1 eq) was added dropwise under stirring over 45 minutes. The mixture was allowed to stir for 12 to 16 h at room temperature. The resulting precipitate is filtered off using an inert gas frit. The filter cake is washed twice with *n*-pentane (10 mL) and dried under vacuum. Compound 10 is obtained as a beige-white solid (2.85 g, 84%).

$C_{11}H_{15}Li$ (154.18 g/mol)

^{1}H-NMR (400 MHz, **CD_2Cl_2**): δ = 2.21 (s, 6H, H-3), 2.20 (s, 3H, H-7), 2.16 (s, 6H, H-5) ppm.

^{13}C-{^{1}H}-NMR (101 MHz, **CD_2Cl_2**): δ = 134.6 (C1), 132.8 (C2), 132.1 (C6), 128.78 (C4), 20.2 (C3), 15.9 (C7), 15.6 (C5) ppm.

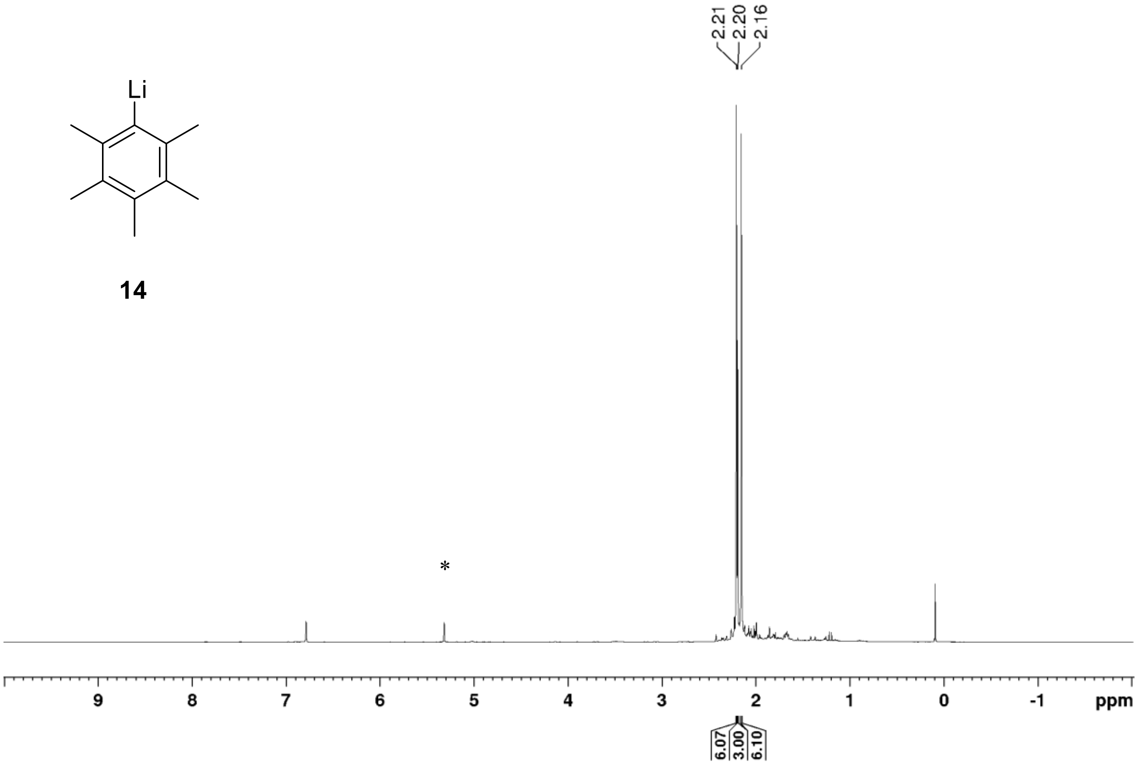

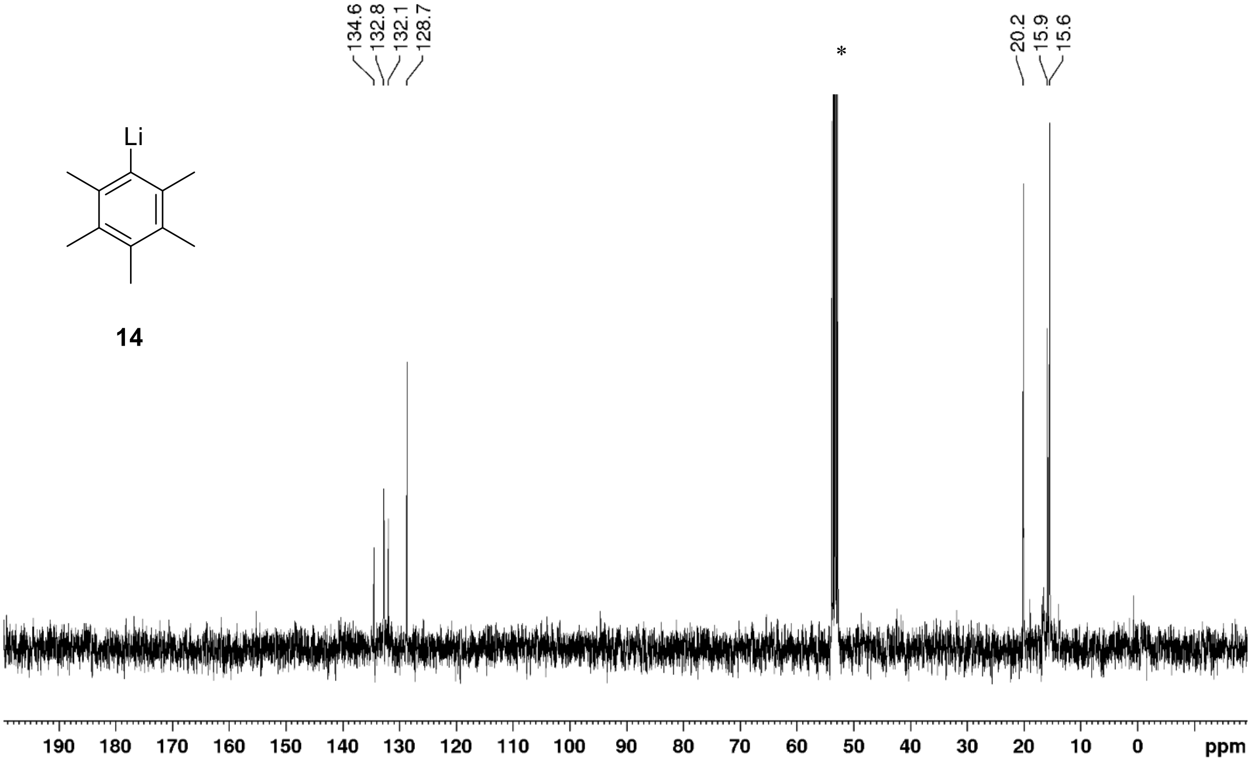
134.6
132.8
132.1
128.7
*
20.2
15.9
15.6
Li
14
190 180 170 160 150 140 130 120 110 100 90 80 70 60 50 40 30 20 10 0 ppm

Synthesis of 1-(*tert*-butyldimethylsilyl)-2-pentamethylphenyl-1,2-dihydro-1,2-azaborinine (11)

The synthesis was carried out according to a procedure by Edel *et al*. with modifications.[15]

1-(*tert*-butyldimethylsilyl)-2-chloro-1,2-dihydr-1,2-azaborinine (0.5 g, 2.2 mmol, 1 eq) is solved in benzene (15 mL). Pentamethylphenyllithium (0.68 g, 4.4 mmol, 2 eq) is added portion wise over 30 minutes to the stirred solution. The resulting suspension is stirred for 12 h at room temperature. After dilution with *n*-hexane, distilled water was added and the aqueous phase was extracted three times with *n*-hexane (15 mL). The organic phase was washed with brine (15 mL) dried over $MgSO_4$, concentrated *in vacuo* and the crude product was purified by column chromatography (silica, *n*-hexane/dichloromethane gradient) to afford **10** as a colorless solid (0.57 g, 76%).

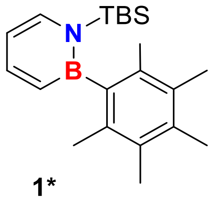

1*

$C_{21}H_{34}BNSi$ (339.41 g/mol)

^{1}H-NMR (400 MHz, **C_6D_6**): δ = 7.57 (ddd, $^3J_{HH}$ = 11.14 Hz, $^3J_{HH}$ = 6.22 Hz, $^4J_{HH}$ = 1.04 Hz, 1H, H-4), 7.36 (d, $^3J_{HH}$ = 6.48 Hz, 1H, H-6), 6.95 (dd, $^3J_{HH}$ = 11.14 Hz, $^3J_{HH}$ = 0.84 Hz, 1H, H-3), 6.30 (ps. t, 1H, H-5), 2.21 (s, 6H, H-13), 2.18 (s, 3H, H-17), 2.14 (s, 6H, H-15), 0.83 (s, 9H, H-10), -0,04 (s, 6H, H-8) ppm.

^{13}C-{^{1}H}-NMR (101 MHz, **C_6D_6**): δ = 143.3 (C11), 142.8 (C4), 138.2 (C6), 134.2 (C12), 133.5 (C3), 133.4 (C16), 131.6 (C14), 111.7 (C5), 27.6 (C10), 22.5 (C13), 19.3 (C9), 16.7 (C17), 16.2 (C15), -2.9 (C8) ppm.

^{11}B-{^{1}H}-NMR (128 MHz, **C_6D_6**): δ = 41.0 ppm.

HR-MS (APCI): m/z calc. For [M+H]+ 340.26305, found 340.26346.

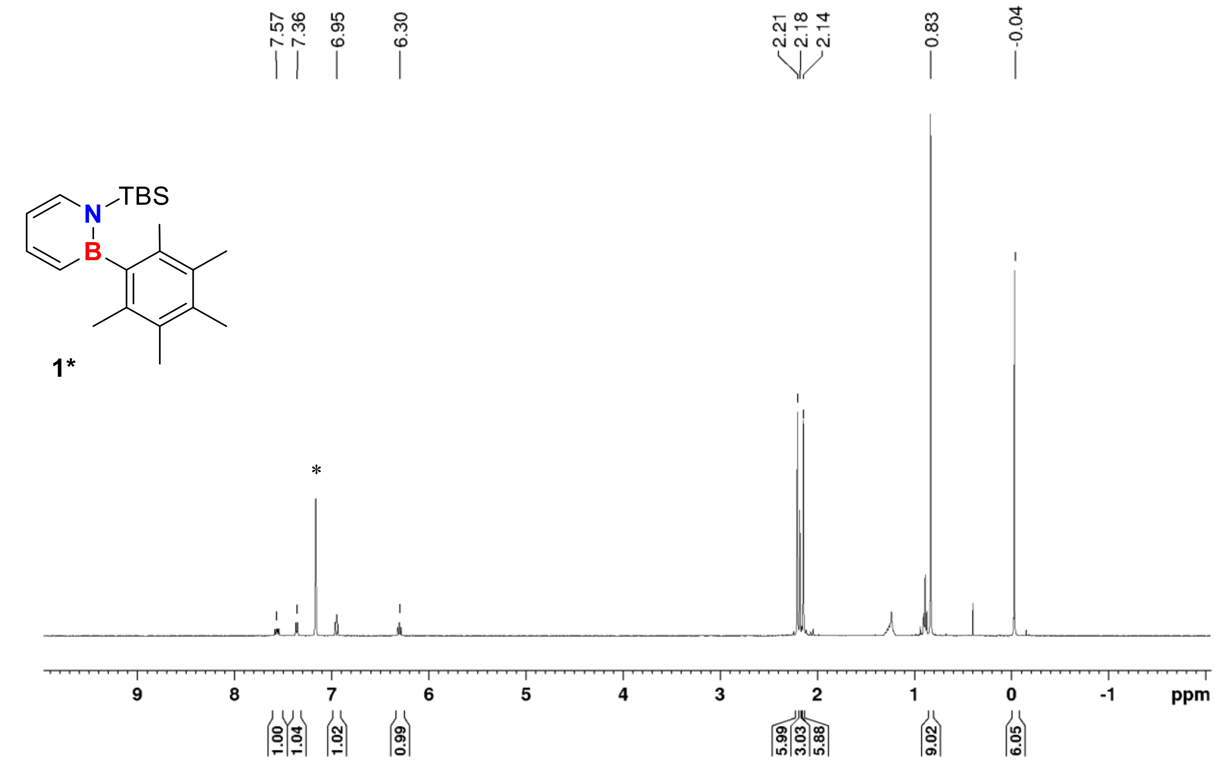

Figure S92. ^{1}H-NMR spectrum of compound **11** in C_6D_6 measured at a 400 MHz spectrometer. The solvent signal is marked with an asterisk.

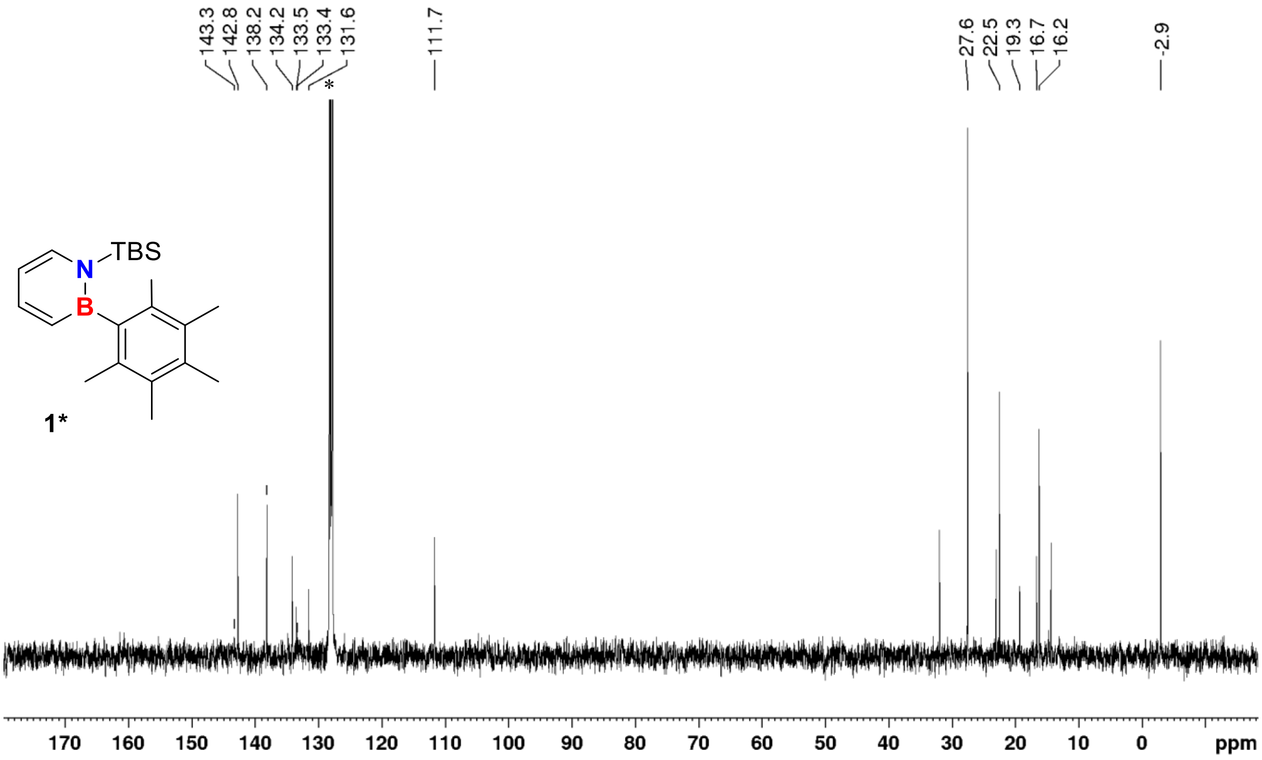

Figure S93. ^{13}C-{^{1}H}-NMR spectrum of compound **11** in C_6D_6 measured at a 400 MHz spectrometer. The solvent signal is marked with an asterisk.

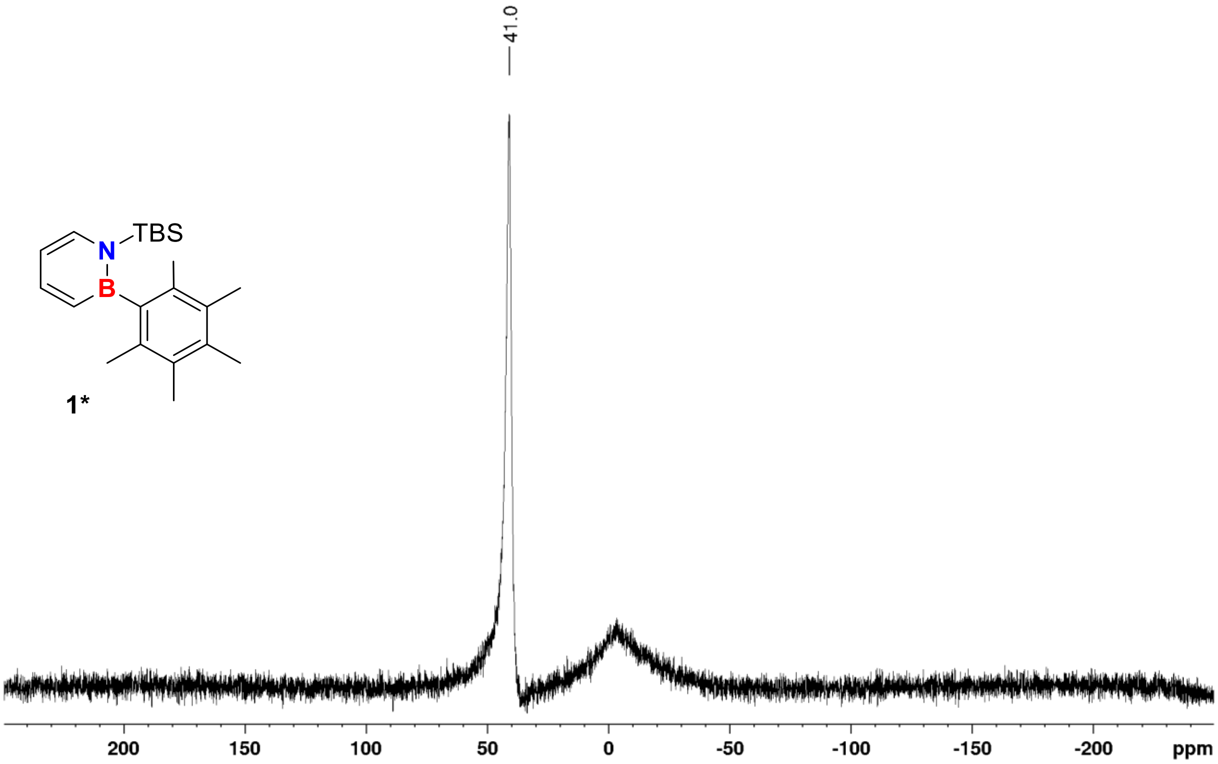

Figure S94. ^{11}B-{^{1}H}-NMR spectrum of compound **11** in C_6D_6 measured at a 400 MHz spectrometer. The broad signal between -30 and 30 ppm corresponds to the borosilicate glass of the NMR tube.

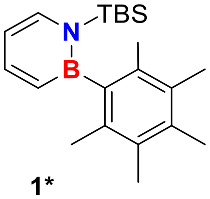

1*

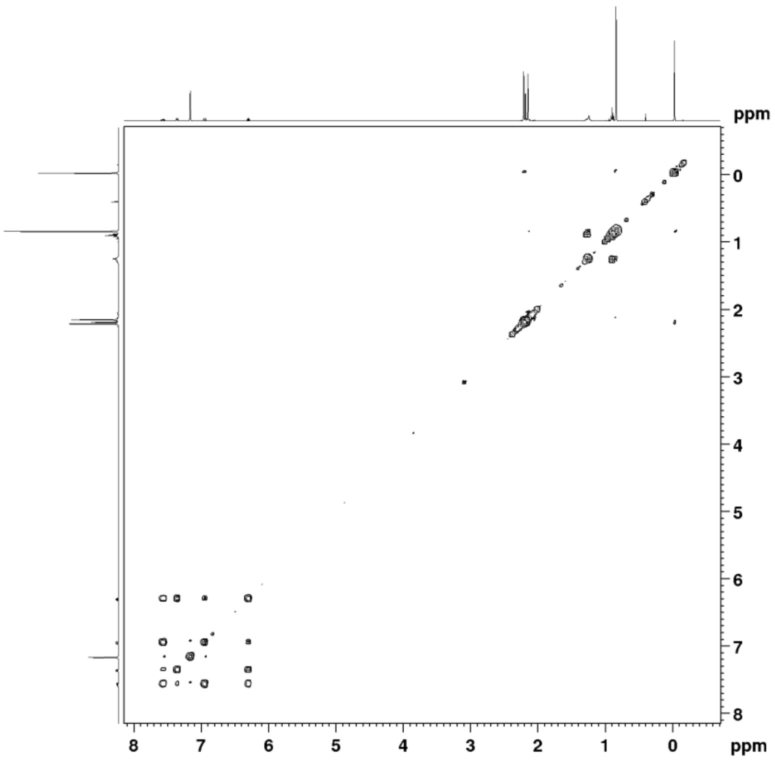

Figure S95. ^{1}H-^{1}H-COSY-NMR spectrum of compound **11** in C_6D_6 measured at a 400 MHz spectrometer.

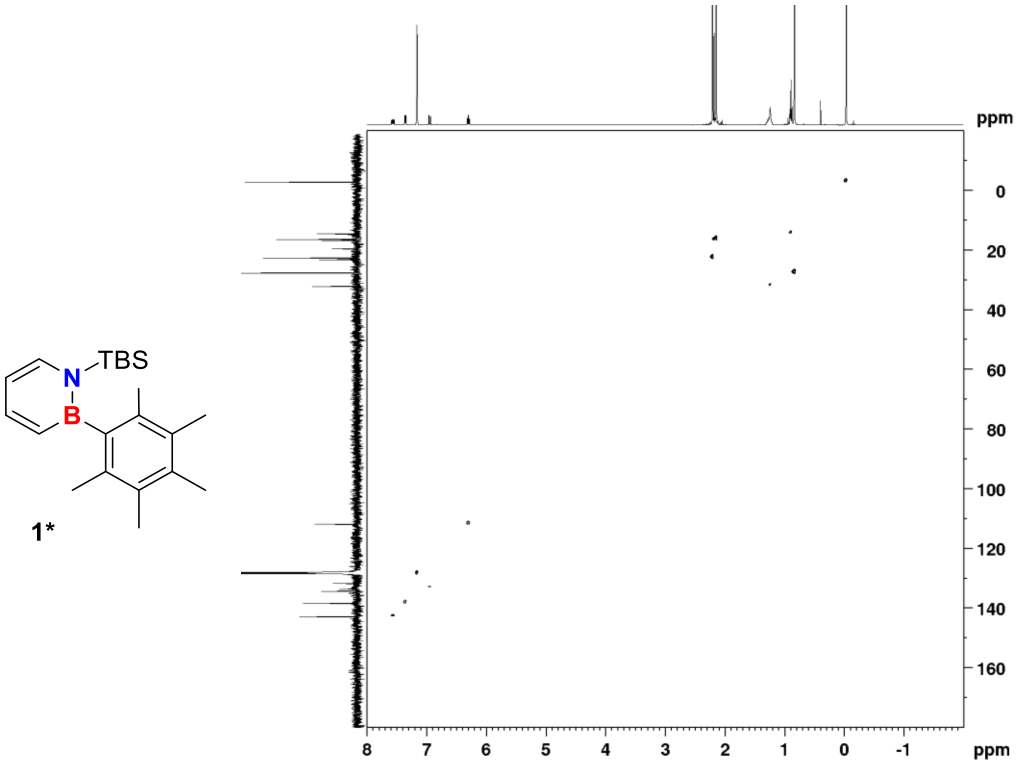

Figure S96. ^{1}H-^{13}C-HSQC-NMR spectrum of compound **11** in C_6D_6 measured at a 400 MHz spectrometer.

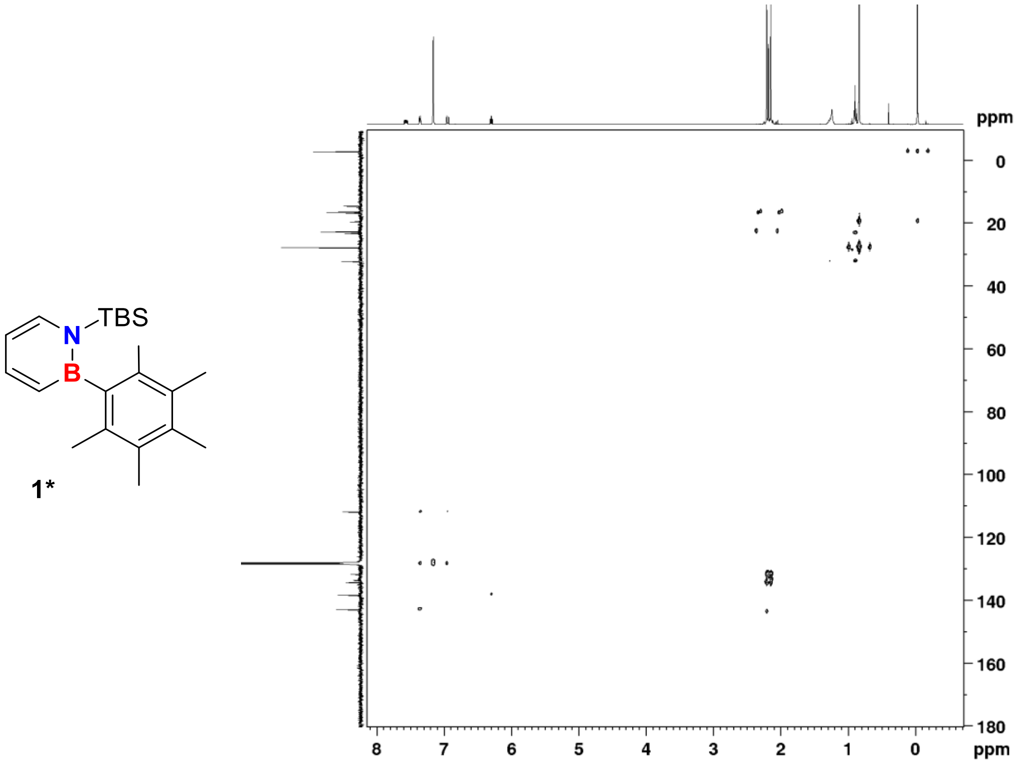

Figure S97. ^{1}H-^{13}C-HMBC-NMR spectrum of compound **11** in C_6D_6 measured at a 400 MHz spectrometer.

Synthesis of 3-Chloro-1-(*tert*-butyldimethylsilyl)-2-pentamethylphenyl-1,2-dihydro-1,2-azaborinine (6)

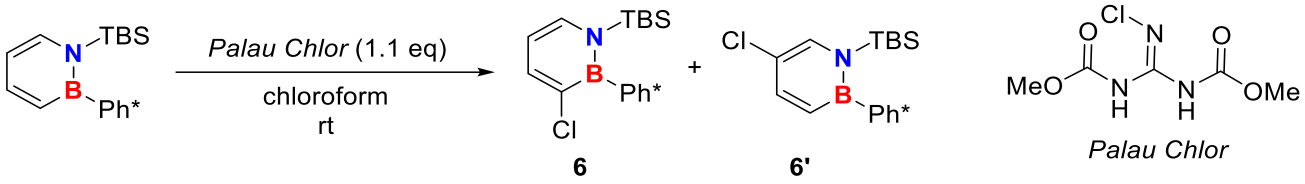

Palau Chlor® (0.21 g, 0.82 mmol, 1.1 eq) was solved in chloroform (20 mL). 1-(*tert*-butyldimethylsilyl)-2-pentamethylphenyl-1,2-dihydro-1,2-azaborinine (**11**) (0.25 g, 0.75 mmol, 1 eq) was solved in 1.5 mL chloroform and added dropwise at room temperature. The reaction mixture was stirred for 2 h at room temperature. Distilled water was added and the aqueous phase was extracted three times with *n*-hexane (15 mL). The combined organic layers were washed with brine and dried over $MgSO_4$. After removing the solvent, the crude product was purified by column chromatography (silica, *n*-hexane/dichloromethane gradient). Product **6** was obtained as a colorless solid (143.2 mg, 51%). Side product **6'** is obtained as a colorless oil (58.9 mg, 21%).

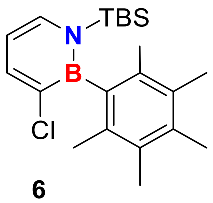

6

$C_{21}H_{33}BNClSi$ (373.85 g/mol)

^{1}H-NMR (400 MHz, **CD_2Cl_2**): δ = 7.60 (dd, $^3J_{HH}$ = 7.08 Hz, $^4J_{HH}$ = 0.98 Hz, 1H, H-4), 7.40 (dd, $^3J_{HH}$ = 6.89 Hz, $^4J_{HH}$ = 0.98 Hz, 1H, H-6), 6.34 (ps. t, 1H, H-5), 2.26 (s, 3H, H-17), 2.18 (s, 6H, H-15), 2.01 (s, 6H, H-13), 0.90 (s, 9H, H-10), -0.06 (s, 6H, H-8) ppm.

^{13}C-{^{1}H}-NMR (151 MHz, **CD_2Cl_2**): δ = 140.4 (C4), 139.0 (br. C11), 137.2 (C2), 134.4 (br. C3, detected *via* HSQC), 134.1 (C12), 133.8 (C16), 131.2 (C14), 110.0 (C5), 27.0 (C10), 21.0 (C13), 18.9 (C9), 16.1 (C17), 15.6 (C15), -3.6 (C8) ppm.

^{11}B-{^{1}H}-NMR (128 MHz, **CD_2Cl_2**): δ = 39.5 ppm.

HR-MS (ESI): m/z calc. For [M+H]+ 374.22366, found 374.22334.

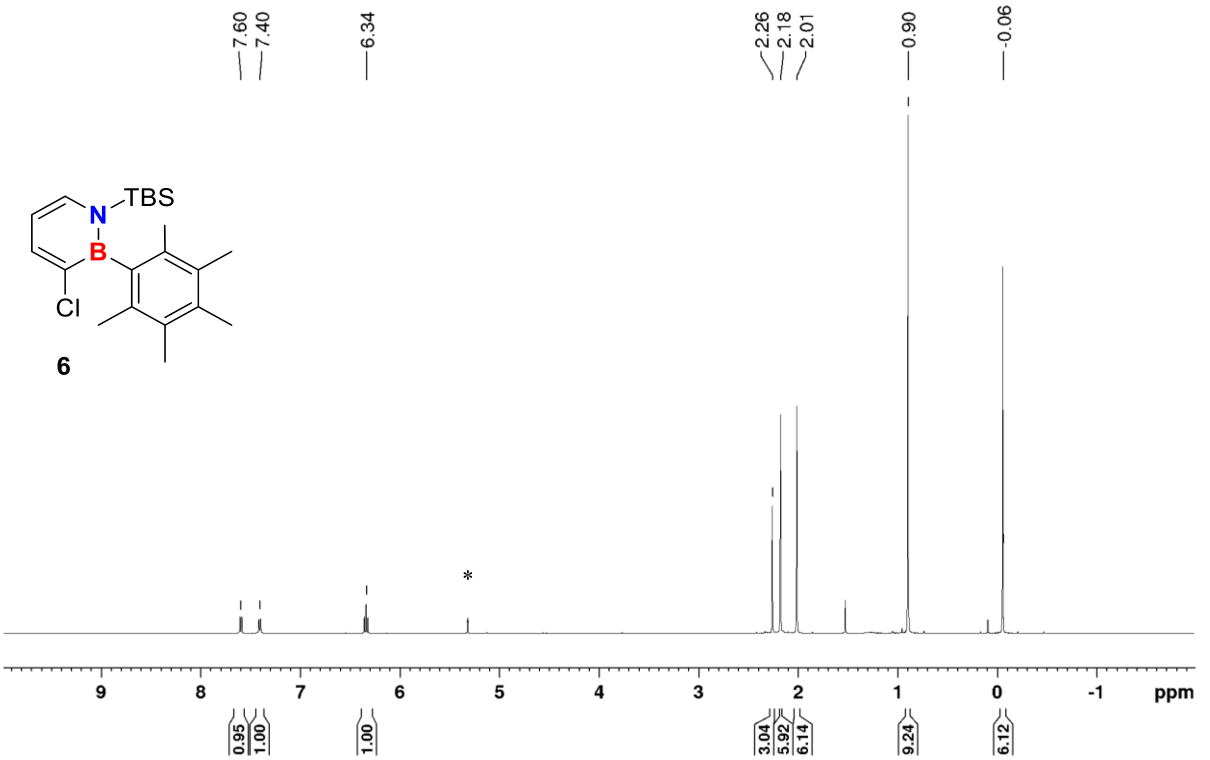

Figure S98. ^{1}H-NMR spectrum of compound **6** in CD_2Cl_2 measured at a 400 MHz spectrometer. The solvent signal is marked with an asterisk.

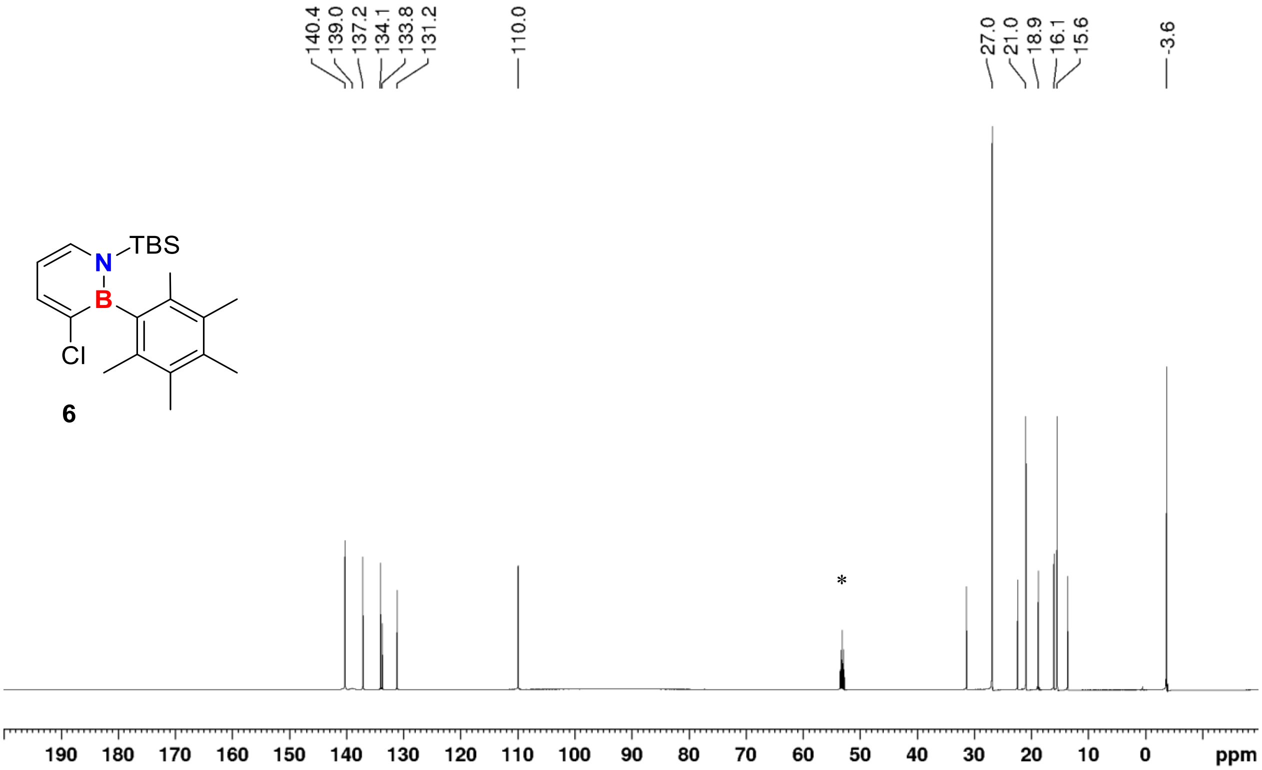

Figure S99. ^{13}C-{^{1}H}-NMR spectrum of compound **6** in CD_2Cl_2 measured at a 600 MHz spectrometer.

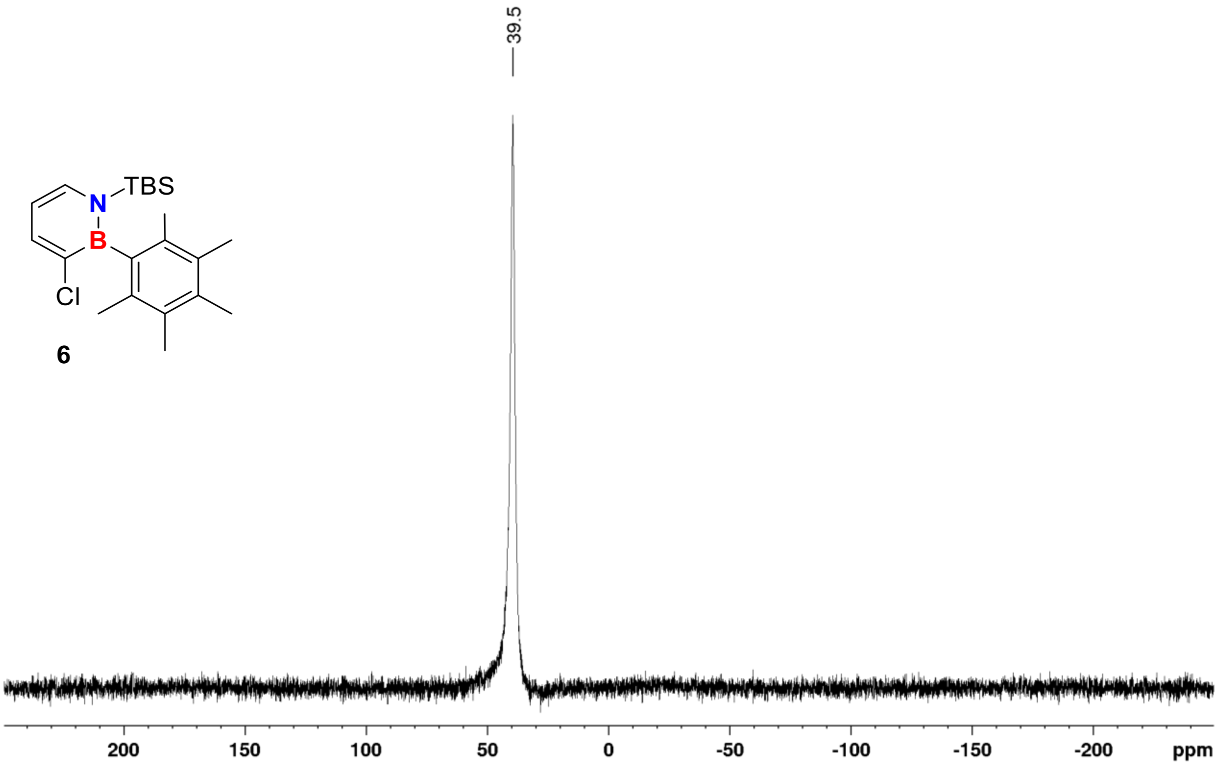

Figure S100. ^{11}B-{^{1}H}-NMR spectrum of compound **6** in CD_2Cl_2 measured at a 400 MHz spectrometer.

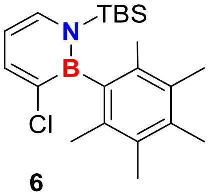

6

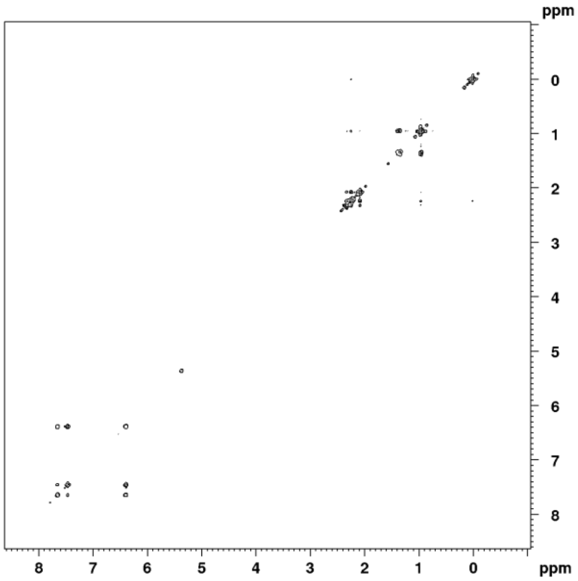

Figure S101. ^{1}H-^{1}H-COSY-NMR spectrum of compound **6** in CD_2Cl_2 measured at a 600 MHz spectrometer.

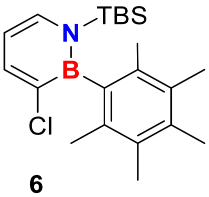

6

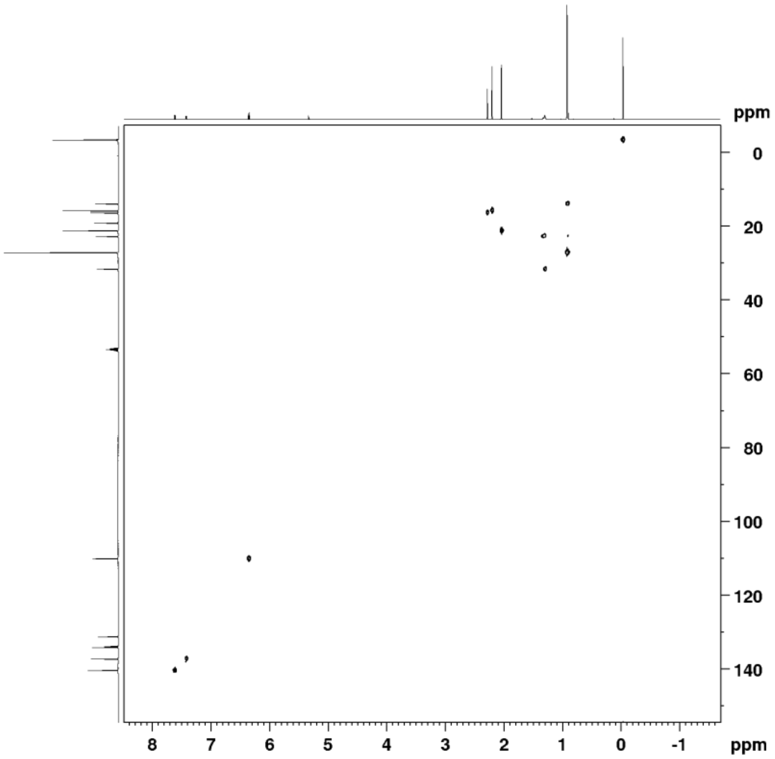

Figure S102. ^{1}H-^{13}C-HSQC-NMR spectrum of compound **6** in CD_2Cl_2 measured at a 600 MHz spectrometer.

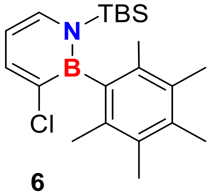

6

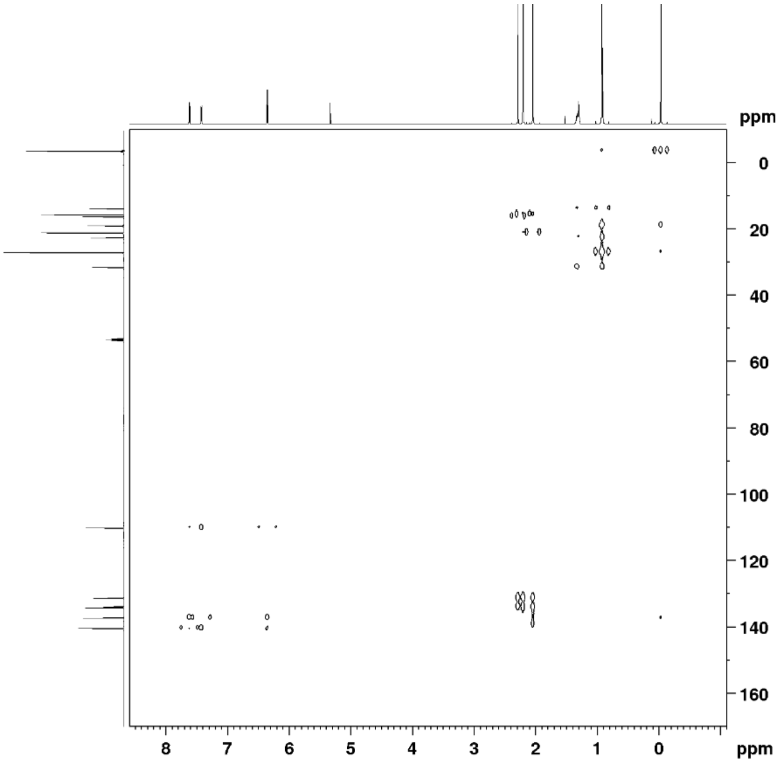

Figure S103. ^{1}H-^{13}C-HMBC-NMR spectrum of compound **6** in CD_2Cl_2 measured at a 600 MHz spectrometer.

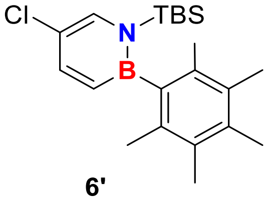

6'

$C_{21}H_{33}BNClSi$ (373.85 g/mol)

^{1}H-NMR (600 MHz, **CD_2Cl_2**): δ = 7.48 s, 1H, H-6), 1.47 (m, 1H, H-4), 6.55 (s, 1H, H-3), 2.24 (s, 3H, H-17), 2.16 (s, 6H, H-15), 2.00 (s, 6H, H-13), 0.90 (s, 9H, H-10), -0.03 (s, 6H, H-8) ppm.

^{13}C-{^{1}H}-NMR (151 MHz, **CD_2Cl_2**): δ = 143.4 (C4), 141.8 (br. C11), 135.8 (C6), 134.0 (C12), 133.8 (C16), 133.6 (br. C3), 131.6 (C14), 118.2 (C5), 27.2 (C10), 22.0 (C13), 19.1 (C9), 16.3 (C17), 15.8 (C15), -3.5 (C8) ppm.

^{11}B-{^{1}H}-NMR (193 MHz, **CD_2Cl_2**): δ = 39.7 ppm.

HR-MS (ESI): m/z calc. For [M+H]+ 374.22366, found 374.22334.

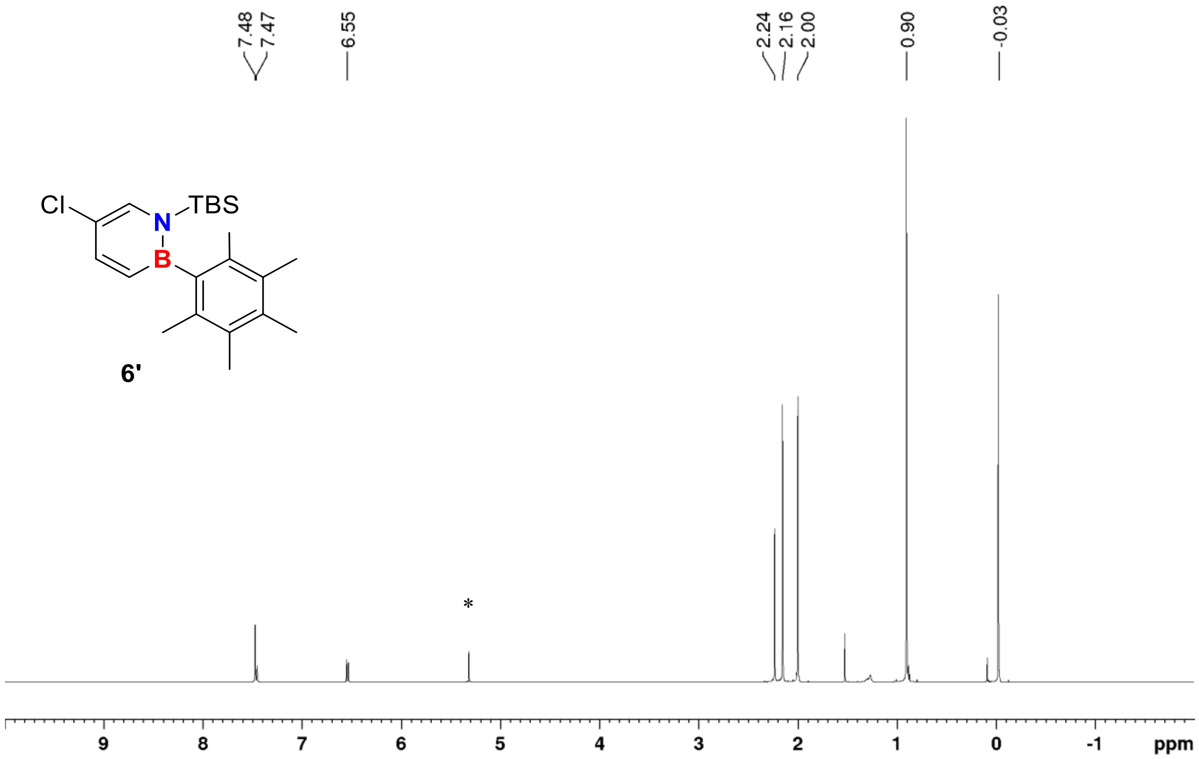

Figure S104. ^{1}H-NMR spectrum of compound **6’** in CD_2Cl_2 measured at a 400 MHz spectrometer. The solvent signal is marked with an asterisk.

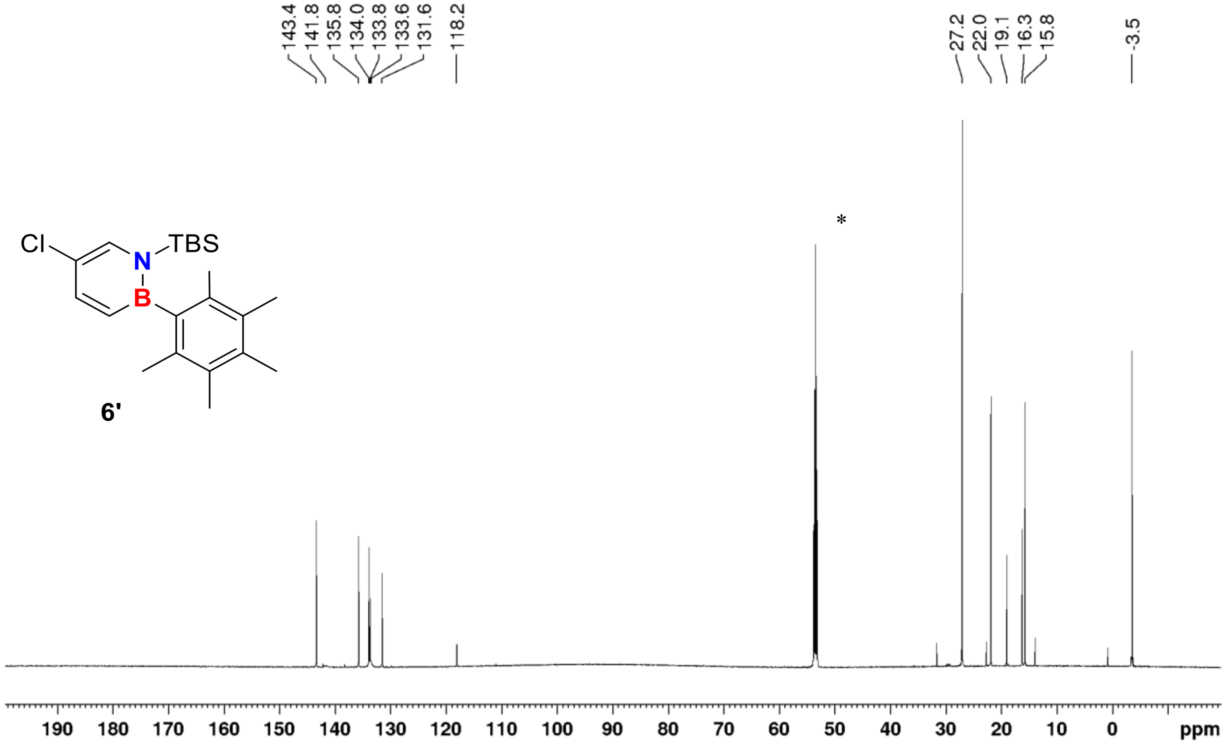

Figure S105. ^{13}C-{^{1}H}-NMR spectrum of compound **6’** in CD_2Cl_2 measured at a 600 MHz spectrometer.

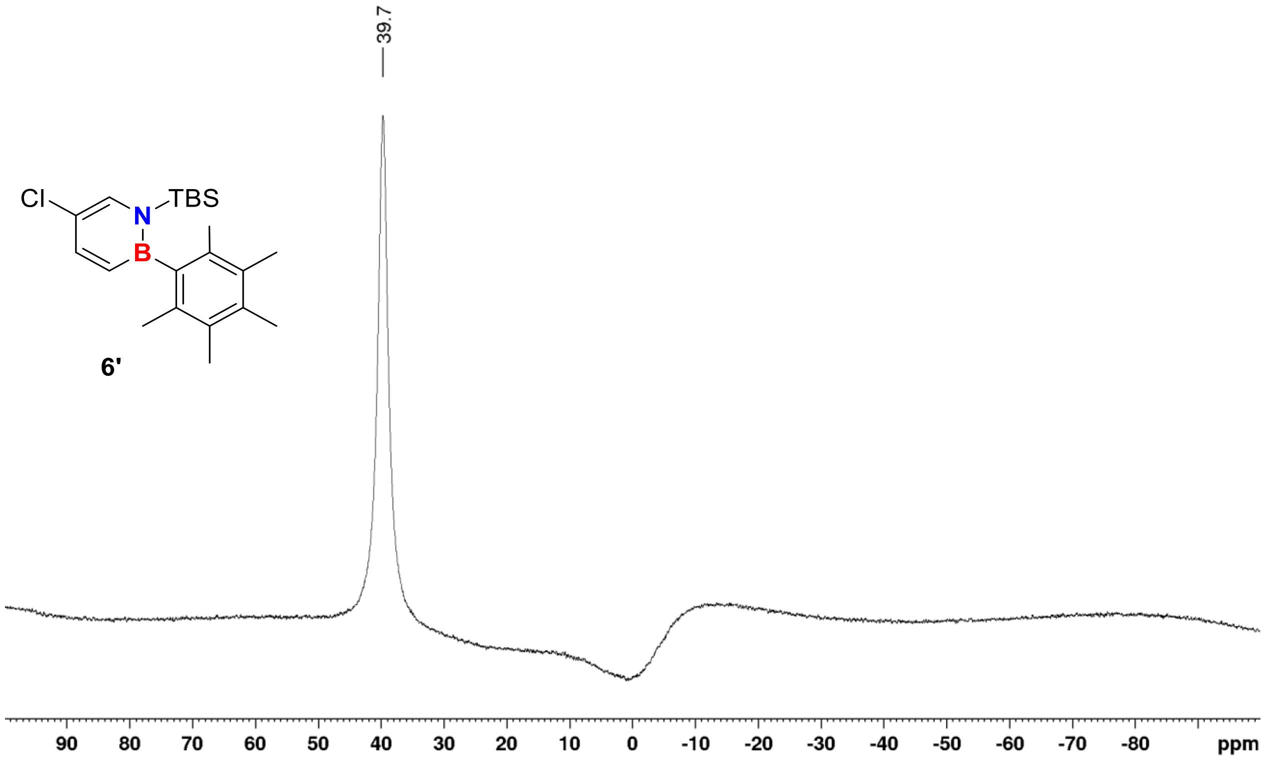

Figure S106. ^{11}B-{^{1}H}-NMR spectrum of compound **6** in CD_2Cl_2 measured at a 600 MHz spectrometer.

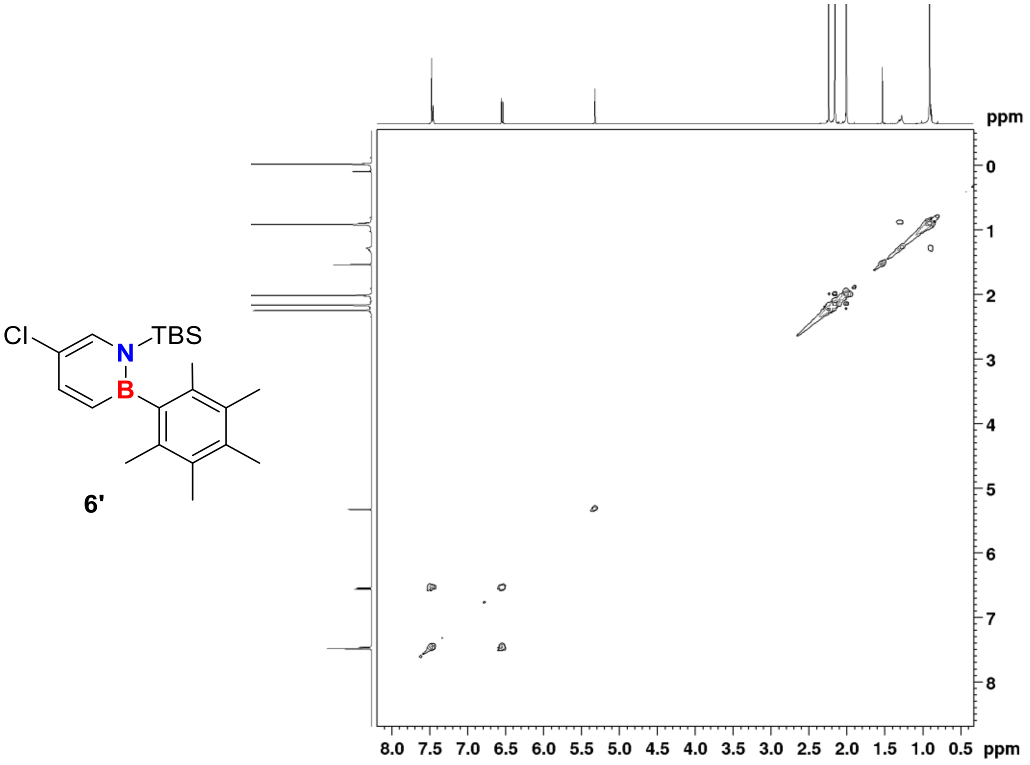

Figure S107. ^{1}H-^{1}H-COSY-NMR spectrum of compound **6’** in CD_2Cl_2 measured at a 600 MHz spectrometer.

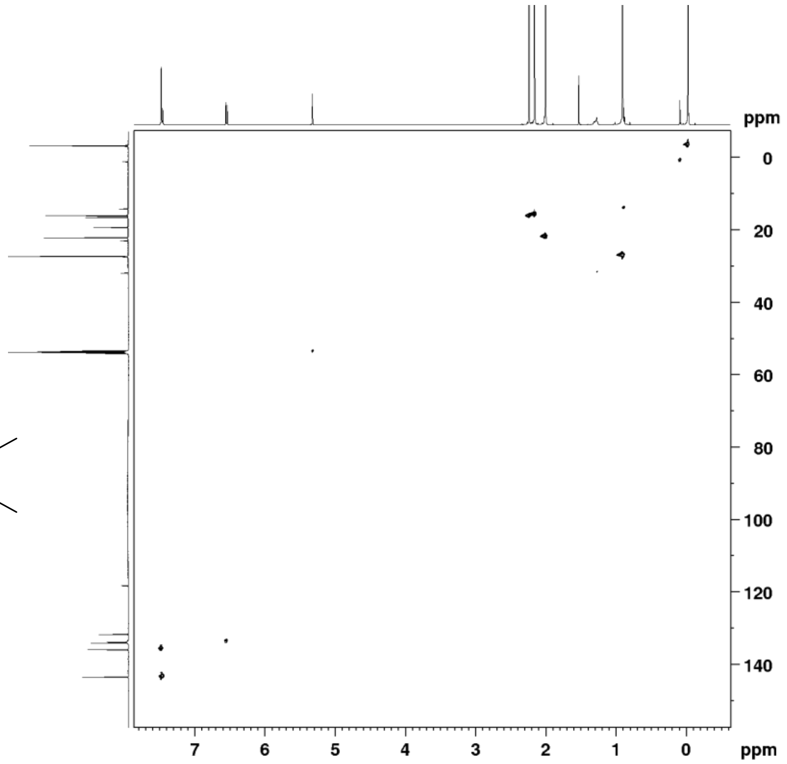

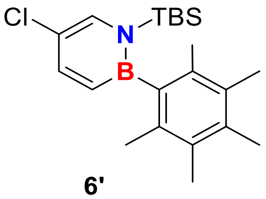

6'

Figure S108. ^{1}H-^{13}C-HSQC-NMR spectrum of compound **6’** in CD_2Cl_2 measured at a 600 MHz spectrometer.

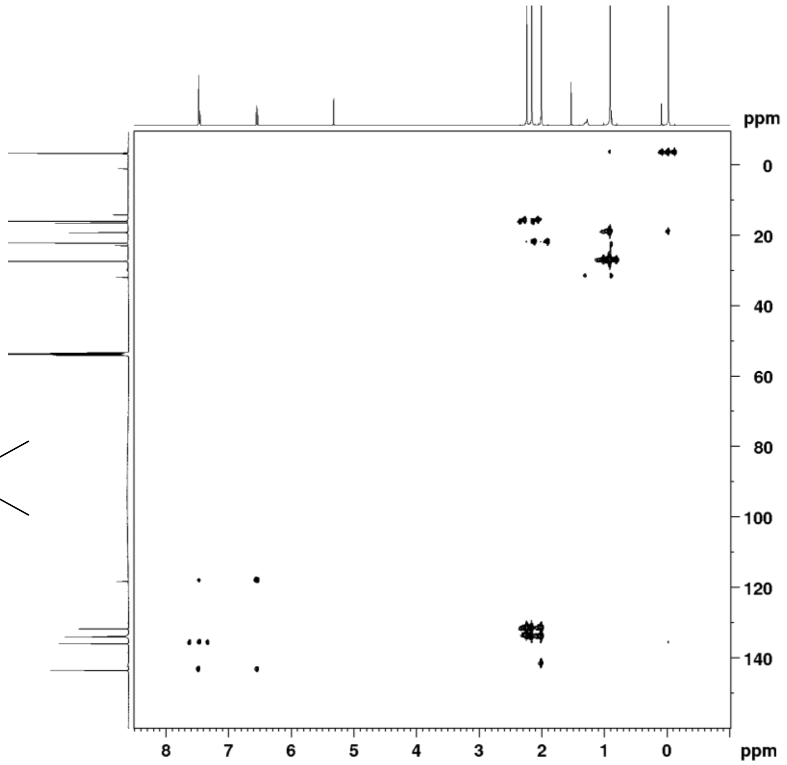

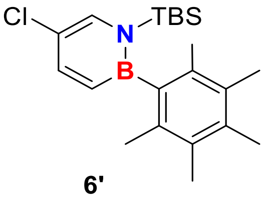

6'

Figure S109. ^{1}H-^{13}C-HMBC-NMR spectrum of compound **6’** in CD_2Cl_2 measured at a 600 MHz spectrometer.

Synthesis of 5-bromo-3-chloro-1-(*tert*-butyldimethylsilyl)-2-mesityl-1,2-dihydro-1,2-azaborinine (8)

3-Chloro-1-(*tert*-butyldimethylsilyl)-2-pentamethylphenyl-1,2-dihydro-1,2-azaborinine (**8**) (93.5 mg, 0.24 mmol, 1 eq) was solved in dichloromethane (5 mL). A bromine solution (14 µL, 0.26 mmol, 1.05 eq) in dichloromethane (3 mL) was added dropwise at 0 °C. The reaction mixture was allowed to warm to room temperature and stirred for 3 h. The reaction was *quenched* by adding a saturated Na_2SO_3 solution. The aqueous layer was extracted three times with *n*-hexane (15 mL). The combined organic layer was washed with brine and dried over $MgSO_4$. After removing the solvent, the crude product was purified by column chromatography (silica, *n*-hexane/dichloromethane gradient). Product **8** was obtained as a colorless solid (101.1 mg, 89%).

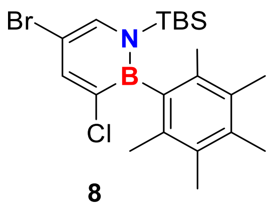

8

$C_{21}H_{32}BNClBrSi$ (452.74 g/mol)

^{1}H-NMR (600 MHz, **CD_2Cl_2**): δ = 7.71 (d, $^4J_{HH}$ = 1.74 Hz, 1H, H-4), 7.53 (d, $^4J_{HH}$ = 1.74 Hz, 1H, H-6), 2.25 (s, 3H, H-17), 2.17 (s, 6H, H-), 2.00 (s, 6H, H-), 0.90 (s, 9H, H-10), -0.05 (s, 6H, H-8) ppm.

^{13}C-{^{1}H}-NMR (600 MHz, **CD_2Cl_2**): δ = 143.5 (C4), 141.6 (br. C3), 138.2 (br. C11), 137.4 (C6), 134.6 (C16), 134.4 (C12), 131.7 (C14), 103.4 (C5), 27.1 (C10), 21.4 (C13), 19.1 (C9), 16.5 (C17), 15.8 (C15), -3.4 (C8) ppm.

^{11}B-{^{1}H}-NMR (192 MHz, **CD_2Cl_2**): δ = 38.8 ppm.

HR-MS (ESI): m/z calc. For [2M+Ag]+ 1009.15834, found 1009.16032.

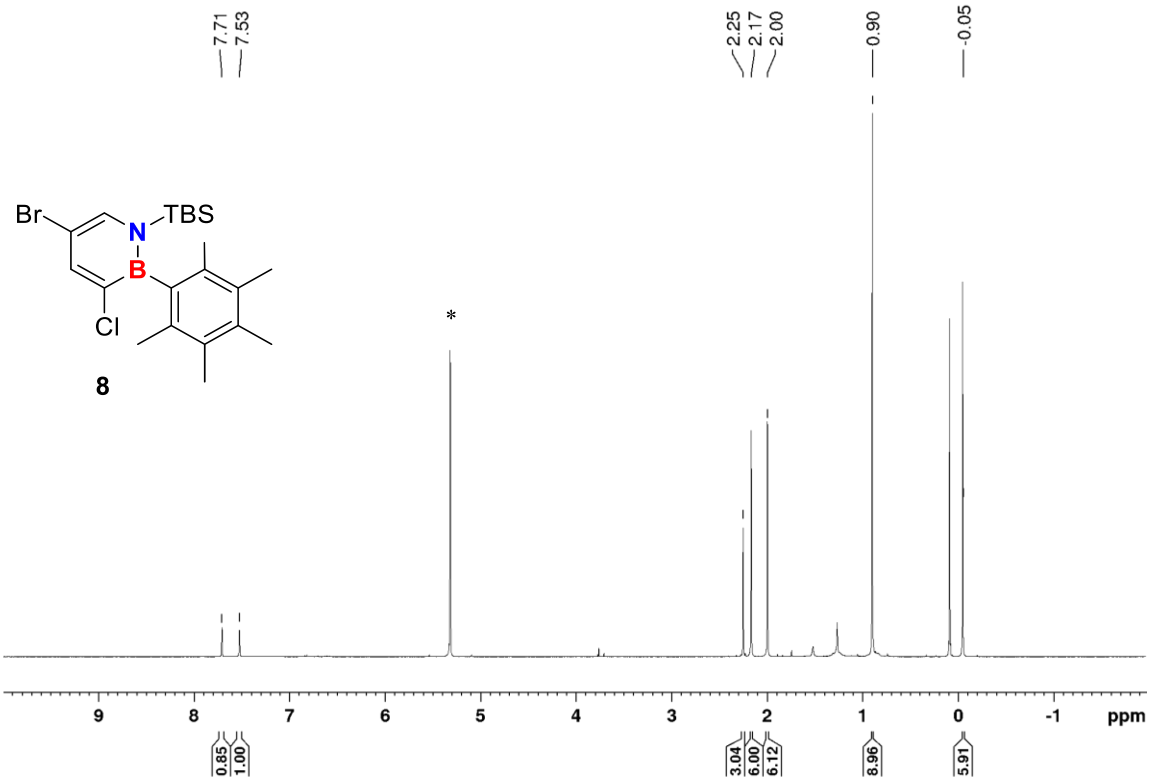

Figure S110. ^{1}H-NMR spectrum of compound **8** in CD_2Cl_2 measured at a 600 MHz spectrometer. The solvent signal is marked with an asterisk.

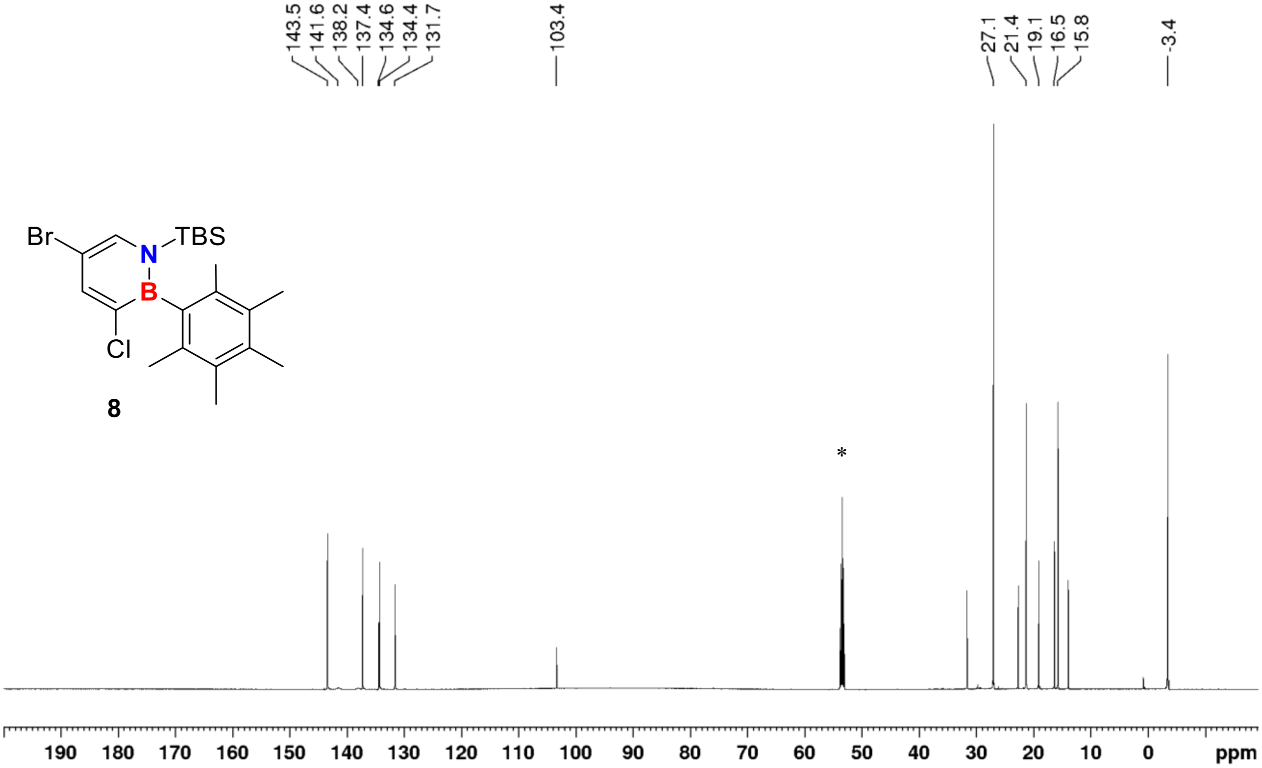

Figure S111. ^{13}C-{^{1}H}-NMR spectrum of compound **8** in CD_2Cl_2 measured at a 600 MHz spectrometer.

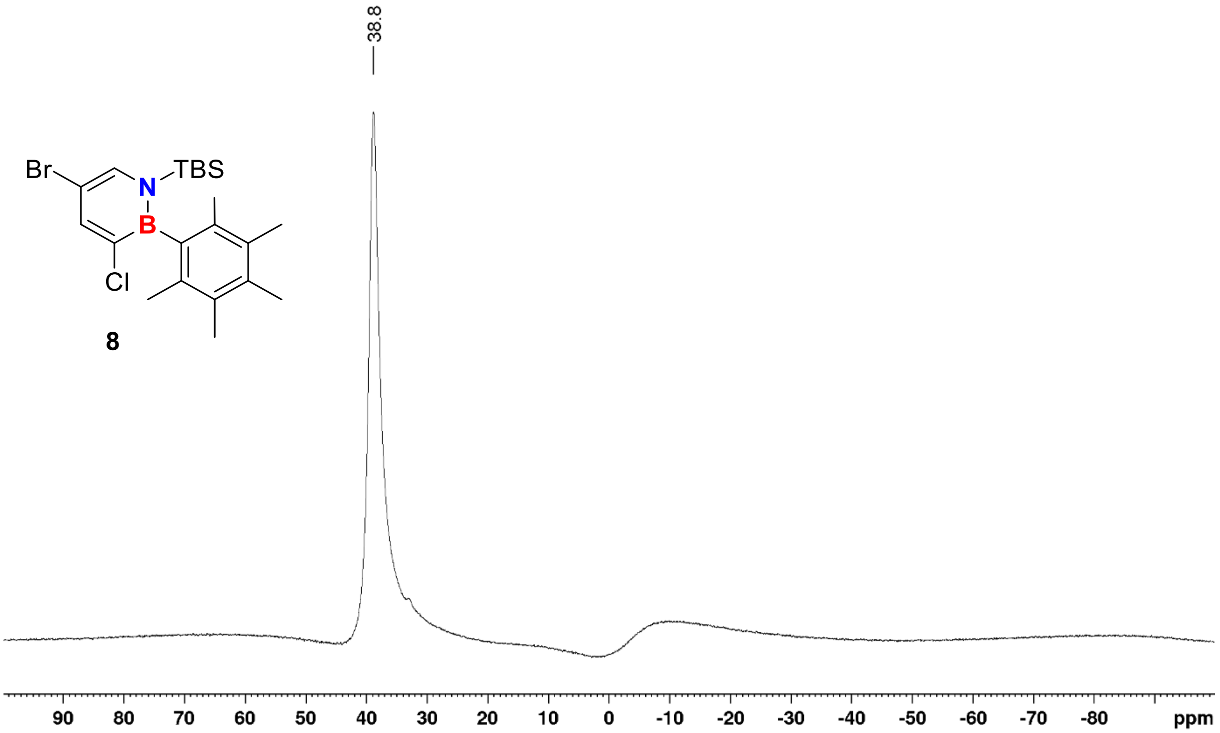

8

Figure S112. ^{11}B-{^{1}H}-NMR spectrum of compound **8** in CD_2Cl_2 measured at a 600 MHz spectrometer.

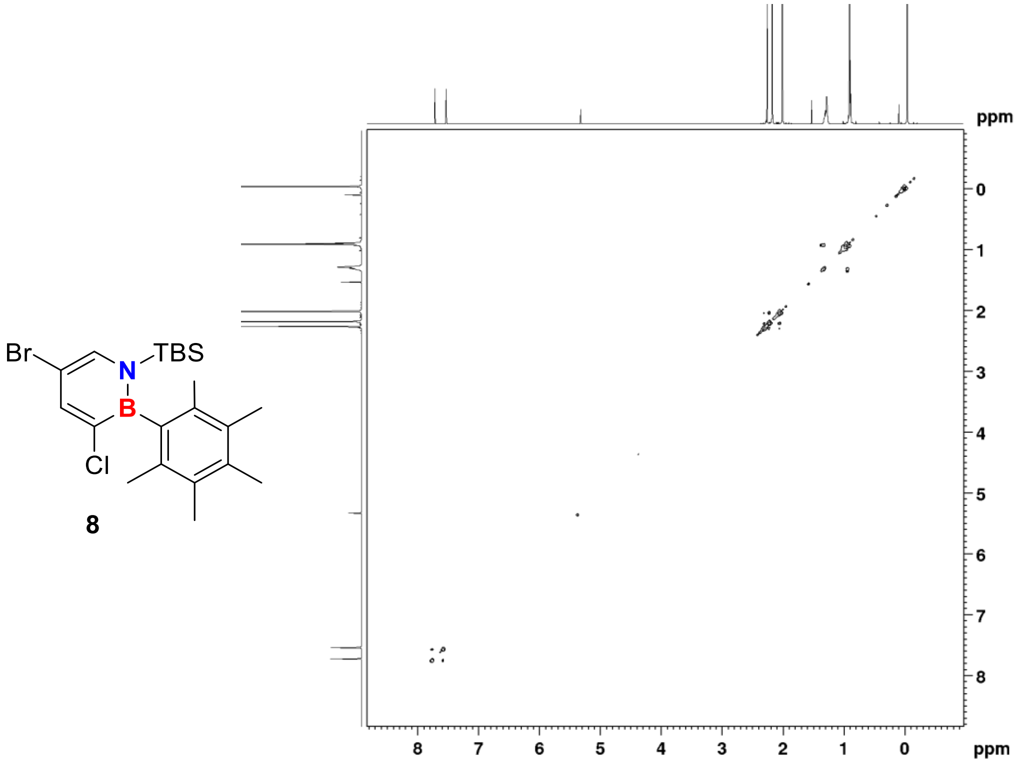

8

Figure S113. ^{1}H-^{1}H-COSY-NMR spectrum of compound **8** in CD_2Cl_2 measured at a 600 MHz spectrometer.

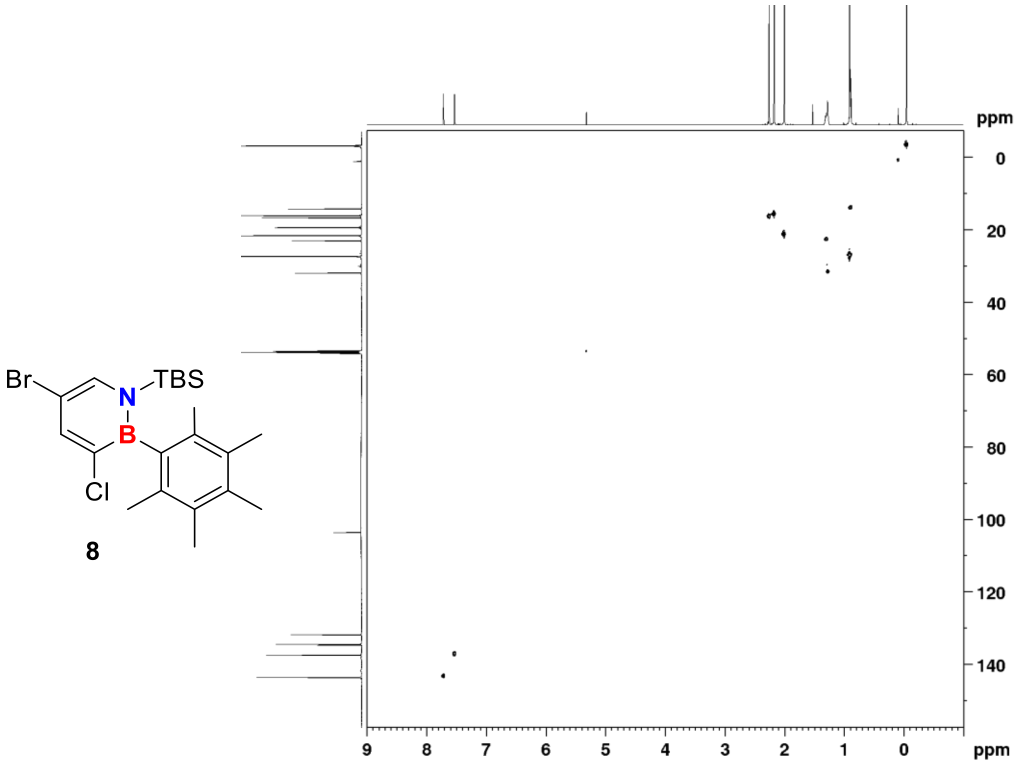

Figure S114. ^{1}H-^{13}C-HSQC-NMR spectrum of compound **8** in CD_2Cl_2 measured at a 600 MHz spectrometer.

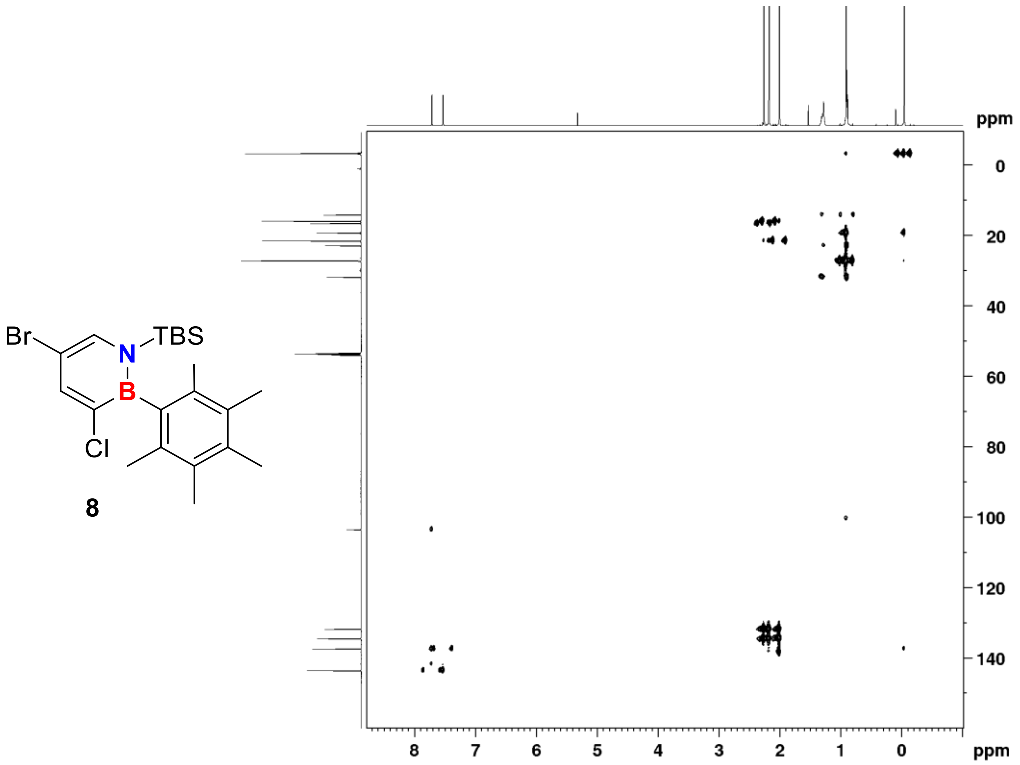

Figure S115. ^{1}H-^{13}C-HMBC-NMR spectrum of compound **8** in CD_2Cl_2 measured at a 600 MHz spectrometer.

Synthesis of 3-chloro-1-(*tert*-butyldimethylsilyl)-2-pentamethylphenyl-5-phenyl-1,2-dihydro-1,2-azaborinine (9) – Route A

5-bromo-3-chloro-1-(*tert*-butyldimethylsilyl)-2-mesityl-1,2-dihydro-1,2-azaborinine (**8**) (25 mg, 0.05 mmol, 1eq) and phenyl boronic acid (10.8 mg, 0.08 mmol, 1.5 eq) were placed in a Schlenk. In a glovebox dry Cs_2CO_3 (35.4 mg, 0.11 mmol, 2eq), $Pd(P^tBu_3)_2G2$ (1.3 mg, 4mol%) and dry, degassed toluene (1.5 mL) were added. The flask was sealed with a septum and transferred to a Schlenk line. Distilled, degassed water (0.3 mL) was added. The reaction mixture was heated to 55 °C for 16 h. Distilled water was added and the aqueous phase was extracted three times with *n*-hexane (5 mL). The combined organic layers were washed with brine and dried over $MgSO_4$. After removing the solvent, the crude product was purified by column chromatography (silica, *n*-hexane/dichloromethane gradient). Product **9** was obtained as a colorless solid ().

Synthesis of 3-chloro-1-(*tert*-butyldimethylsilyl)-2-pentamethylphenyl-5-phenyl-1,2-dihydro-1,2-azaborinine (9) – Route B

5-bromo-3-chloro-1-(*tert*-butyldimethylsilyl)-2-mesityl-1,2-dihydro-1,2-azaborinine (**8**) (25 mg, 0.05 mmol, 1eq) and phenyl boronic acid (10.8 mg, 0.08 mmol, 1.5 eq) were placed in a Schlenk. In a glovebox dry Cs_2CO_3 (35.4 mg, 0.11 mmol, 2eq), $Pd(PCy_3)_2G2$ (1.3 mg, 4mol%) and dry, degassed toluene (1.5 mL) were added. The flask was sealed with a septum and transferred to a Schlenk line. Distilled, degassed water (0.3 mL) was added. The reaction mixture was heated to 55 °C for 16 h. Distilled water was added and the aqueous phase was extracted three times with *n*-hexane (5 mL). The combined organic layers were washed with brine and dried over $MgSO_4$. After removing the solvent, the crude product was purified by column chromatography (silica, *n*-hexane/dichloromethane gradient). Product **9** was obtained as a colorless solid ().

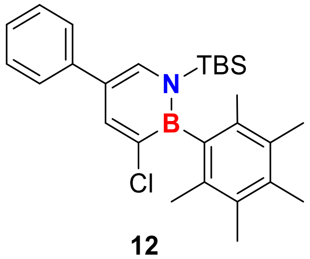

12

$C_{28h38b}NClSi$ (449.94 g/mol)

^{1}H-NMR (400 MHz, **C_6D_{12}**): δ = 7.85 (d, $^4J_{HH}$ = 1.51 Hz, 1H, H-4), 7.55 (d, $^4J_{HH}$ = 1.51 Hz, 1H, H-6), 7.38 (m, 2H, H-), 7.29 (m, 2H, H-), 7.17 (m, 1H, H-21), 0.95 (s, 9H, H-10), -0.03 (s, 6H, H-8) ppm.

^{13}C-{^{1}H}-NMR (400 MHz, **C_6D_{12}**): δ = 141.2 (C4), 141.0 (C3), 139.5 (C3), 138.8 (C11), 136.1 (C6), 134.5 (C12), 134.3 (C16), 131.6 (C14), 129.0 (C19), 126.8 (C21), 126.5 (C20), 124.4 (C5), 27.4 (C10), 21.4 (C13), 19.2 (C9), 16.5 (C17), 15.9 (C15), -3.2 (C8) ppm.

^{11}B-{^{1}H}-NMR (192 MHz, **C_6D_{12}**): δ = 39.4 ppm.

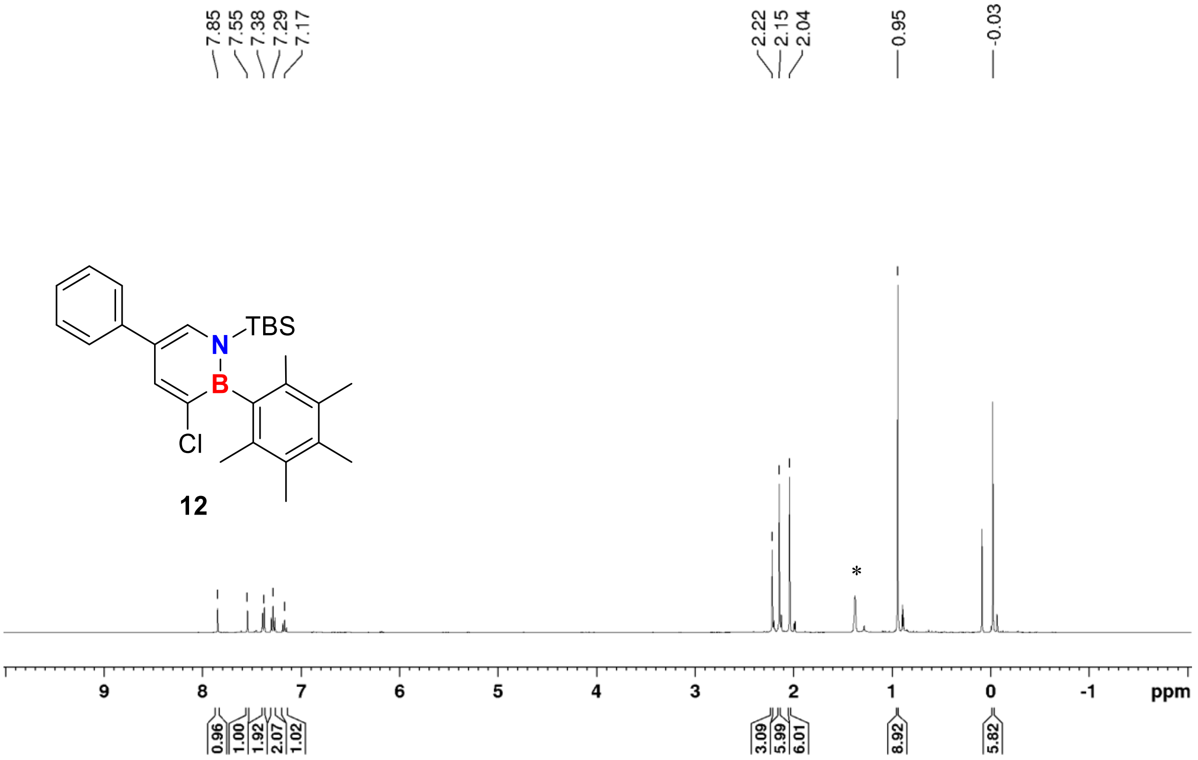

Figure S116. ^{1}H-NMR spectrum of compound **9** in C_6D_{12} measured at a 400 MHz spectrometer. The solvent signal is marked with an asterisk.

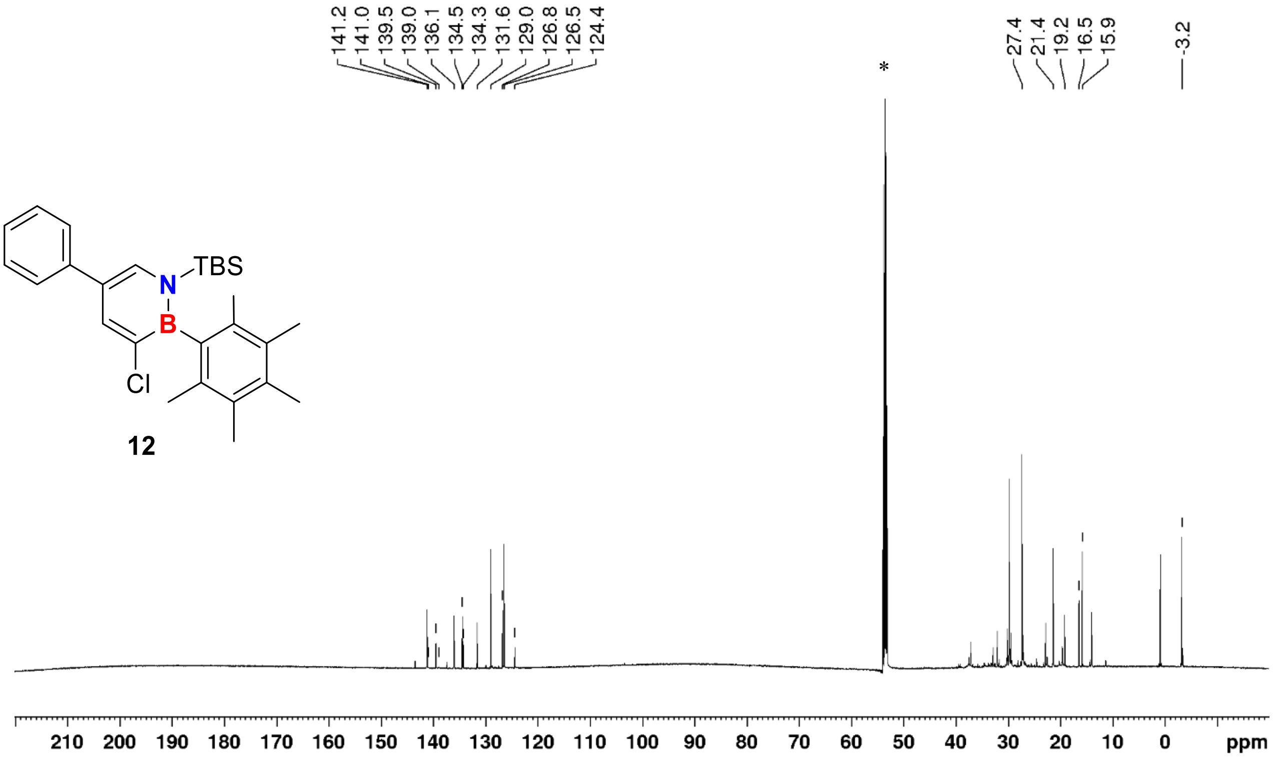

Figure S117. ^{13}C-{^{1}H}-NMR spectrum of compound **9** in CD_2Cl_2 measured at a 600 MHz spectrometer.

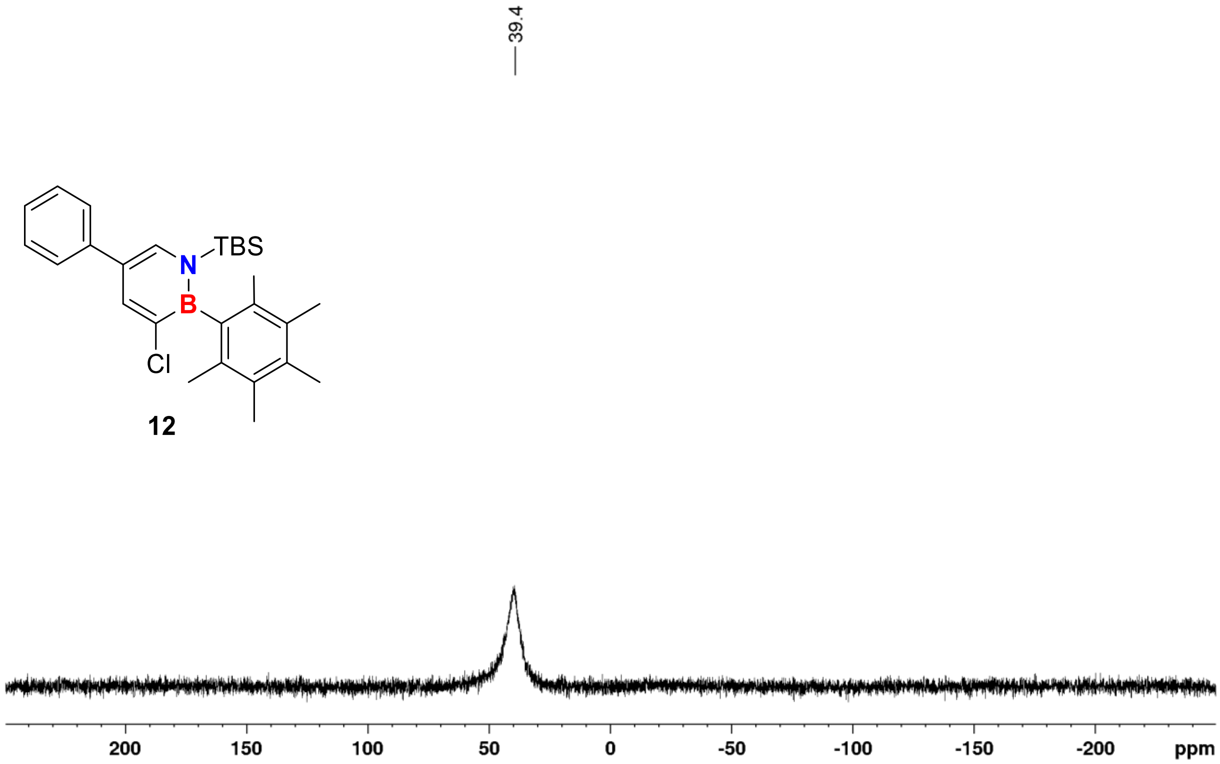

Figure S118. ^{11}B-{^{1}H}-NMR spectrum of compound **9** in C_6D_{12} measured at a 400 MHz spectrometer.

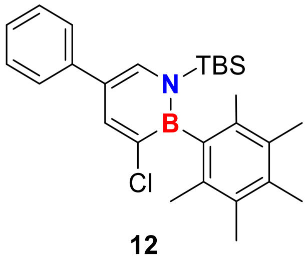

12

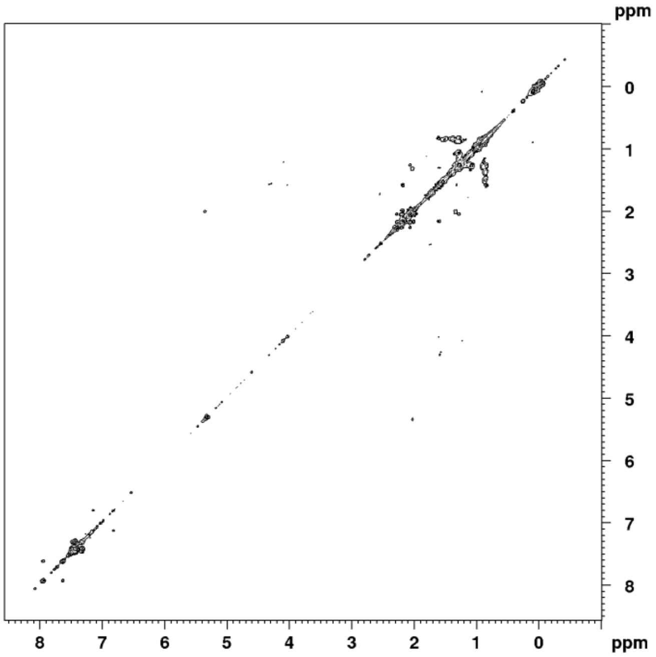

Figure S119. ^{1}H-^{1}H-COSY-NMR spectrum of compound **9** in CD_2Cl_2 measured at a 600 MHz spectrometer.

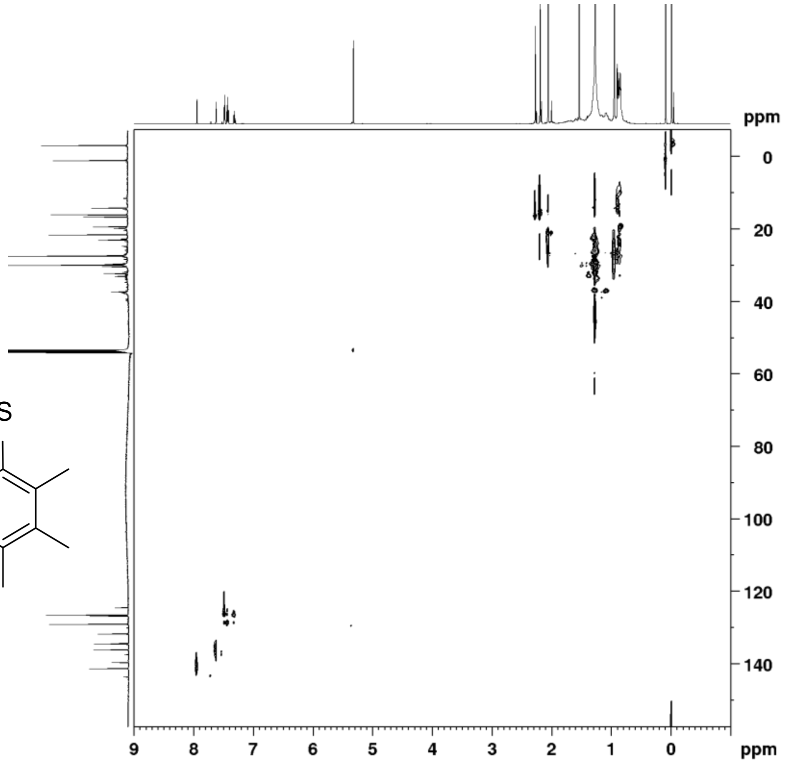

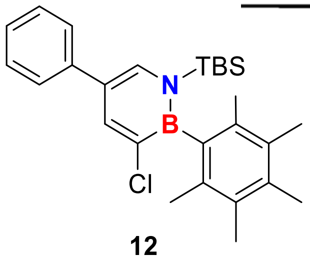

12

Figure S120. ^{1}H-^{13}C-HSQC-NMR spectrum of compound **9** in CD_2Cl_2 measured at a 600 MHz spectrometer.

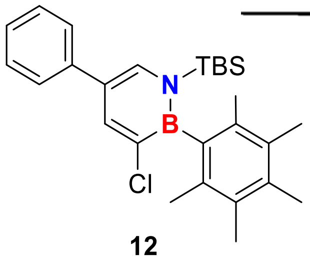

12

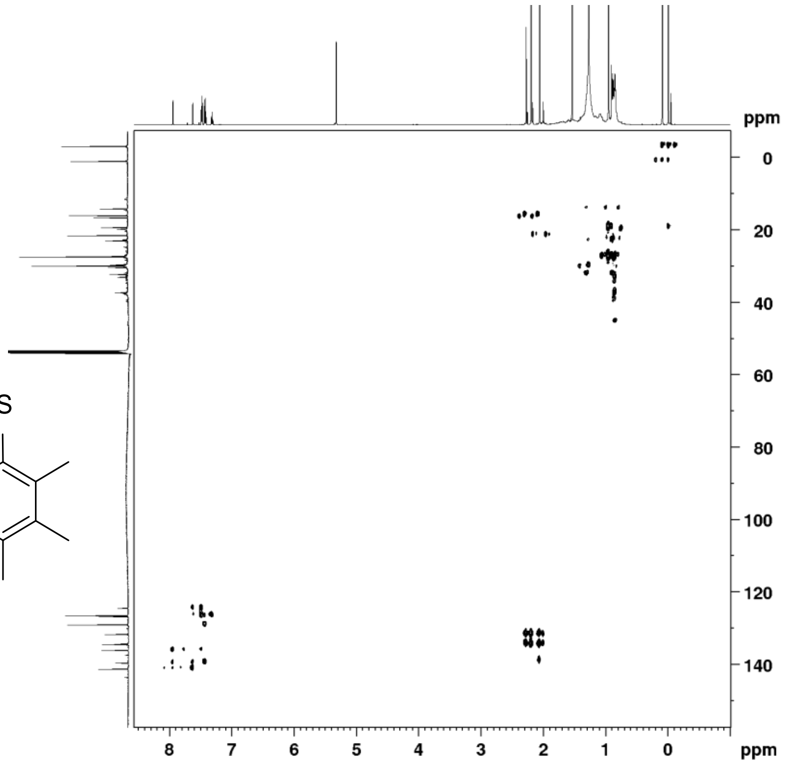

Figure S121. ^{1}H-^{13}C-HMBC-NMR spectrum of compound **9** in CD_2Cl_2 measured at a 600 MHz spectrometer.

4. Irradiation experiments

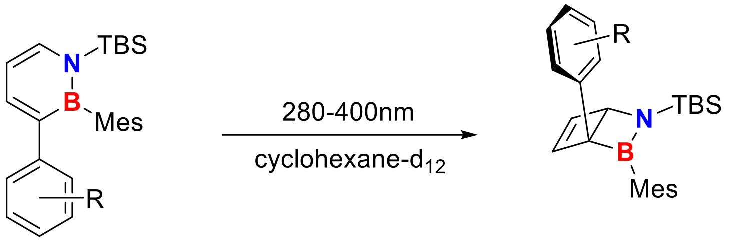

8 **8**$_{Dewar}$

All irradiations were carried out using a dichroitic mirror that selects the wavelength range 280-400 nm, without additional filters. The samples were placed in quartz J. Young NMR tubes and solved in deuterated cyclohexane under argon before the irradiation. Upon irradiation the sample was cooled with compressed air and a fan to prevent thermal cycloreversion. The exact irradiation time was dependent on the concentrations of the solution. The irradiation time to reach above 95% conversion to the *Dewar* isomer in 0.05 M solutions was 2 minutes for **8a-8k**. The irradiation yielded the compounds **1**$_{Dewar}$-**6**$_{Dewar}$ in almost quantitative yield.

NMR data of 8a*Dewar*

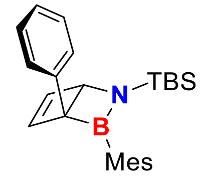

8a*Dewar*

$C_{25}H_{34}BNSi$ (387.26 g/mol)

^{1}H-NMR (600 MHz, **C_6D_{12}**): δ = 7.05 (m, 4H, H-18/H-19), 6.93 (m, 1H, H-20), 6.68 (ps. t, 1H, H-5), 6.62 (br. m, 2H, H-14), 6.48 (d, $^{4}J_{HH}$ = 2.51 Hz, 1H, H-4), 4.88 (d, $^{4}J_{HH}$ = 2.51 Hz, 1H, H-6), 2.24 (br. s, 3H, H-13), 2.18 (s, 3H, H-16), 1.96 (br. s, 3H, H-13), 0.93 (s, 9H, H-10), -0.03 (s, 3H, H-8), -0.11 (s, 3H, H-8) ppm.

^{13}C-{^{1}H}-NMR (151 MHz, **C_6D_{12}**): δ = 147.8 (C5), 141.7 (C15), 139.3 (br. C11), 138.7 (br. C11), 138.3 (C4), 137.4 (C15), 128.6 (C20), 127.7 (br. C14), 127.0 (C18), 125.3 (C19), 69.1 (C6), 60.2 (C3), 27.8 (C10), 23.2 (br. C13), 22.5 (br. C13), 21.4 (C16), 18.8 (C9), -5.3 (C8), -5.4 (C8) ppm.

^{11}B-{^{1}H}-NMR (192 MHz, **C_6D_{12}**): δ = 59.0 ppm.

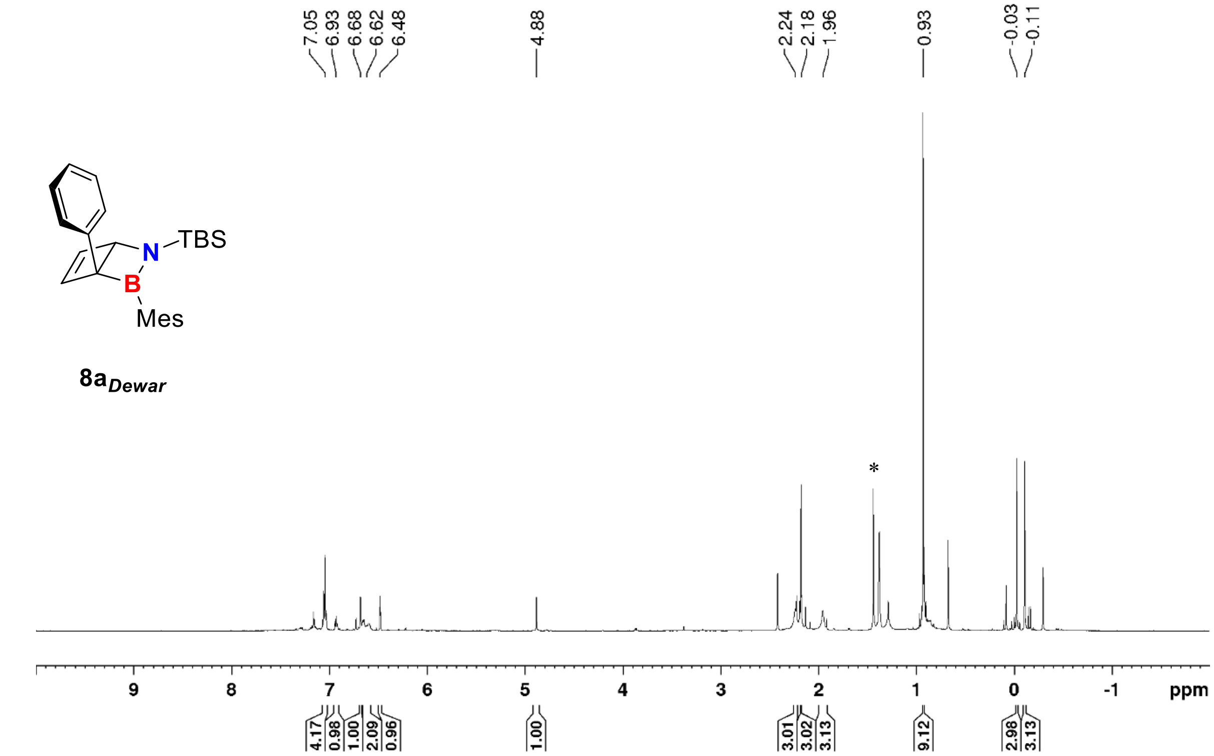

Figure S122. ^{1}H-NMR spectrum of compound **8a***Dewar* in C6D12 measured at a 600 MHz spectrometer. The solvent signal is marked with an asterisk.

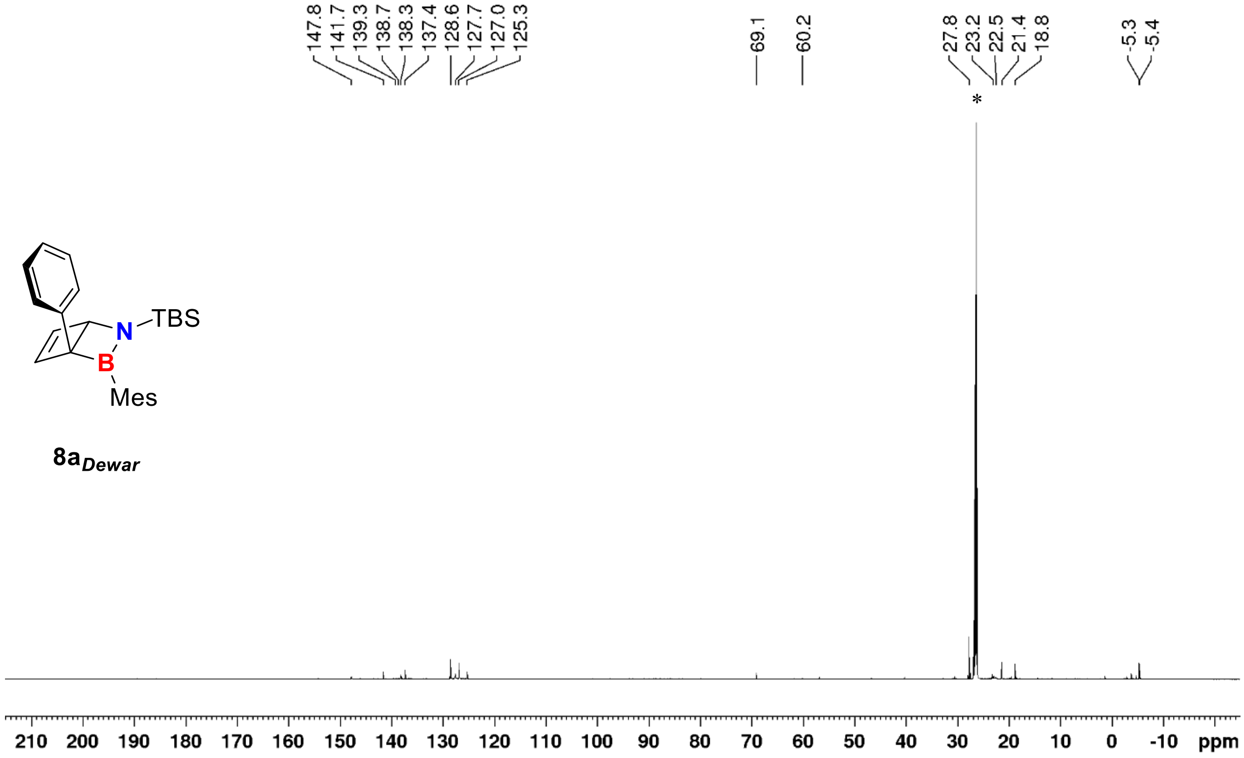

Figure S123. ^{13}C-{^{1}H}-NMR spectrum of compound **8a***Dewar* in C_6D_{12} measured at a 600 MHz spectrometer. The solvent signal is marked with an asterisk.

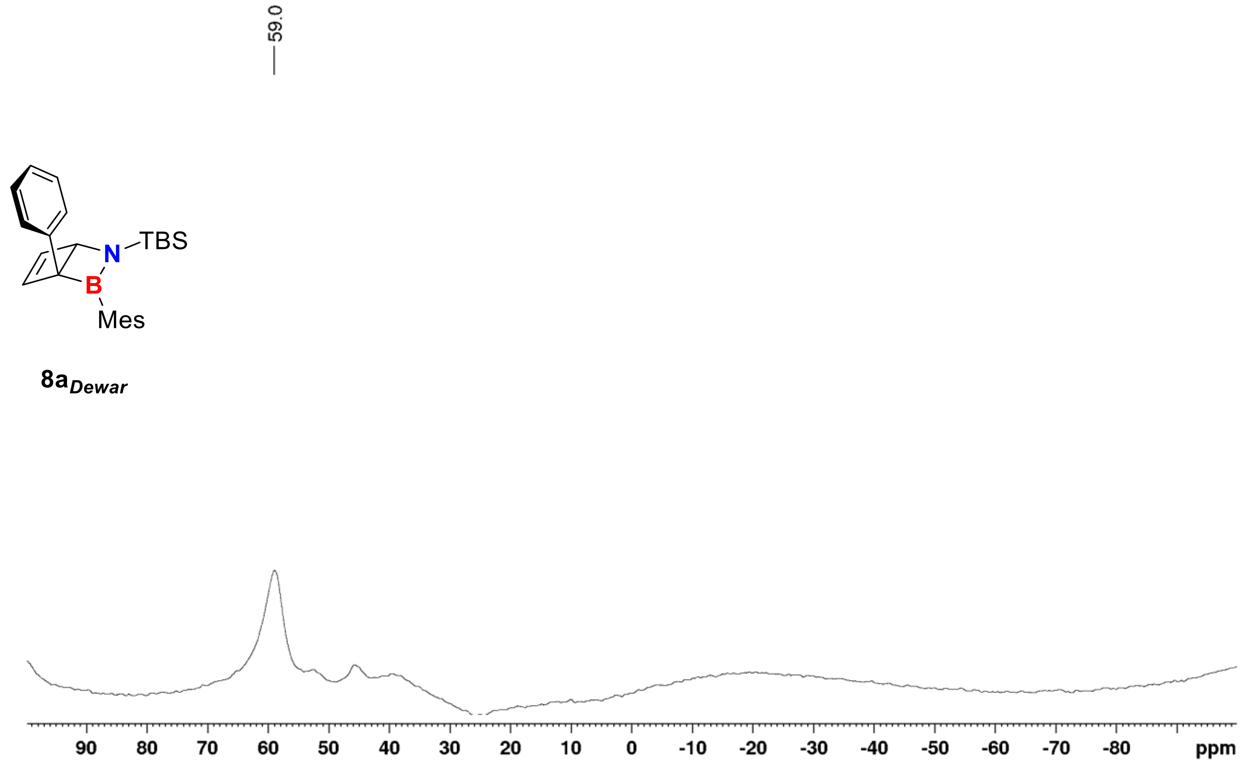

Figure S124. ^{11}B-{^{1}H}-NMR spectrum of compound **8a***Dewar* in C_6D_{12} measured at a 600 MHz spectrometer.

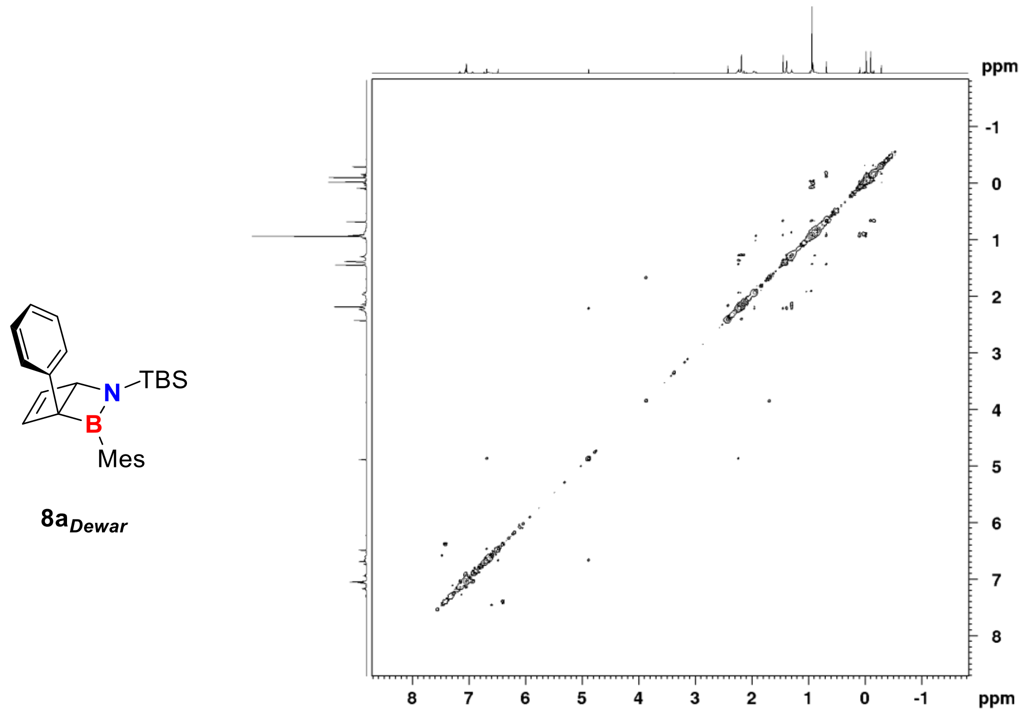

Figure S125. ^{1}H-^{1}H-COSY-NMR spectrum of compound **8a***Dewar* in C_6D_{12} measured at a 600 MHz spectrometer.

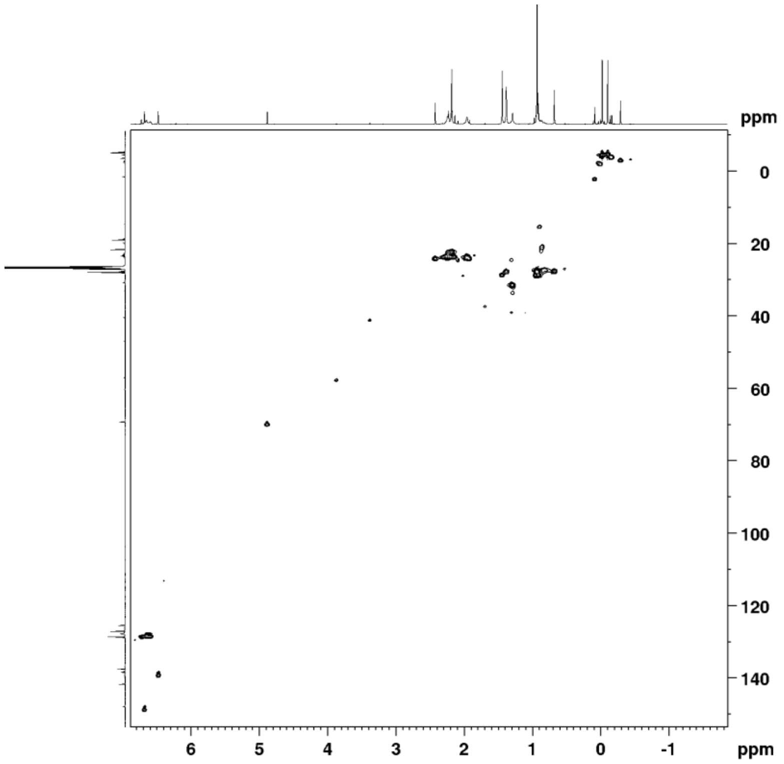

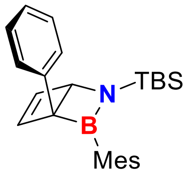

8a*Dewar*

Figure S126. ¹H-¹³C-HSQC-NMR spectrum of compound **8a*Dewar*** in C_6D_{12} measured at a 600 MHz spectrometer.

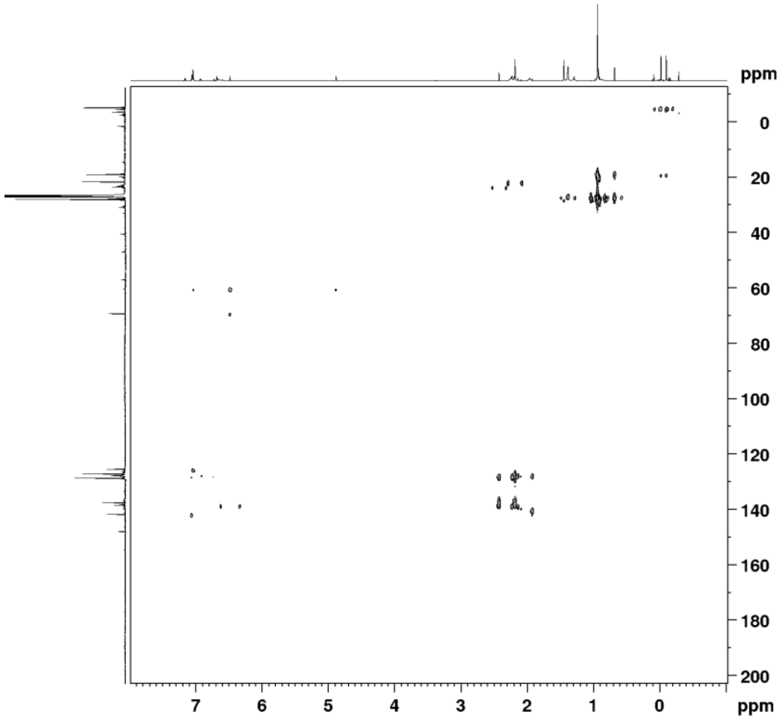

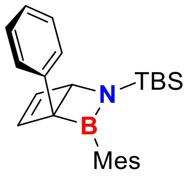

8a*Dewar*

Figure S127. ¹H-¹³C-HMBC-NMR spectrum of compound **8a*Dewar*** in C_6D_{12} measured at a 600 MHz spectrometer.

NMR data of 8b*Dewar*

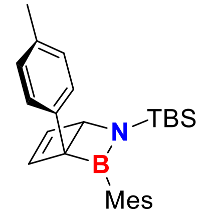

8b*Dewar*

$C_{26}H_{36}BNSi$ (401.48 g/mol)

^{1}H-NMR (600 MHz, **C_6D_{12}**): δ = 6.93 (dm, $^{3}J_{HH}$ = 8.16 Hz, 2H, H-18), 6.88 (dm, $^{3}J_{HH}$ = 8.16 Hz, 2H, H-19), 6.65 (ps. t, 1H, H-5), 6.65 (br. s, 1H, H-14), 6.59 (br. s, 1H, H-14), 6.47 (d, $^{4}J_{HH}$ = 2.40 Hz, 1H, H-4), 2.22 (br. s, 3H, H-13), 2.19 (s, 3H, H-16), 2.18 (s, 3H, H-21), 1.98 (br. s, 3H, H-13), 0.92 (s, 9H, H-10), -0.03 (s, 3H, H-8), -0.11 (s, 3H, H-8) ppm.

^{13}C-{^{1}H}-NMR (151 MHz, **C_6D_{12}**): δ = 148.0 (C5), 138.6 (C4), 138.2 (C12), 137.4 (C12), 134.4 (C15), 129.3 (C19), 128.5 (C11), 127.6 (C14), 127.0 (C18), 112.3 (C5), 69.2 (C6), 59.7 (C3), 26.9 (C10), 23.3 (C21), 22.5 (br. C13),21.5 (br. C13), 21.2 (C16), 21.2 (C21), 18.8 (C9), -5.3 (C8), -5.4 (C8) ppm.

^{11}B-{^{1}H}-NMR (192 MHz, **C_6D_{12}**): δ = 52.9, 39.0 (corresponding dihydroazaborinine) ppm.

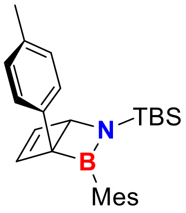

8b*Dewar*

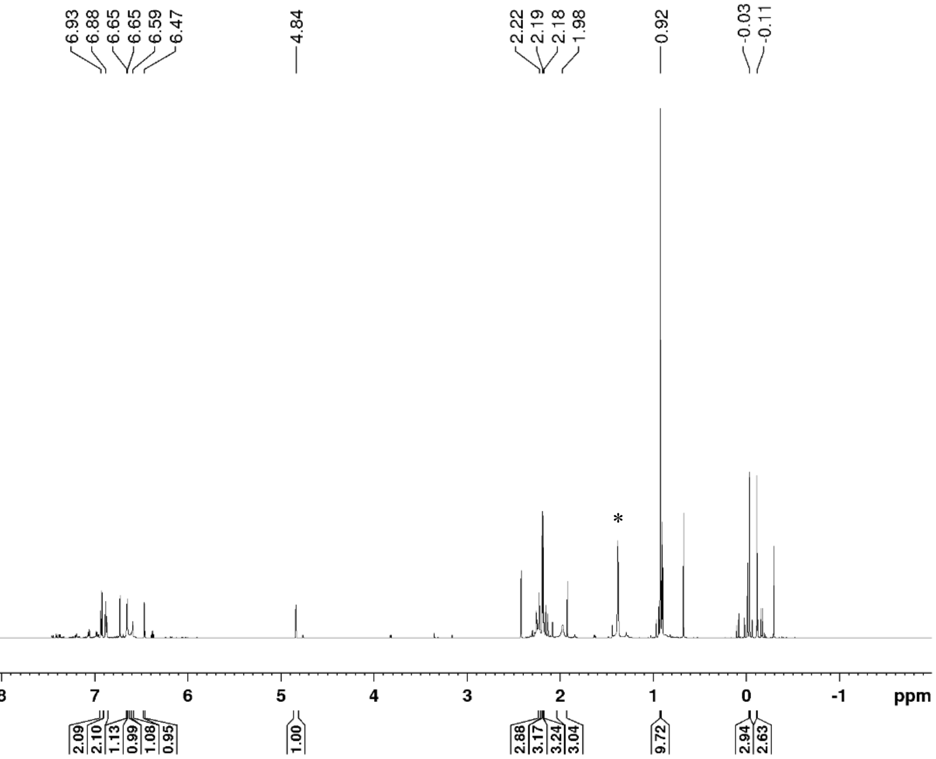

Figure S128. ^{1}H-NMR spectrum of compound **8b*Dewar*** in C6D12 measured at a 600 MHz spectrometer. The solvent signal is marked with an asterisk.

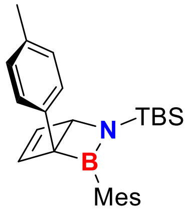

8b*Dewar*

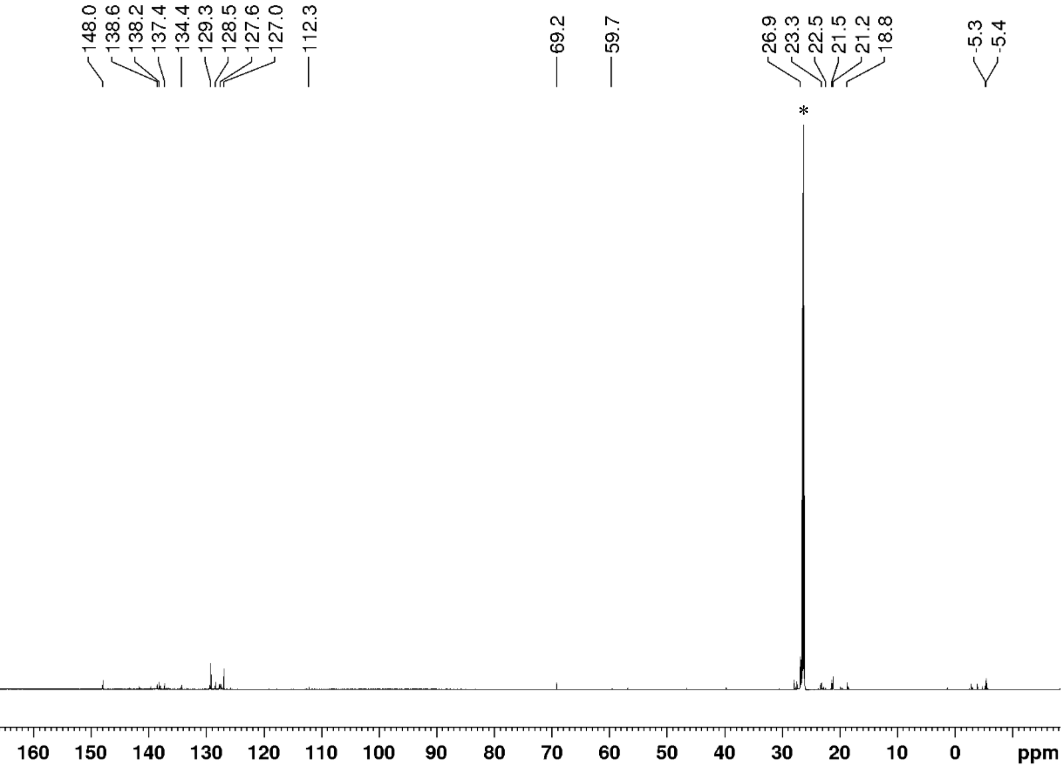

Figure S129. ^{13}C-{^{1}H}-NMR spectrum of compound **8b*Dewar*** in C_6D_{12} measured at a 600 MHz spectrometer. The solvent signal is marked with an asterisk.

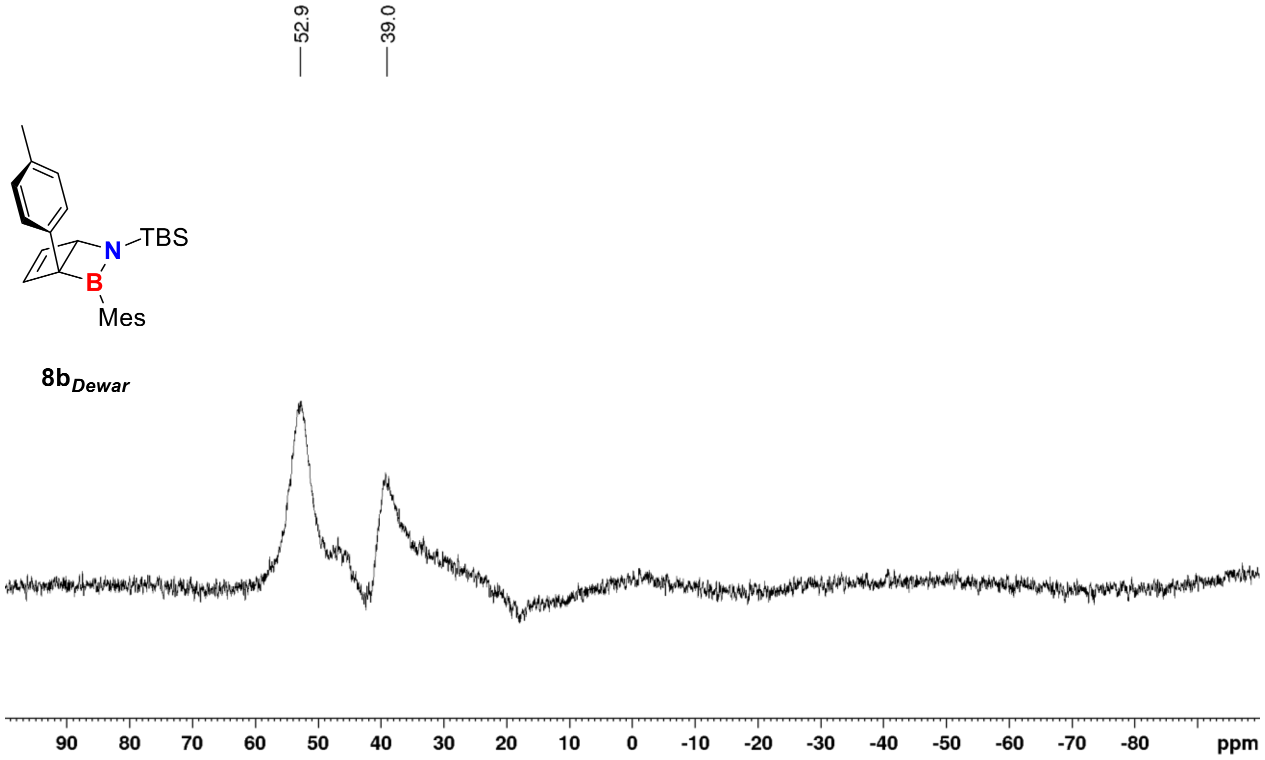

8b$_{Dewar}$

Figure S130. ^{11}B-{^{1}H}-NMR spectrum of compound **8b$_{Dewar}$** in C_6D_{12} measured at a 600 MHz spectrometer.

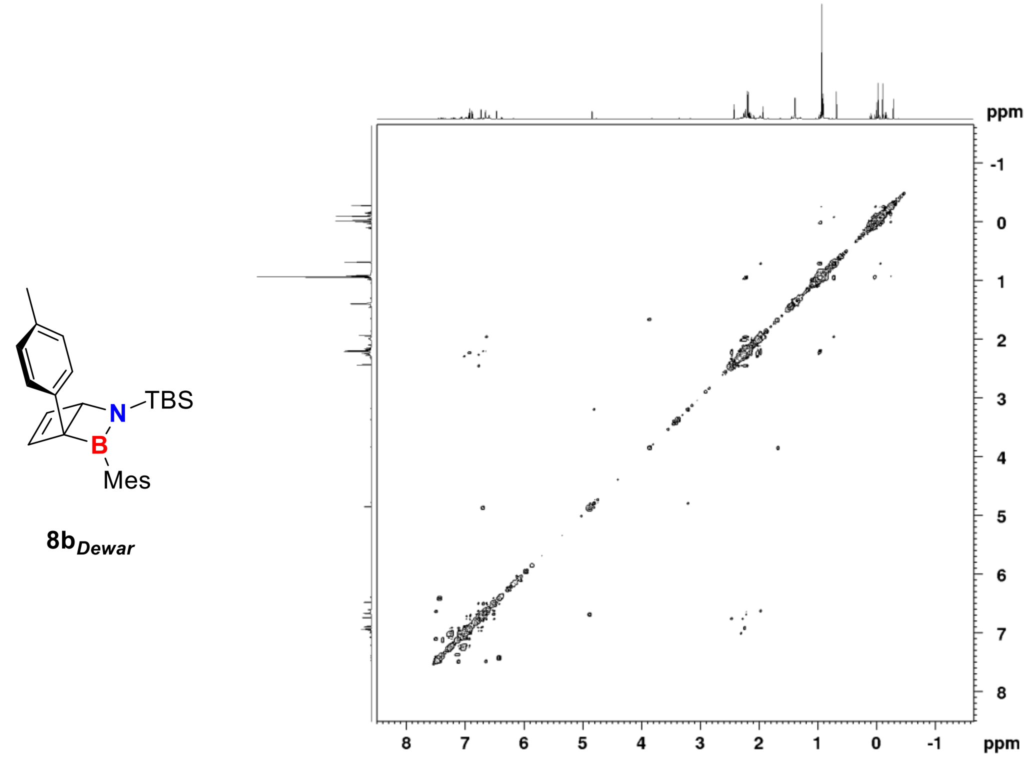

8b$_{Dewar}$

Figure S131. ^{1}H-^{1}H-COSY-NMR spectrum of compound **8b$_{Dewar}$** in C_6D_{12} measured at a 600 MHz spectrometer.

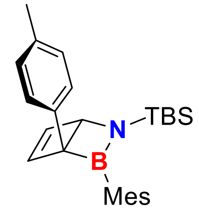

8b*Dewar*

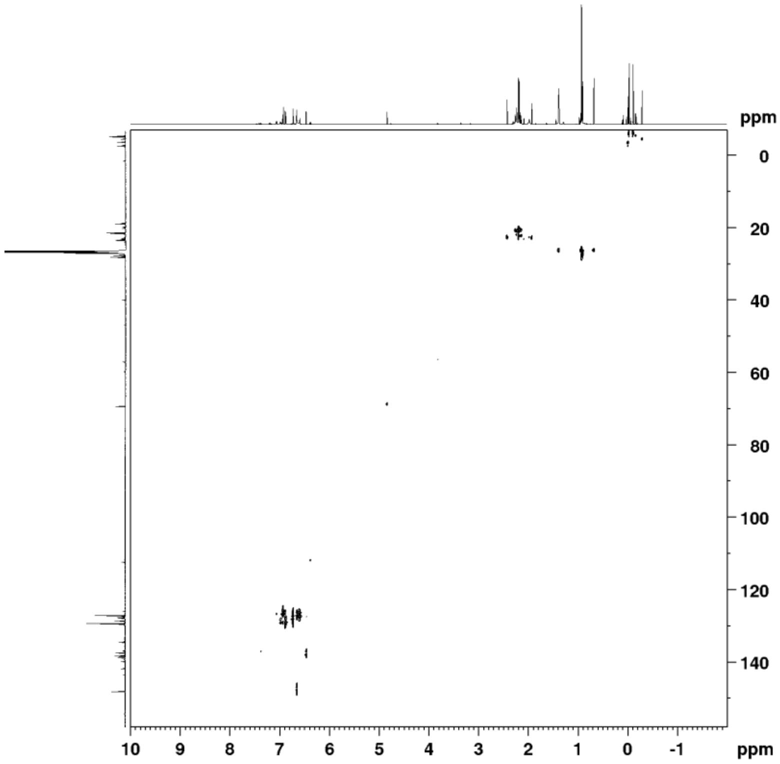

Figure S132. ^{1}H-^{13}C-HSQC-NMR spectrum of compound **8b*Dewar*** in C_6D_{12} measured at a 600 MHz spectrometer.

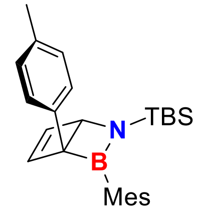

8b*Dewar*

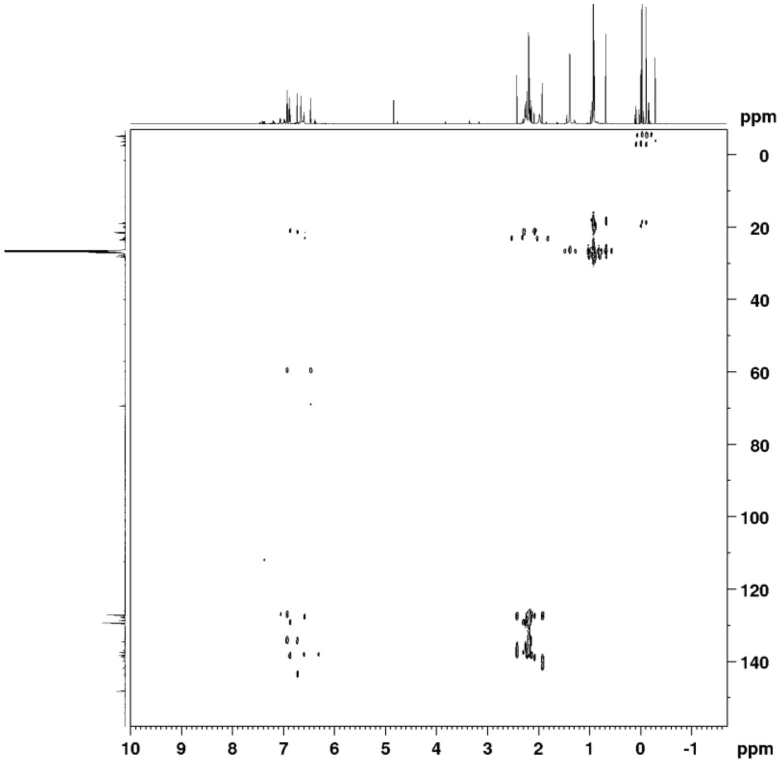

Figure S133. ^{1}H-^{13}C-HMBC-NMR spectrum of compound **8b*Dewar*** in C_6D_{12} measured at a 600 MHz spectrometer.

NMR data of 8c*Dewar*

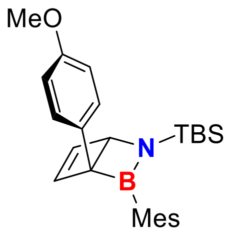

8c*Dewar*

$C_{26}H_{36}BNOSi$ (417.48 g/mol)

^{1}H-NMR (600 MHz, **C_6D_{12}**): δ = 6.95 (dm, $^3J_{HH}$ = 8.16 Hz, 2H, H-19), 6.66 (br. s, 1H, H-14), 6.62 (m, 4H, H-5/H-14/H-18), 6.47 (d, dm, $^4J_{HH}$ = 2.41 Hz, 1H, H-4), 4.79 (d, $^4J_{HH}$ = 2.41 Hz, 1H, H-6), 3.60 (s, 3H, H-22), 2.23 (br. s, 3H, H-13), 2.19 (s, 3H, H-16), 2.01 (br. s, 3H, H-13), 0.92 (s, 9H, H-10), -0.04 (s, 3H, H-8), -0.12 (s, 3H, H-8) ppm.

^{13}C-{^{1}H}-NMR (151 MHz, **C_6D_{12}**): δ = 158.4 (C17), 148.2 (C5), 139.3 (C12), 138.2 (C12), 137.4 (C4), 133.4 (C11), 129.4 (C15), 128.1 (C20), 127.7 (C19), 127.6 (br. C14), 114.0 (C18), 69.5 (C6), 58.9 (C3), 54.7 (C22), 28.0 (C10), 23.3 (C13), 22.5 (C13), 21.5 (C16), 18.8 (C9), -5.2 (C8), -5.4 (C8) ppm.

^{11}B-{^{1}H}-NMR (192 MHz, **C_6D_{12}**): δ = 53.0 ppm.

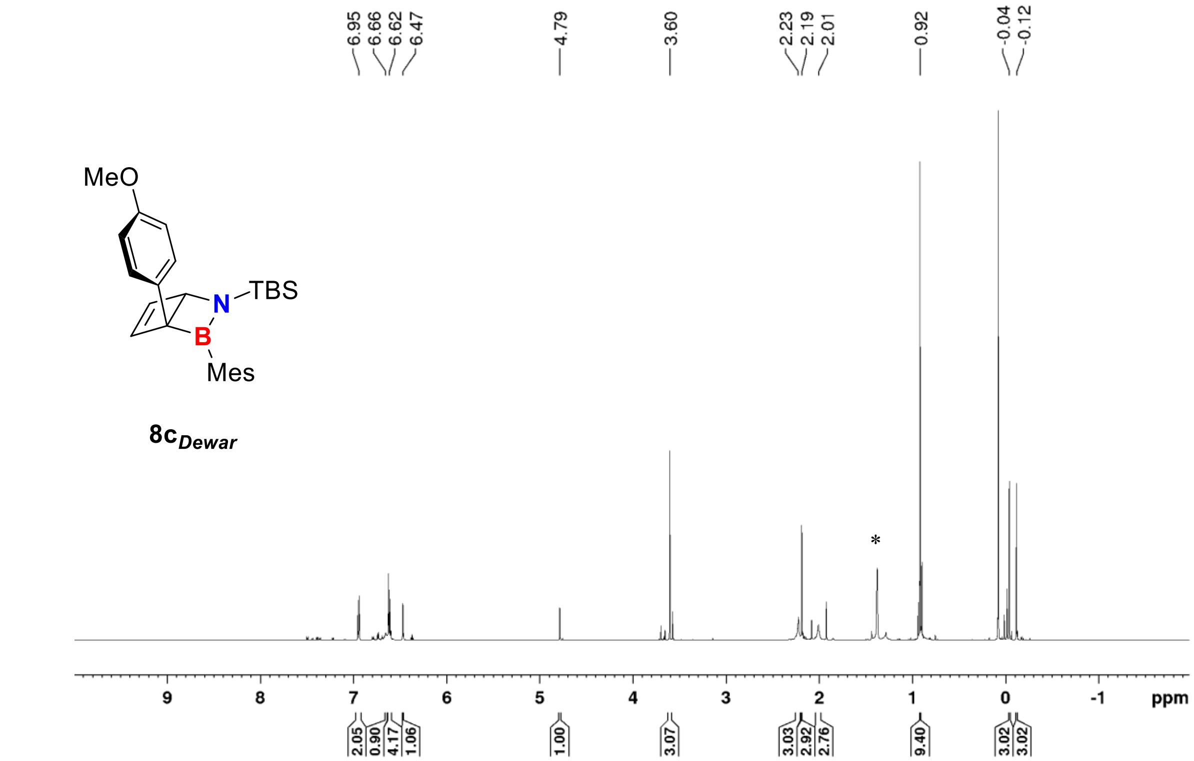

Figure S134. ^{1}H-NMR spectrum of compound **8c$_{Dewar}$** in C6D12 measured at a 600 MHz spectrometer. The solvent signal is marked with an asterisk.

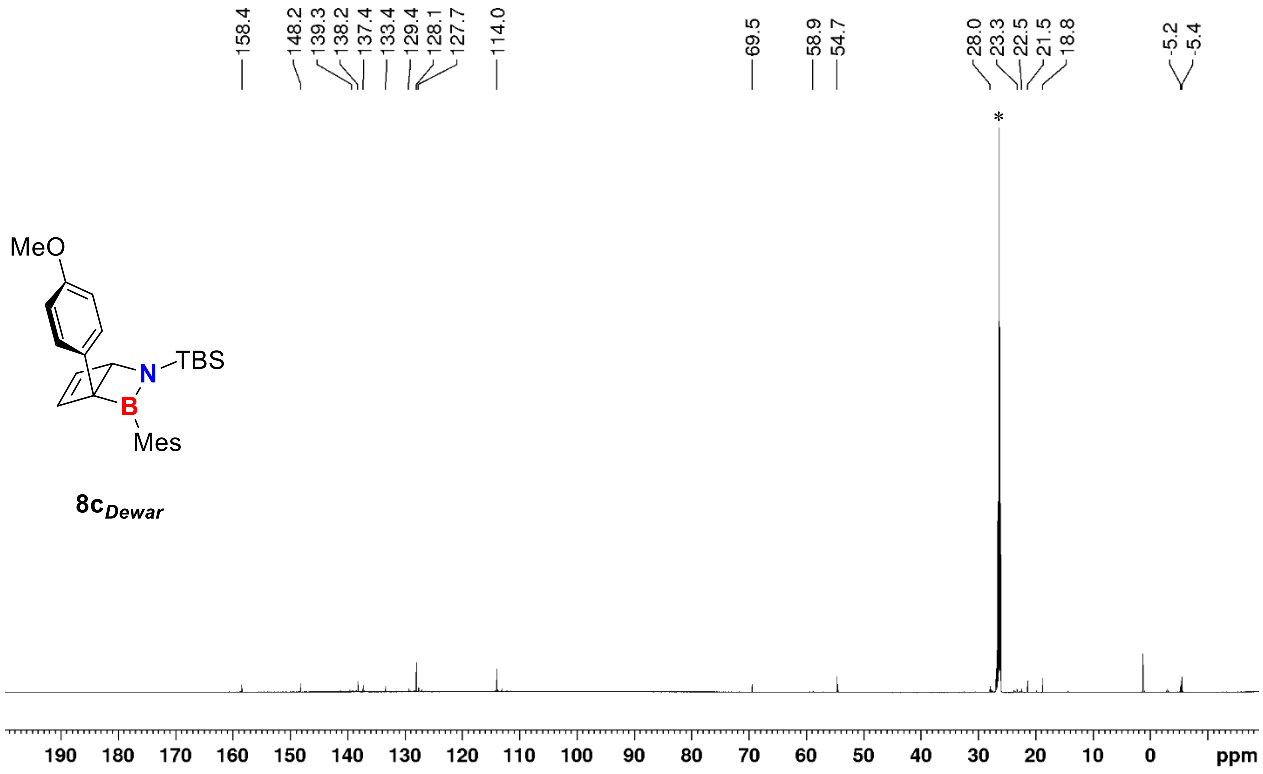

Figure S135. ^{13}C-{^{1}H}-NMR spectrum of compound **8c$_{Dewar}$** in C_6D_{12} measured at a 600 MHz spectrometer. The solvent signal is marked with an asterisk.

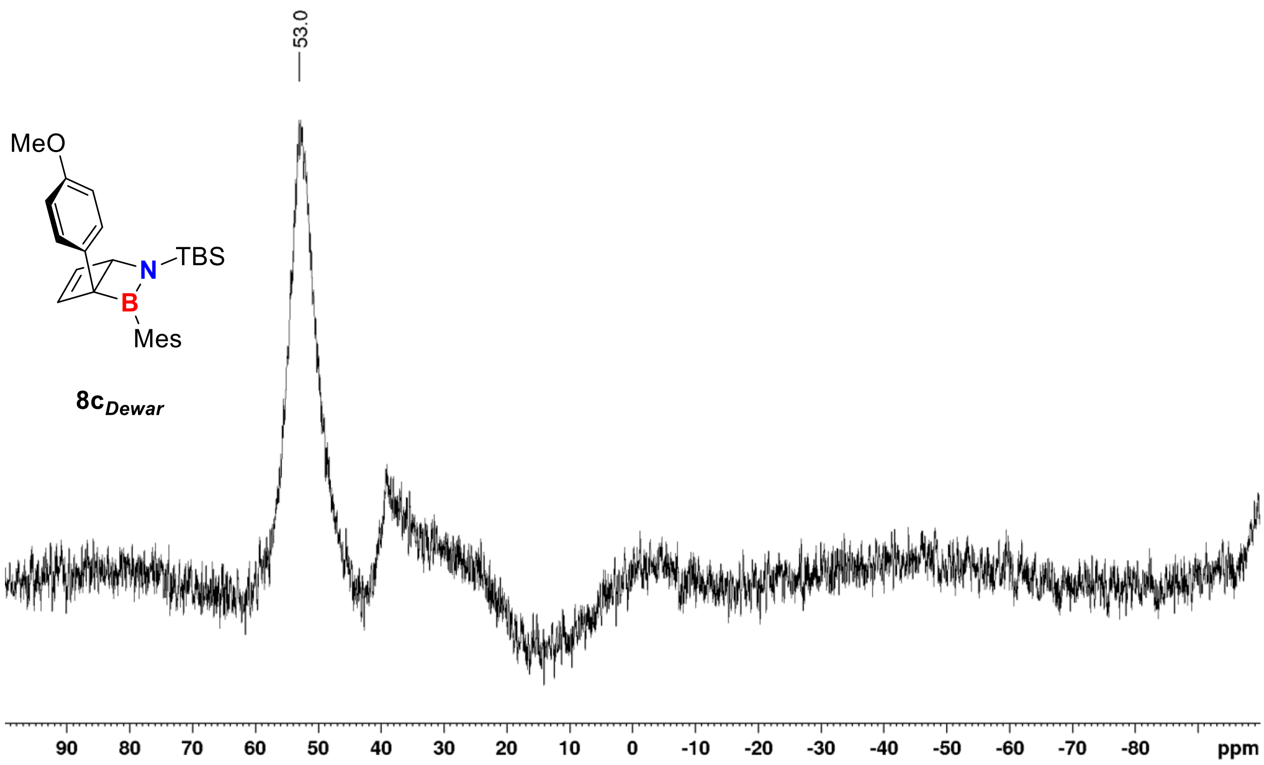

Figure S136. ^{11}B-{^{1}H}-NMR spectrum of compound **8c*Dewar*** in C_6D_{12} measured at a 600 MHz spectrometer.

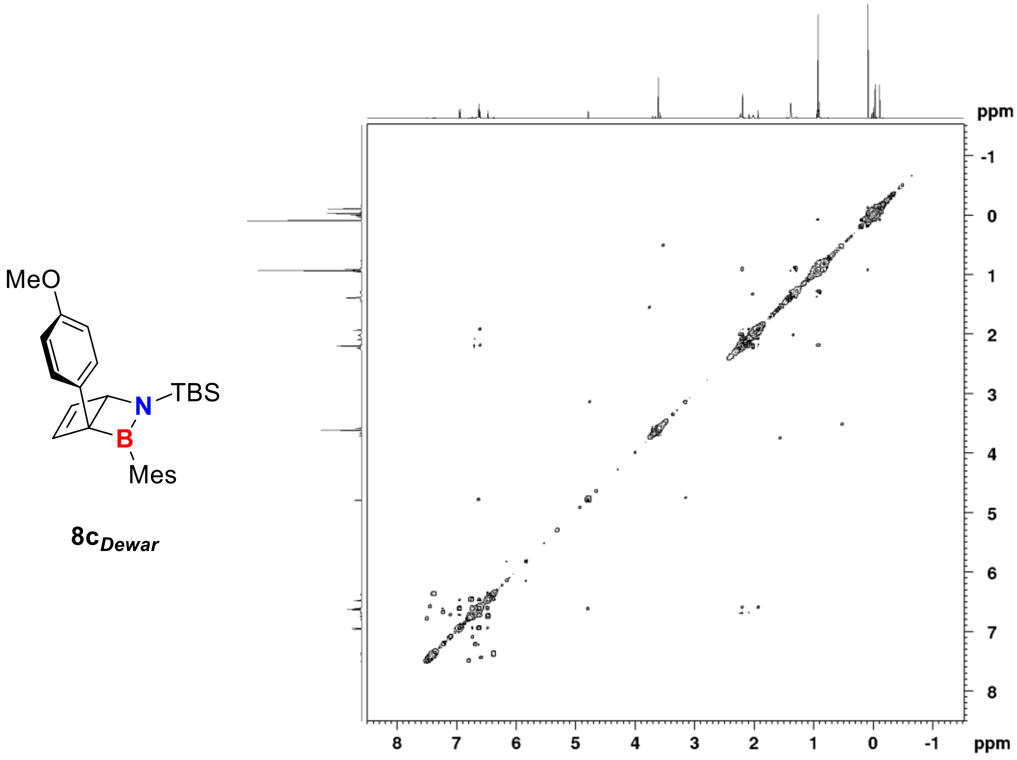

Figure S137. ^{1}H-^{1}H-COSY-NMR spectrum of compound **8c*Dewar*** in C_6D_{12} measured at a 600 MHz spectrometer.

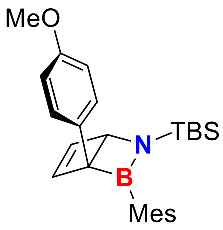

8c*Dewar*

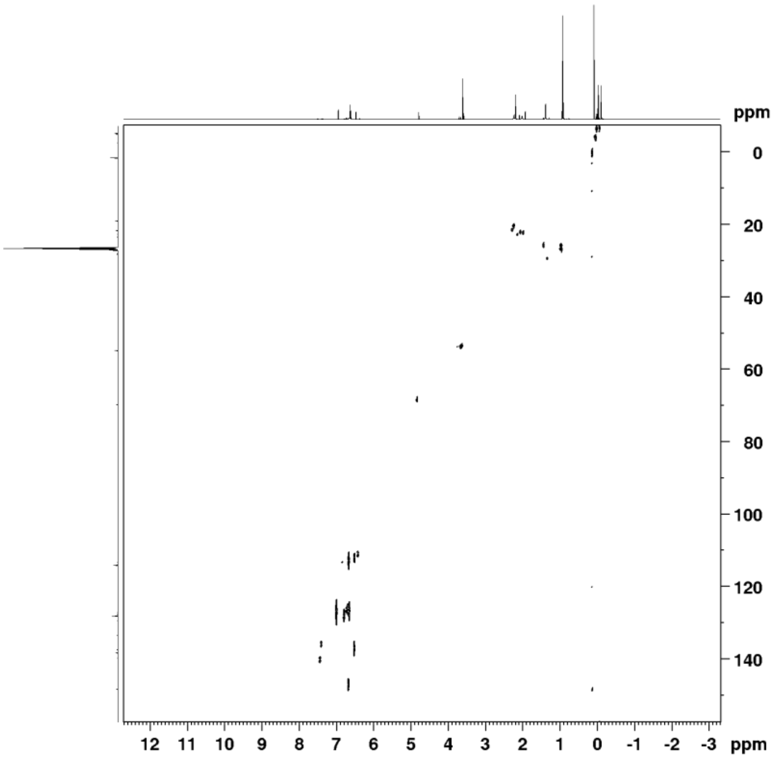

Figure S138. ^{1}H-^{13}C-HSQC-NMR spectrum of compound **8c*Dewar*** in C_6D_{12} measured at a 600 MHz spectrometer.

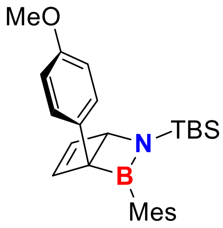

8c*Dewar*

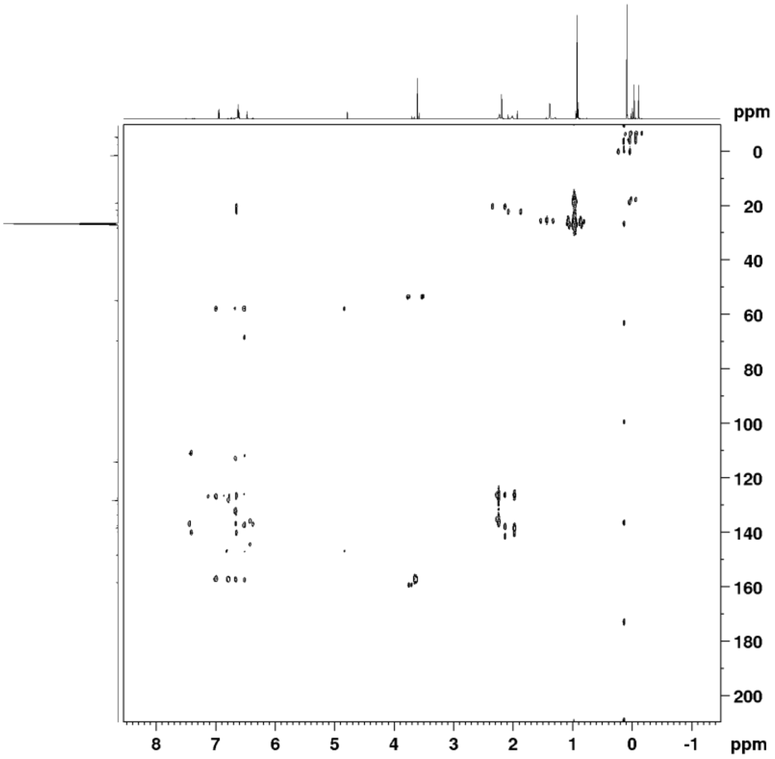

Figure S139. ^{1}H-^{13}C-HMBC-NMR spectrum of compound **8c*Dewar*** in C_6D_{12} measured at a 600 MHz spectrometer.

NMR data of 8e*Dewar*

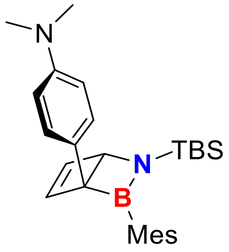

8e*Dewar*

$C_{28h39}BN_2Si$ (430.52 g/mol)

^{1}H-NMR (700 MHz, **C_6D_{12}**): δ = 6.91 (dm, $^3J_{HH}$ = 8.95 Hz, 2H, H-18), 6.65 (br. s, 1H, H-14), 6.63 (br. s, 1H, H-14), 6.61 (ps. t, 1H, H-5), 6.49 (dm, $^3J_{HH}$ = 8.95 Hz, 2H, H-19), 6.46 (d, $^4J_{HH}$ = 2.35 Hz, 1H, H-4), 4.74 (d, $^4J_{HH}$ = 2.35 Hz, 1H, H-6), 2.77 (s, 6H, H-22), 2.23 (br. s, 3H, H-13), 2.19 (s, 3H, H-16), 2.06 (br. s, 3H, H-13), 0.92 (s, 9H, H-10), -0.04 (s, 3H, H-8), -0.12 (s, 3H, H-8) ppm.

^{13}C-{^{1}H}-NMR (176 MHz, **C_6D_{12}**): δ = 148.4 (C17), 147.5 (C5), 140.8 (C11), 138.5 (C12), 137.6 (C12), 137.1 (C4), 135.6 (C15), 128.2 (C14), 127.1 (C18), 126.6 (C14), 112.7 (C19), 111.7 (C20), 68.8 (C6) 57.9 (C3), 39.9 (C22), 27.1 (C10), 22.5 (C13), 21.6 (C13), 20.6 (C16), 17.9 (C9), -6.1 (C8), -6.3 (C8) ppm.

^{11}B-{^{1}H}-NMR (128 MHz, **C_6D_{12}**): δ = 48.4 ppm.

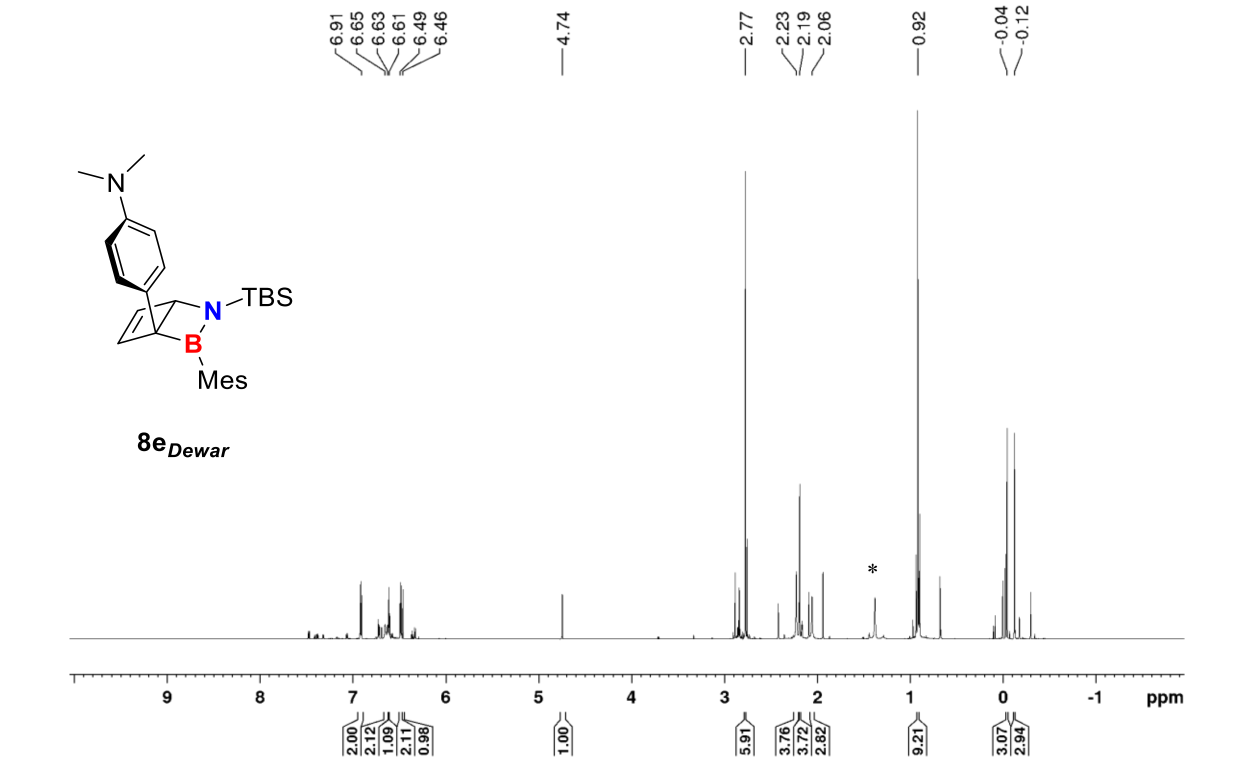

Figure S140. ^{1}H-NMR spectrum of compound **8e*Dewar*** in C6D12 measured at a 700 MHz spectrometer. The solvent signal is marked with an asterisk.

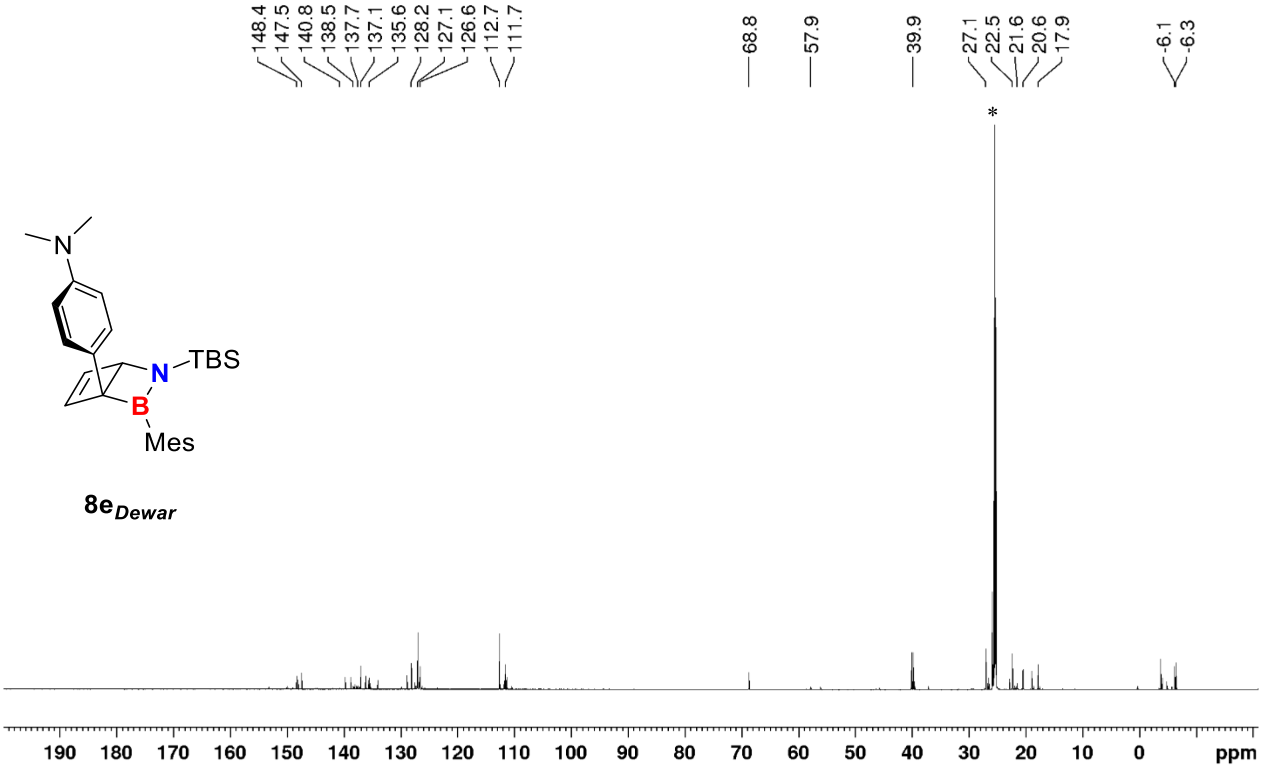

Figure S141. ^{13}C-{^{1}H}-NMR spectrum of compound **8e*Dewar*** in C_6D_{12} measured at a 700 MHz spectrometer. The solvent signal is marked with an asterisk.

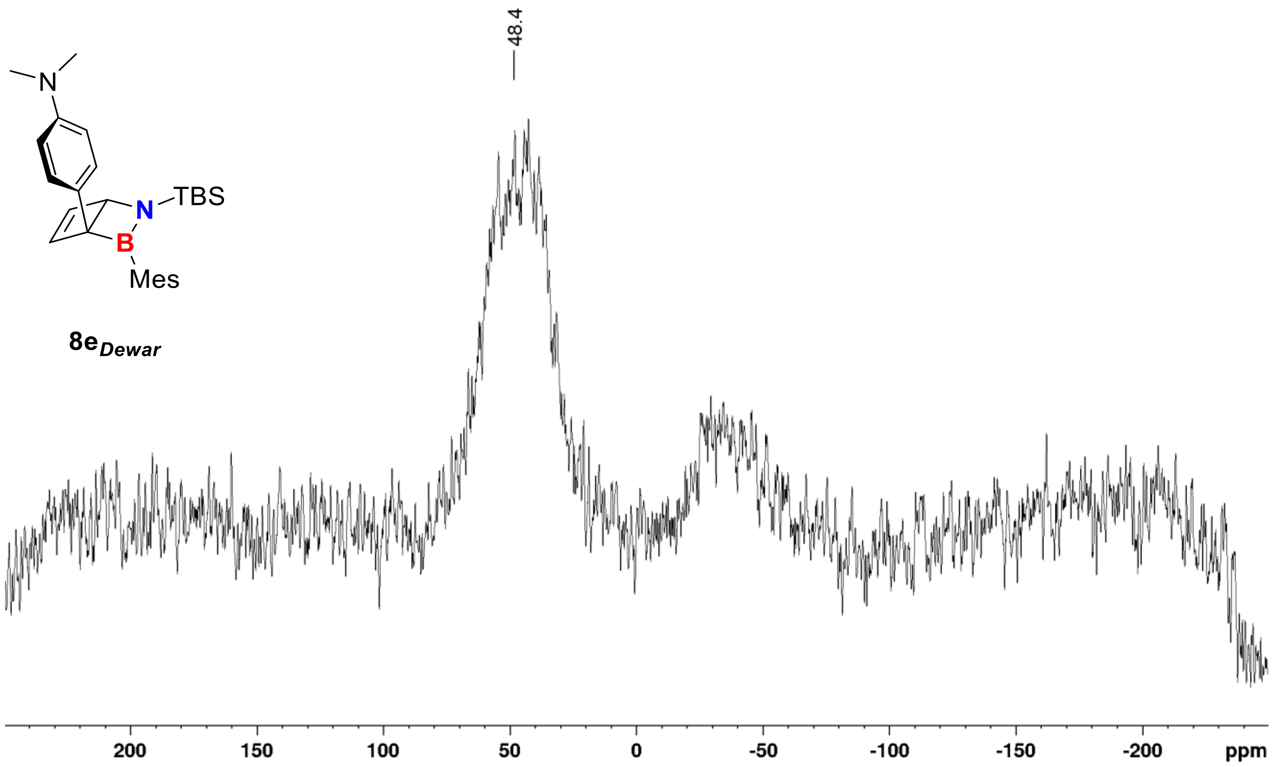

Figure S142. ^{11}B-{^{1}H}-NMR spectrum of compound **8e*Dewar*** in C_6D_{12} measured at a 400 MHz spectrometer.

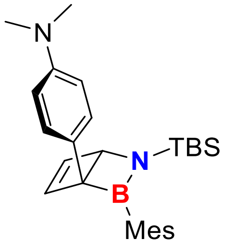

8e*Dewar*

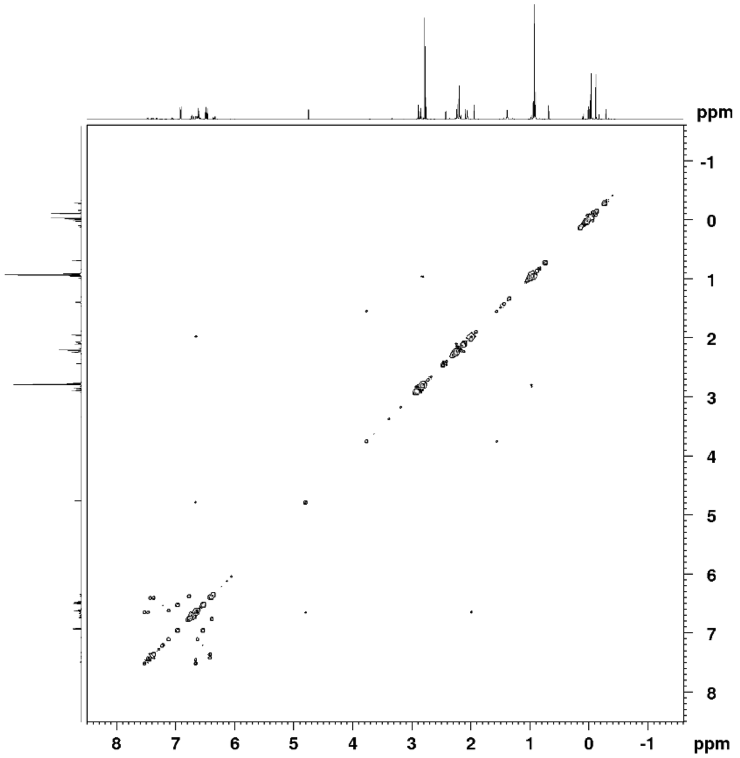

Figure S143. ^{1}H-^{1}H-COSY-NMR spectrum of compound **8e*Dewar*** in C_6D_{12} measured at a 700 MHz spectrometer.

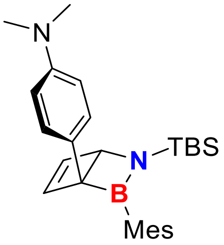

8e*Dewar*

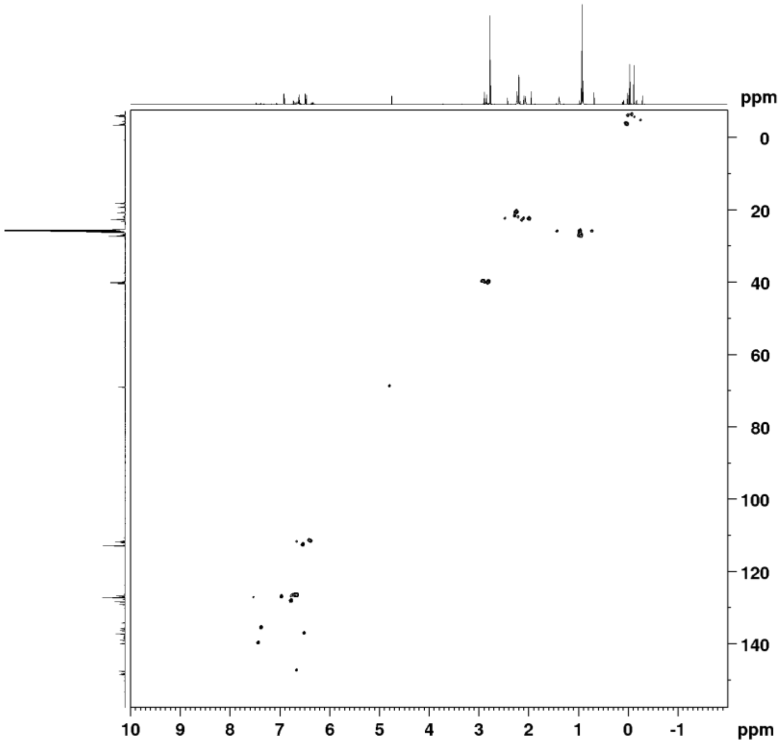

Figure S144. ^{1}H-^{13}C-HSQC-NMR spectrum of compound **8e*Dewar*** in C_6D_{12} measured at a 700 MHz spectrometer.

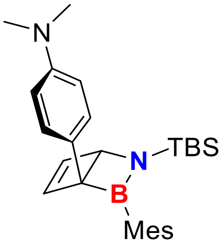

8e*Dewar*

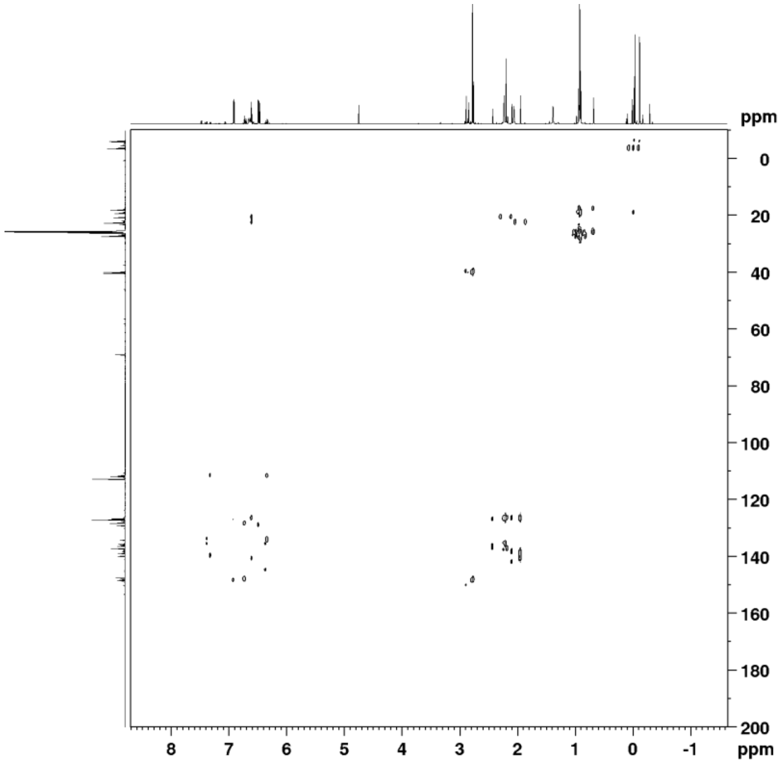

Figure S145. ^{1}H-^{13}C-HMBC-NMR spectrum of compound **8e*Dewar*** in C_6D_{12} measured at a 700 MHz spectrometer.

NMR data of 8f*Dewar*

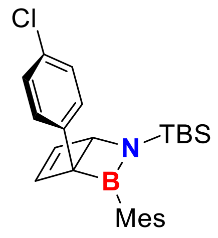

8f*Dewar*

$C_{25}H_{33}BClNSi$ (421.89 g/mol)

^{1}H-NMR (600 MHz, **C_6D_{12}**): δ = 7.04 (dm, $^{3}J_{HH}$ = 8.30 Hz, 2H, H-18), 6.97 (dm, $^{3}J_{HH}$ = 8.30 Hz, 2H, H-19), 6.64 (m, 3H, H-5/H-14), 6.49 (m, 1H, H-4), 4.84 (m, 1H, H-6), 2.23 (br. s, 3H, H-13), 2.19 (s, 3H, H-16), 1.97 (br. s, 3H, H-13), 0.92 (s, 9H, H-10), -0.03 (s, 3H, H-8), -0.10 (s, 3H, H-8) ppm.

^{13}C-{^{1}H}-NMR (151 MHz, **C_6D_{12}**): δ = 147.5 (C5), 142.0 (C20), 140.1 (C12), 139.3 (C11), 138.7 (C4), 138.5 (C3), 137.7 (C17), 131.9 (C15), 129.7 (C15), 128.8 (C19), 128.3 (C18), 127.7 (C14), 69.1 (C6), 59.2 (C3), 27.9 (C10), 23.2 (C13), 22.5 (C13), 21.4 (C16), 18.7 (C9), -5.3 (C8), -5.5 (C8) ppm.

^{11}B-{^{1}H}-NMR (192 MHz, **C_6D_{12}**): δ = 52.4, 39.0 (corresponding dihydroazaborinine)

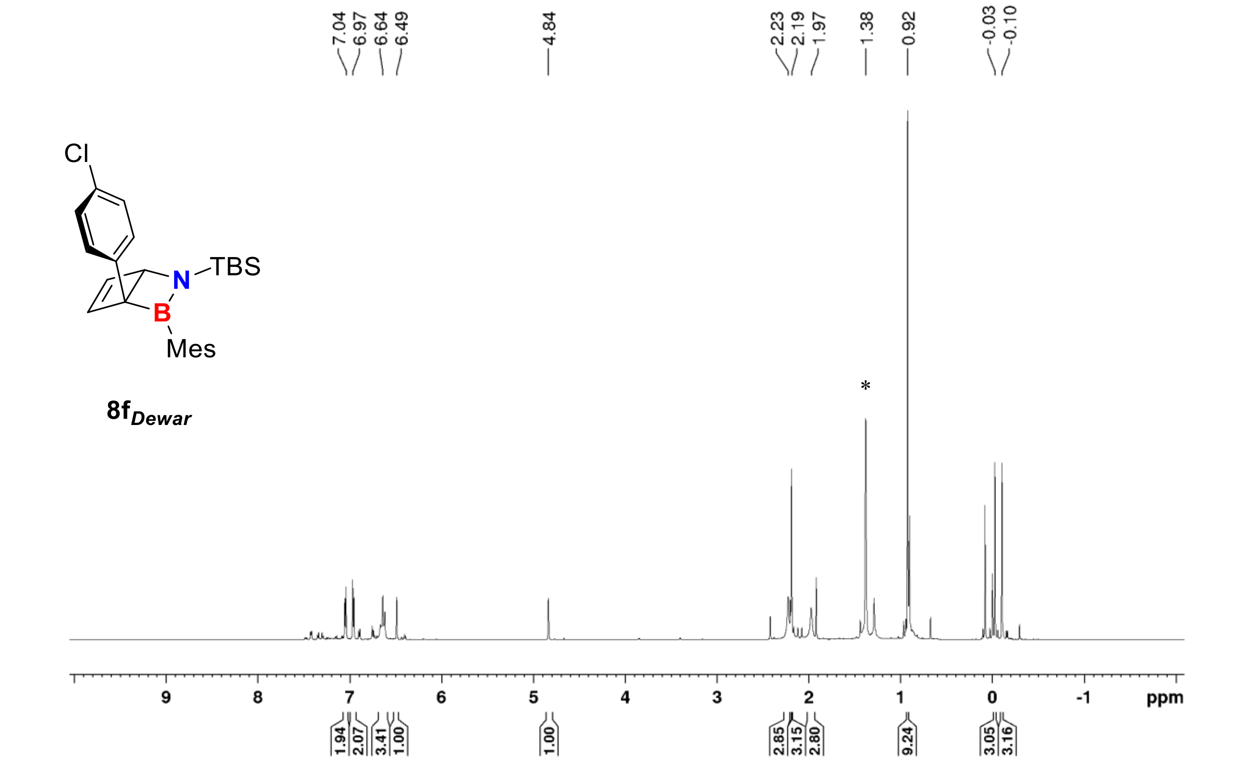

Figure S146. ^{1}H-NMR spectrum of compound **8f**$_{Dewar}$ in C6D12 measured at a 600 MHz spectrometer. The solvent signal is marked with an asterisk.

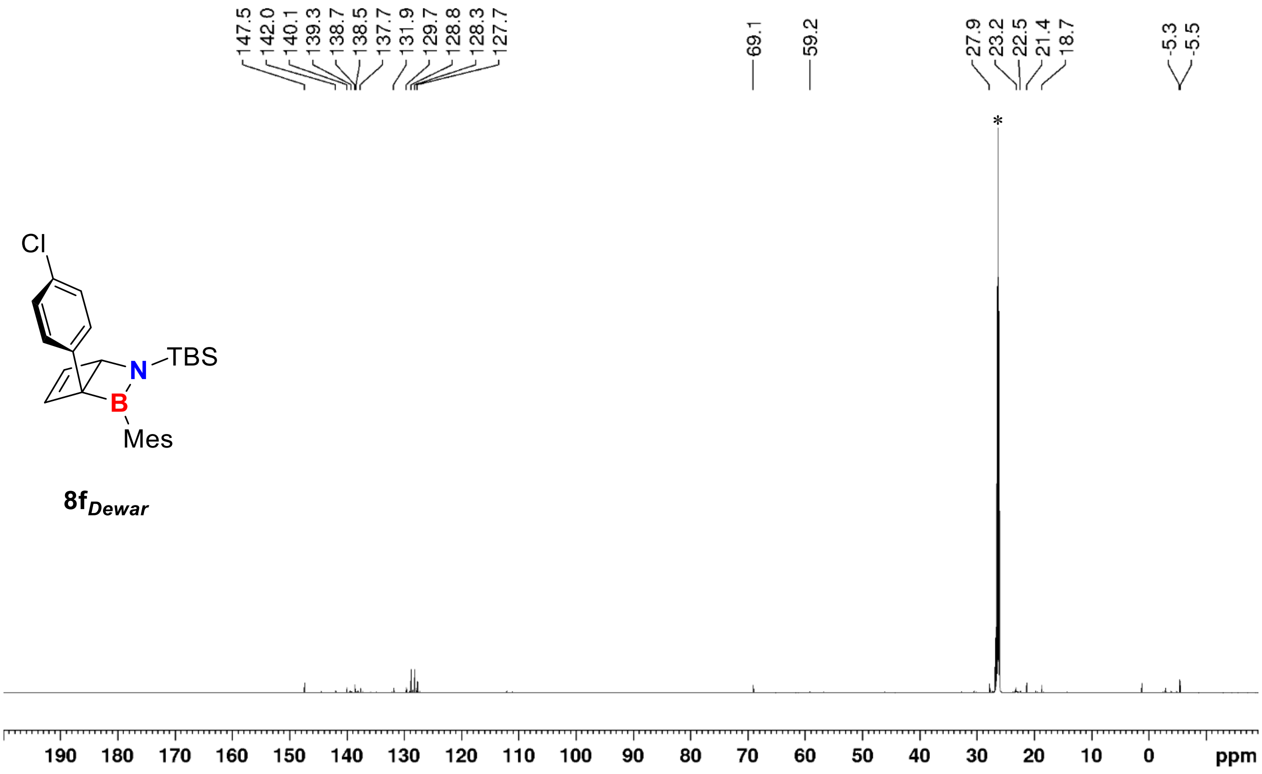

Figure S147. ^{13}C-{^{1}H}-NMR spectrum of compound **8f**$_{Dewar}$ in C_6D_{12} measured at a 600 MHz spectrometer. The solvent signal is marked with an asterisk.

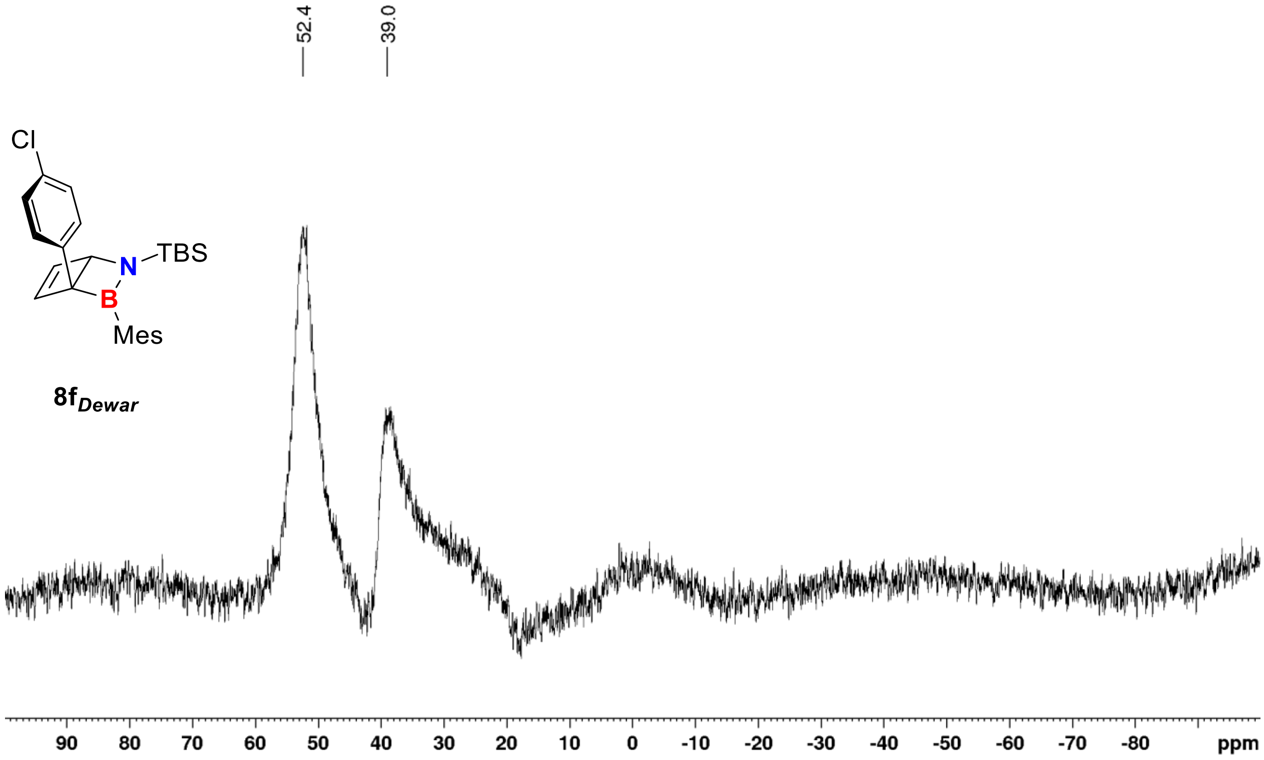

Figure S148. ^{11}B-{^{1}H}-NMR spectrum of compound **8f$_{Dewar}$** in C_6D_{12} measured at a 600 MHz spectrometer.

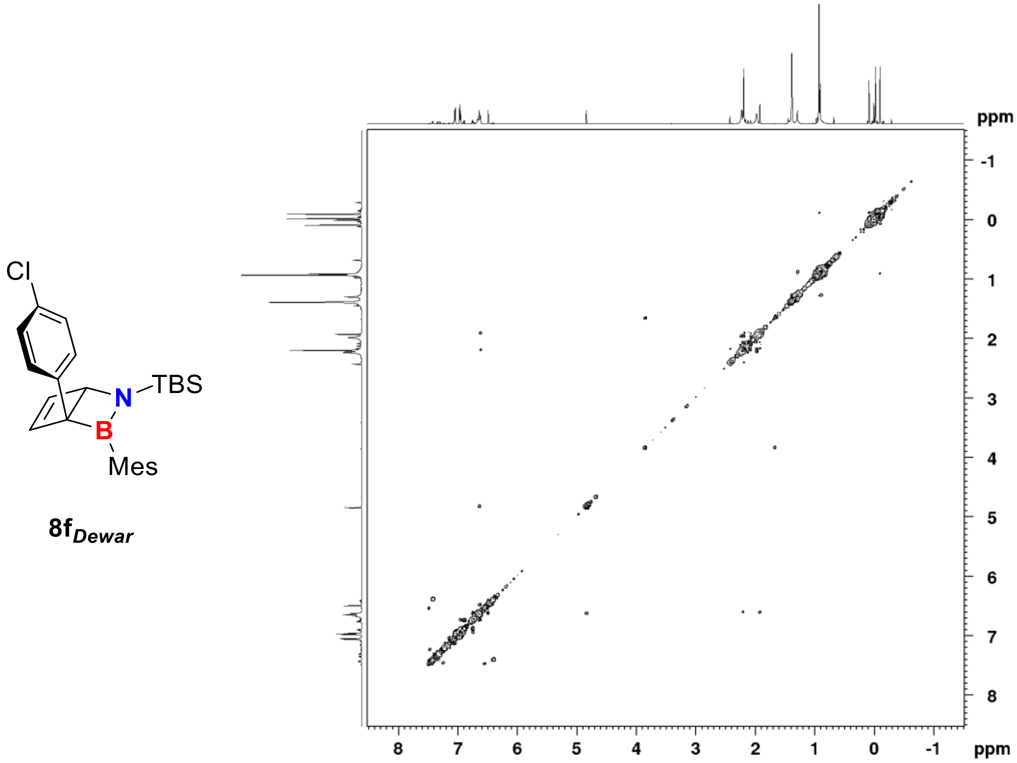

Figure S149. ^{1}H-^{1}H-COSY-NMR spectrum of compound **8f$_{Dewar}$** in C_6D_{12} measured at a 600 MHz spectrometer.

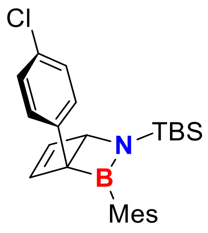

8f*Dewar*

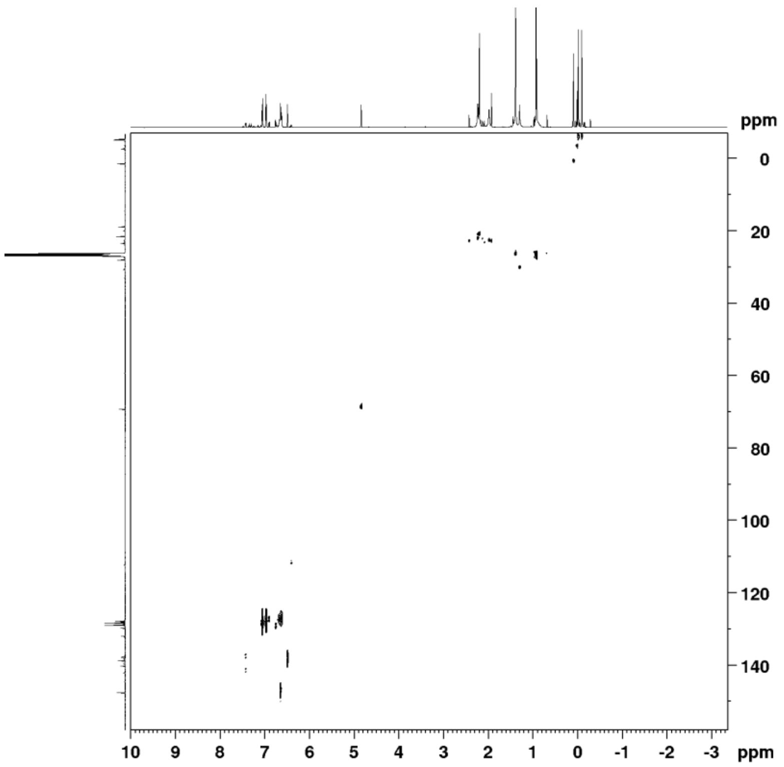

Figure S150. ^{1}H-^{13}C-HSQC-NMR spectrum of compound **8f***Dewar* in C_6D_{12} measured at a 600 MHz spectrometer.

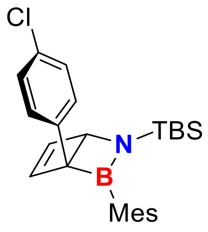

8f*Dewar*

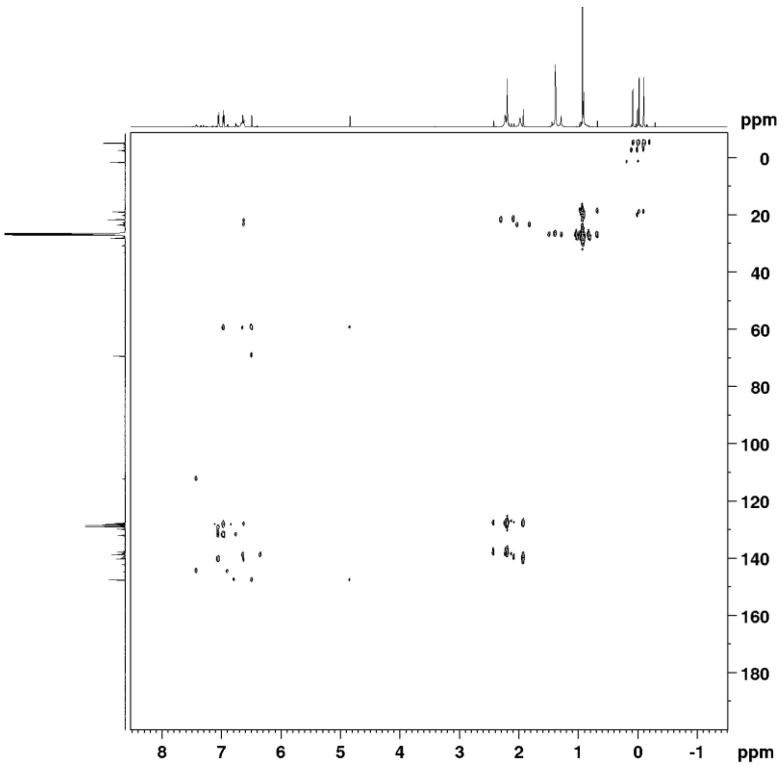

Figure S151. ^{1}H-^{13}C-HMBC-NMR spectrum of compound **8f***Dewar* in C_6D_{12} measured at a 600 MHz spectrometer.

NMR data of 8g*Dewar*

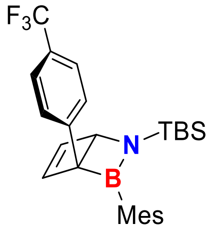

8g*Dewar*

$C_{26}H_{33}BF_3NSi$ (455.45 g/mol)

^{1}H-NMR (700 MHz, **C_6D_{12}**): δ = 7.36 (d, $^3J_{HH}$ = 8.41 Hz, 2H, H-18),7.15 (d, $^3J_{HH}$ = 8.41 Hz, 2H, H-19), 6.69 (ps. t, 1H, H-5), 6.68 (br. s, 1H, H-14), 6.62 (br. s, 1H, H-14), 6.52 (d, $^4J_{HH}$ = 2.36 Hz, 1H, H-4), 4.92 (d, $^4J_{HH}$ = 2.36 Hz, 1H, H-6), 2.24 (br. s, 3H, H-13), 2.19 (s, 3H, H-16), 1.97 (br. s, 3H, H-13), 0.94 (s, 9H, H-10), -0.01 (s, 3H, H-8), -0.09 (s, 3H, H-8) ppm.

^{13}C-{^{1}H}-NMR (176 MHz, **C_6D_{12}**): δ = 147.0 (C5), 146.1 (C17), 139.3 (C12), 139.0 (C4), 138.5 (C15), 137.9 (C20), 135.7(C11), 127.8 (C14), 127.1 (C19), 125.6 (C18), 125.6 (C18), 69.1 (C6), 59.8 (C3), 26.8 (C10), 23.3 (C13), 22.6 (C13), 21.5 (C16), 18.8 (C9), -5.3 (C8), -5.5 (C8) ppm.

^{11}B-{^{1}H}-NMR (128 MHz, **C_6D_{12}**): δ = 53.4 ppm.

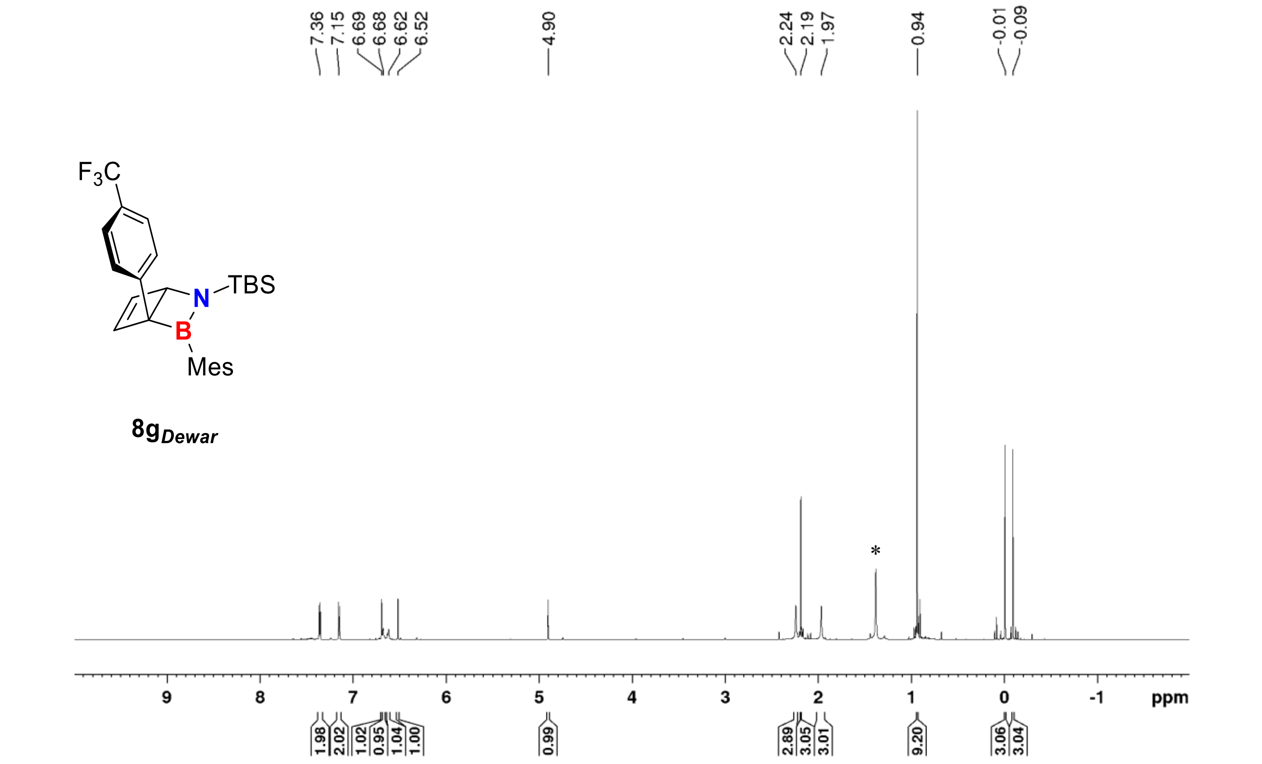

Figure S152. ^{1}H-NMR spectrum of compound **8g*Dewar*** in C6D12 measured at a 700 MHz spectrometer. The solvent signal is marked with an asterisk.

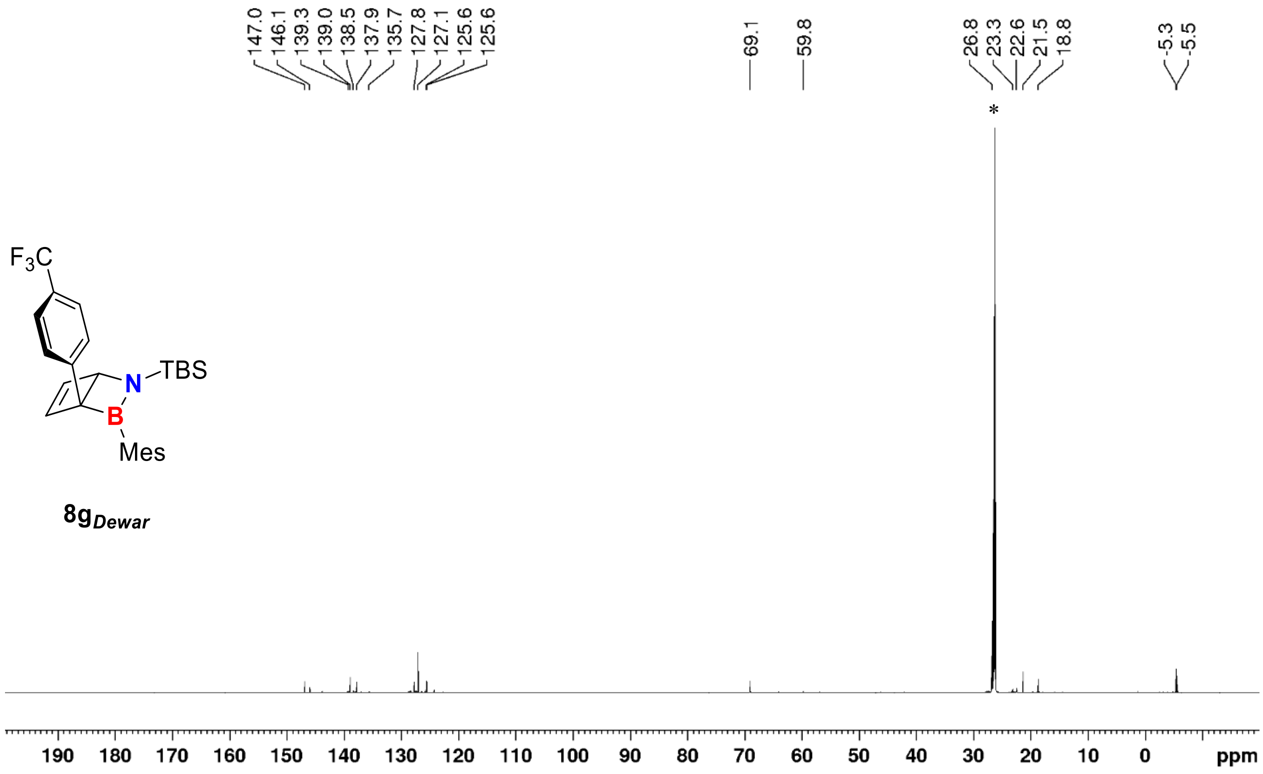

Figure S153. ^{13}C-{^{1}H}-NMR spectrum of compound **8g*Dewar*** in C_6D_{12} measured at a 700 MHz spectrometer. The solvent signal is marked with an asterisk.

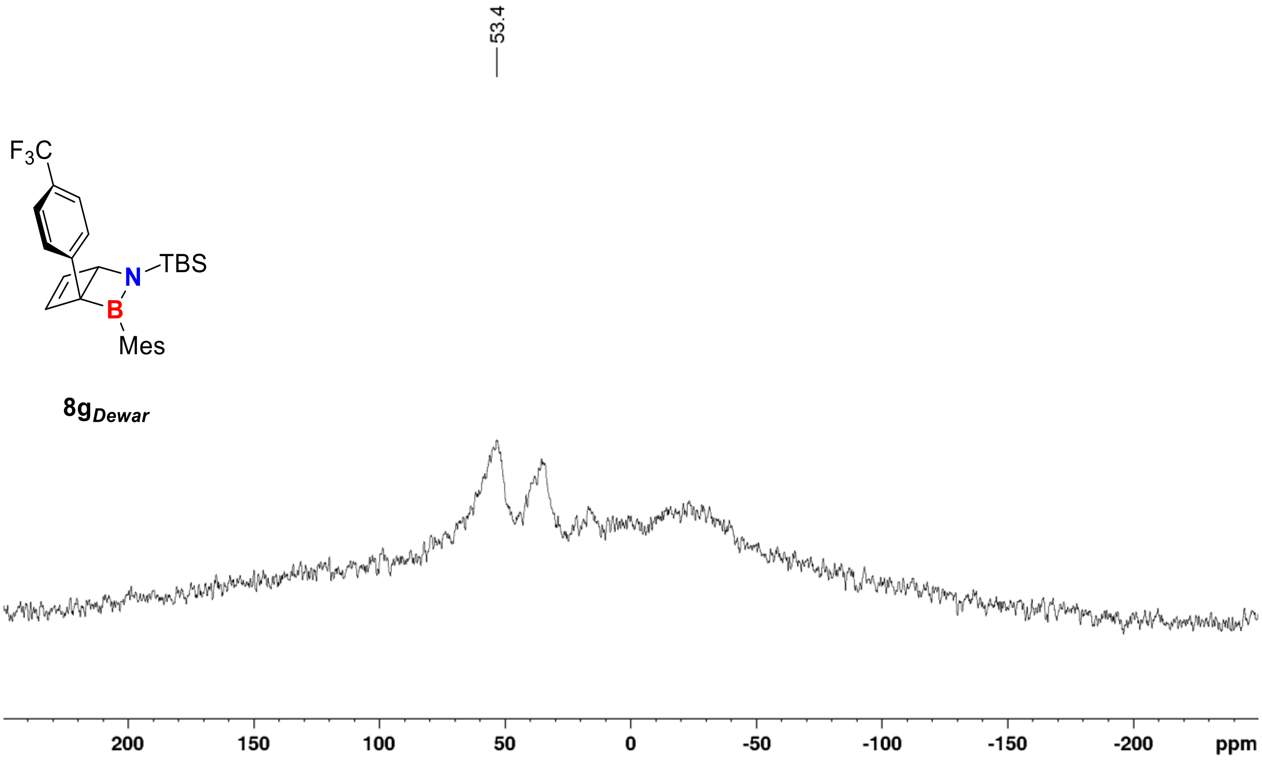

Figure S154. ^{11}B-{^{1}H}-NMR spectrum of compound **8g*Dewar*** in C_6D_{12} measured at a 400 MHz spectrometer.

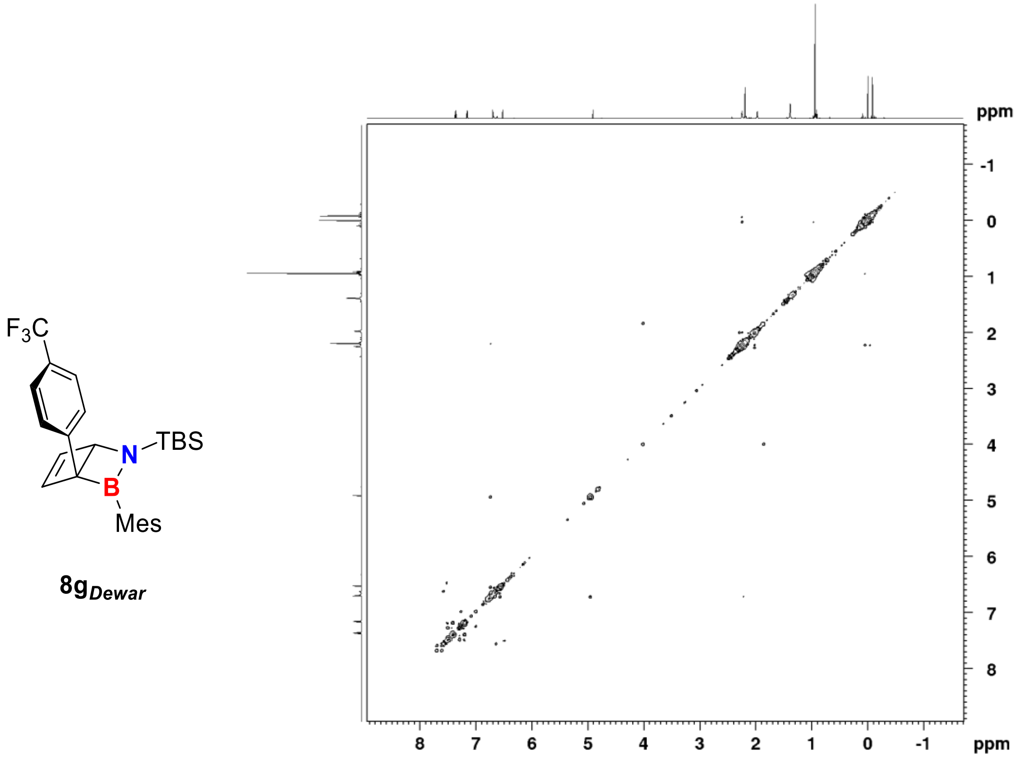

Figure S155. ^{1}H-^{1}H-COSY-NMR spectrum of compound **8g*Dewar*** in C_6D_{12} measured at a 700 MHz spectrometer.

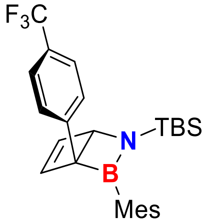

8g*Dewar*

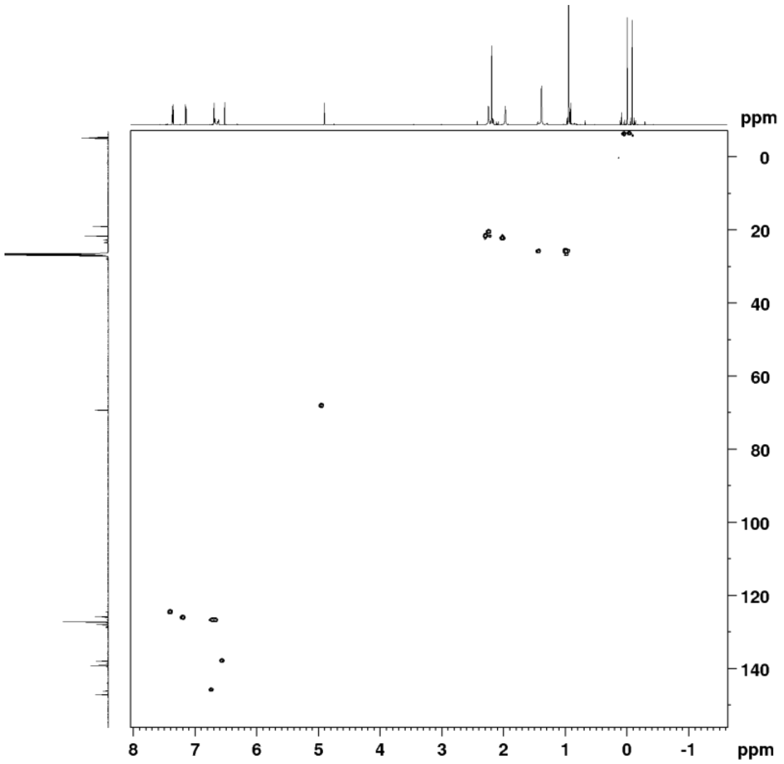

Figure S156. ^{1}H-^{13}C-HSQC-NMR spectrum of compound **8g*Dewar*** in C_6D_{12} measured at a 700 MHz spectrometer.

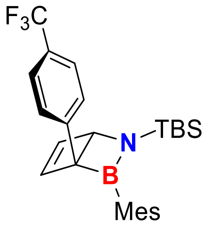

8g*Dewar*

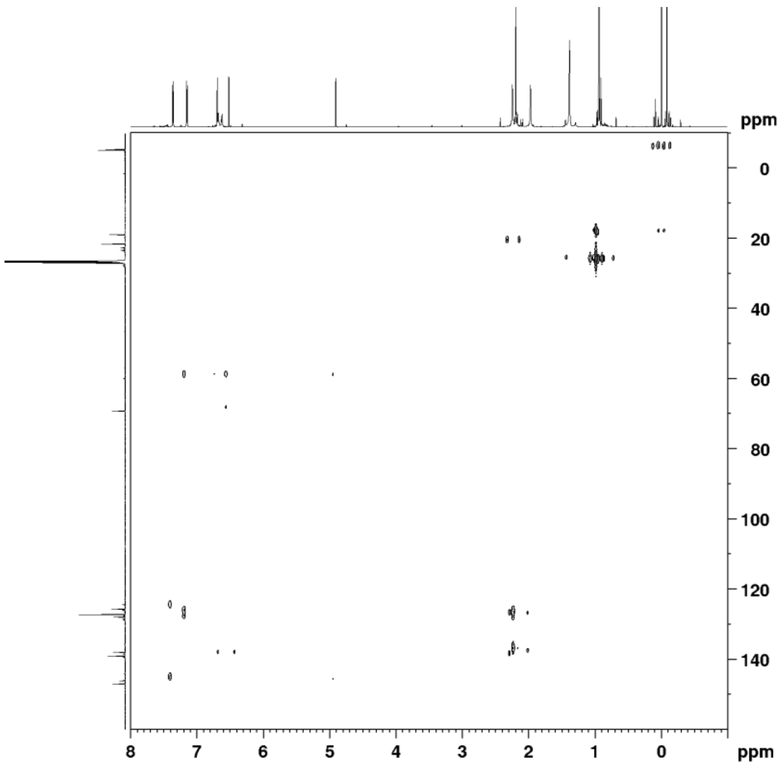

Figure S157. ^{1}H-^{13}C-HMBC-NMR spectrum of compound **8g*Dewar*** in C_6D_{12} measured at a 700 MHz spectrometer.

NMR data of 8h*Dewar*

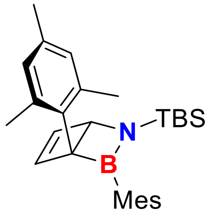

8h*Dewar*

$C_{28}H_{40}BNSi$ (429.53 g/mol)

^{1}H-NMR (700 MHz, **C_6D_{12}**): δ = 6.87 (ps. t, 1H, H-5), 6.62 (br. s, 1H, H-14), 6.56 (s, 2H, H-20), 6.46 (br. s, 1H, H-14), 6.41 (d, $^4J_{HH}$ = 2.58 Hz, 1H, H-4), 5.68 (d, $^4J_{HH}$ = 2.58 Hz, 1H, H-6), 2.26 (s, 3H, H-19), 2.18 (m, 6H, H-13), 2.14 (s, 3H, H-19), 2.11 (s, 3H, H-16), 1.65 (s, 3H, H-22), 0.95 (s, 9H, H-10), -0.05 (s, 3H, H-8), -0.11 (s, 3H, H-8) ppm.

^{13}C-{^{1}H}-NMR (176 MHz, **C_6D_{12}**): δ = 146.0 (C5), 138.7 (C21), 138.0 (C12), 137.0 (C18), 136.7 (C4), 134.5 (C17), 133.7 (C15), 130.4 (C20), 127.5 (C14), 127.3 (C14), 69.2 (C6), 63.8 (C3), 26.8 (C10), 22.8 (C19), 22.0 (C22), 21.4 (C13), 20.7 (C16), 18.9 (C9), -5.2 (C8), -5.8 (C8) ppm.

^{11}B-{^{1}H}-NMR (700 MHz, **C_6D_{12}**): δ = 52.5 ppm.

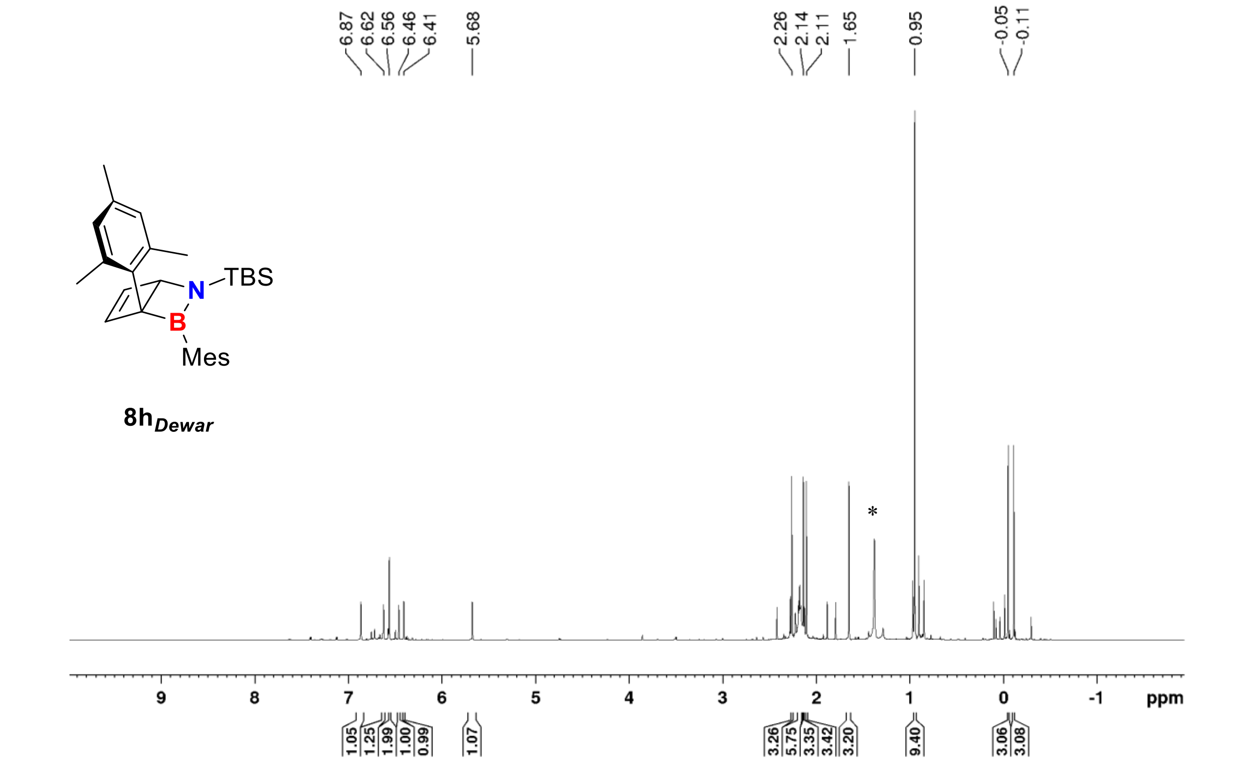

Figure S158. ^{1}H-NMR spectrum of compound **8h**$_{\boldsymbol{Dewar}}$ in C6D12 measured at a 700 MHz spectrometer. The solvent signal is marked with an asterisk.

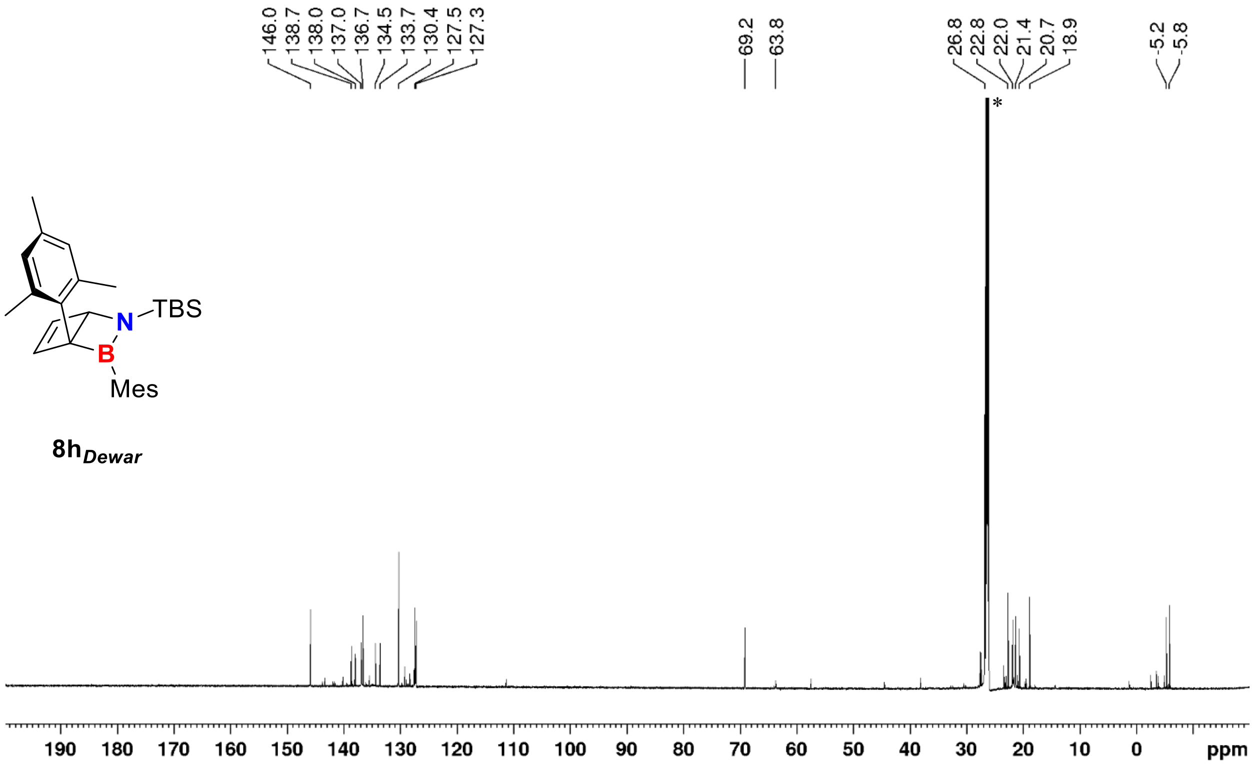

Figure S159. ^{13}C-{^{1}H}-NMR spectrum of compound **8h**$_{\boldsymbol{Dewar}}$ in C_6D_{12} measured at a 700 MHz spectrometer. The solvent signal is marked with an asterisk.

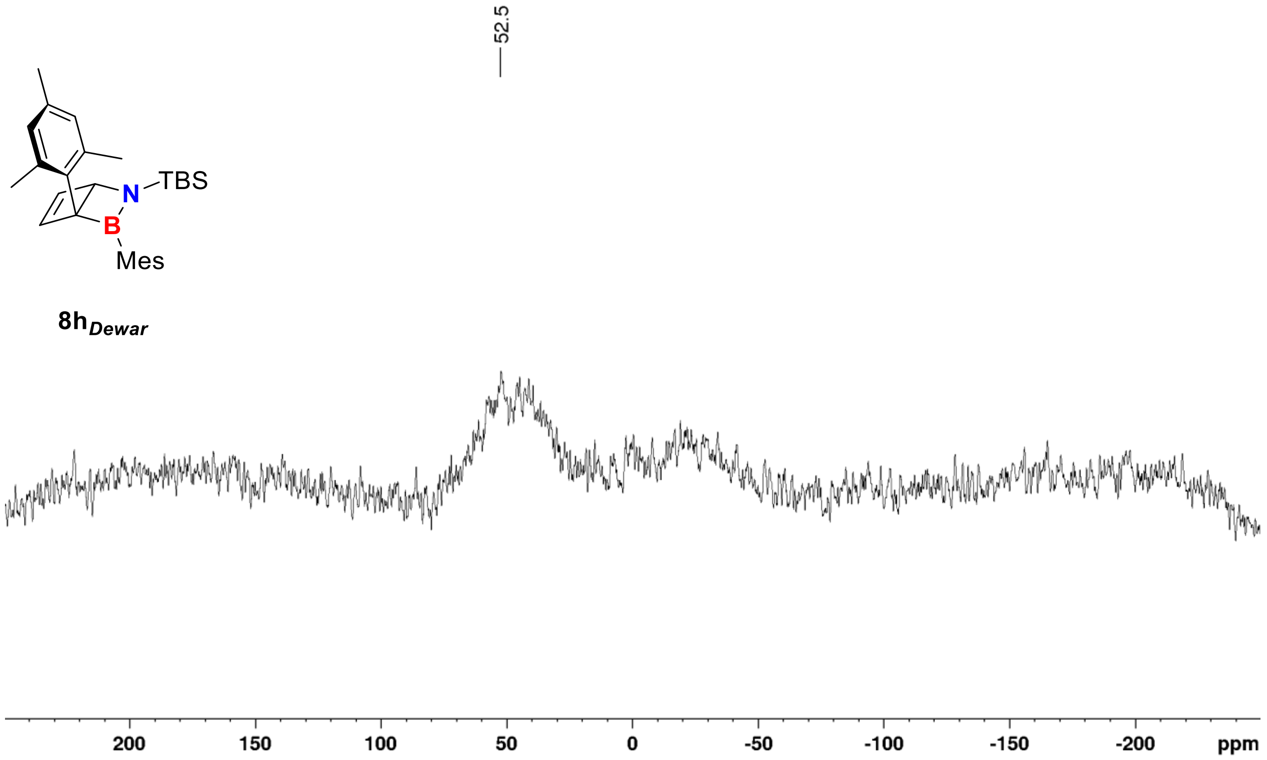

Figure S160. ^{11}B-{^{1}H}-NMR spectrum of compound **8h*Dewar*** in C_6D_{12} measured at a 600 MHz spectrometer.

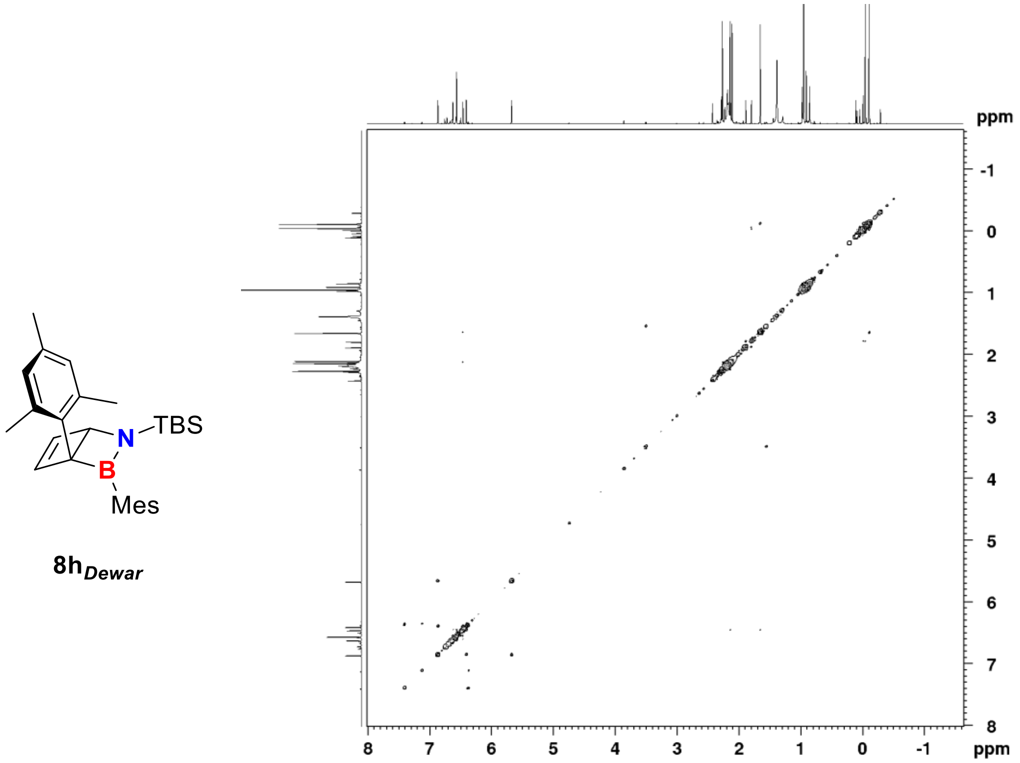

Figure S161. ^{1}H-^{1}H-COSY-NMR spectrum of compound **8h*Dewar*** in C_6D_{12} measured at a 700 MHz spectrometer.

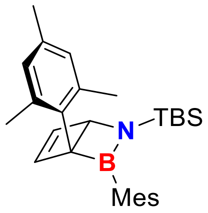

8h*Dewar*

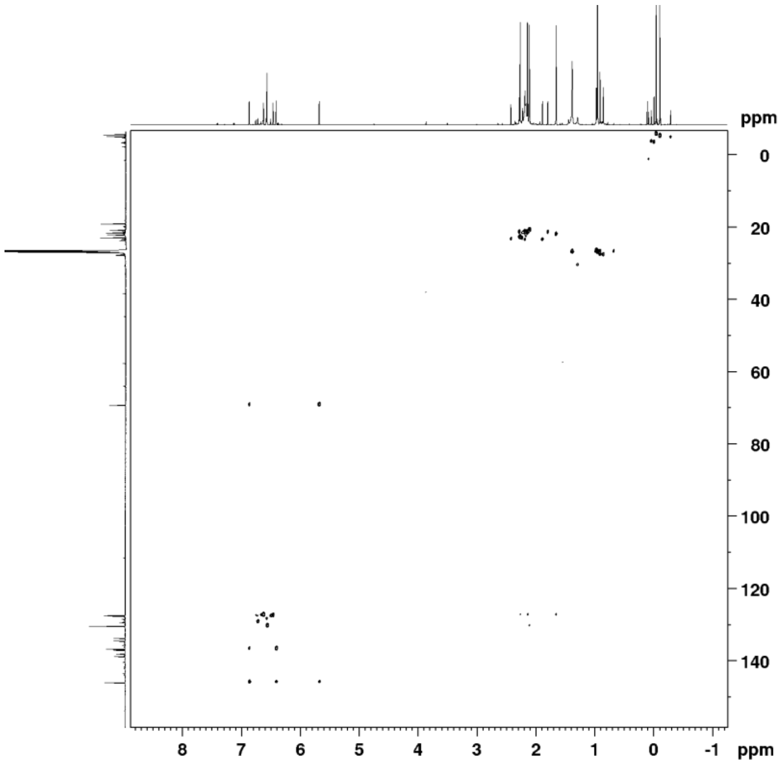

Figure S162. ^{1}H-^{13}C-HSQC-NMR spectrum of compound **8h*Dewar*** in C_6D_{12} measured at a 700 MHz spectrometer.

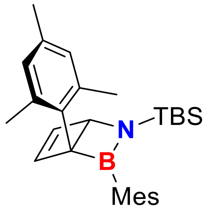

8h*Dewar*

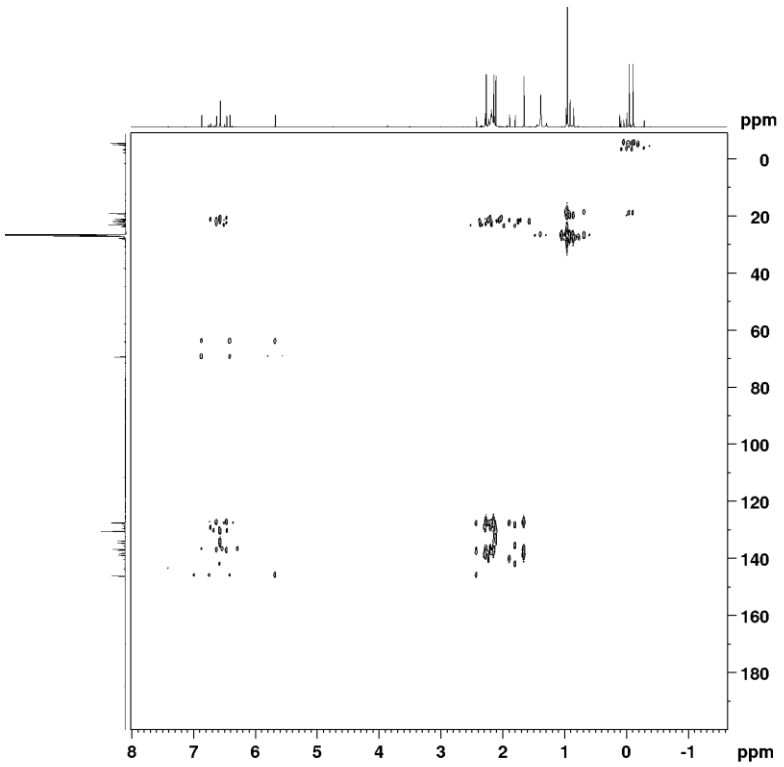

Figure S163. ^{1}H-^{13}C-HMBC-NMR spectrum of compound **8h*Dewar*** in C_6D_{12} measured at a 700 MHz spectrometer.

NMR data of 8j*Dewar*

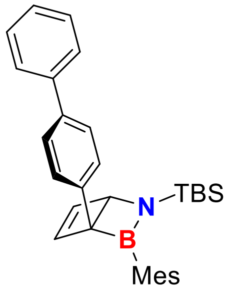

8j*Dewar*

$C_{31}H_{38}BNSi$ (393.47 g/mol)

^{1}H-NMR (700 MHz, **C_6D_{12}**): δ = 7.40 (m, 2H, H-18), 7.30 (m, 2H, H-22), 7.24 (m, 2H, H-19), 7.13 (m, 3H, H-23/H-24), 6.71 (ps. t, 1H, H-5), 6.67 (br. s, 1H, H-14), 6.62 (br. s, 1H, H-14), 6.51 (d, $^4J_{HH}$ = 2.32 Hz, 1H, H-4), 4.89 (d, $^4J_{HH}$ = 2.32 Hz, 1H, H-6), 2.26 (br. s, 3H, H-13), 2.19 (s, 3H, H-16), 2.03 (br. s, 3H, H-13), 0.94 (s, 9H, H-10), -0.01 (s, 3H, H-8), -0.09 (s, 3H, H-8) ppm.

^{13}C-{^{1}H}-NMR (176 MHz, **C_6D_{12}**): δ = 147.7 (C5), 142.5 (C20), 140.7 (C21), 139.3 (C17), 139.0 (C12), 138.6 (C4), 138.5 (C12), 137.5 (C15), 136.4 (br. C11), 128.9 (C19), 127.7 (C14), 127.5 (C22), 127.4 (C18), 127.0 (C23/C24), 69.4 (C6), 59.7 (C3), 26.9 (C10), 23.3 (C13), 22.6 (C13), 21.5 (C16), 18.8 (C9), -5.3 (C8), -5.4 (C8) ppm.

^{11}B-{^{1}H}-NMR (700 MHz, **C_6D_{12}**): δ =

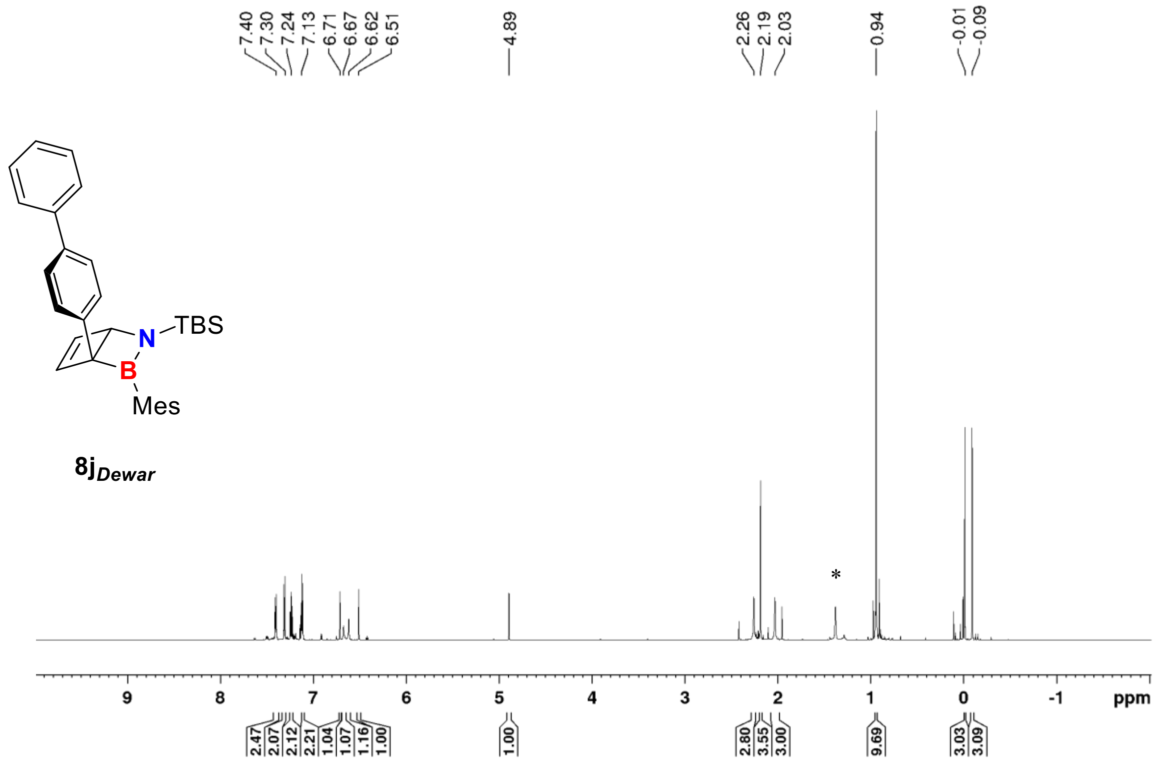

Figure S164. ^{1}H-NMR spectrum of compound **8j**$_{\textit{Dewar}}$ in C6D12 measured at a 700 MHz spectrometer. The solvent signal is marked with an asterisk.

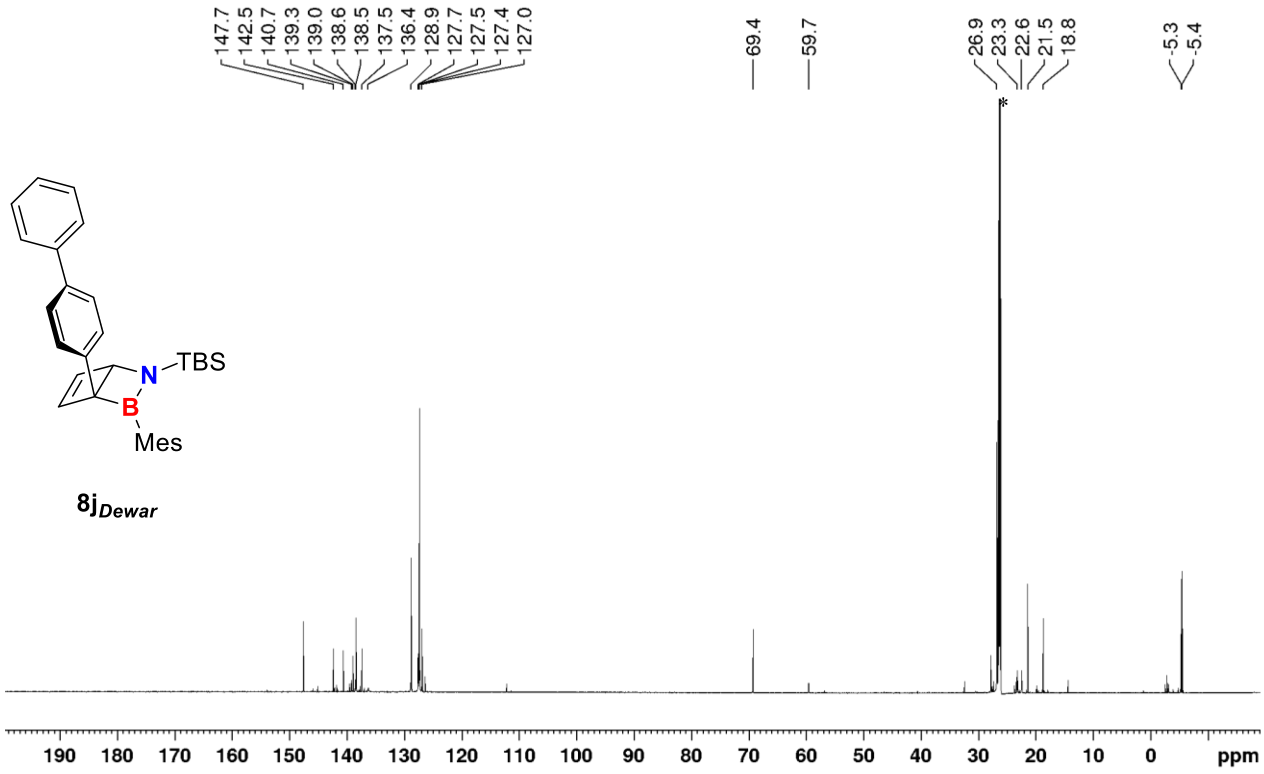

Figure S165. ^{13}C-{^{1}H}-NMR spectrum of compound **8j**$_{\textit{Dewar}}$ in C_6D_{12} measured at a 700 MHz spectrometer. The solvent signal is marked with an asterisk.

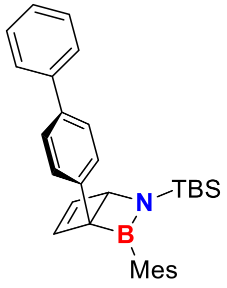

8j*Dewar*

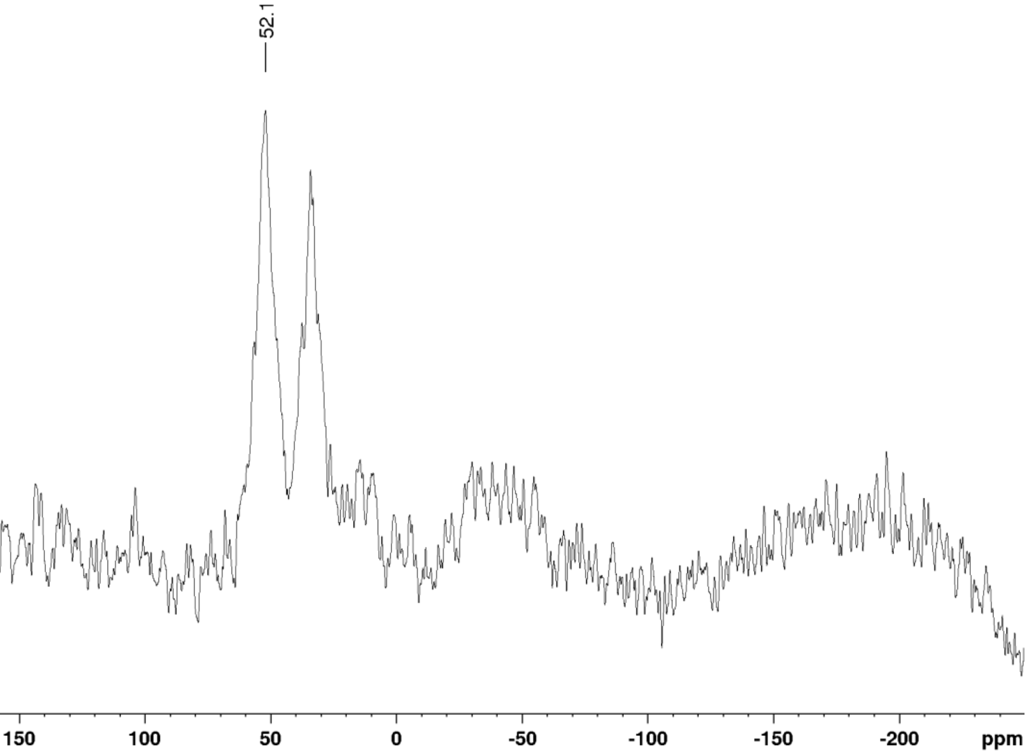

Figure S166. ^{11}B-{^{1}H}-NMR spectrum of compound **8j*Dewar*** in C_6D_{12} measured at a 400 MHz spectrometer.

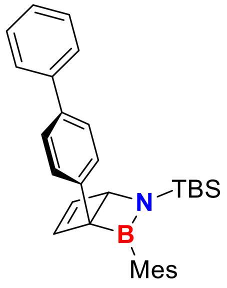

8j*Dewar*

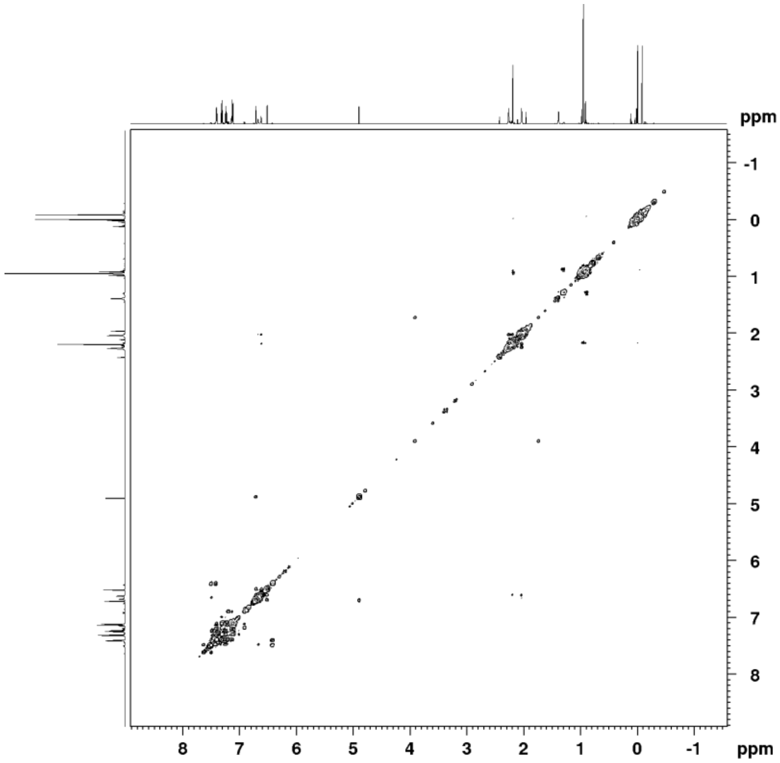

Figure S167. ^{1}H-^{1}H-COSY-NMR spectrum of compound **8j*Dewar*** in C_6D_{12} measured at a 700 MHz spectrometer.

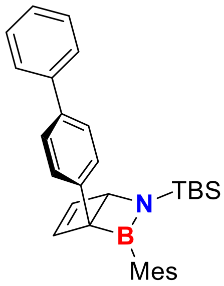

8j*Dewar*

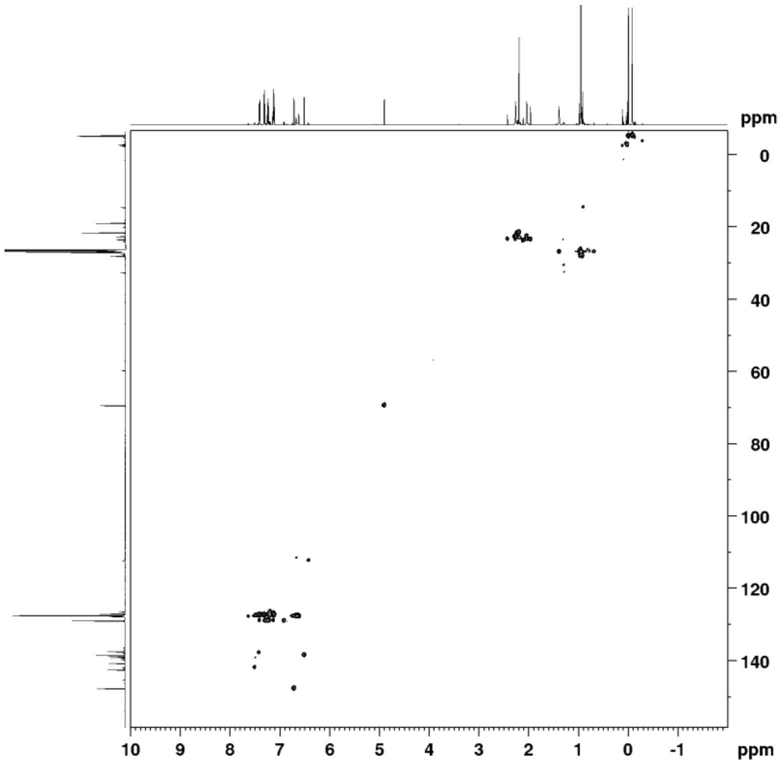

Figure S168. ^{1}H-^{13}C-HSQC-NMR spectrum of compound **8j*Dewar*** in C_6D_{12} measured at a 700 MHz spectrometer.

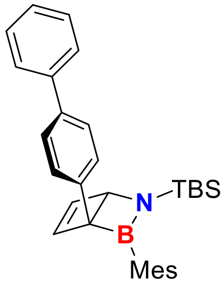

8j*Dewar*

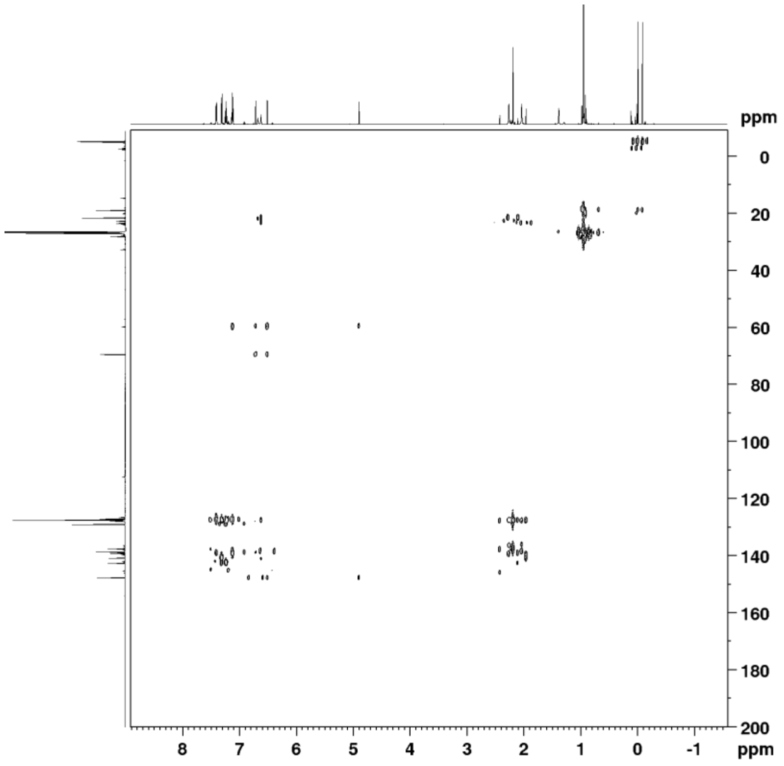

Figure S169. ^{1}H-^{13}C-HMBC-NMR spectrum of compound **8j*Dewar*** in C_6D_{12} measured at a 700 MHz spectrometer.

NMR data of 8k*Dewar*

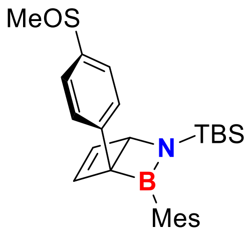

8k*Dewar*

$C_{26}H_{36}BNO_2SSi$ (465.53 g/mol)

^{1}H-NMR (600 MHz, **C_6D_{12}**): δ = 7.36 (dm, $^3J_{HH}$ = 8.15 Hz, 2H, H-), 7.18 ($^3J_{HH}$ = 8.15 Hz, 2H, H-), 6.70 (ps. q, 1H, H-5), 6.67 (br. s, 1H, H-14), 6.62 (br. s, 1H, H-14), 6.52 (d, $^4J_{HH}$ = 2.40 Hz, 1H, H-4), 4.90 (dd, $^3J_{HH}$ = 5.00 Hz, $^4J_{HH}$ = 2.40 Hz, 1H, H-6), 2.38 (s, 3H, H-22), 2.24 (br. s, 3H, H-13), 2.18 (s, 3H, H-16), 1.97 (br. s, 3H, H-13), 0.94 (s, 9H, H-10), -0.01 (s, 3H, H-8), -0.09 (s, 3H, H-8) ppm.

^{13}C-{^{1}H}-NMR (151 MHz, **C_6D_{12}**): δ = 147.1 (C5), 145.2 (C20), 144.6 (C17), 139.4 (C11), 138.9 (C4), 138.5 (C14), 137.8 (C15), 127.8 (C12), 127.6 (C18), 123.7 (C19), 69.3 (C6), 69.2 (C6), 59.8 (C3), 44.8 (C22), 27.9 (C10), 23.2 (C13), 22.5 (C13), 21.5 (C16), 18.8 (C9), -5.3 (C8), -5.5 (C8) ppm.

^{11}B-{^{1}H}-NMR (192 MHz, **C_6D_{12}**): δ = 51.8 ppm.

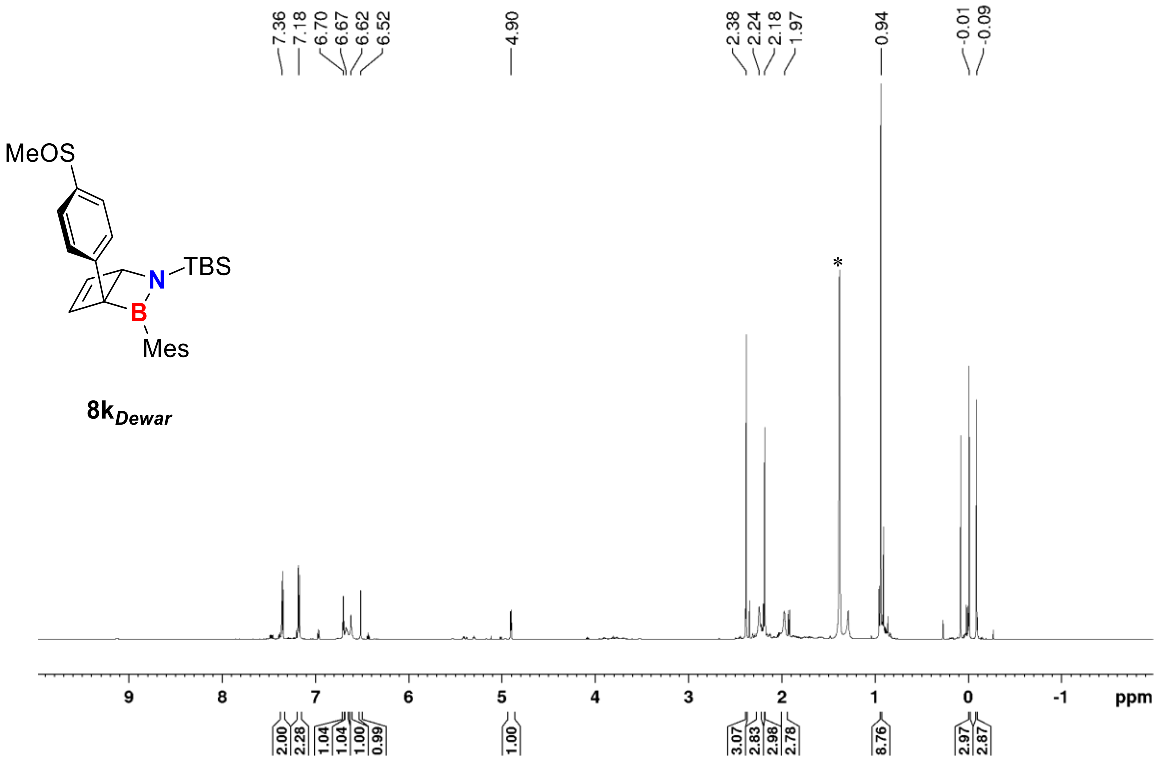

Figure S170. ^{1}H-NMR spectrum of compound **8k*Dewar*** in C6D12 measured at a 600 MHz spectrometer. The solvent signal is marked with an asterisk.

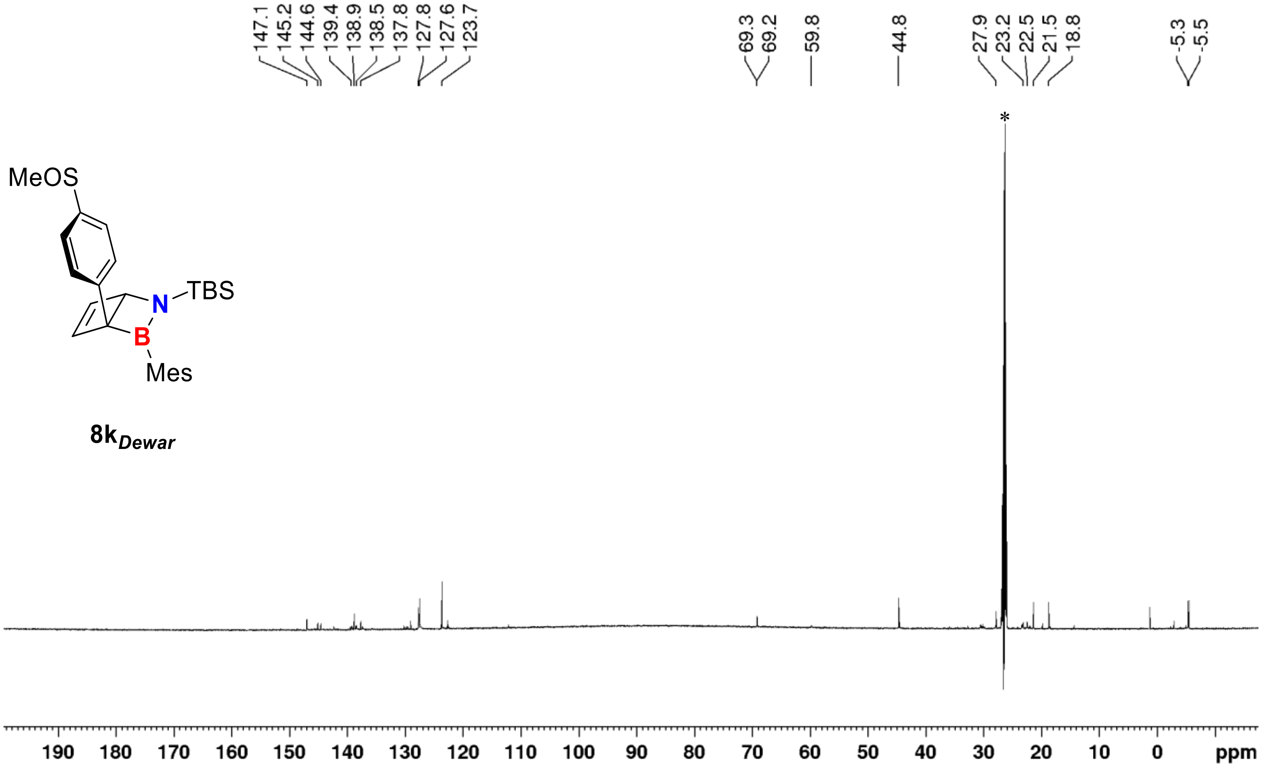

Figure S171. ^{13}C-{^{1}H}-NMR spectrum of compound **8k*Dewar*** in C_6D_{12} measured at a 600 MHz spectrometer. The solvent signal is marked with an asterisk.

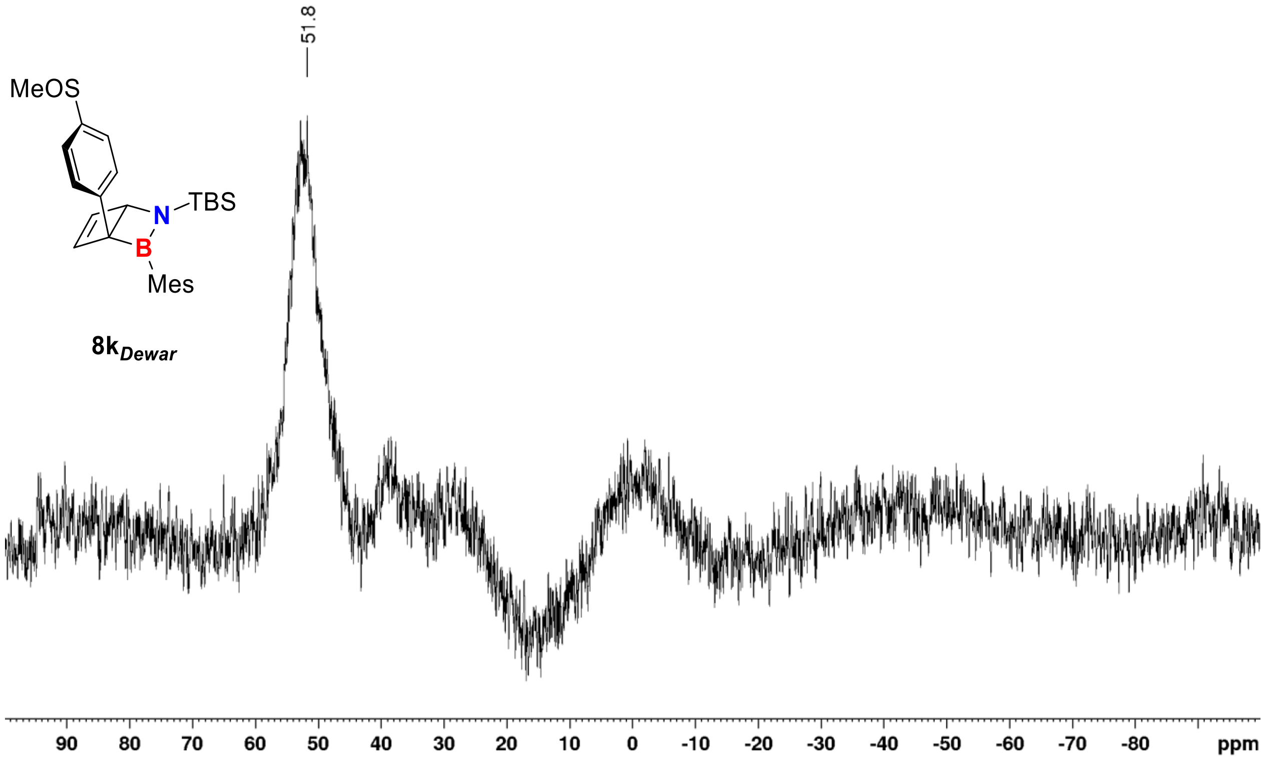

Figure S172. ^{11}B-{^{1}H}-NMR spectrum of compound **8k*Dewar*** in C_6D_{12} measured at a 600 MHz spectrometer.

5. Kinetic experiments

Sample preparation

The dihydroazaborinine **8a-8k** except for **8d** were solved in deuterated cyclohexane. As their solubility varies drastically different concentrations were prepared (see Table S2). This solution was placed in a J. Young quartz tube and an irradiation as described in section 4 was conducted to obtain the *Dewar* isomer.

Table S2. Mass oft the dihydroazaborinine (in mg) and volume of C_6D_{12} (in mL) used for the preparations of the samples for the kinetic studies. The resulting concentration is given in M.

	M (g/mol)	m (mg)	V (mL)	c (M)
**8a**	387.26	11.1	0.60	0.05
		15.1	0.40	0.10
		12.4	0.45	0.07
**8b**	401.48	19.6	0.46	0.11
		21.5	0.55	0.10
		10.7	0.50	0.05
**8c**	417.48	14.0	0.45	0.03
		10.0	0.48	0.05
**8e**	430.52	20.8	0.48	0.10
**8f**	421.89	6.0	0.45	0.03
		12.4	0.45	0.07
**8g**	455.45	15.9	0.70	0.05
		21.1	0.50	0.10
**8h**	429.53	11.2	0.50	0.05
**8i**	393.47	23.8	0.60	0.10
**8j**	463.55	21.9	0.47	0.10
		14.7	0.45	0.07
**8k**	465.53	8.0	0.43	0.04

Experimental details

It was demonstrated earlier by Edel *et al.*,[15] that the thermal ring opening follows first order kinetics. In agreement with that, we observed first order kinetics for the dihydroazaborinines **8a-8k**. This result was highly reproducible as the kinetic for **8b-8k**, except for **8h**, was measured two times. For compound **8a** each temperature was measured three times. The thermal ring-opening of each compound was monitored using proton NMR spectroscopy at four different temperatures. Since the half-lives vary drastically, these four

temperatures had to be adjusted accordingly. The specific temperatures measured for each compound are listed in Table S3. The sample was allowed to reach the desired temperature, then every 2 min or every 5 min a ^{1}H NMR spectrum was recorded (16 scans; total experiment time = 126 s) until at least 40% conversion to the dihydroazaborinine were observed.

Table S3. Temperatures considered for the kinetic analysis in K.

Subst.	T_1 (K)	T_2 (K)	T_3 (K)	T_4 (K)
**8a**	338	333	328	323
**8b**	328	323	318	313
**8c**	318	313	308	303
**8e**	303	298	293	288
**8f**	323	318	313	308
**8g**	328	323	318	313
**8h**	338	333	328	323
**8i**	313	308	303	298
**8j**	323	318	313	308
**8k**	303	298	293	288

Table S4. Rate constants calculated from NMR data at the temperatures given in Table S3 under the estimation of a first order kinetic.

	k_{T1} ($s^{-1} \cdot 10^4$)	k_{T2} ($s^{-1} \cdot 10^4$)	k_{T3} ($s^{-1} \cdot 10^4$)	k_{T4} ($s^{-1} \cdot 10^4$)
**8a**	3.787	2.206	1.272	0.677
	2.677	1.528	1.146	0.654
	2.493	1.532	0.829	0.470
**8b**	1.928	1.095	0.592	0.421
	1.929	1.112	0.652	0.349
**8c**	2.146	1.215	0.703	0.361
	1.697	0.988	0.510	0.293
**8e**	3.335	1.808	1.003	0.550
	3.058	1.893	1.007	0.528
**8f**	0.789	0.557	0.242	0.169
	0.788	0.466	0.330	0.173
**8g**	1.400	0.604	0.454	0.258
	1.433	-	0.434	0.244
**8h**	0.934	0.510	0.291	0.159
**8i**	6.067	2.999	1.650	1.121
	6.591	3.674	1.641	1.162
**8j**	1.656	0.928	0.400	0.295
	1.281	0.736	0.415	0.219
**8k**	2.568	1.562	0.904	0.301
	2.625	1.535	0.913	0.320

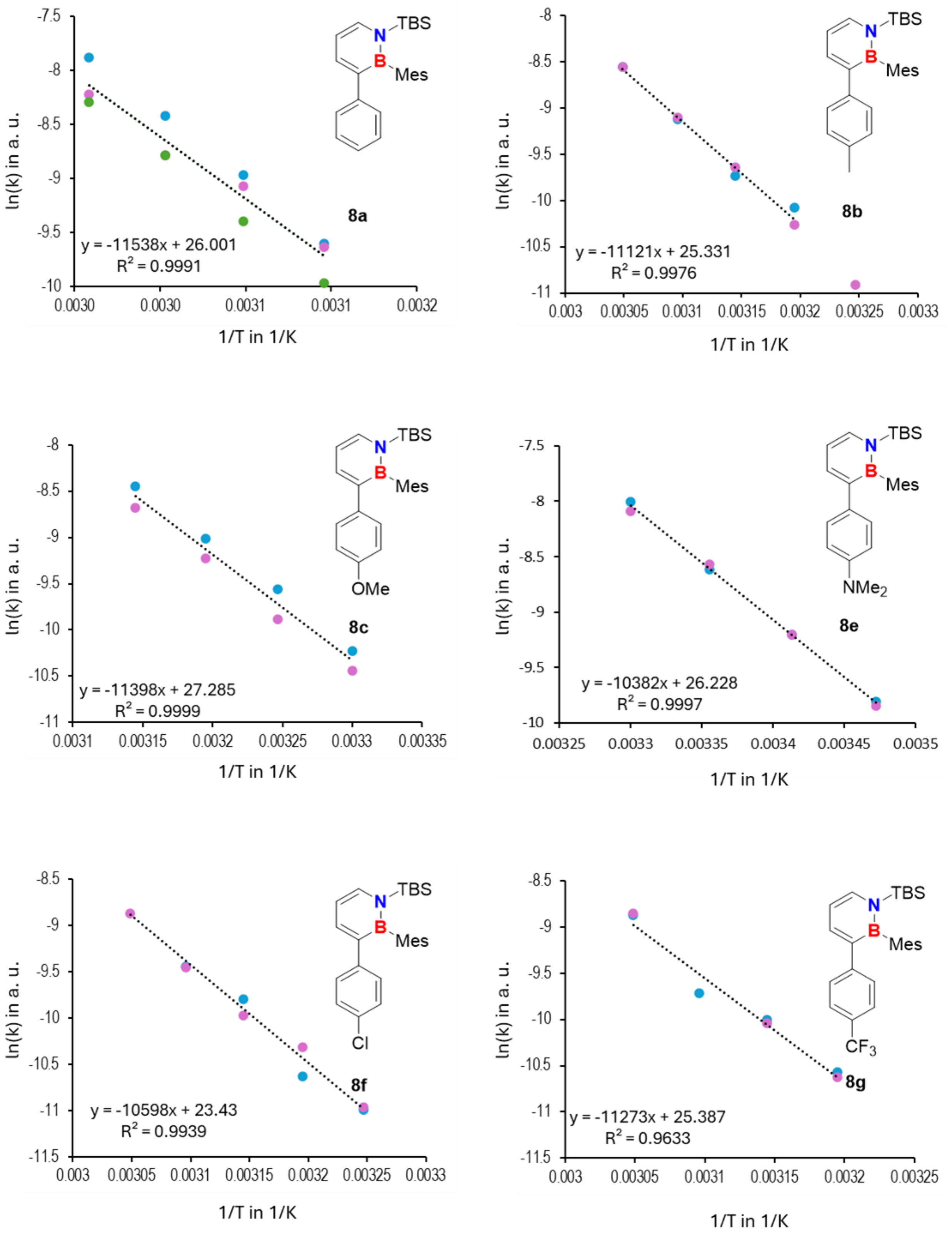

Figure S173. Arrhenius plots for the compounds **8a**-**8k**, except for **8d**. The first measurements are given in blue and the second measurements in pink. The unit of the rate constants used is s^{-1}, as the reaction is first order.

Figure S173 (continued)

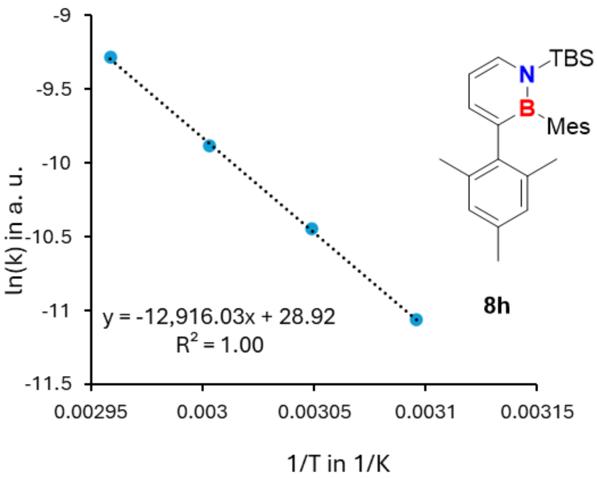

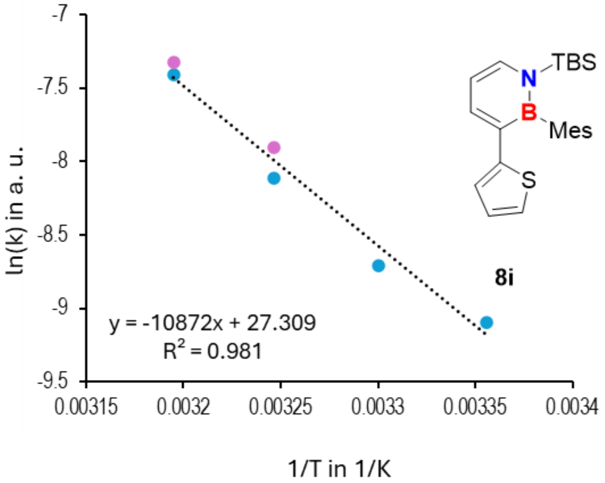

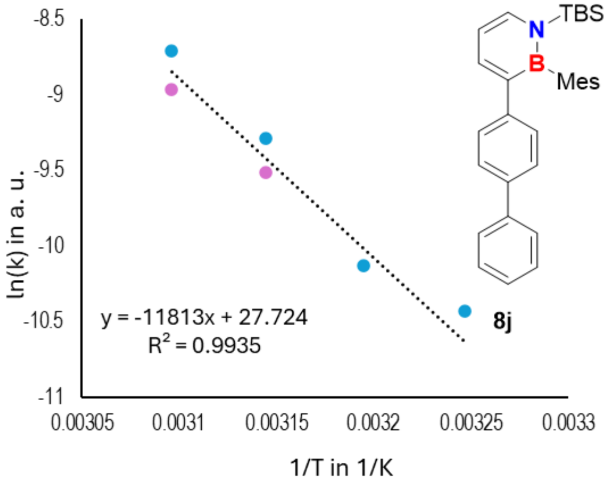

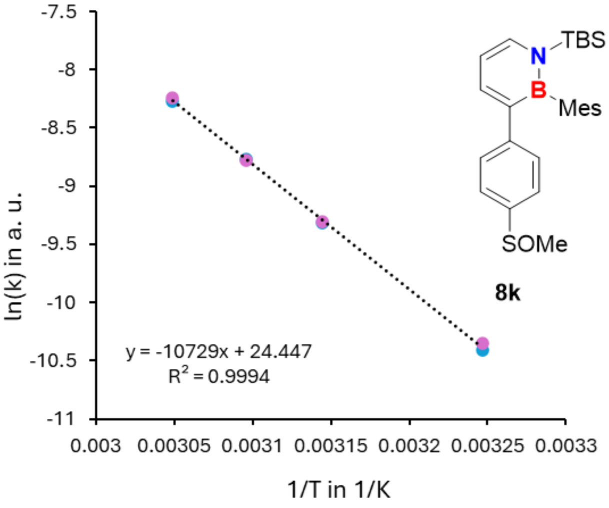

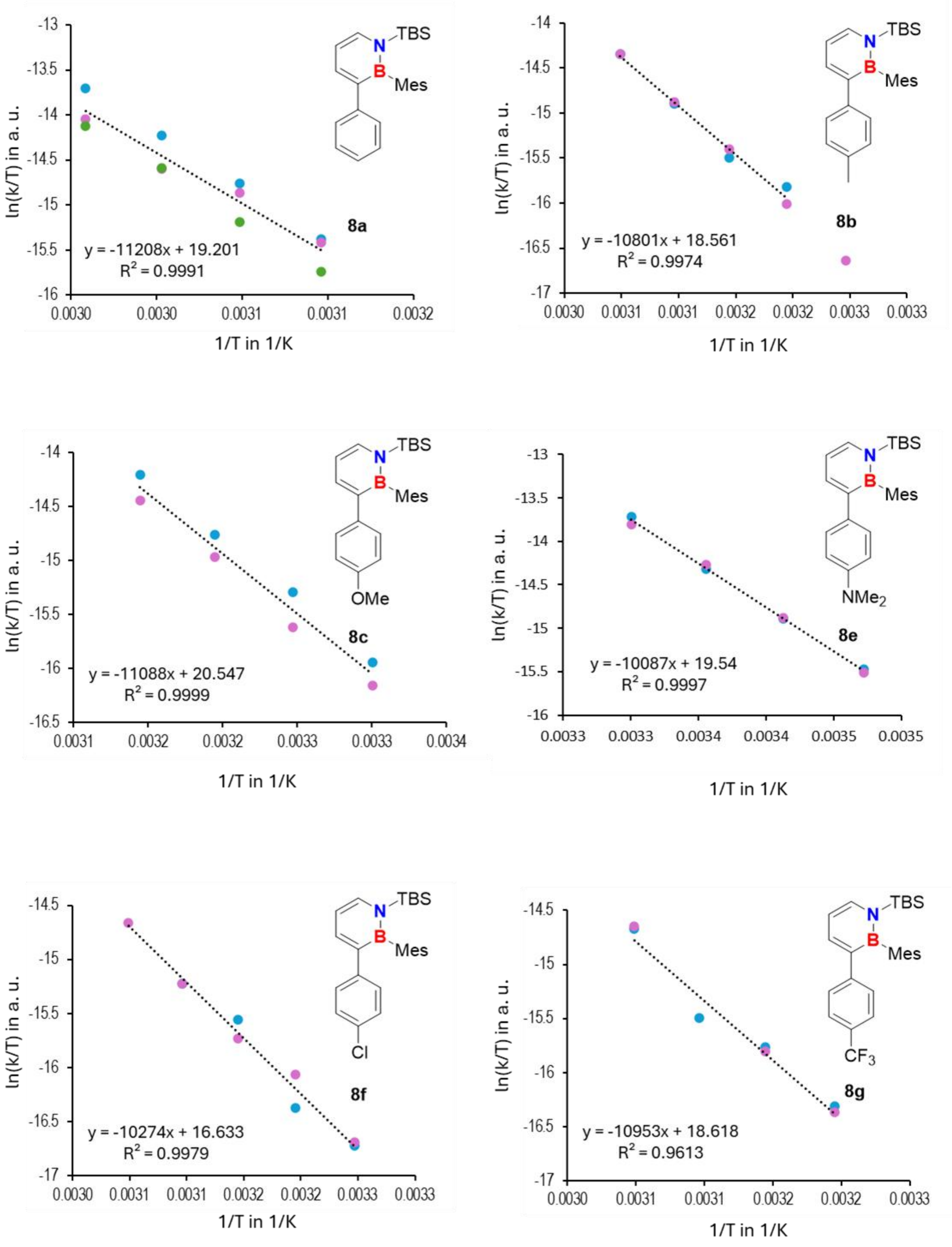

Figure S174. Eyring plots for the compounds **8a-8k**, except for **8d**. The first measurements are given in blue and the second measurements in pink. The unit of the rate constants used is s^{-1}, as the reaction is first order.

Figure S174 (continued)

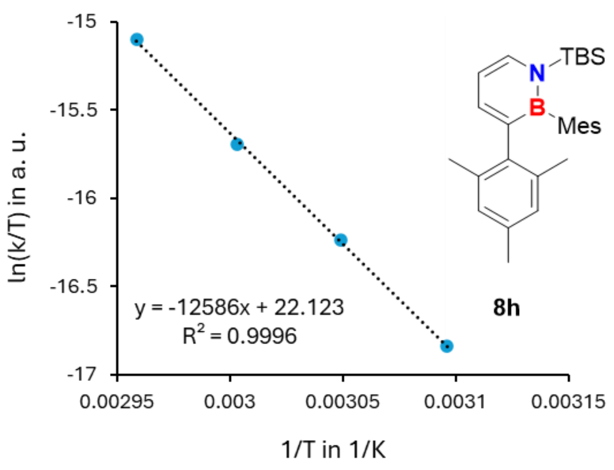

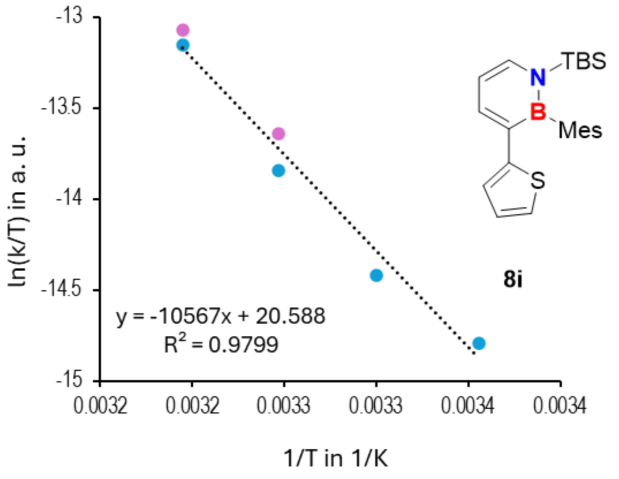

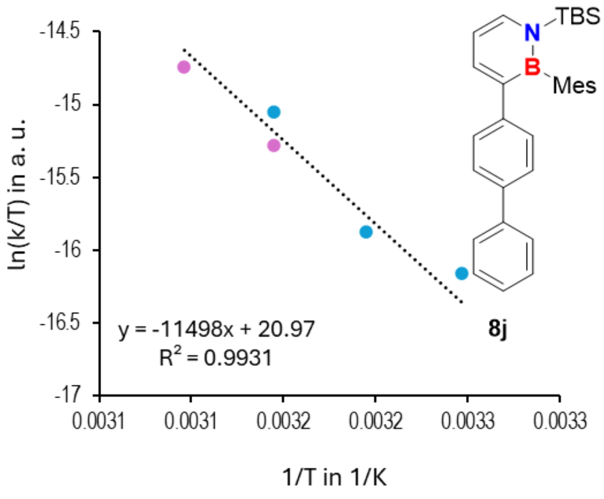

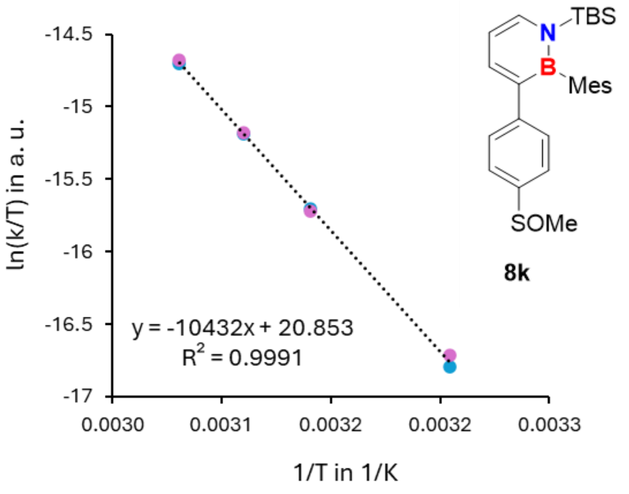

The parameters from the linear regression of these Arrhenius and Eyring plots enable the determination of the following reaction barriers and half-life's.

Table S5. Activation barriers determined from the Arrhenius treatment, $\Delta H^{‡}_{ro}$, $\Delta S^{‡}_{ro}$ and $\Delta G^{‡}_{ro}$ calculated from the parameters of an Eyring plot, as well as calculated data for **8a**, **8e** and **8k**.

		**8a**	**8b**	**8c**	**8e**	**8f**	**8g**	**8h**	**8i**	**8j**	**8k**
Arrhenius	$E_{a1, exp}$ (kcal/mol)	22.93	22.10	22.65	20.63	21.06	22.40	25.66	21.60	23.47	17.18
	$t_{1/2,1}$ (d)	3.86	1.86	0.67	0.06	2.15	2.93	21.23	0.11	1.73	1.19
Eyring	$\Delta H^{‡}_{ro}$ (kcal/mol)	22.27	21.46	22.03	20.04	20.43	21.76	25.01	21.00	22.85	16.55
	$\Delta S^{‡}_{ro}$ (kcal/molK)	-0.009	-0.010	-0.006	-0.008	-0.014	-0.010	-0.003	-0.006	-0.006	-0.026
	$\Delta G^{‡}_{ro}$ (kcal/mol)	24.97	24.54	23.94	22.54	24.63	24.81	25.98	22.88	24.50	24.28
Theory	$\Delta G^{‡}_{ro}$ (kcal/mol)	29.43	-	-	28.30	-	-	-	-	-	27.80
	$\Delta\Delta G^{‡}_{ro}$ (kcal/mol)	4.46			5.76						3.52

ro: ring opening

As can be seen from the difference in reaction barriers ($\Delta\Delta G^{‡}_{ro}$), calculations and experiments show the same trend. However, there is a deviation of about 5 kcal/mol in the absolute values.

Time dependence (1. order kinetic)

For deriving the concentration dependence, the following assumptions are made. The reaction under consideration is of the type:

$$A \xrightarrow{k_1} B$$

The processe is assumed to be irreversible, follow a first order kinetic and has the rate constants k_1. This is in very good agreement with our experimental findings. The initial concentration of compound A is $A_0 = 1$. While the initial concentrations of B is $B_0 = 0$.

If compound A follows a simple first order kinetic, its concentrations behavior is given as:

$$\frac{d[A]}{dt} = -k_1 \cdot [A]$$

This equation can be transformed to

$$\frac{d[A]}{[A]} = -k_1 \cdot dt$$

An integration from A_0 to A (left side) or 0 to t (right side) respectively, results in

$$\ln[A] - \ln A_0 = -k_1 \cdot t$$

which finally rearranges to the well-known formula equation 1.

$$\boldsymbol{[A] = A_0 \cdot e^{-k \quad \cdot t}} \qquad \textbf{(1)}$$

Hammett and Creary Parameters

The different Hammett parameters were sourced from Hansch *et al.*[16]

X	**8a** *H*	**8b** *Me*	**8j** *Ph*	**8c** *OMe*	**8i**	**8e** *NMe_2*	**8f** *Cl*	**8g** *CF_3*	**8k** *SOMe*
σ	0	-0.1	-0.01	-0.27	-0.09	-0.83	0.23	0.54	0.49
σ^+	0	-0.31	-0.18	-0.78	-	-1.7	0.11	0.61	-
σ^-	0	-0.17	0.02	-0.26	-	-0.12	0.19	0.65	0.73
$\sigma^\cdot$	0	0.11	0.46	0.24	-	0.90	0.12	0.08	0.18

Creary Plot

Beyond the extensively discussed correlations with the Hammett substituent σ^+ and σ^-, a reasonable correlation is likewise observed when employing Creary parameters.[17] These parameters are derived based on rearrangement rates that proceed *via* diradical intermediates and serve as a measure of a substituents ability to stabilize radical intermediates by resonance or inductive effects. As illustrated in the Figures S175 and S176, a deviation from the linear regression is, in this case, not observed for the CF_3 derivative **8g**, but rather for compound **8j** (X = Ph).

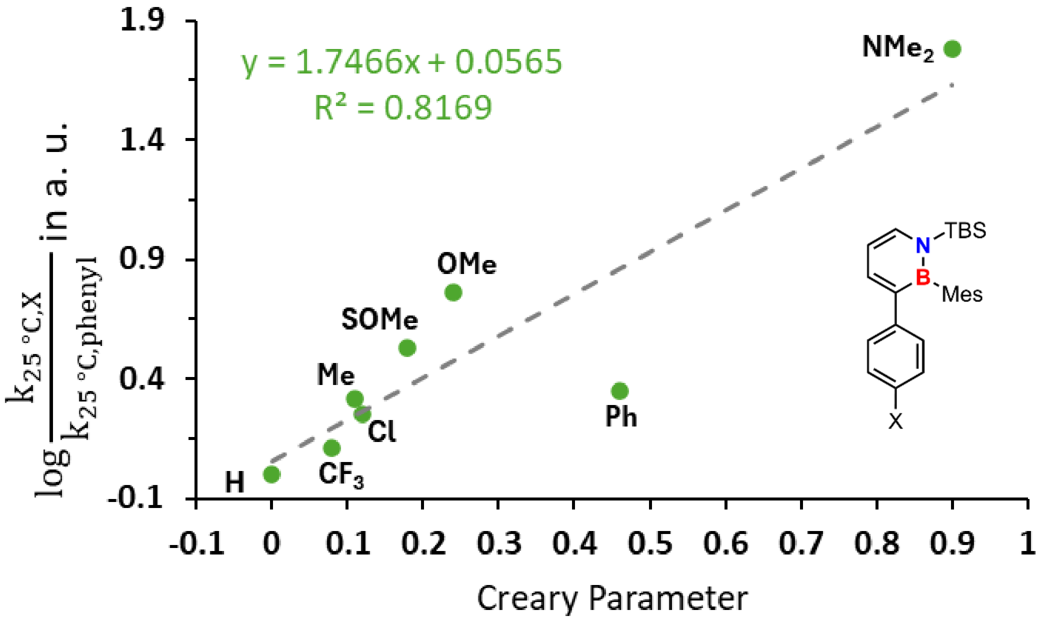

Figure S175. Creary plot of the investigated dihydroazaborinines upon inclusion of compound **8j** for the linear regression. The annotation at the individual points refers to the position marked with an X in the dihydroazaborinine on the right.

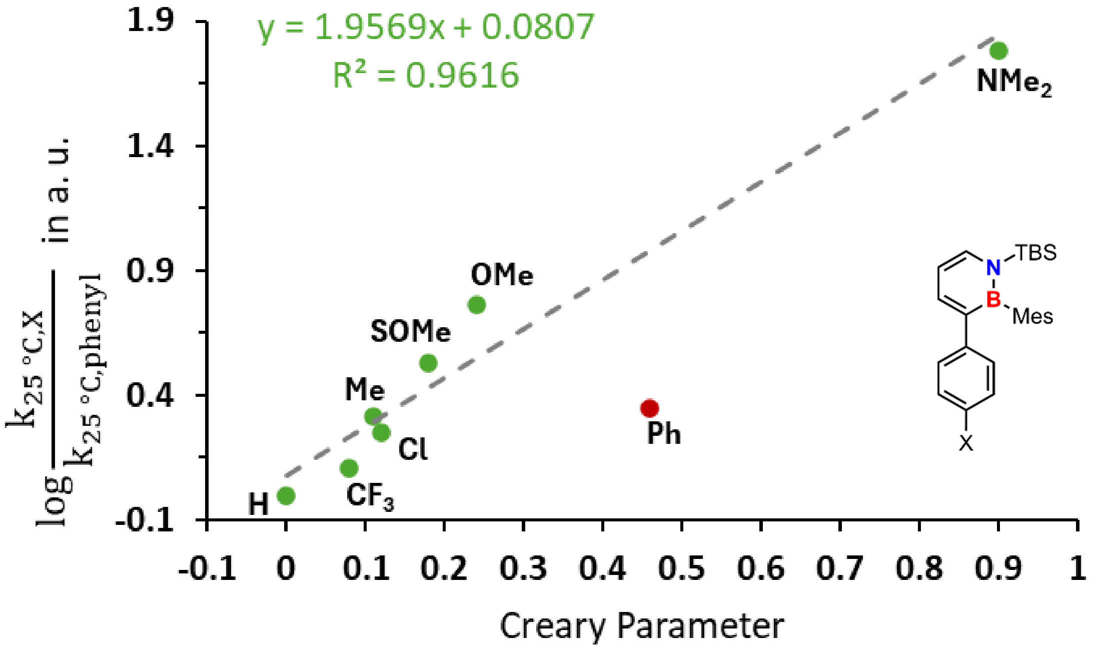

Figure S176. Creary plot of the investigated dihydroazaborinines excluding compound **8j** for the linear regression. The annotation at the individual points refers to the position marked with an X in the dihydroazaborinine on the right.

To assess whether any of the structures arising during the thermal ring-opening possess (partial) diradical character, the corresponding wavefunctions were tested for instabilities. All computed species relevant to the thermal ring opening of compounds **8a**, **8e**, and **8k** were examined computationally to test whether singlet instabilities exist for restricted wavefunctions. All wavefunctions were found to be stable under the considered perturbations. Consequently, the available computational data do not provide evidence for a diradical pathway in the thermal ring opening process.

7. Crystal structures

Refinement details

The implementation NoSpherA2 for non-spherical atom form factors in *Olex2* makes use of tailor made aspherical atomic form factors calculated from a Hirshfeld-partitioned electron density (ED), not from spherical atom form factors.[18] The electron density is calculated from a Gaussian basis set single determinant SCF wavefunction for a fragment of the crystal. This fragment can be embedded in an electrostatic crystal field by employing cluster charges or modelled using implicit solvation models, depending on the software used. This was computed using B3LYP/6-31G(d,p),[19-22] normal integration accuracy, a charge of 0 and a multiplicity of 1 with Orca 5.0.[23]

Table S6. Parameters and results of the X-ray diffraction measurement of compound **8j**.

Parameter	unit	8j
Molecular formula		$C_{31}H_{38}BNSi$
CCDC		2336783
$D_{calc.}$	g cm^{-3}	1.107
μ	mm^{-1}	0.863
Molar weight	g/mol	463.564
Colour		clear colorless
Shape		block-shaped
Size	mm^3	0.19×0.15×0.15
T/K		150.00(10)
Crystal System		triclinic
Space Group		P-1
a/Å		10.4913(7)
b/Å		12.3246(5)
c/Å		12.7384(5)
α/°		77.908(3)
β/°		68.387(4)
γ/°		65.507(5)
V	Å^3	1390.35(14)
Z		2
Z'		1
Wavelength	Å	1.54184
Radiation type		Cu K□
Θ_{min}	°	3.74
Θ_{max}	°	79.59
Measured Refl's.		47066
Indep't Refl's		5926
Refl's I≥2 σ(I)		5779
R_{int}		0.0136
Parameters		650
Restraints		0
Largest Peak		0.1289
Deepest Hole		-0.0807
GooF		1.5446
wR_2 (all data)		0.0297
wR_2		0.0296
R_1 (all data)		0.0122
R_1		0.0117

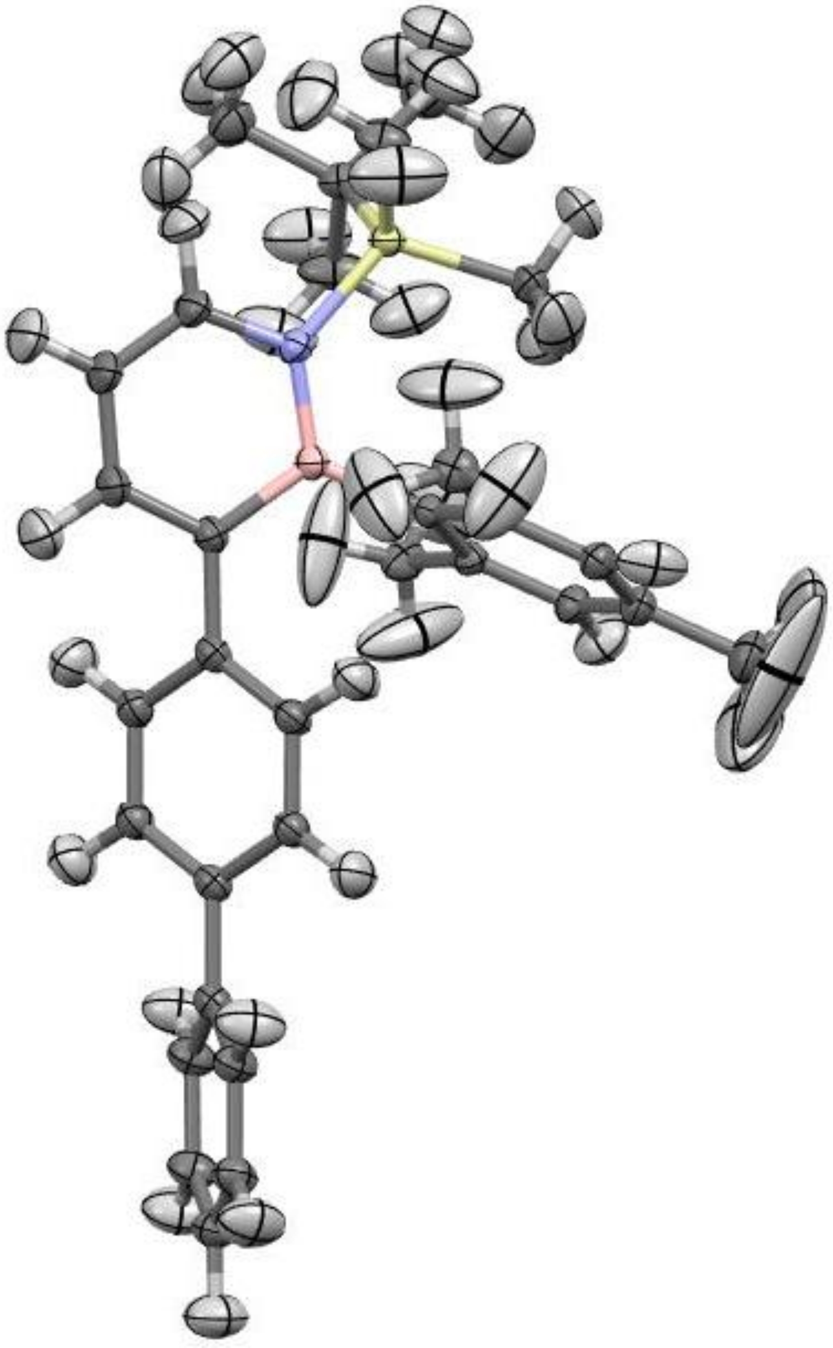

Figure S177. Crystal structure of **8j**. Thermal ellipsoids are drawn at the 50% probability level.

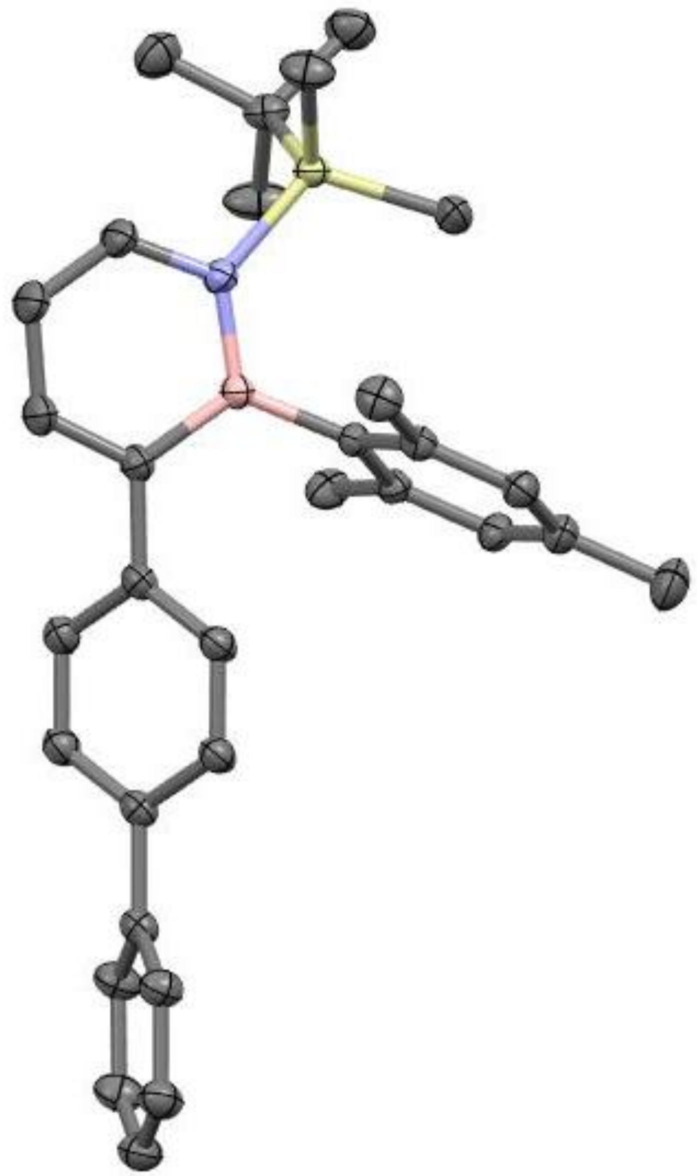

Figure S178. Crystal structure of **8j**. Hydrogens are omitted for clarity and thermal ellipsoids are drawn at the 50% probability level.

Table S7. Parameters and results of the X-ray diffraction measurement of compound **2**.

Parameter	unit	2
Molecular formula		$C_{19}H_{29}BBrNSi$
CCDC		2290772
$D_{calc.}$	g cm^{-3}	1.249
μ	mm^{-1}	3.221
Molar weight	g/mol	390.262
Colour		clear colorless
Shape		plate-shaped
Size	mm^3	0.14×0.12×0.01
T/K		150.00(10)
Crystal System		monoclinic
Space Group		$P2_1/n$
a/Å		7.4120(2)
b/Å		14.9644(3)
c/Å		18.8926(4)
α/°		90
β/°		97.992(2)
γ/°		90
V	Å^3	2075.14(8)
Z		4
Z'		1
Wavelength	Å	1.54184
Radiation type		Cu K□
Θ_{min}	°	3.78
Θ_{max}	°	78.72
Measured Refl's.		58429
Indep't Refl's		4333
Refl's I≥2 σ(I)		4136
R_{int}		0.0333
Parameters		469
Restraints		7
Largest Peak		0.5664
Deepest Hole		-0.4811
GooF		1.1232
wR_2 (all data)		0.0432
wR_2		0.0428
R_1 (all data)		0.0214
R_1		0.0204

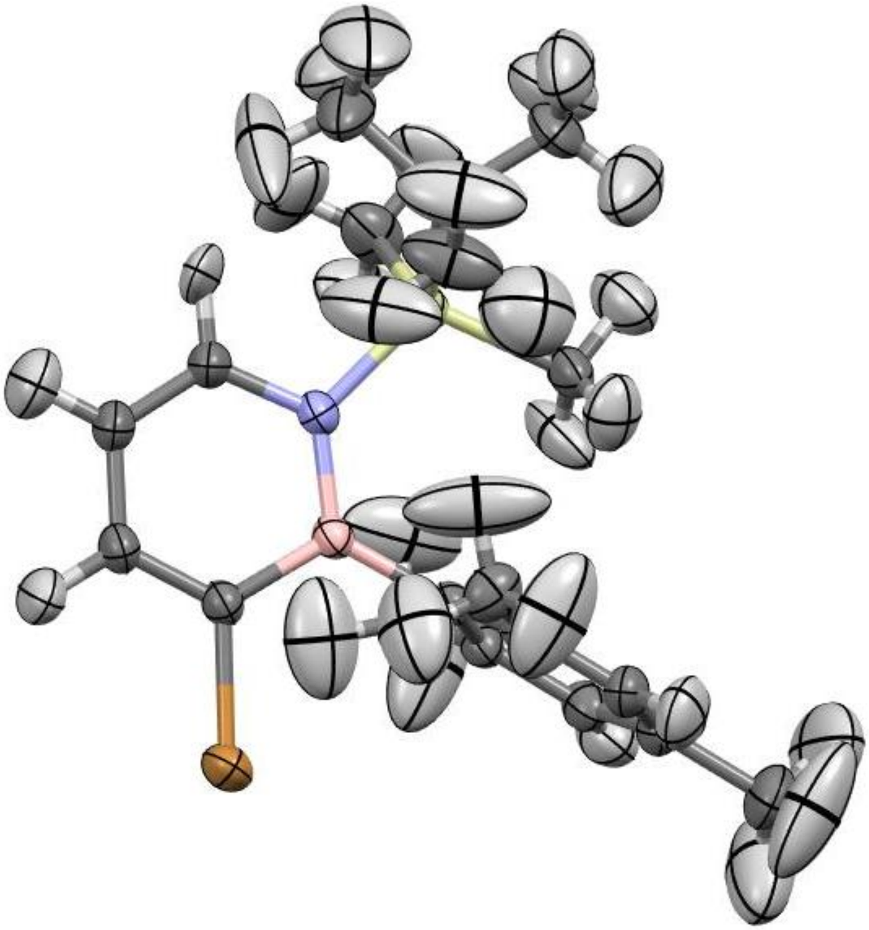

Figure S179. Crystal structure of **2**. Thermal ellipsoids are drawn at the 50% probability level.

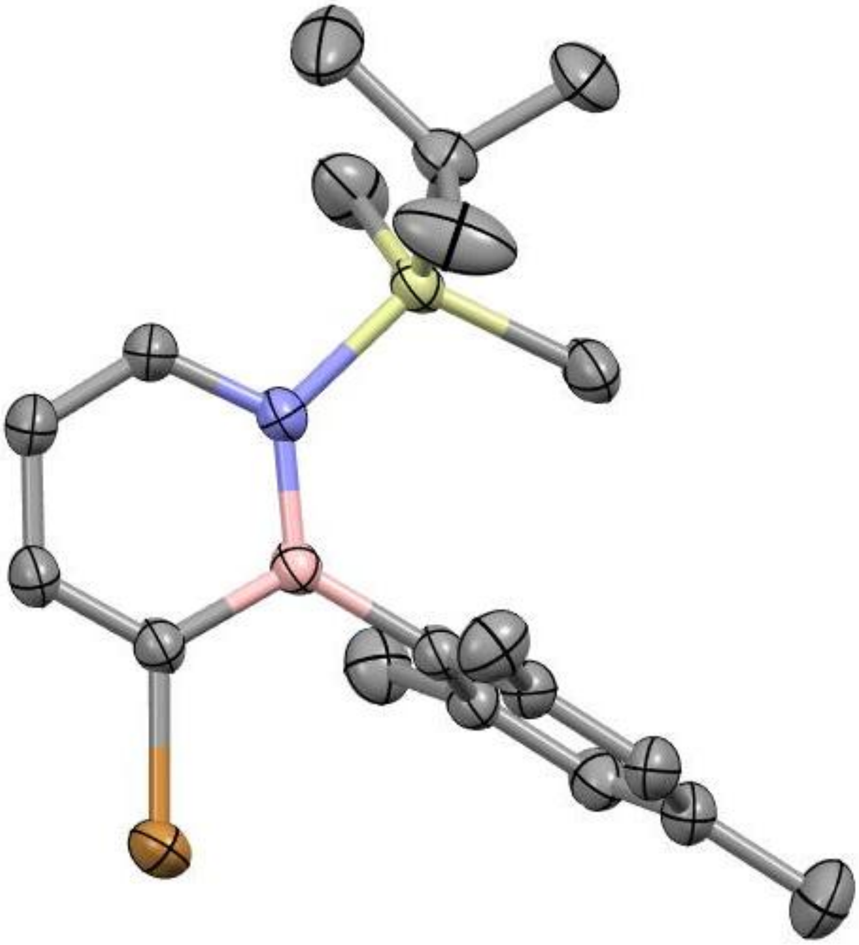

Figure S180. Crystal structure of **2**. Hydrogens are omitted for clarity and thermal ellipsoids are drawn at the 50% probability level.

Table S8. Parameters and results of the X-ray diffraction measurement of compound **3**.

Parameter	unit	3
Molecular formula		$C_{19}H_{28}BBr_2NSi$
CCDC		2341277
$D_{calc.}$	g cm^{-3}	1.437
μ	mm^{-1}	5.260
Molar weight	g/mol	469.158
Colour		clear colorless
Shape		block-shaped
Size	mm^3	0.23×0.16×0.09
T/K		150.00(10)
Crystal System		monoclinic
Space Group		$P2_1/c$
a/Å		15.5465(1)
b/Å		12.7242(1)
c/Å		22.8100(2)
α/°		90
β/°		106.072(1)
γ/°		90
V	Å^3	4335.84(6)
Z		8
Z'		2
Wavelength	Å	1.54184
Radiation type		Cu K_α
Θ_{min}	°	2.96
Θ_{max}	°	78.86
Measured Refl's.		137963
Indep't Refl's		9107
Refl's I≥2 σ(I)		8888
R_{int}		0.0196
Parameters		968
Restraints		0
Largest Peak		0.6432
Deepest Hole		-0.3347
GooF		1.0683
wR_2 (all data)		0.0400
wR_2		0.0397
R_1 (all data)		0.0176
R_1		0.0169

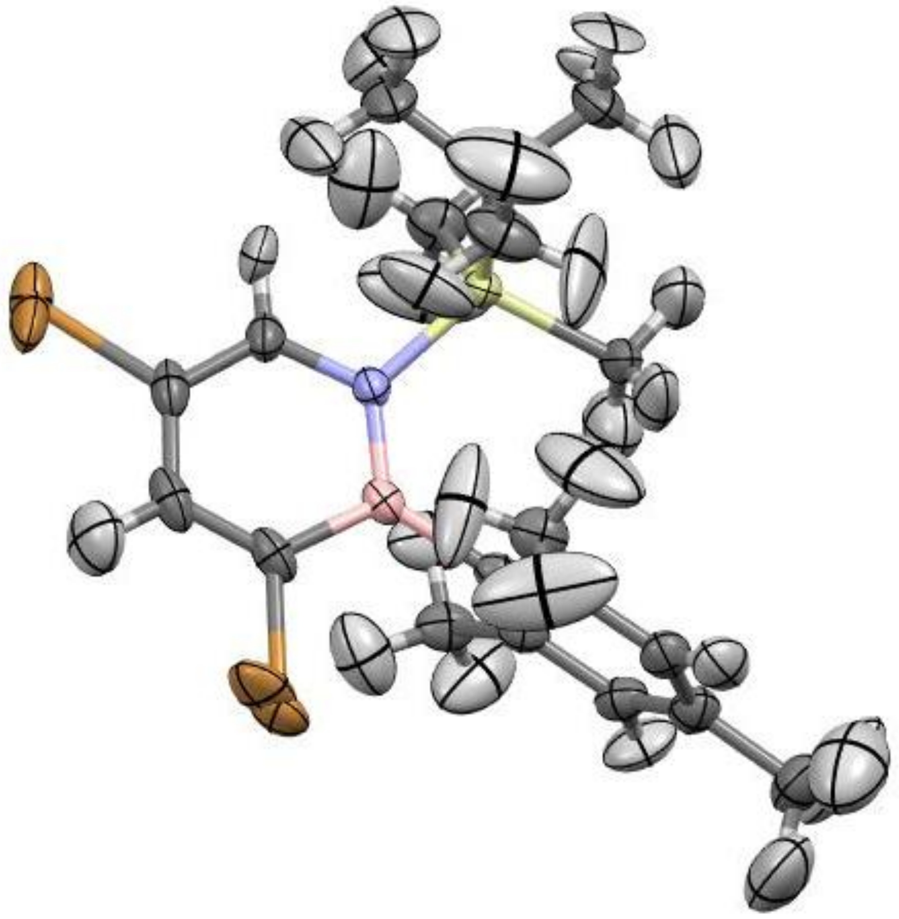

Figure S181. Crystal structure of **3**. Thermal ellipsoids are drawn at the 50% probability level.

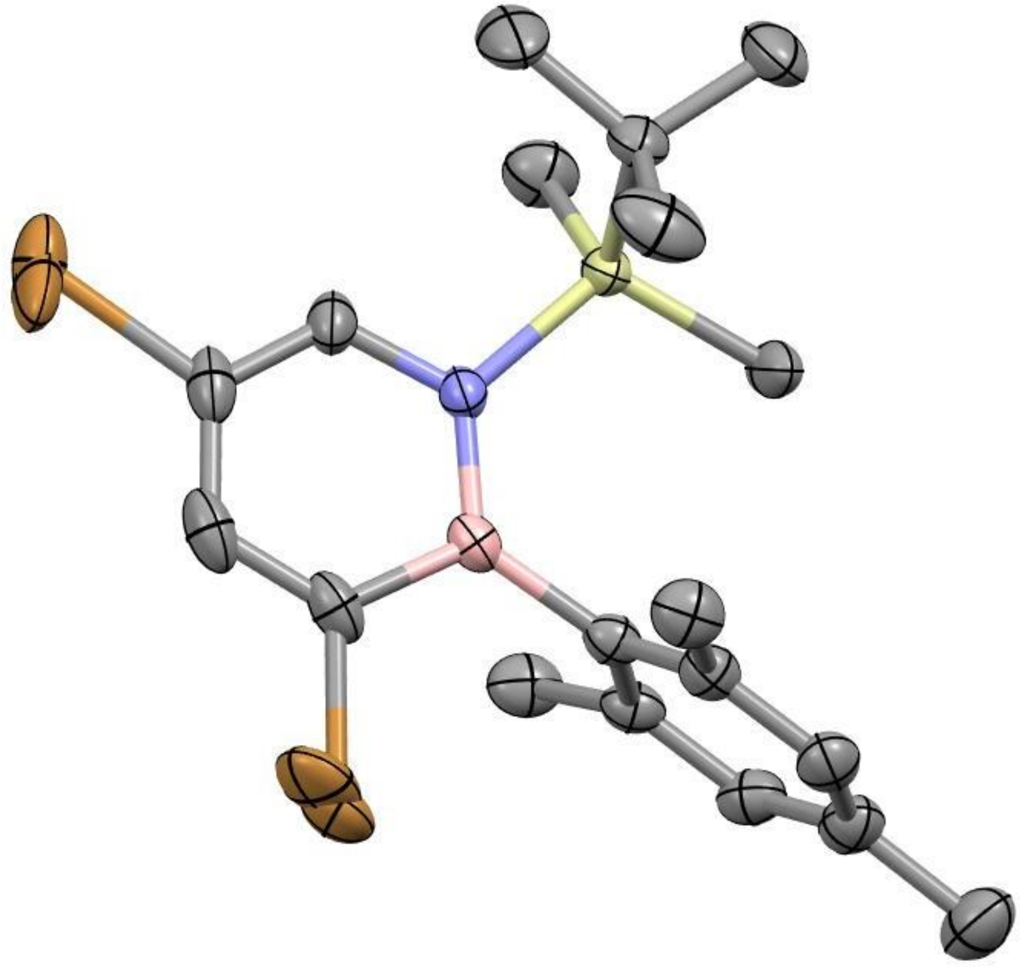

Figure S182. Crystal structure of **3**. Hydrogens are omitted for clarity and thermal ellipsoids are drawn at the 50% probability level.

Table S9. Parameters and results of the X-ray diffraction measurement of compound **4**.

Parameter	unit	4
Molecular formula		$C_{19}H_{28}BBr_2NSi$
CCDC		2350121
$D_{calc.}$	g cm^{-3}	1.448
μ	mm^{-1}	5.300
Molar weight	g/mol	469.158
Colour		clear colorless
Shape		plate-shaped
Size	mm^3	0.22×0.07×0.01
T/K		150.00(10)
Crystal System		monoclinic
Space Group		$P2_1/c$
a/Å		7.1663(1)
b/Å		15.6167(3)
c/Å		19.4040(4)
α/°		90
β/°		97.800(2)
γ/°		90
V	Å^3	2151.49(7)
Z		4
Z'		1
Wavelength	Å	1.54184
Radiation type		Cu K_α
Θ_{min}	°	3.65
Θ_{max}	°	80.04
Measured Refl's.		74913
Indep't Refl's		4640
Refl's I≥2 σ(I)		4367
R_{int}		0.0356
Parameters		469
Restraints		23
Largest Peak		0.9558
Deepest Hole		-0.6210
GooF		1.0677
wR_2 (all data)		0.0644
wR_2		0.0633
R_1 (all data)		0.0302
R_1		0.0281

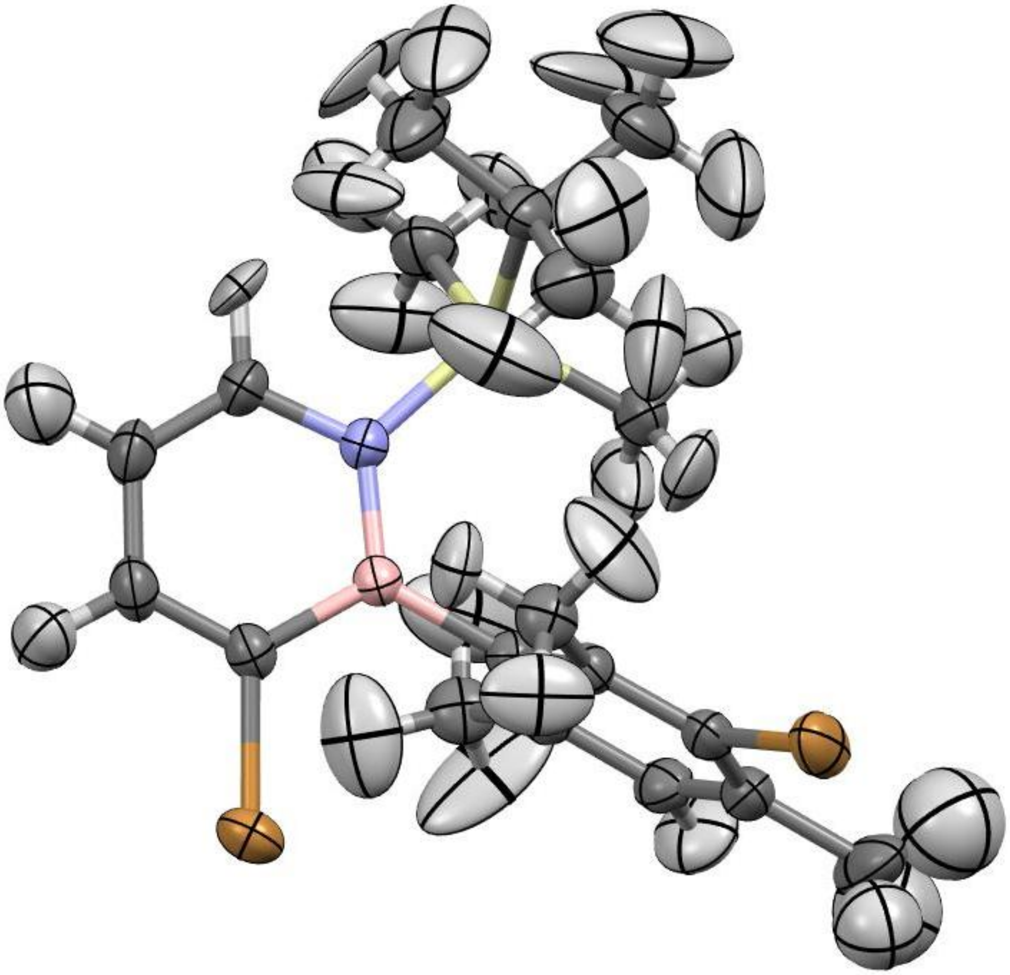

Figure S183. Crystal structure of **4**. Thermal ellipsoids are drawn at the 50% probability level.

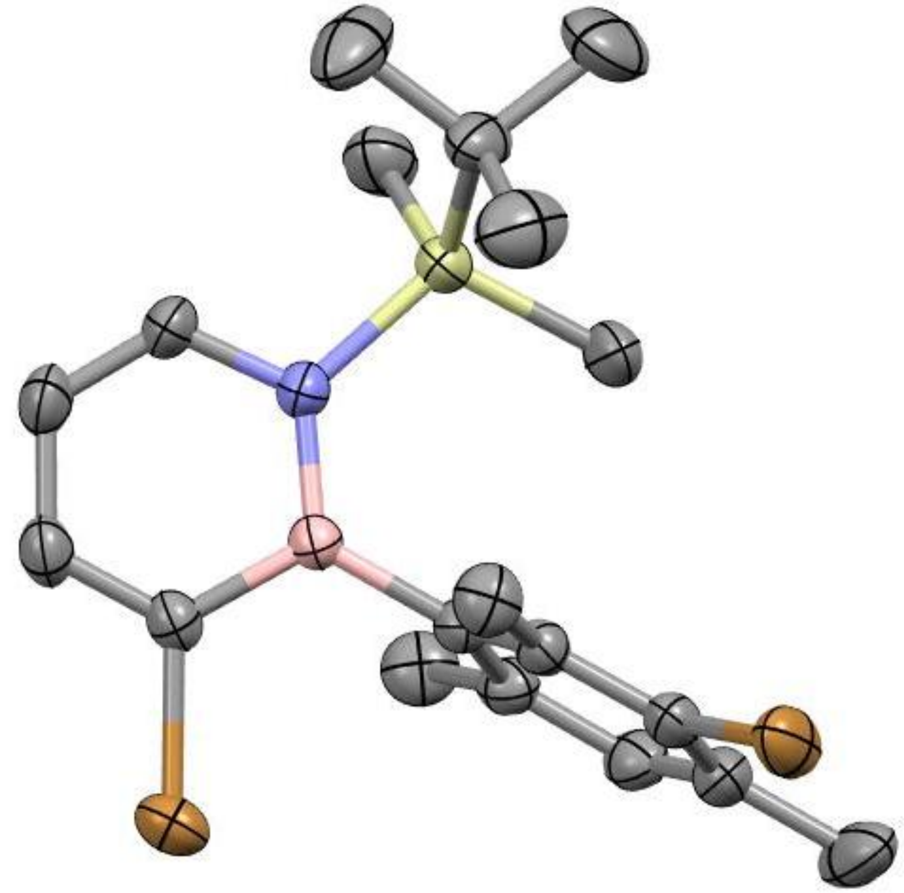

Figure S184. Crystal structure of **4**. Hydrogens are omitted for clarity and thermal ellipsoids are drawn at the 50% probability level.

Table S10. Parameters and results of the X-ray diffraction measurement of compound **5**.

Parameter	unit	5
Molecular formula		$C_{19}H_{28b}Br_3NSi$
CCDC		2348307
$D_{calc.}$	g cm^{-3}	1.627
μ	mm^{-1}	7.206
Molar weight	g/mol	548.053
Colour		clear colorless
Shape		block-shaped
Size	mm^3	0.18×0.16×0.13
T/K		150.00(10)
Crystal System		orthorhombic
Space Group		*Pbca*
a/Å		13.4858(1)
b/Å		16.3267(1)
c/Å		20.3238(1)
α/°		90
β/°		90
γ/°		90
V	Å^3	4474.87(5)
Z		8
Z'		1
Wavelength	Å	1.54184
Radiation type		Cu K□
Θ_{min}	°	4.35
Θ_{max}	°	79.11
Measured Refl's.		129273
Indep't Refl's		4786
Refl's I≥2 σ(I)		4750
R_{int}		0.0194
Parameters		470
Restraints		0
Largest Peak		0.6327
Deepest Hole		-0.7114
GooF		1.0348
wR_2 (all data)		0.0332
wR_2		0.0331
R_1 (all data)		0.0179
R_1		0.0176

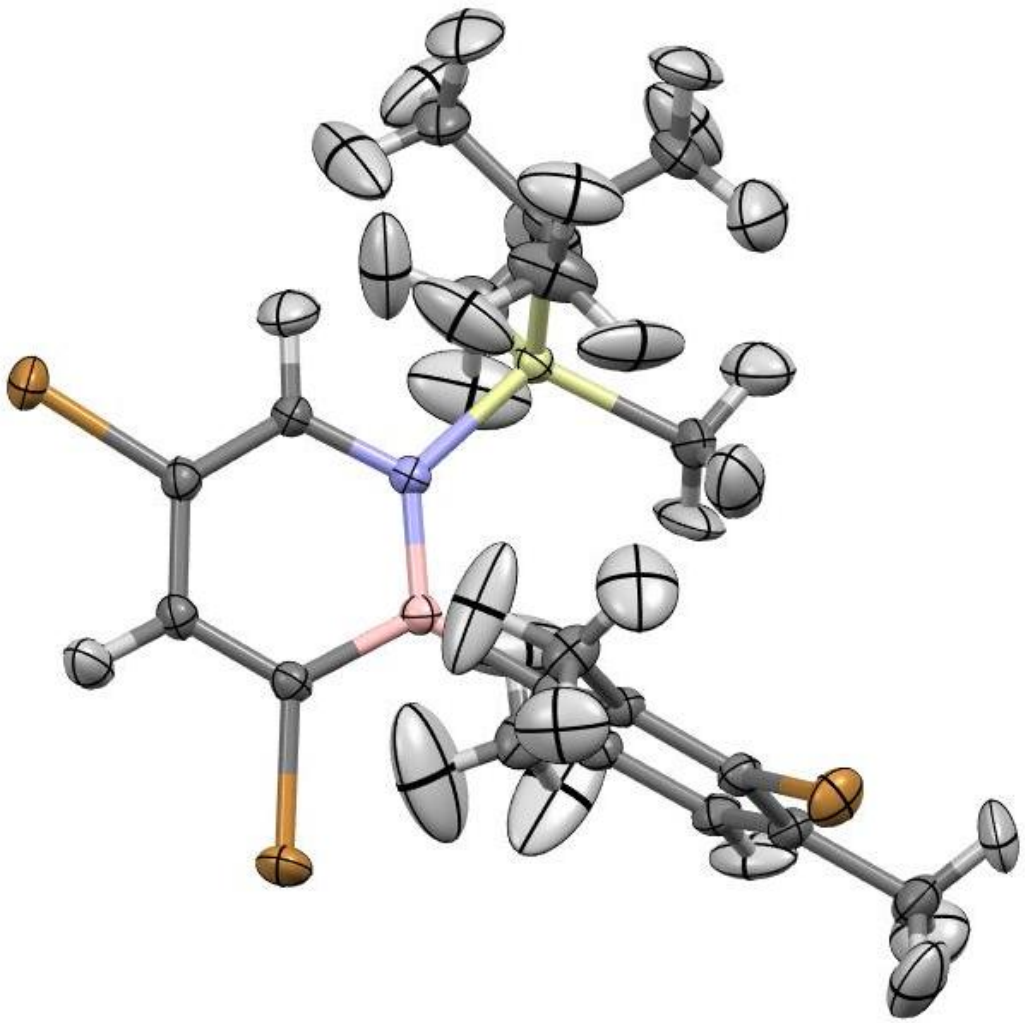

Figure S185. Crystal structure of **5**. Thermal ellipsoids are drawn at the 50% probability level.

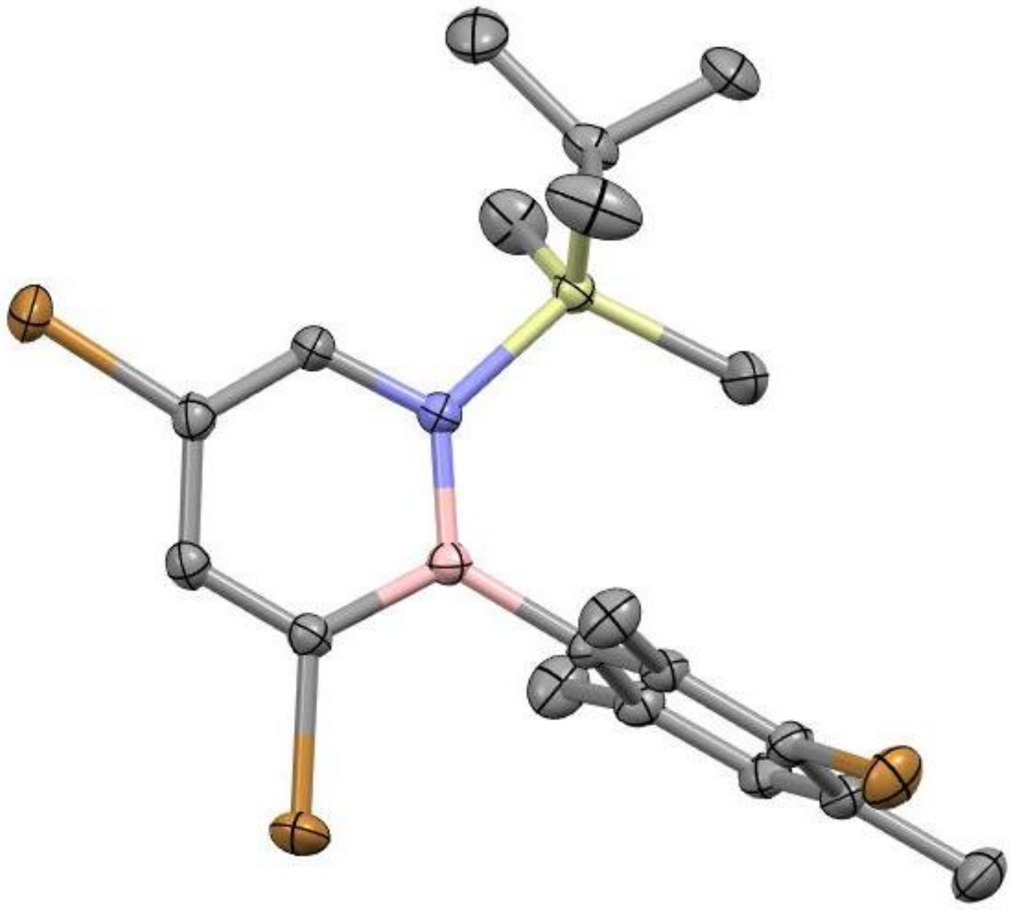

Figure S186. Crystal structure of **5**. Hydrogens are omitted for clarity and thermal ellipsoids are drawn at the 50% probability level.

9. GC-FID Calibrations

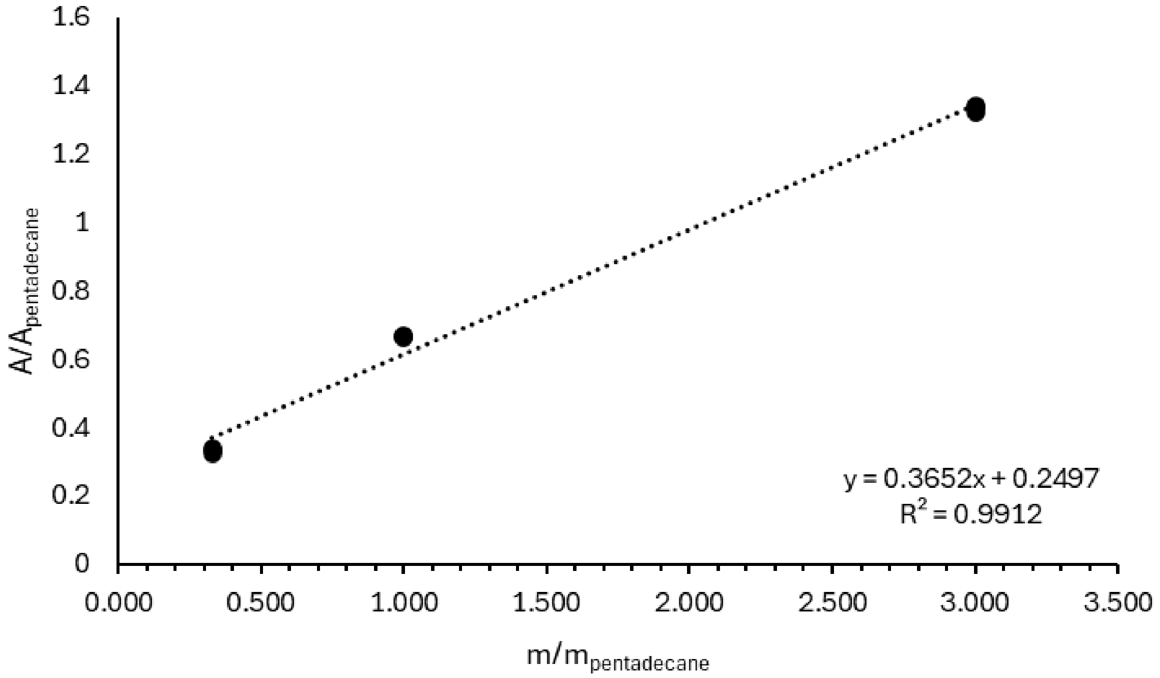

Figure S187. Calibration of **8a** with *n*-pentadecane on GC-FID.

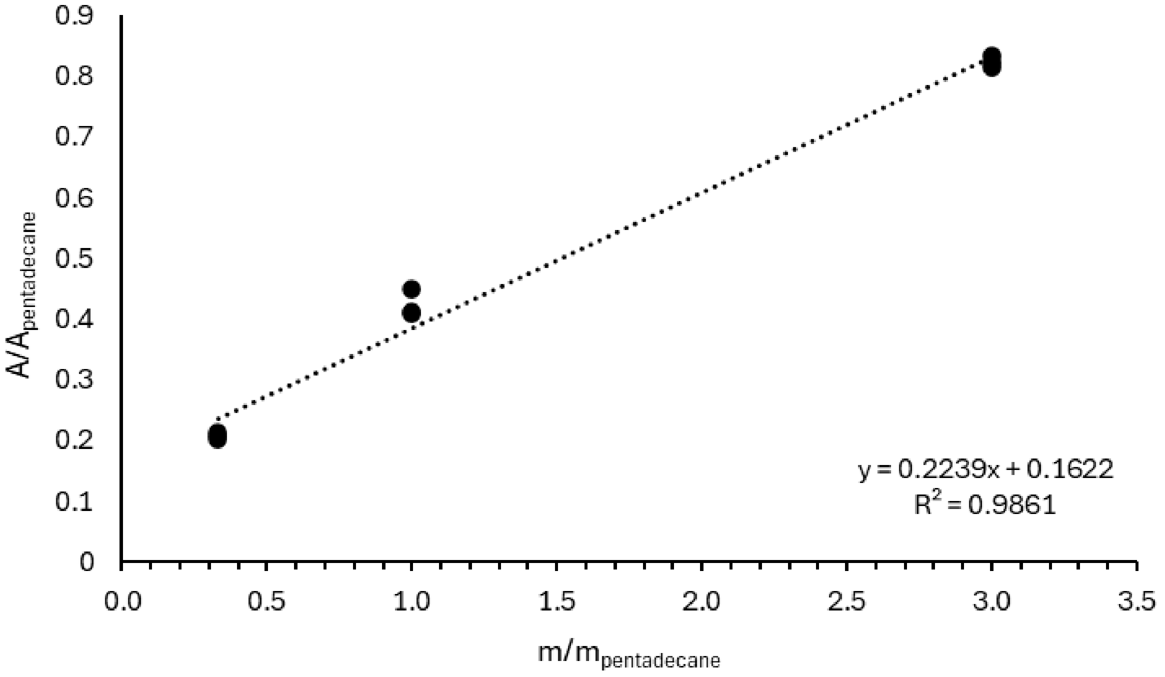

Figure S188. Calibration of **8b** with *n*-pentadecane on GC-FID.

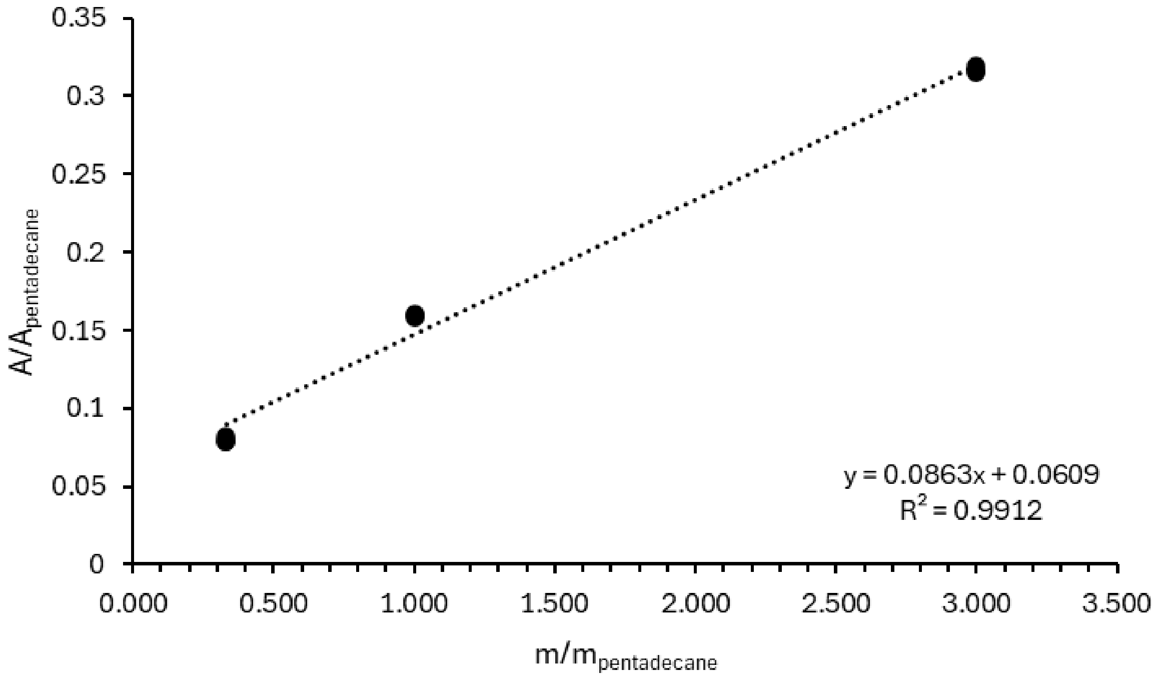

Figure S189. Calibration of **8c** with *n*-pentadecane on GC-FID.

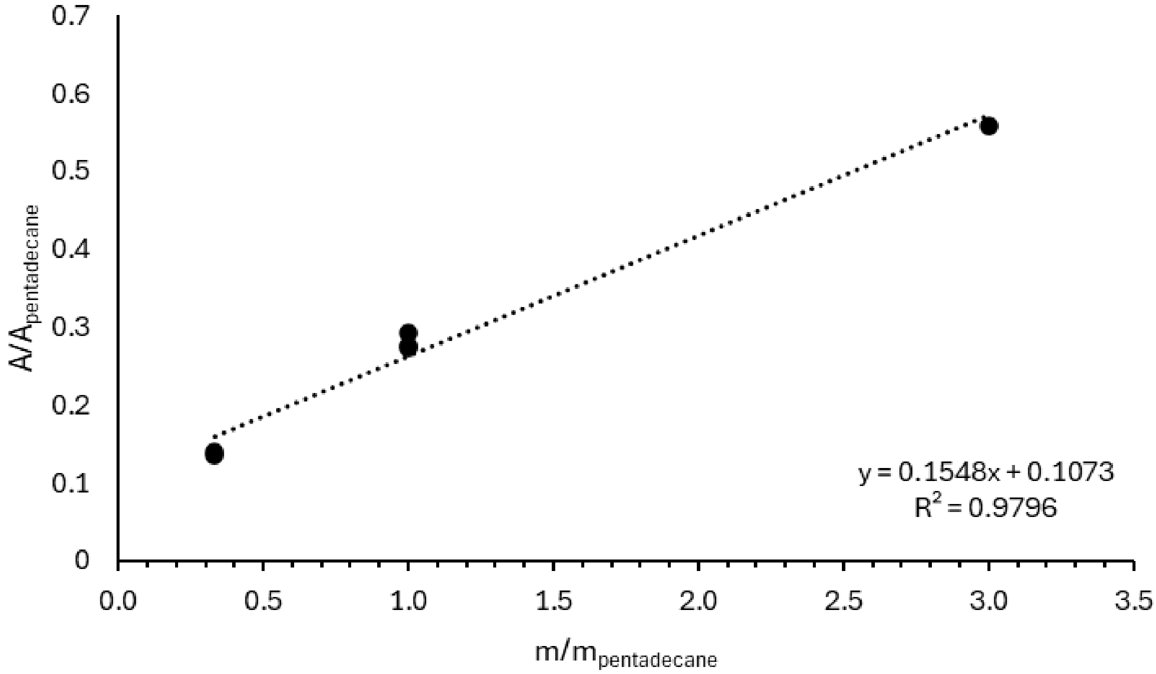

Figure S190. Calibration of **8e** with *n*-pentadecane on GC-FID.

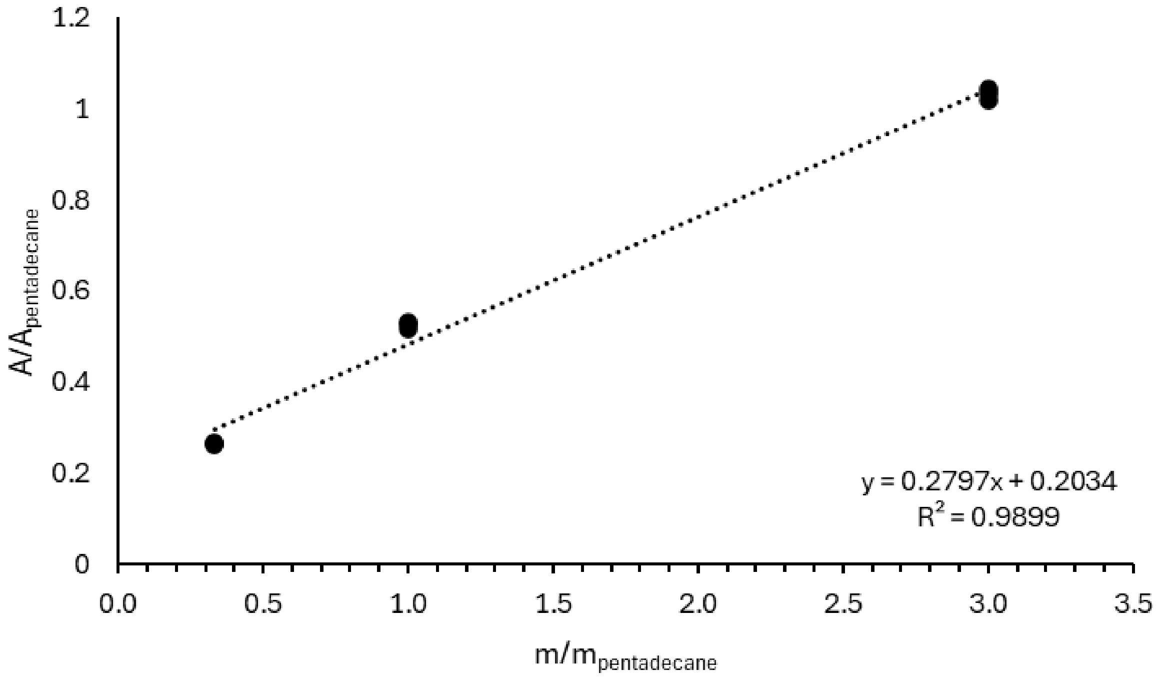

Figure S191. Calibration of **8f** with *n*-pentadecane on GC-FID.

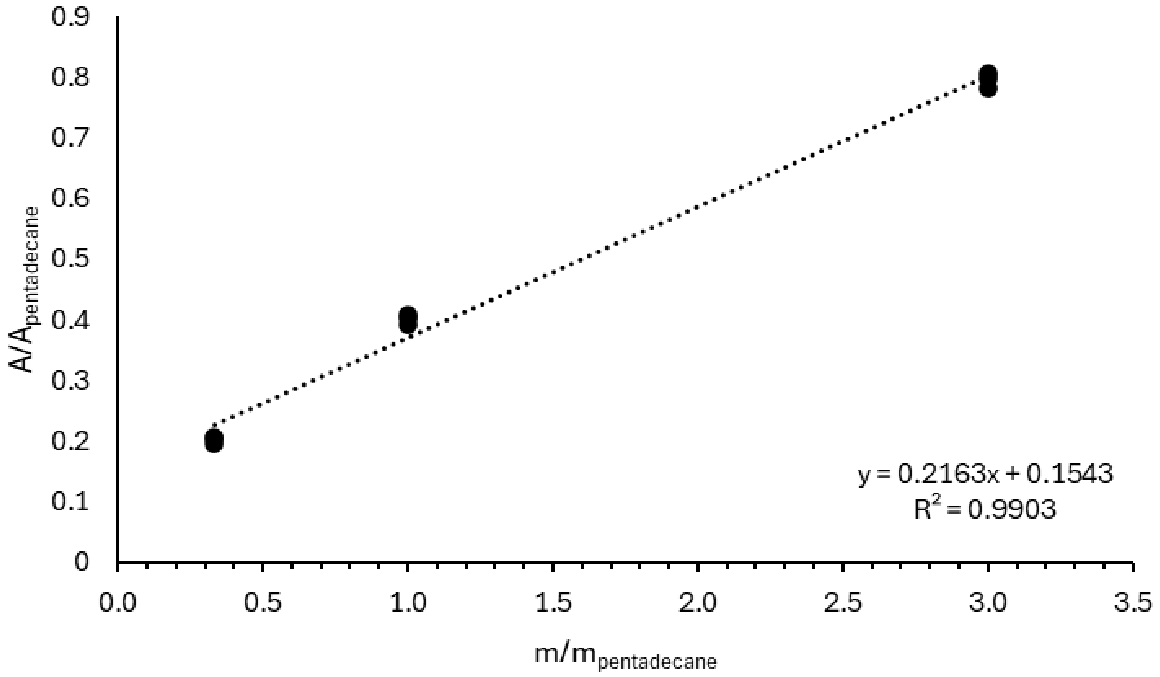

Figure S192. Calibration of **8g** with *n*-pentadecane on GC-FID.

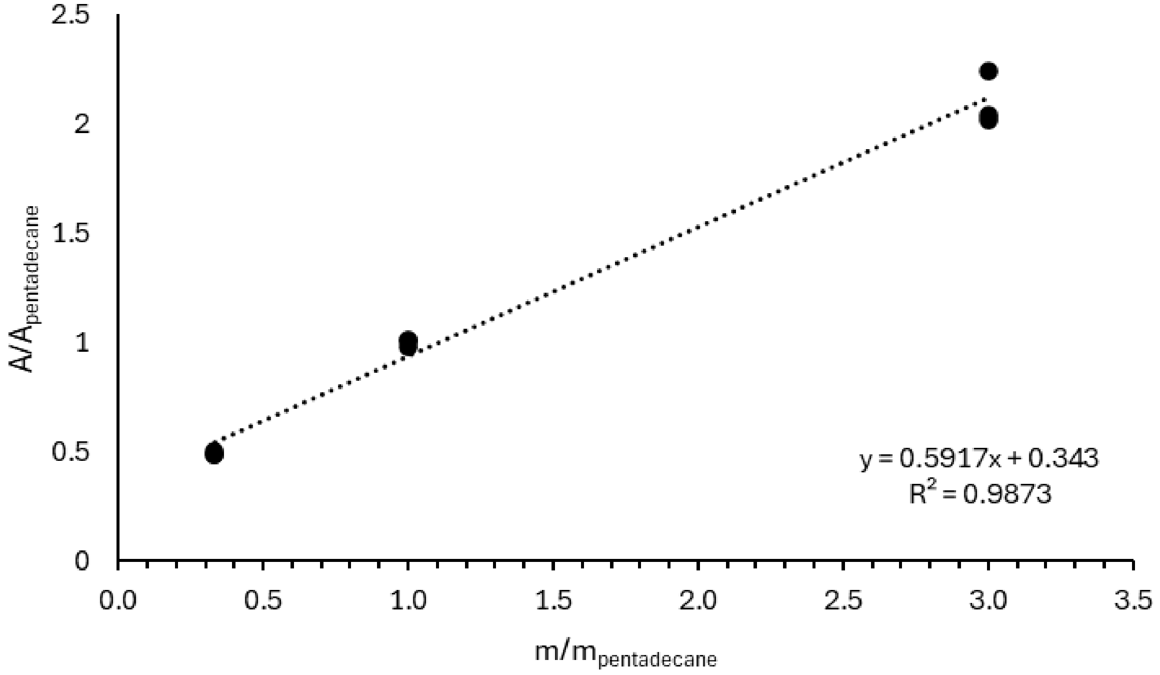

Figure S193. Calibration of **8h** with *n*-pentadecane on GC-FID.

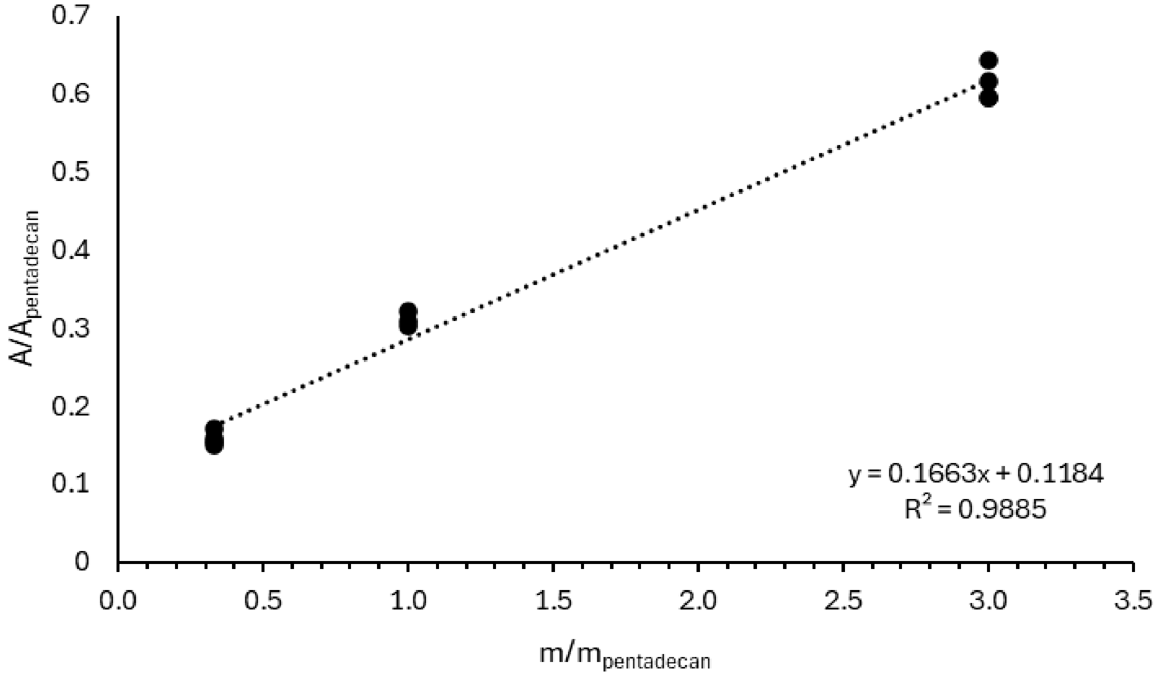

Figure S194. Calibration of **8j** with *n*-pentadecane on GC-FID.

8. Computations

Electronic structure computations: All geometries were optimized using the B3LYP functional[19-22] and the 6-311+G(d,p) basis set[24] in Gaussian 16.[25] The following coordinates are given in Å. Verification of the stationary points involved analytical frequency computations, confirming minima with zero imaginary vibrational frequencies and one imaginary vibrational frequency for transition states.

For a verification of the transitions states intrinsic reaction coordinates were calculated by using the local quadratic approximation.

Free energies were computed using standard thermochemistry equations implemented in Gaussian. In this section, total energies are given in Hartree/particle and kcal/mol.

The low-lying excited states were computed using time-dependent density functional theory employing the B3LYP functional (TD-B3LYP) as implemented[26] in Gaussian 16 in conjunction with the 6-311+G(d,p) basis set using the B3LYP/6-311+G(d,p) optimized geometries.

Relative energies of the optimized structures (B3LYP/6-311+G(d,p))

Table S11. Zero point vibrational energy (ZPVE) and Gibbs energy (G) of the optimized structures that occur during the thermal ring opening of **8a**, **8e** and **8k**. Relative Gibbs energies (G_{rel}) are given with respect to the corresponding *Dewar* isomers.

	ZPVE		Gibbs Energie (G)		$\Delta G^‡$	G_{rel}
	hartree/particle	kcal/mol	hartree/particle	kcal/mol	kcal/mol	kcal/mol
**8a**$_{\textbf{Dewar}}$	-1,342.05	-842,137.74	-1,342.11	-842,179.67		0.00
**8a**$_{\textbf{TS1}}$	-1,342.01	-842,114.64	-1,342.07	-842,154.54	25.13	25.13
**8a**$_{\textbf{IM}}$	-1,342.01	-842,115.85	-1,342.07	-842,155.56		24.11
**8a**$_{\textbf{TS2}}$	-1,342.01	-842,112.65	-1,342.07	-842,152.36	3.20	**27.31**
**8a**	-1,342.13	-842,189.30	-1,342.19	-842,228.66		-49.00
**8e**$_{\textbf{Dewar}}$	-1,475.98	-926,178.82	-1,476.05	-926,223.79		0.00
**8e**$_{\textbf{TS1}}$	-1,475.95	-926,160.05	-1,476.01	-926,203.10	20.69	20.69
**8e**$_{\textbf{IM}}$	-1,475.95	-926,161.63	-1,476.02	-926,204.86		18.93
**8e**$_{\textbf{TS2}}$	-1,475.94	-926,155.34	-1,476.01	-926,198.89	5.97	**24.90**
**8e**	-1,476.06	-926,230.64	-1,476.13	-926,273.60		-49.80
**8k**$_{\textbf{Dewar}}$	-1,854.75	-1,163,861.82	-1,854.82	-1,163,907.20		0.00
**8k**$_{\textbf{TS1}}$	-1,854.71	-1,163,838.93	-1,854.78	-1,163,882.60	24.61	24.61
**8k**$_{\textbf{IM}}$	-1,854.72	-1,163,840.26	-1,854.79	-1,163,883.76		23.44
**8k**$_{\textbf{TS2}}$	-1,854.71	-1,163,837.82	-1,854.78	-1,163,881.53	2.23	**25.67**
**8k**	-1,854.83	-1,163,913.97	-1,854.90	-1,163,957.36		-50.16

APT charges

Table S12. APT charges calculated for B, N, C3 and C6 during the thermal ring opening of **8a***Dewar*, **8e***Dewar* and **8k***Dewar*.

	APT charge			
	B	N	C3	C6
**8a**$_{\text{Dewar}}$	0.9	-1.2	**-0.3**	0.2
**8a**$_{\text{TS1}}$	0.6	-1.2	-0.2	0.4
**8a**$_{\text{IM}}$	0.1	-1.0	**0.1**	0.4
**8a**$_{\text{TS2}}$	0.2	-1.2	-0.4	0.7
**8a**	0.6	-0.9	-0.3	0.1
**8e**$_{\text{Dewar}}$	0.9	-1.2	**-0.2**	0.2
**8e**$_{\text{TS1}}$	0.5	-1.3	0.1	0.3
**8e**$_{\text{IM}}$	-0.1	-1.2	**0.7**	0.5
**8e**$_{\text{TS2}}$	0.0	-1.4	-0.2	1.0
**8e**	0.6	-0.9	-0.2	0.1
**8k**$_{\text{Dewar}}$	0.9	-1.2	**-0.3**	0.2
**8k**$_{\text{TS1}}$	0.6	-1.2	-0.3	0.4
**8k**$_{\text{IM}}$	0.1	-1.1	**-0.1**	0.5
**8k**$_{\text{TS2}}$	0.6	-1.2	-0.4	0.4
**8k**	0.6	-0.9	-0.3	0.1

3c2e bond

The electrons of the 2e3c bond reside in the highest occupied molecular orbital (HOMO) of **8a**$_{\mathbf{IM}}$ and **8k**$_{\mathbf{IM}}$. This HOMO is illustrated for **8a**$_{\mathbf{IM}}$. If only the negative IsoValues are plotted and a certain angle is chosen, one can perceive the increased electron density between the three atoms.

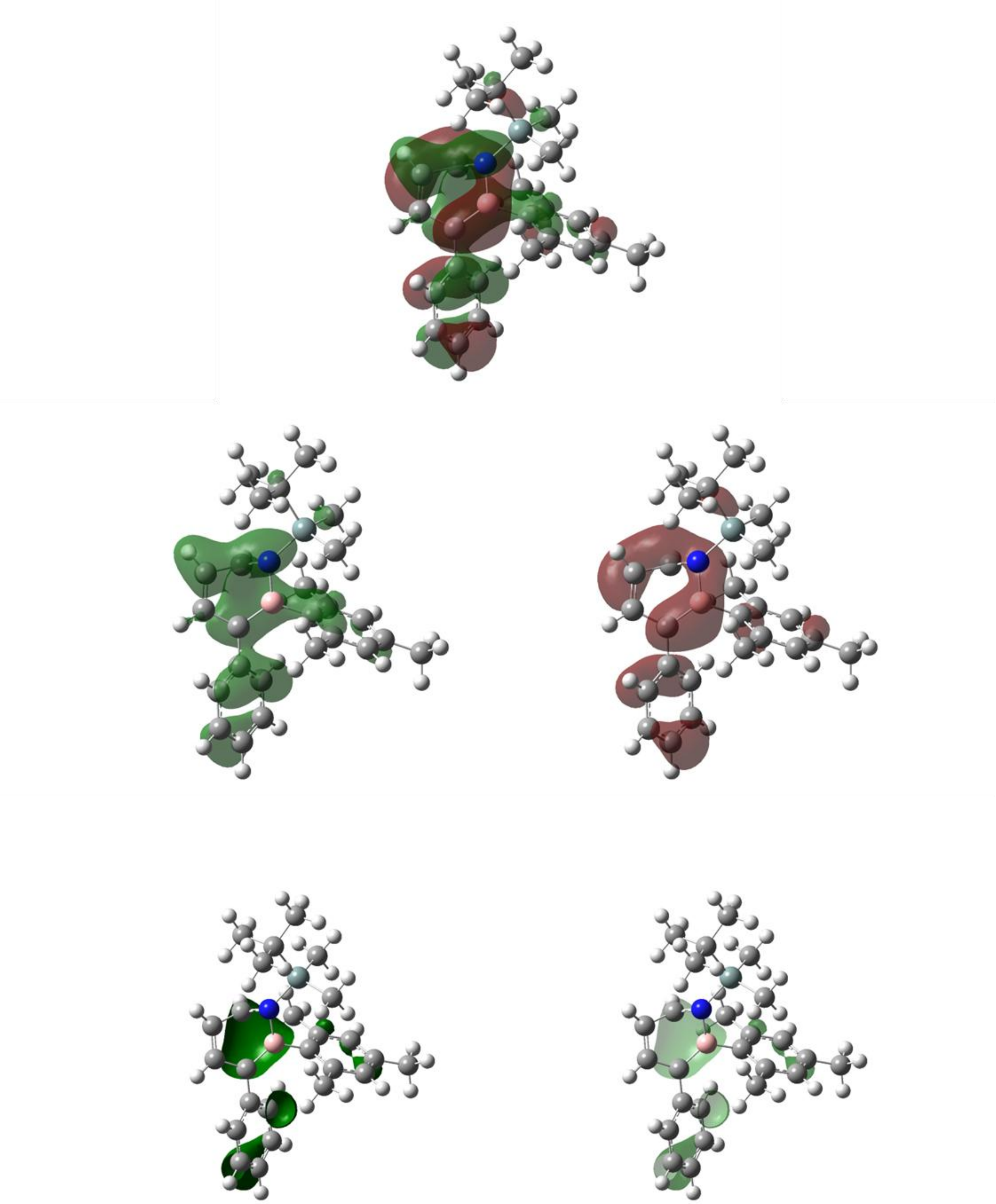

Figure S195. Illustration of the HOMO (2e3c bond) of **8a$_{IM}$**. Orbital lobes with positive IsoValues are shown in red, negative lobes are green.

Frontier orbitals of 8a, 8c, 8e and 8k

The longest wavelength absorption is due to an electronic transition S0 → S1 that is dominated by excitation of an electron from the highest occupied molecular orbital (HOMO) to the lowest unoccupied molecular orbital (LUMO) of the dihydroazaborinines. This process is qualitatively associated with charge transfer from the C3 substituent to the dihydroazaborinine ring. This transfer becomes more pronounced for substituents with stronger electronic effects. This may account for the experimentally observed bathochromic shift of the absorption maxima depending on the C3 substituent. For **8e** the strongest charge transfer is visible, while moderate effects are observed for **8c** and **8k**, and no effect can be qualitatively identified for **8a**.

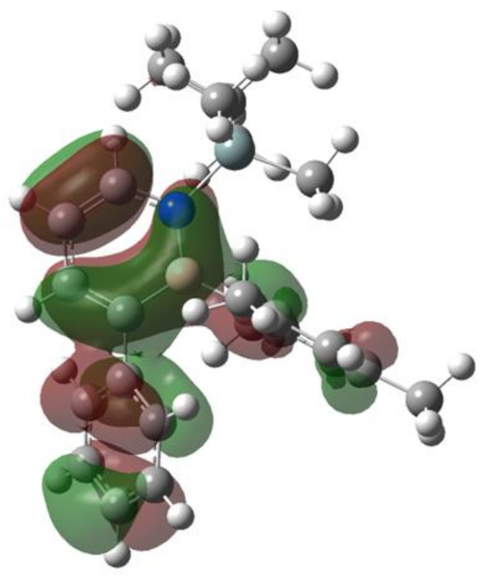

8a - HOMO

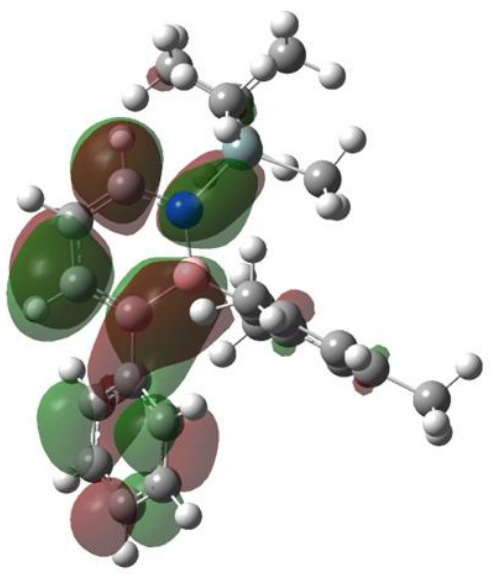

8a - LUMO

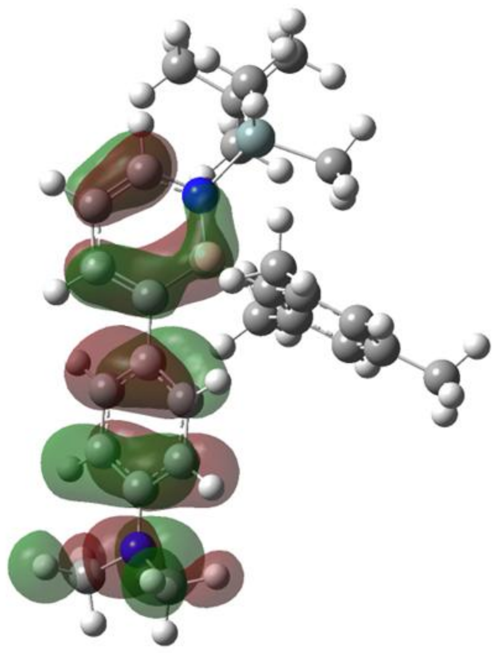

8e - HOMO

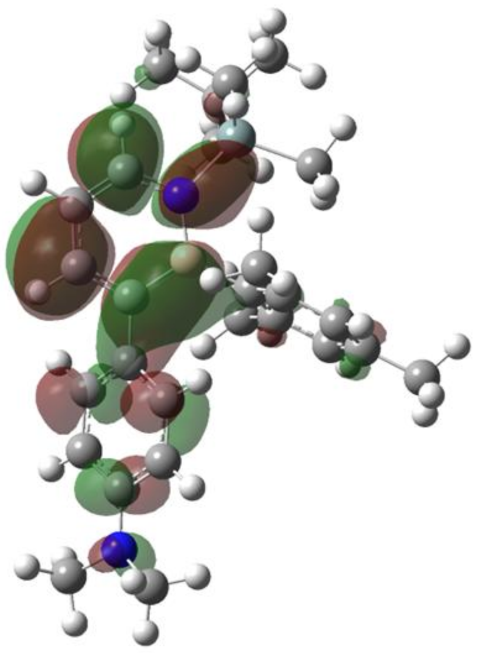

8e - LUMO

Figure S196. Frontier orbitals of the dihydroazaborinines **8a** and **8e**. Positive and negative IsoValues are shown with 60% transparency.

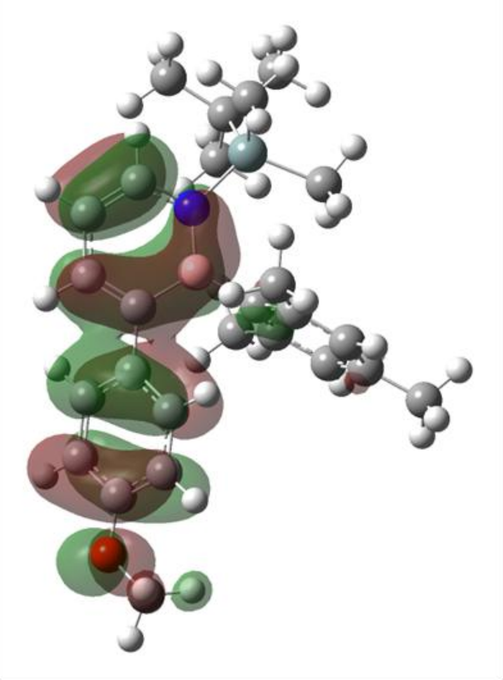

8c - HOMO

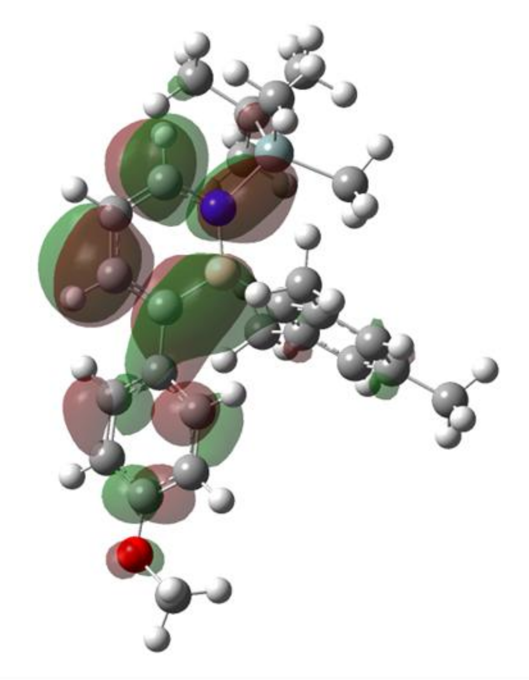

8c - LUMO

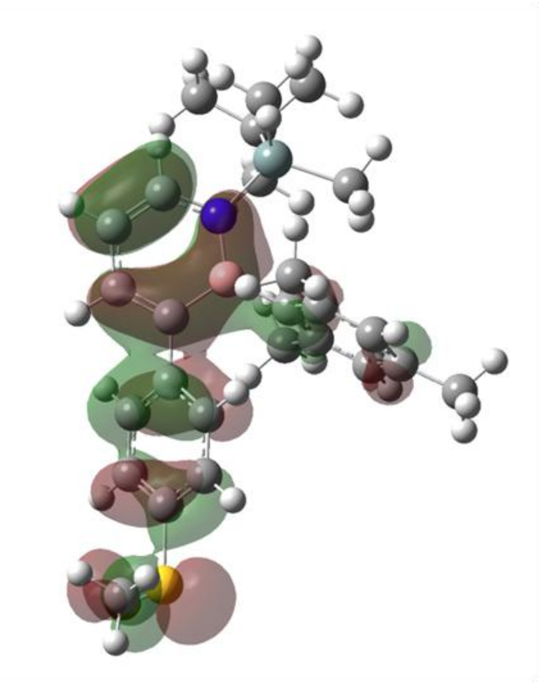

8k - HOMO

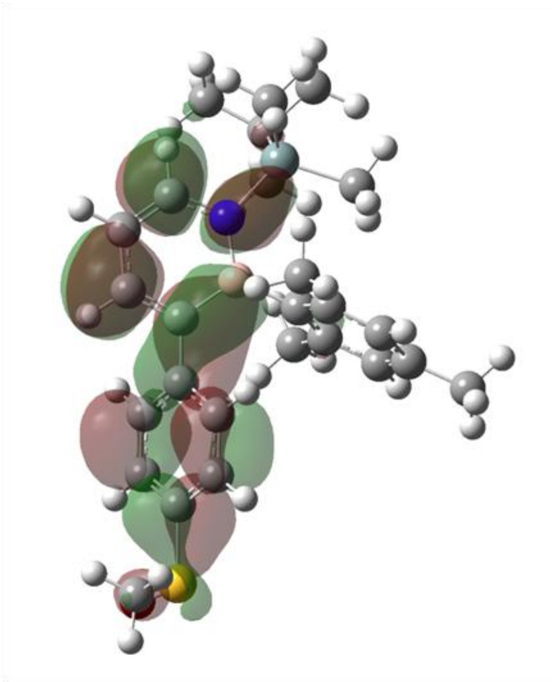

8k - LUMO

Figure S197. Frontier orbitals of the dihydroazaborinines **8c** and **8k**. Positive and negative IsoValues are shown with 60% transparency.

Absorption maxima

The UV-Vis absorption was calculated using TD- B3LYP/6-311+G(d,p).

Table 13. Measured absorption maximum λ_{max} (in nm) of the different C3 *p*-X-C6H4 substituted dihydroazaborinines in cyclohexane solution and calculated absorption maxima $\lambda_{max, clac}$ (in nm; TD-B3LYP/6-311+G(d,p)). The first row gives the *para* substituent of the phenyl ring attached to the C3, except for thiophene (8i), which is directly linked to the dihydroazaborinine C3.

X	**8a** *H*	**8b** *Me*	**8c** OMe	**8j** *Ph*	8i	**8e** NMe_2	**8f** *Cl*	**8g** CF_3	**8k** SOMe
λ_{max}	302	306	310	312	312	333	306	310	309
$\lambda_{max, calc}$	300	304	317	-	-	353	304	307	309

Coordinates 8a

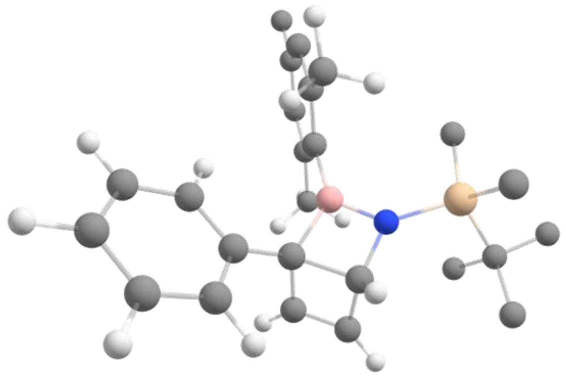

$8a_{Dewar}$

C	-0.21758300	-1.93863000	2.22640900
C	-0.70790900	-1.55890200	0.82910400
C	0.75032500	-1.94660600	0.37834000
C	1.02581900	-2.25707100	1.84470800
H	-0.72460600	-1.91837100	3.18510300
H	0.93490600	-2.71592300	-0.37190400
H	1.92294800	-2.53909600	2.38173100
C	-0.95213200	1.30272600	0.15951700
C	-1.64575000	1.64917000	-1.02068700
C	-0.95517200	2.21339300	1.23800600
C	-2.30084500	2.87996900	-1.10660400
C	-1.61779200	3.43709600	1.11612600
C	-2.29467200	3.79313400	-0.05116100
H	-2.83258700	3.13012200	-2.02059300
H	-1.60815700	4.12725900	1.95543400
C	-0.25591600	1.87594300	2.53756200
H	-0.71708700	1.01181400	3.02685700
H	0.79713900	1.62553300	2.37933500
H	-0.29891800	2.71295300	3.23758000
C	-1.69615600	0.70090000	-2.19950000
H	-0.69775600	0.50647000	-2.60426100
H	-2.12290300	-0.26625000	-1.91806200
H	-2.30581100	1.11052200	-3.00750500
C	-2.97826100	5.13349200	-0.17772700
H	-3.31461800	5.50344400	0.79390800
H	-2.29771500	5.88395600	-0.59555100
H	-3.84666300	5.07757300	-0.83877000
C	2.21383700	1.90247900	-1.25782100
H	1.28463700	2.06160000	-1.80948700
H	2.15301400	2.49663900	-0.34276900
H	3.03346100	2.30032600	-1.86363800
C	2.66074400	-0.87164900	-2.53334500
H	2.71638100	-1.95270000	-2.37778200
H	1.79841600	-0.67533300	-3.17731000

H	3.55810400	-0.57154100	-3.08284300
C	4.08023500	-0.17314400	0.15768500
C	3.87300000	0.40465900	1.57308600
H	4.78523700	0.28004500	2.17056900
H	3.64754100	1.47538400	1.54738300
H	3.05829300	-0.09441000	2.10370800
C	4.44714000	-1.66887400	0.26601800
H	5.34479700	-1.79285400	0.88518400
H	3.65278700	-2.26208800	0.72628600
H	4.66563800	-2.10642900	-0.71240000
C	5.25981300	0.56764300	-0.51204400
H	5.09046400	1.64666800	-0.56621300
H	6.17823900	0.41071700	0.06757800
H	5.45370200	0.20621900	-1.52697200
C	-1.96816700	-2.18835900	0.31008700
C	-1.96517500	-3.31220200	-0.52485100
C	-3.20737900	-1.64580600	0.68283500
C	-3.15957300	-3.87428400	-0.97615500
H	-1.02386300	-3.75827100	-0.82482100
C	-4.40082500	-2.21061900	0.24108100
H	-3.23174000	-0.76528600	1.31626000
C	-4.38245700	-3.32792200	-0.59398000
H	-3.13190100	-4.74332900	-1.62474800
H	-5.34653700	-1.77335800	0.54264700
H	-5.31081500	-3.76570500	-0.94320300
N	1.05224100	-0.54450300	-0.07524900
B	-0.23670100	-0.08541700	0.29418300
Si	2.49449200	0.07522100	-0.90678800

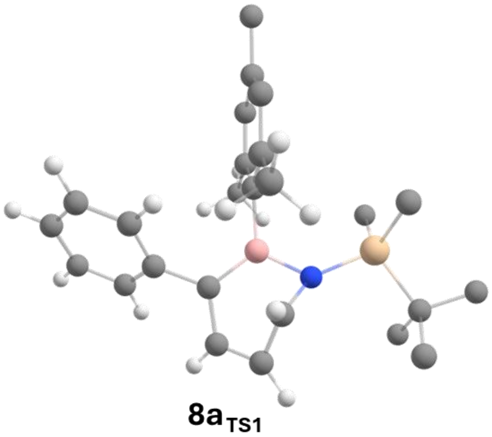

8a$_{TS1}$

C	0.87840900	0.95667400	0.02362000
C	0.98751800	1.80004300	-1.10398700
C	1.57157700	3.06424900	-0.98057800
C	2.05690500	3.53575100	0.23933400
C	1.95863700	2.69264400	1.34614700
C	1.38796100	1.42006400	1.25772300
H	1.65280100	3.69486500	-1.86192300
H	2.33781700	3.03252400	2.30615000
C	0.91523500	-1.81830600	0.01934000
C	0.14726000	-3.07033000	0.14691600
C	-1.01388300	-2.93869500	-0.50362400
H	0.46094700	-3.91735300	0.74720300
H	-1.92411600	-3.51890600	-0.40524100
B	0.16335100	-0.46563300	-0.01749700
N	-1.30952500	-0.54344300	-0.28410100
Si	-2.73617200	0.48019400	-0.06940100
C	-3.07772300	1.51235500	-1.61609200
H	-4.02205400	2.05678600	-1.51993100
H	-2.28789100	2.25236800	-1.76756400
H	-3.14252000	0.90115900	-2.52110800
C	-4.26704000	-0.63296700	0.29210200
C	-2.37589100	1.61873600	1.38130100
H	-2.20721400	1.06164700	2.30628200
H	-1.48831000	2.22531400	1.18849800
H	-3.21625300	2.29910800	1.54834300
C	-4.01755700	-1.50947700	1.53663000
H	-3.85766700	-0.90320300	2.43335600
H	-4.88757400	-2.15041900	1.72872900
H	-3.14552400	-2.15619800	1.41515100
C	-5.49620600	0.26470800	0.56189300
H	-5.34643100	0.91919800	1.42558900
H	-5.74835400	0.89298900	-0.29735300
H	-6.37395500	-0.35797900	0.77580400
C	-4.58232200	-1.53716700	-0.91810000
H	-5.45416700	-2.16815400	-0.70310100
H	-4.81737700	-0.95434900	-1.81369300
H	-3.75490900	-2.20847600	-1.16507600

C	-0.92913400	-1.59055500	-1.11534100
H	-0.68061300	-1.43482800	-2.16505400
C	0.54709300	1.34440800	-2.47942300
H	1.21947100	0.57719800	-2.88064300
H	-0.45649700	0.91719500	-2.46888000
H	0.54805100	2.17603100	-3.18740300
C	1.34399300	0.55698000	2.50115100
H	0.35450900	0.12128400	2.66357200
H	2.05203100	-0.27447600	2.42873700
H	1.60367700	1.13900500	3.38819000
C	2.64502900	4.92084500	0.36338800
H	3.42090500	4.95706800	1.13216500
H	3.08604700	5.25303400	-0.57961800
H	1.87675600	5.65154500	0.64047600
C	2.39126100	-1.92846500	-0.08554800
C	3.07032500	-2.92972700	0.63966500
C	3.17417000	-1.06117500	-0.86672800
C	4.45707100	-3.03328400	0.61000900
H	2.51287900	-3.62422900	1.25614000
C	4.56016300	-1.17855200	-0.91313000
H	2.69387000	-0.29127700	-1.45230400
C	5.21316300	-2.16037400	-0.17077000
H	4.94731800	-3.80414600	1.19475200
H	5.13144100	-0.49689800	-1.53382900
H	6.29314900	-2.24816400	-0.20399800

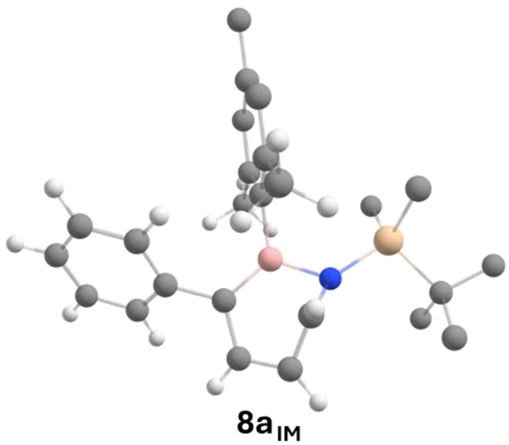

8a$_{IM}$

C	0.72177500	0.92695300	-0.15048300
C	0.83299000	1.75992300	-1.28755600
C	1.22397800	3.09562500	-1.14469700
C	1.52012000	3.65087300	0.10020900
C	1.44252200	2.81636400	1.21475400
C	1.06058100	1.47589200	1.10856000
H	1.30821800	3.71575700	-2.03322600
H	1.69504800	3.21482500	2.19390000
C	1.22389100	-1.79698200	-0.18936600
C	0.57006800	-3.06929800	-0.44840200
C	-0.65461400	-2.90096600	-0.97704400
H	0.97667300	-4.02752900	-0.14543100
H	-1.45488100	-3.63484500	-0.97137200
B	0.26452600	-0.60182100	-0.20831300
N	-1.31848600	-0.82856200	-0.07321800
Si	-2.68698000	0.28128100	0.19550300
C	-2.88534700	1.55767200	-1.18293800
H	-3.77640500	2.16897400	-1.00925200
H	-2.02404500	2.22934300	-1.20884600
H	-2.99124400	1.10098900	-2.17097600
C	-4.24635900	-0.85465800	0.25876400
C	-2.44593600	1.15960900	1.83841600
H	-2.26732000	0.45675200	2.65543200
H	-1.60193000	1.85063500	1.79326800
H	-3.33830300	1.74241200	2.08604000
C	-4.11036800	-1.89323600	1.39141500
H	-4.02474600	-1.41655100	2.37251000
H	-4.99713700	-2.53944600	1.41979000
H	-3.23346400	-2.53068300	1.25459500
C	-5.49456700	0.01569100	0.52897900
H	-5.42582800	0.55208900	1.47991300
H	-5.66546900	0.75188100	-0.26200500
H	-6.38816400	-0.61902600	0.57960400
C	-4.44607000	-1.59170700	-1.08112200
H	-5.34618400	-2.21802500	-1.03614500
H	-4.57589800	-0.89854100	-1.91780100

H	-3.60768200	-2.25236700	-1.31854400
C	-0.92360700	-1.45296700	-1.22533900
H	-1.06141500	-1.04601500	-2.22677800
C	0.57817600	1.24165800	-2.68790300
H	1.05881500	0.27425800	-2.85416700
H	-0.49022600	1.11224600	-2.88815400
H	0.96173600	1.94106900	-3.43407800
C	1.08205400	0.62142200	2.35851000
H	0.25535500	-0.09010900	2.38848700
H	2.00753800	0.03754300	2.40877700
H	1.03377300	1.24027100	3.25766700
C	1.89902100	5.10598200	0.23739100
H	2.58473800	5.26272900	1.07380100
H	2.37906200	5.48070400	-0.67001500
H	1.01500500	5.72752300	0.41961900
C	2.69018300	-1.76258700	-0.04534700
C	3.37168900	-2.86597000	0.51414900
C	3.46930600	-0.65459400	-0.43174300
C	4.74892700	-2.84888100	0.70122300
H	2.81596300	-3.73715500	0.83770000
C	4.85076700	-0.65179700	-0.26894200
H	2.98969800	0.20572000	-0.87504200
C	5.50048200	-1.74346500	0.30456700
H	5.23724700	-3.70517100	1.15350500
H	5.42177200	0.21221100	-0.59076400
H	6.57621100	-1.73471400	0.43870100

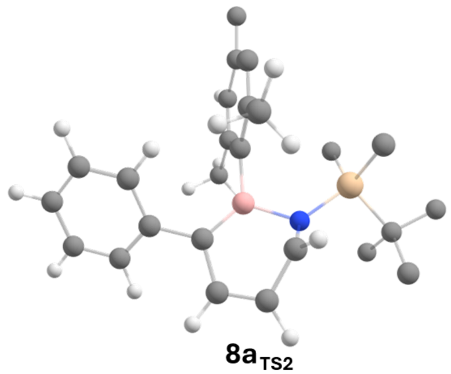

8a$_{TS2}$

C	-0.69677700	0.92950700	0.08585000
C	-0.75984300	1.74234600	1.24122500
C	-1.08992300	3.09570600	1.12472200
C	-1.37902400	3.68098100	-0.10937700
C	-1.35294700	2.86320500	-1.23912400
C	-1.02155100	1.50764700	-1.16171800
H	-1.14024100	3.70441200	2.02349600
H	-1.60264500	3.28868300	-2.20738600
C	-1.26908600	-1.77353200	0.20456200
C	-0.63063200	-3.07768900	0.37313500
C	0.60905700	-3.05660000	0.87662300
H	-1.09631900	-4.00235700	0.04904000
H	1.32117200	-3.87648500	0.86612400
B	-0.34257900	-0.61430400	0.14416600
N	1.28010500	-0.84992600	0.17133700
Si	2.67693800	0.28226000	-0.02145800
C	3.00859100	1.26160200	1.55586800
H	3.95489400	1.80331000	1.46283700
H	2.22208600	2.00088200	1.72209500
H	3.08168100	0.63418300	2.44832100
C	4.16171800	-0.89021500	-0.39969900
C	2.38283200	1.44442700	-1.46090100
H	2.10158800	0.90999200	-2.37079000
H	1.59998400	2.17020500	-1.23913100
H	3.30435500	1.99662900	-1.66958000
C	3.87711900	-1.73263700	-1.66130100
H	3.71658100	-1.10501300	-2.54279300
H	4.73216300	-2.38515100	-1.87808900
H	2.99543200	-2.36647600	-1.53871000
C	5.42132700	-0.02854100	-0.64965300
H	5.29868700	0.64447600	-1.50269700
H	5.69221500	0.57541300	0.22151600
H	6.27674700	-0.67817200	-0.87121500
C	4.44819100	-1.83498600	0.78427800
H	5.31538200	-2.46747000	0.55750200
H	4.67710100	-1.28957800	1.70483000
H	3.61367100	-2.51245000	0.98921600
C	1.10980300	-1.68278600	1.18579600
H	1.46757400	-1.43877700	2.19389600

C	-0.54864000	1.16447500	2.62441900
H	-1.21885500	0.31890000	2.80408700
H	0.47022000	0.79675800	2.77251300
H	-0.73818600	1.91663600	3.39331600
C	-1.07611400	0.67056200	-2.42041700
H	-0.25683800	-0.05007400	-2.47277700
H	-2.00543900	0.09262000	-2.45570700
H	-1.03924100	1.29922500	-3.31328600
C	-1.69984500	5.15227400	-0.21787100
H	-2.17787500	5.52442900	0.69161400
H	-0.79020900	5.74325800	-0.37379300
H	-2.36735800	5.35358100	-1.05918000
C	-2.73694300	-1.70899300	0.06113300
C	-3.45291300	-2.77082500	-0.53524100
C	-3.49775400	-0.60423900	0.49556400
C	-4.83345700	-2.72304300	-0.70022500
H	-2.92156400	-3.64088500	-0.90188400
C	-4.87901800	-0.55977100	0.33595900
H	-3.00071900	0.22705800	0.97511700
C	-5.56155800	-1.61685600	-0.26493800
H	-5.34212100	-3.55618000	-1.17407300
H	-5.42650000	0.30690400	0.69158800
H	-6.63806500	-1.58123700	-0.38781900

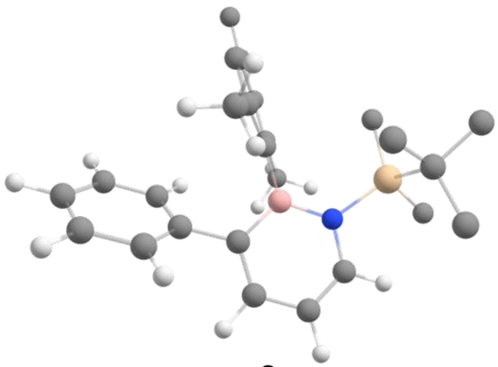

8a

C	-0.59952400	-3.02247400	-0.81962100
C	-1.10393000	-1.79196900	-0.45868400
C	1.64331500	-2.16914400	-1.04280500
C	0.76690100	-3.20784600	-1.13882100
H	-1.26155000	-3.88269500	-0.89103500
H	2.68532900	-2.33455200	-1.28517800
H	1.13196500	-4.17508300	-1.46133000
C	-0.64943700	0.88287500	-0.16568900
C	-0.85423800	1.74099800	-1.26957300
C	-1.01905000	1.34556900	1.11517200
C	-1.37416700	3.02369000	-1.07724500
C	-1.52263500	2.63966000	1.27558200
C	-1.70294300	3.50009400	0.19249900
H	-1.53020100	3.66516200	-1.94065800
H	-1.79477000	2.97855400	2.27167700
C	-0.92381200	0.44664800	2.32841000
H	-1.71225900	-0.31285700	2.30872200
H	0.02964300	-0.08332500	2.37631200
H	-1.03726800	1.01780000	3.25275900
C	-0.53012800	1.29267200	-2.68058100
H	0.54996300	1.23453400	-2.85334600
H	-0.93380700	0.29879400	-2.89542100
H	-0.94041500	1.98805000	-3.41619000
C	-2.22340800	4.90377600	0.38949200
H	-2.91021300	4.95915400	1.23777900
H	-1.40375500	5.60388900	0.58703000
H	-2.75099200	5.26180600	-0.49800200
C	2.31002000	2.00482400	-0.17240800
H	1.72486400	2.41542600	-0.99515900
H	1.75467900	2.19138700	0.74711000
H	3.25023400	2.56370900	-0.12412100
C	3.73686600	0.09021200	-2.04770300
H	4.16428500	-0.89513800	-2.24519900
H	3.10613800	0.35367900	-2.90192300
H	4.56409400	0.80588700	-2.01964700
C	3.79933800	-0.42287900	1.05468200
C	2.94842700	-0.45040800	2.34060900
H	3.56887200	-0.74584900	3.19596600

H	2.51932800	0.52817300	2.57389400
H	2.12920800	-1.17078800	2.26732400
C	4.40338300	-1.82685800	0.83888600
H	5.01148500	-1.88370200	-0.06933400
H	5.05967900	-2.07754000	1.68161400
H	3.63703200	-2.60334000	0.78719800
C	4.96890400	0.57128200	1.25492100
H	4.62134200	1.57784600	1.50027000
H	5.60015100	0.23413100	2.08600400
H	5.61054800	0.64068100	0.37077000
C	-2.55049600	-1.71616200	-0.10841100
C	-3.40547600	-0.74671100	-0.65529300
C	-3.10968000	-2.65011300	0.78123900
C	-4.76104500	-0.72018900	-0.33623400
H	-3.00938900	-0.01203700	-1.34365800
C	-4.46399300	-2.62197200	1.10587200
H	-2.46683700	-3.39541500	1.23738200
C	-5.29825400	-1.65576900	0.54660500
H	-5.39970100	0.03544100	-0.78084100
H	-4.86533900	-3.35141100	1.80131600
H	-6.35269100	-1.62994400	0.79799300
N	1.28281200	-0.89122200	-0.65921400
B	-0.13011600	-0.59751500	-0.41064900
Si	2.74368700	0.19906200	-0.44269500

IRC 8a

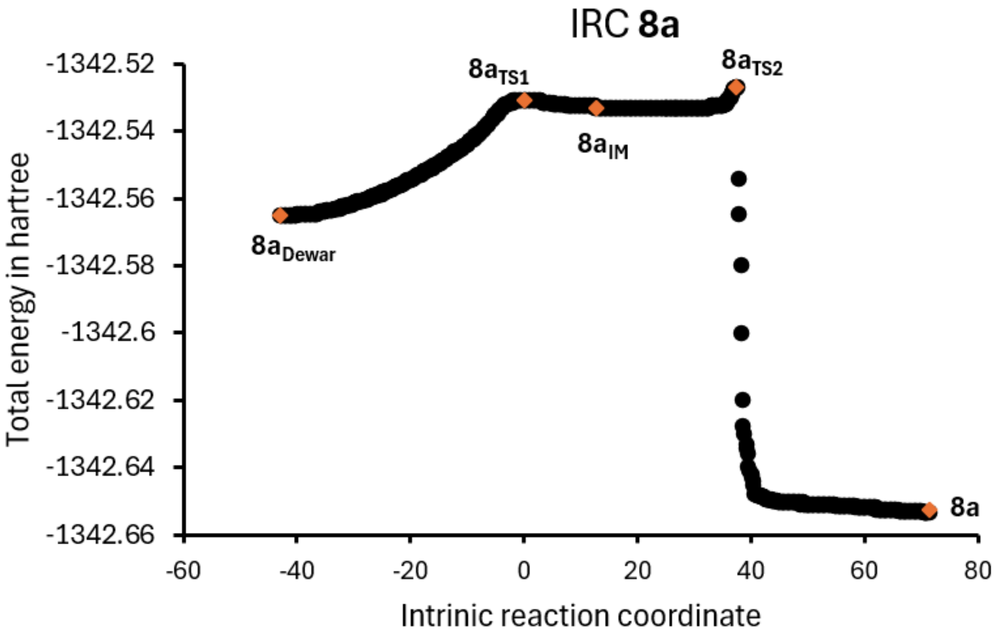

Figure S198. Intrinsic reaction coordinate of the thermal ring opening of **8a*Dewar***. The total energies of the optimized structures are shown in orange. All energies are given in hartree.

Coordinates 8e

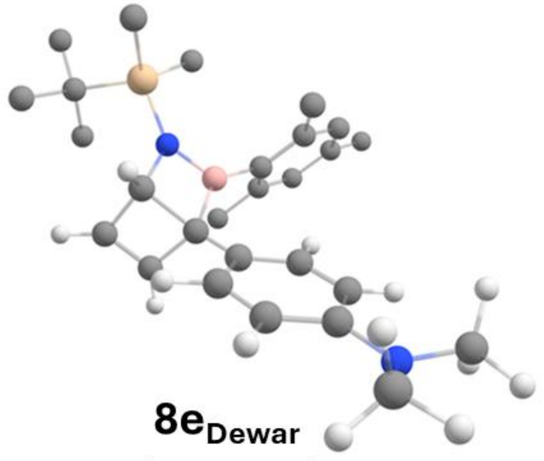

C	-0.05903700	-1.45217000	2.43881200
C	-0.47420000	-0.93268100	1.06150000
C	0.62094600	-1.93598100	0.52887700
C	0.87821100	-2.29545200	1.98657700
H	-0.41036600	-1.18628200	3.43035000
H	0.36962100	-2.72535300	-0.18059900
H	1.61097300	-2.92850600	2.47184700
C	0.48341600	1.71947100	0.19051800
C	-0.05328600	2.29019300	-0.98426200
C	0.93593800	2.58000200	1.21402700
C	-0.11244600	3.67974100	-1.11855200
C	0.86724300	3.96474400	1.04383000
C	0.34895400	4.53708800	-0.11872100
H	-0.53255800	4.10304200	-2.02696200
H	1.22276200	4.61193800	1.84117900
C	1.48583200	2.01869900	2.50783400
H	0.71429000	1.48025500	3.06788400
H	2.30054600	1.31065600	2.33011000
H	1.86766900	2.81338500	3.15225800
C	-0.57345000	1.41725700	-2.10643700
H	0.22934500	0.83294600	-2.56760000
H	-1.32337900	0.70641100	-1.74783300
H	-1.03416600	2.02071700	-2.89145900
C	0.31118200	6.03582400	-0.29903800
H	0.19694600	6.55044600	0.65827700
H	1.23757300	6.40038900	-0.75716300
H	-0.51426800	6.33894400	-0.94785200
C	3.45321500	0.82521400	-1.47272600
H	2.64240600	1.35782000	-1.97435200
H	3.73819800	1.41766800	-0.59987900
H	4.30759700	0.79949200	-2.15572500
C	2.53767200	-1.90860400	-2.57337300
H	2.13888500	-2.90098900	-2.34502200
H	1.78747000	-1.38209700	-3.17074300

H	3.42334800	-2.04570200	-3.20120600
C	4.35289200	-1.80195400	-0.03633900
C	4.53804900	-1.14392500	1.34672700
H	5.35828100	-1.62963000	1.89096000
H	4.79021400	-0.08216600	1.26245400
H	3.63822000	-1.22433400	1.96160400
C	4.05010900	-3.30432800	0.14993200
H	4.85706500	-3.78374700	0.71888500
H	3.12051600	-3.47886800	0.69756900
H	3.97527000	-3.82631000	-0.80845500
C	5.67304000	-1.66935300	-0.82887000
H	5.98133200	-0.62640500	-0.94330900
H	6.48091200	-2.19188200	-0.30106500
H	5.60268200	-2.11087400	-1.82812300
C	-1.92670400	-0.98213800	0.67658100
C	-2.54025100	-2.13827700	0.18383200
C	-2.74283500	0.14410100	0.83453100
C	-3.89276600	-2.17635900	-0.13979400
H	-1.95420000	-3.04069500	0.04468100
C	-4.09865400	0.12308300	0.52624400
H	-2.30685800	1.06934400	1.19645800
C	-4.71802500	-1.04659500	0.03842400
H	-4.30191000	-3.09887400	-0.52810900
H	-4.67033100	1.03047500	0.66386600
N	1.45467300	-0.82333600	-0.04444300
B	0.53061300	0.16320200	0.38543300
Si	2.94002300	-0.92577100	-1.01027600
C	-6.82371600	0.16101200	-0.31731000
H	-6.46384500	0.82360200	-1.11988400
H	-7.87503200	-0.06009900	-0.50078300
H	-6.76604000	0.70852500	0.62665800
N	-6.08478000	-1.08873100	-0.23901500
C	-6.61168300	-2.20791200	-1.00274400
H	-7.69227000	-2.09810400	-1.09252500
H	-6.18520100	-2.27578800	-2.01583100
H	-6.42220500	-3.15429900	-0.49014200

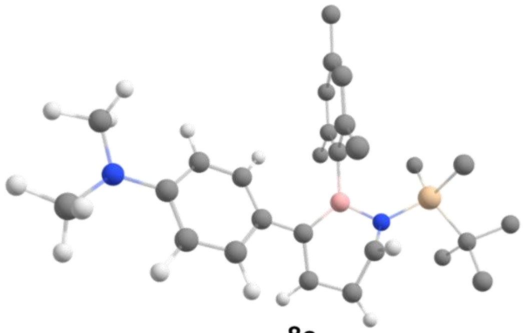

8e$_{TS1}$

C	-0.17414000	1.26730400	0.16040200
C	-0.20161800	2.12372500	1.28282700
C	0.05953600	3.48864300	1.12916000
C	0.36082900	4.04356100	-0.11513900
C	0.41180000	3.18588000	-1.21501400
C	0.15250500	1.81728600	-1.09707800
H	0.02997200	4.13246700	2.00429400
H	0.66565200	3.59124300	-2.19090100
C	0.53137600	-1.39370900	0.60985700
C	0.08011400	-2.74210600	1.00670600
C	-1.20369000	-1.82564900	-0.52048100
C	-1.05195100	-3.01710600	0.34679100
H	0.57334900	-3.32756600	1.77806200
H	-1.82953700	-3.73956700	0.56912900
B	-0.52395200	-0.27120200	0.36844300
N	-1.89908000	-0.75927600	0.08147500
Si	-3.47955000	-0.05469100	-0.28291800
C	-3.54528800	0.58836000	-2.06045200
H	-4.54638800	0.95345700	-2.30979300
H	-2.85011100	1.42242100	-2.18832100
H	-3.28525100	-0.18097300	-2.79343400
C	-4.82954400	-1.40953300	-0.04595300
C	-3.76819000	1.37760300	0.90118200
H	-3.77295900	1.05481600	1.94516600
H	-2.99543300	2.14075200	0.78613000
H	-4.73344000	1.84885300	0.69270600
C	-6.22457500	-0.78745500	-0.28129000
H	-6.33510000	-0.38895100	-1.29424900
H	-7.00230100	-1.54988100	-0.14685800
H	-6.43928900	0.02093700	0.42381700
C	-4.63563300	-2.56137500	-1.05470800
H	-4.70889700	-2.21518700	-2.09006800
H	-3.66969400	-3.06052000	-0.93746700
H	-5.41178500	-3.32429200	-0.91169500
C	-4.77855600	-1.97498300	1.38861200
H	-5.54151500	-2.75375600	1.51638100
H	-3.80642800	-2.41659300	1.62027800
H	-4.97664500	-1.20143000	2.13679200
C	0.27556500	0.93952000	-2.32333500

H	1.06123000	0.18944000	-2.19114700
H	-0.65454500	0.40494800	-2.53502900
H	0.52358500	1.53100300	-3.20738600
C	0.60277200	5.52607700	-0.27062200
H	1.29757400	5.73346200	-1.08837400
H	-0.33018400	6.05631400	-0.49296500
H	1.01467000	5.96133300	0.64328000
C	-0.46455100	1.57766100	2.67105800
H	0.38883500	0.99315200	3.03190700
H	-0.63871000	2.38562600	3.38522400
H	-1.33288000	0.91497700	2.69446000
H	-0.94428300	-1.81925700	-1.57743300
C	1.98512000	-1.22860300	0.39173800
C	2.76877000	-2.33016000	-0.00477300
C	2.68334100	-0.02449000	0.60345800
C	4.14226300	-2.25058100	-0.17655400
H	2.27950900	-3.27720800	-0.20522200
C	4.05732800	0.07181800	0.44543800
H	2.14405500	0.86059700	0.90924100
C	4.83513300	-1.04244400	0.05839300
H	4.67421600	-3.13534000	-0.49820500
H	4.52679300	1.02778800	0.63219300
N	6.21313500	-0.95683800	-0.07134500
C	6.94394100	-2.05669400	-0.68009600
H	8.01034800	-1.83643500	-0.64565200
H	6.78307800	-2.98642200	-0.12797300
H	6.66026300	-2.22770500	-1.72925500
C	6.85352300	0.34907000	-0.05497400
H	7.93245500	0.21576200	-0.12595200
H	6.52894700	0.99193800	-0.88637800
H	6.64883200	0.87454400	0.88152200

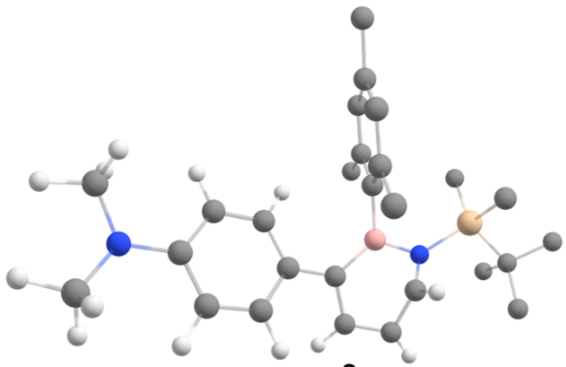

$8e_{IM}$

C	0.20404400	1.08321300	0.15255600
C	-0.02126500	1.74672700	1.38170500
C	-0.21525300	3.13105900	1.41029300
C	-0.19998700	3.90567600	0.24942200
C	-0.01182000	3.24623500	-0.96378000
C	0.17918100	1.86133400	-1.02877200
H	-0.38901300	3.61731500	2.36689400
H	-0.02127000	3.82062000	-1.88688400
C	-0.69151100	-1.52943800	-0.10439400
C	-0.21224800	-2.90087700	0.04613500
C	1.00664200	-2.93764600	0.61015300
H	-0.72157500	-3.75531100	-0.38803600
H	1.69762900	-3.77478600	0.57307900
B	0.45353400	-0.49218400	0.03240700
N	1.98063300	-0.92547400	-0.14467000
Si	3.51099000	-0.02609400	-0.15098200
C	3.72340600	1.03656600	1.40012800
H	4.69241600	1.54531000	1.39075700
H	2.94577100	1.80358300	1.44278400
H	3.67107600	0.45178400	2.32295900
C	4.90934500	-1.35868200	-0.21910400
C	3.62122400	1.07909600	-1.66997200
H	3.41821000	0.53282400	-2.59391000
H	2.91673600	1.91002600	-1.60285300
H	4.62627900	1.50501300	-1.74604800
C	4.75303000	-2.22880500	-1.48345900
H	4.83533500	-1.63465300	-2.39875400
H	5.54148700	-2.99207500	-1.51879800
H	3.78715600	-2.73935300	-1.50534600
C	6.28527000	-0.65715000	-0.26426500
H	6.39471400	-0.01816300	-1.14529100
H	6.46527600	-0.04182600	0.62248000
H	7.08611700	-1.40647900	-0.30686600
C	4.87085900	-2.26872000	1.02553600
H	5.68269700	-3.00627900	0.97831900
H	5.00107000	-1.70303100	1.95324500
H	3.93354800	-2.82573000	1.10050400
C	1.44334000	-1.55528500	0.97284600
H	1.66409900	-1.26264200	1.99850500

C	-0.07477700	0.99387700	2.69417100
H	-0.65624000	0.07375600	2.60750900
H	0.92621800	0.71386600	3.03952800
H	-0.52540700	1.61041900	3.47556500
C	0.27755800	1.22170700	-2.40005800
H	0.86156000	0.30190400	-2.38824400
H	-0.71804200	0.96137600	-2.77933900
H	0.72711500	1.90650500	-3.12349000
C	-0.36651300	5.40545400	0.31037300
H	-0.75340000	5.80282400	-0.63123600
H	-1.05211400	5.69903600	1.10976000
H	0.59119400	5.90113500	0.50564000
C	-2.12562600	-1.29275500	-0.12662500
C	-3.03491000	-2.28843500	0.30342000
C	-2.70797800	-0.08490400	-0.57343900
C	-4.40489200	-2.11531100	0.26940900
H	-2.64860600	-3.21974100	0.70033200
C	-4.07541400	0.10692500	-0.61636300
H	-2.06833000	0.72455100	-0.89080600
C	-4.97380000	-0.90861400	-0.20715500
H	-5.03845000	-2.91551600	0.62617400
H	-4.45083600	1.05689200	-0.97062500
N	-6.33689300	-0.73071800	-0.26771500
C	-6.88884600	0.56675000	-0.63004400
H	-6.54677500	0.87938200	-1.62119700
H	-7.97471100	0.49463400	-0.66178200
H	-6.61709300	1.34995200	0.08977500
C	-7.23136100	-1.74361200	0.27251900
H	-7.10987900	-1.87230500	1.35641600
H	-8.26123800	-1.44882700	0.07813000
H	-7.06662100	-2.71272500	-0.20786100

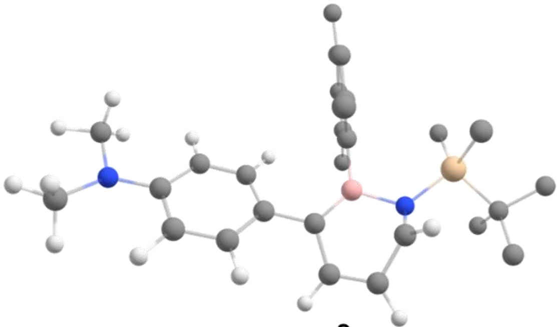

8e$_{TS2}$

C	0.22551900	1.06460100	0.02426200
C	0.25450300	1.87897500	-1.12810400
C	0.07220100	3.26184600	-1.01623900
C	-0.14427200	3.87556500	0.21613400
C	-0.19208600	3.06180500	1.35118600
C	-0.02265300	1.67780400	1.27559100
H	0.10045500	3.87189200	-1.91533500
H	-0.37826400	3.51694500	2.32045200
C	-0.69100100	-1.52005500	-0.20467700
C	-0.24187600	-2.91102800	-0.22071000
C	1.65666400	-1.93059900	0.79691200
C	0.98021600	-3.14841000	0.27468600
H	-0.82964500	-3.68919600	-0.70119100
H	1.56841800	-4.05078000	0.13381400
B	0.38946100	-0.50386000	-0.12827300
N	1.96051000	-0.95518700	-0.05106200
Si	3.49164700	-0.00474900	0.14241300
C	3.66875900	0.59931300	1.92115200
H	4.64821100	1.06647400	2.06207600
H	2.90940800	1.35391900	2.14006000
H	3.57978100	-0.19749900	2.66430800
C	4.89347700	-1.25845200	-0.29248700
C	3.56850700	1.45376900	-1.03218600
H	3.38888800	1.16525600	-2.06953800
H	2.84920700	2.22752900	-0.76398700
H	4.56942400	1.89300100	-0.97723800
C	6.25571400	-0.53323400	-0.19197300
H	6.45156000	-0.15535200	0.81616200
H	7.06599500	-1.22988600	-0.43923600
H	6.32993200	0.30679400	-0.88786300
C	4.91424700	-2.45643100	0.67769700
H	5.04414000	-2.14632100	1.71908300
H	4.00506200	-3.06117300	0.61312500
H	5.75051100	-3.12238700	0.43088900
C	4.71274900	-1.78280700	-1.73317800
H	5.51828500	-2.48506000	-1.98218200
H	3.76204300	-2.30709900	-1.85960500
H	4.74859600	-0.97434300	-2.46928300
C	-0.15430600	0.85553200	2.53807100
H	-0.95357200	0.11667300	2.43787400

H	0.76104600	0.30188900	2.76786400
H	-0.38046000	1.49204600	3.39655800
C	-0.35360100	5.36710800	0.32490400
H	-0.06933700	5.87762900	-0.59785900
H	-1.40455100	5.60361400	0.52384900
H	0.23373700	5.79417100	1.14282400
C	0.41752000	1.27891500	-2.50864700
H	-0.55479100	0.98556800	-2.92032200
H	0.86265400	1.99747700	-3.20162300
H	1.03510200	0.37920400	-2.49918000
H	2.03159400	-1.91391900	1.82810600
C	-2.13988700	-1.27106300	-0.20965000
C	-3.03930000	-2.24610400	0.27102300
C	-2.73841700	-0.08258700	-0.67470600
C	-4.41595900	-2.06669800	0.27724000
H	-2.64783900	-3.17109400	0.68028800
C	-4.11131400	0.11568600	-0.66969000
H	-2.11446100	0.71448300	-1.05353300
C	-4.99985600	-0.87875700	-0.20792300
H	-5.03413500	-2.86060700	0.67400400
H	-4.49053300	1.05738100	-1.04287800
N	-6.38413200	-0.70263600	-0.24864100
C	-7.22945600	-1.62393900	0.49244200
H	-8.27413400	-1.37308700	0.30820400
H	-7.07592900	-2.65135800	0.15379100
H	-7.05168100	-1.59175300	1.57907600
C	-6.91487800	0.63373100	-0.46363600
H	-8.00275300	0.58003700	-0.50670400
H	-6.63362600	1.34077300	0.33257800
H	-6.57063500	1.04177500	-1.41689600

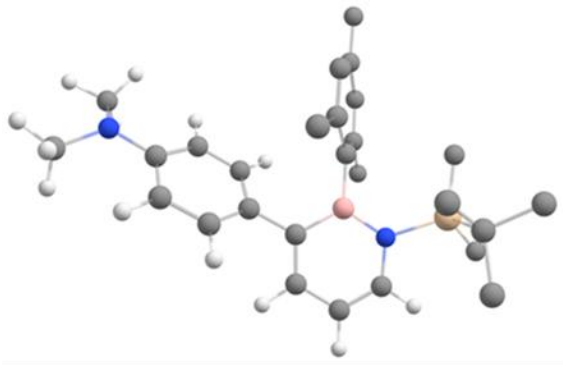

8e

C	-0.19233600	-2.79128800	-1.05982800
C	-0.54542400	-1.52078000	-0.65694300
C	2.15807800	-2.25629900	-1.11929200
C	1.15240100	-3.15356000	-1.31481900
H	-0.96072000	-3.54530700	-1.21600600
H	3.17951200	-2.55487100	-1.31713100
H	1.39748500	-4.14700100	-1.67017400
C	0.26414700	1.04631400	-0.19867300
C	0.24520700	1.97402700	-1.26413000
C	-0.11002800	1.49870800	1.08468900
C	-0.09906700	3.30830900	-1.03056200
C	-0.43535800	2.84281400	1.28797300
C	-0.42987600	3.76875000	0.24439000
H	-0.11334800	4.00396900	-1.86562100
H	-0.71480500	3.17141000	2.28554200
C	-0.20821500	0.54009900	2.25082500
H	-1.05645500	-0.13957000	2.12060500
H	0.68566400	-0.07940900	2.35136700
H	-0.35209000	1.07652600	3.19158800
C	0.58088900	1.54747300	-2.67919300
H	1.64997500	1.34319700	-2.80232700
H	0.05380500	0.63203500	-2.96371800
H	0.31345100	2.32656100	-3.39661800
C	-0.75774300	5.22230600	0.48943000
H	-1.48116800	5.33686500	1.30066300
H	0.13867900	5.78699600	0.76987600
H	-1.17322100	5.69379100	-0.40466500
C	3.34226300	1.73341500	0.02454800
H	2.86229100	2.26572000	-0.79652500
H	2.77074200	1.94631800	0.92839300
H	4.34664700	2.15129700	0.14801500
C	4.59359800	-0.25771200	-1.89444600
H	4.88130600	-1.28254500	-2.13835800
H	4.05783700	0.14852400	-2.75751000
H	5.51417700	0.32236300	-1.77869200
C	4.41343200	-0.94101000	1.16998600
C	3.49189600	-0.92989300	2.40655000

H	4.01573800	-1.35856700	3.27021200
H	3.18487000	0.08263600	2.68366800
H	2.58928300	-1.52486700	2.24301300
C	4.84347000	-2.39775300	0.89486800
H	5.48924200	-2.48218200	0.01523500
H	5.41381500	-2.77987400	1.75081100
H	3.98793800	-3.06232400	0.75666700
C	5.68783500	-0.12428900	1.49413400
H	5.45954400	0.90332100	1.78744800
H	6.22060000	-0.59052800	2.33187300
H	6.38373400	-0.08864100	0.64985600
C	-1.98179400	-1.25835800	-0.36967400
C	-2.66645700	-0.13275400	-0.84947200
C	-2.73958900	-2.16669200	0.38625400
C	-4.01812500	0.07318300	-0.60207300
H	-2.14005600	0.59890800	-1.44822600
C	-4.08984400	-1.97309100	0.65216500
H	-2.25531800	-3.04703900	0.79622800
C	-4.77010600	-0.83501200	0.17188200
H	-4.48448800	0.95472300	-1.01975900
H	-4.60737200	-2.71239000	1.24799300
N	1.95438900	-0.95784300	-0.68647400
B	0.58357600	-0.48128000	-0.49548200
Si	3.53525100	-0.09867400	-0.33557900
C	-6.83064400	0.42222900	-0.26702400
H	-6.89073500	0.21629800	-1.34717700
H	-7.84503300	0.49607400	0.12485700
H	-6.35721000	1.39780700	-0.13293500
N	-6.11400100	-0.60725100	0.46808500
C	-6.90944000	-1.69985700	1.00394200
H	-6.49475000	-2.05828200	1.94941900
H	-7.91704400	-1.33784200	1.20781300
H	-6.98164800	-2.55627700	0.31549700

IRC 8e

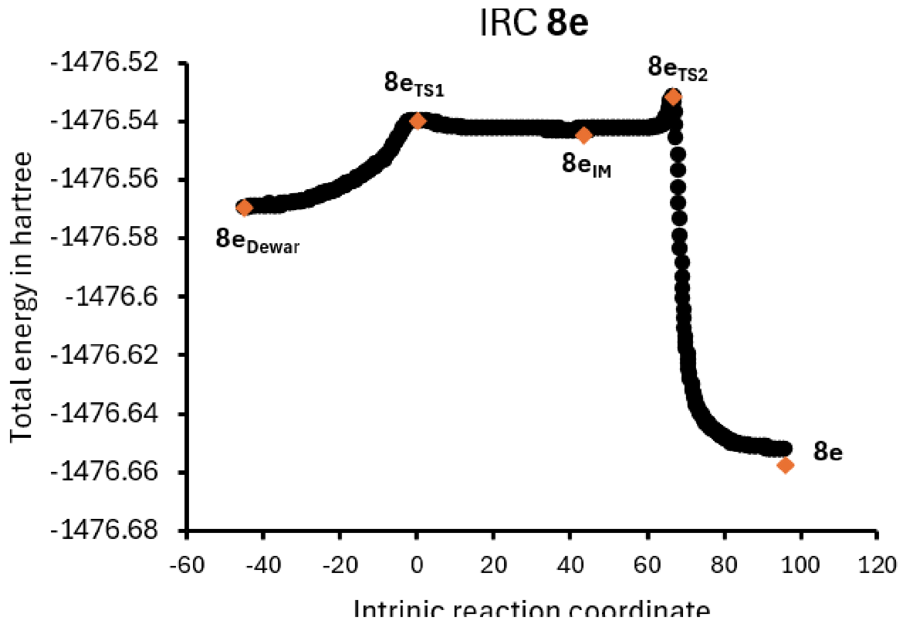

Figure S199. Intrinsic reaction coordinate of the thermal ring opening of **8e**$_{Dewar}$. The total energies of the optimized structures are shown in orange. All energies are given in hartree.

Coordinates of 8k

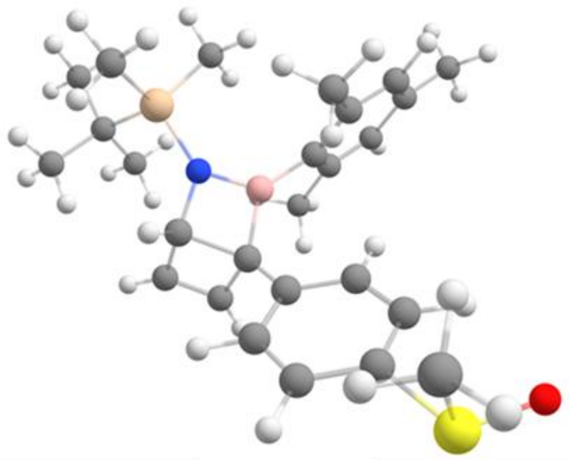

$8k_{Dewar}$

C	-0.24025800	-1.46226900	-2.46407000
C	0.20588500	-0.98633600	-1.08127100
C	-0.92247500	-1.94781600	-0.55215900
C	-1.20770900	-2.27103600	-2.01415900
H	0.11080300	-1.19213300	-3.45396100
H	-0.70889300	-2.76338300	0.13948500
H	-1.97265700	-2.86272700	-2.50114600
C	-0.62972900	1.69810000	-0.19633000
C	-0.01540600	2.24359300	0.95227900
C	-1.09008900	2.57778000	-1.19960500
C	0.10870800	3.62838000	1.08376000
C	-0.95399600	3.95804700	-1.03270600
C	-0.36006400	4.50602800	0.10477000
H	0.58682600	4.03212300	1.97202200
H	-1.31660000	4.62114800	-1.81341600
C	-1.71503600	2.04471700	-2.47119300
H	-0.97366000	1.53435300	-3.09527200
H	-2.50835000	1.32110500	-2.26415900
H	-2.14521000	2.85183100	-3.06775200
C	0.51704500	1.34635900	2.04922100
H	-0.28381500	0.77121000	2.52510400
H	1.24341800	0.62590500	1.66152100
H	1.01152100	1.92996500	2.82846700
C	-0.24854500	6.00109700	0.28269900
H	-0.20092900	6.51506900	-0.68026300
H	-1.11522900	6.39798500	0.82320500
H	0.64294700	6.26930000	0.85493900
C	-3.59223900	0.90218300	1.53791500
H	-2.74673700	1.37794400	2.03968700
H	-3.85622700	1.52940900	0.68308200
H	-4.43726400	0.90712900	2.23281700
C	-2.79727500	-1.89853400	2.56286900
H	-2.45214900	-2.90402700	2.30611000

H	-2.01280000	-1.42176100	3.15802700
H	-3.67724800	-2.00721100	3.20399900
C	-4.64707100	-1.64711200	0.05954900
C	-4.82078700	-0.95016100	-1.30574800
H	-5.67434800	-1.38043100	-1.84496500
H	-5.01523900	0.12117000	-1.19504200
H	-3.93743400	-1.06456000	-1.93902500
C	-4.42025000	-3.15779000	-0.16377400
H	-5.25788100	-3.58492400	-0.72966400
H	-3.50890700	-3.36464500	-0.73079000
H	-4.35605100	-3.70444800	0.78148100
C	-5.94686900	-1.46792900	0.87654000
H	-6.20259800	-0.41406900	1.01714900
H	-6.78690600	-1.93958500	0.35141400
H	-5.88287800	-1.93353100	1.86518700
C	1.65848100	-1.07522000	-0.72208600
C	2.18536800	-2.12020400	0.04907800
C	2.53952400	-0.08897200	-1.19148500
C	3.54533400	-2.17764300	0.34755800
H	1.53376000	-2.90636500	0.41170600
C	3.90077500	-0.13858600	-0.90520300
H	2.15036200	0.73112900	-1.78544500
C	4.39173300	-1.17742600	-0.12214200
H	3.93541100	-3.00592200	0.93127600
H	4.59039900	0.61016500	-1.27938200
N	-1.70285500	-0.80912200	0.04421700
B	-0.75072900	0.14784600	-0.38532300
Si	-3.18131000	-0.86221600	1.03059000
S	6.18800400	-1.25100100	0.20155800
O	6.82770300	-0.13566100	-0.60063100
C	6.12578700	-0.66291600	1.94351900
H	5.68060600	0.33206800	1.95729200
H	5.54435800	-1.36517200	2.54235400
H	7.15684700	-0.62514100	2.29594300

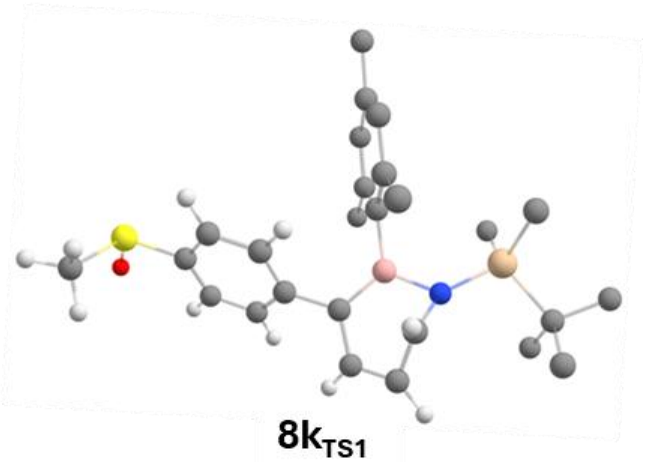

8k$_{TS1}$

C	-0.26303700	1.15363400	0.03390100
C	-0.27111400	1.99715600	-1.09882000
C	0.12513300	3.33266000	-0.98089500
C	0.53283100	3.87581300	0.23741300
C	0.54931800	3.03437900	1.34988900
C	0.16698600	1.69290100	1.26769200
H	0.11969900	3.96311500	-1.86593300
H	0.86905800	3.43167300	2.30946600
C	0.19554600	-1.57942000	0.04307900
C	-0.36568800	-2.93360700	0.18270800
C	-1.54253600	-2.98791700	-0.45213200
H	0.08206400	-3.71895700	0.78221900
H	-2.35353700	-3.69672600	-0.33230500
B	-0.75686100	-0.35889100	-0.00060400
N	-2.20119700	-0.66379700	-0.26037500
Si	-3.76199800	0.15535600	-0.07274000
C	-4.21580600	1.13662000	-1.62239100
H	-5.22626700	1.54784200	-1.53742100
H	-3.53146000	1.97716200	-1.76110300
H	-4.18576500	0.52626100	-2.52967200
C	-5.12167600	-1.16878200	0.25648800
C	-3.58852900	1.32422500	1.38747700
H	-3.35439600	0.79131700	2.31240400
H	-2.79558000	2.05405900	1.20984900
H	-4.52044200	1.87535100	1.54491700
C	-4.77870800	-1.99857000	1.51086100
H	-4.73128100	-1.37474600	2.40850100
H	-5.55098900	-2.75880700	1.68411200
H	-3.81936000	-2.51236100	1.41437800
C	-6.47448100	-0.45862000	0.49198400
H	-6.44243400	0.21288300	1.35505800
H	-6.79461400	0.12390500	-0.37675900
H	-7.25708700	-1.20185700	0.68904700
C	-5.27142700	-2.10996100	-0.95722900
H	-6.04654100	-2.86149900	-0.76071400
H	-5.56712900	-1.56866500	-1.86082500

H	-4.34906200	-2.65364600	-1.18132700
C	-1.67380700	-1.65212000	-1.07966700
H	-1.45190700	-1.47461200	-2.13227200
C	-0.63359200	1.47736500	-2.47426600
H	0.17173100	0.86432400	-2.89560100
H	-1.53228400	0.85999300	-2.45761700
H	-0.80714600	2.29981100	-3.17150600
C	0.23566100	0.84168300	2.51847900
H	-0.68237100	0.26952000	2.67688500
H	1.05654800	0.11987800	2.46098100
H	0.39992400	1.46204000	3.40230200
C	0.91758100	5.33103000	0.35360300
H	1.66181800	5.48504000	1.13872700
H	1.33046700	5.71049900	-0.58415700
H	0.04760000	5.94928500	0.60209800
C	1.66936700	-1.45129300	-0.05333800
C	2.49534300	-2.30358600	0.71115600
C	2.30597700	-0.50442900	-0.87347600
C	3.87960500	-2.18911600	0.68363000
H	2.05322800	-3.05783000	1.35031900
C	3.69238000	-0.39335000	-0.92108400
H	1.71094900	0.14626400	-1.49700900
C	4.47295100	-1.22263800	-0.12623800
H	4.48660500	-2.86106400	1.28264500
H	4.17998300	0.32192200	-1.57428800
S	6.28950700	-1.08983700	-0.23987300
C	6.54505800	-0.19400800	1.34657500
H	7.61610000	-0.00752000	1.42932600
H	5.99495100	0.74621800	1.30544600
H	6.20212100	-0.81716200	2.17359500
O	6.59316800	-0.09622000	-1.34302600

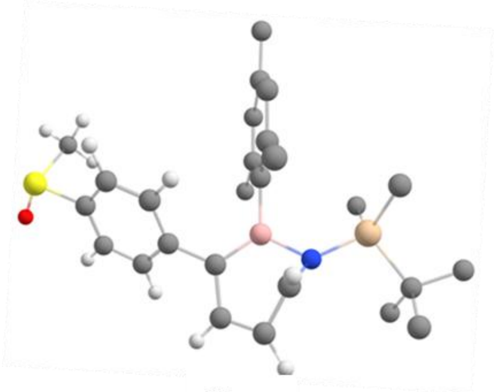

8k$_{IM}$

C	-0.43244300	1.09266400	-0.21777600
C	-0.52009900	1.94440800	-1.34323500
C	-0.29068700	3.31681200	-1.20382100
C	0.03086300	3.89204100	0.02631300
C	0.14670200	3.04332100	1.12624100
C	-0.06974200	1.66568200	1.02276900
H	-0.35729200	3.95228200	-2.08290000
H	0.41954400	3.46119000	2.09166600
C	0.42202800	-1.52847100	-0.36201400
C	-0.04765300	-2.88833800	-0.57283500
C	-1.32048400	-2.91176300	-1.00379400
H	0.52424500	-3.76907900	-0.30247200
H	-2.00554900	-3.75036200	-0.92784800
B	-0.68709500	-0.48144600	-0.27304400
N	-2.20852900	-0.92333500	-0.07855700
Si	-3.70139900	-0.01573300	0.29022800
C	-4.17096900	1.21628600	-1.06175000
H	-5.13114700	1.68610300	-0.82697500
H	-3.42460200	2.01085700	-1.13314400
H	-4.26928400	0.75024600	-2.04613500
C	-5.07303400	-1.36190400	0.45111400
C	-3.46557400	0.89117400	1.91732500
H	-3.14326800	0.22010500	2.71680700
H	-2.72344100	1.68587900	1.81942600
H	-4.40743700	1.35385900	2.22725800
C	-4.71161000	-2.37060000	1.56109500
H	-4.62516800	-1.88647500	2.53841000
H	-5.49391900	-3.13564300	1.64510300
H	-3.76555000	-2.87807800	1.35755300
C	-6.40934300	-0.67682600	0.81753400
H	-6.34952900	-0.13668300	1.76691600
H	-6.73879800	0.02787800	0.04837600
H	-7.19810000	-1.43182400	0.92436800
C	-5.26108200	-2.11871200	-0.87953500
H	-6.05784200	-2.86638300	-0.77821800
H	-5.54686600	-1.45039700	-1.69745700
H	-4.35729500	-2.65465800	-1.18310300
C	-1.81253600	-1.52361500	-1.23733600

H	-2.03247900	-1.14169700	-2.23471400
C	-0.81786800	1.40973200	-2.72880500
H	-0.20497500	0.53569000	-2.96531600
H	-1.86456000	1.10911000	-2.83536900
H	-0.62372600	2.17103400	-3.48738800
C	0.14914600	0.80859100	2.25187800
H	-0.58384800	0.00406600	2.33229200
H	1.13738300	0.33692800	2.22298300
H	0.09745700	1.40940000	3.16287900
C	0.23081000	5.38239600	0.16207700
H	0.64683200	5.81443900	-0.75158500
H	-0.71996200	5.89044100	0.35880500
H	0.90482500	5.62112200	0.98827500
C	1.87371100	-1.27010300	-0.32958400
C	2.74606600	-2.22281700	0.23906300
C	2.44959800	-0.09170100	-0.84414100
C	4.11597700	-2.00081700	0.32186900
H	2.34792100	-3.14315000	0.64825300
C	3.82114600	0.12458800	-0.78981500
H	1.82044100	0.65361900	-1.30808100
C	4.64686000	-0.82101000	-0.18563400
H	4.78388400	-2.73276100	0.76273000
H	4.23722100	1.02825700	-1.22433600
C	6.47840100	0.65415000	1.22653200
H	6.03443200	0.20309000	2.11407800
H	7.52559400	0.89939300	1.40524200
H	5.92679000	1.54246300	0.91609800
S	6.45564400	-0.58578900	-0.13224100
O	7.04917900	-1.85722600	0.44036300

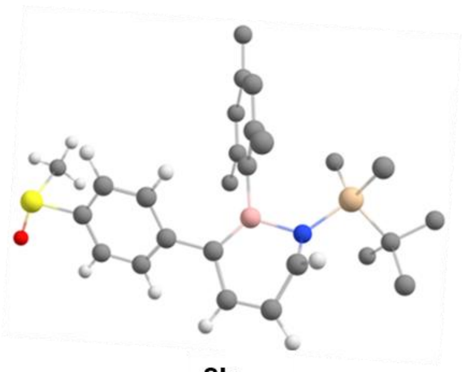

8k$_{TS2}$

C	0.46883200	1.09028700	0.17140300
C	0.58073500	1.90073700	1.32511000
C	0.43070400	3.28641200	1.22058500
C	0.15759900	3.90930700	0.00104300
C	0.01281700	3.09909100	-1.12508400
C	0.16022500	1.71052800	-1.05965100
H	0.51324700	3.89372600	2.11785300
H	-0.22595900	3.55776500	-2.08083000
C	-0.47151100	-1.50547800	0.34785500
C	-0.01253300	-2.88859700	0.47900400
C	1.24644100	-3.03761600	0.90416800
H	-0.61627800	-3.73922900	0.18256100
H	1.83994800	-3.94538100	0.85027300
B	0.60871100	-0.48828600	0.21920100
N	2.17325200	-0.94772300	0.14367800
Si	3.70373100	-0.02523600	-0.14475900
C	4.24372600	0.93307300	1.38672800
H	5.24646600	1.34299000	1.23162800
H	3.57075300	1.77306300	1.57191100
H	4.28176500	0.31956100	2.29090900
C	4.99047200	-1.40037700	-0.56039900
C	3.49530500	1.13515700	-1.60031900
H	3.10363100	0.62411000	-2.48233800
H	2.82696100	1.96320500	-1.36259100
H	4.47033100	1.55597700	-1.86446800
C	4.52847900	-2.22213800	-1.78253500
H	4.41100800	-1.59763700	-2.67296400
H	5.27343600	-2.99136200	-2.02118900
H	3.57548500	-2.72508100	-1.60065800
C	6.34172400	-0.72636000	-0.89487100
H	6.26924900	-0.06216800	-1.76048700
H	6.73689000	-0.14656200	-0.05541200
H	7.08758300	-1.49276200	-1.13768600
C	5.20524400	-2.34926300	0.63534400
H	5.96382200	-3.10113900	0.38538400
H	5.55462100	-1.82073900	1.52739900
H	4.29730600	-2.89963800	0.90045200
C	1.94352700	-1.74449400	1.17739800

H	2.38101400	-1.54713700	2.16362400
C	0.79107400	1.29540800	2.69659900
H	0.03117300	0.53968500	2.91550600
H	1.76291000	0.80323700	2.78983100
H	0.73935600	2.06215100	3.47249400
C	-0.07473900	0.89287700	-2.31066000
H	0.62770100	0.06127200	-2.39897500
H	-1.07880800	0.45602600	-2.30094300
H	0.01100800	1.51138100	-3.20715800
C	0.03451300	5.41088100	-0.09543800
H	-0.37432300	5.83764900	0.82380100
H	1.01252300	5.87568800	-0.26351800
H	-0.61328400	5.70546600	-0.92440600
C	-1.91834200	-1.23296700	0.30733600
C	-2.82766400	-2.20418300	-0.17118700
C	-2.47604800	-0.00989300	0.74254100
C	-4.19592600	-1.96721000	-0.23892500
H	-2.46236200	-3.16248900	-0.51832300
C	-3.84265900	0.23091700	0.69036400
H	-1.83122000	0.75654500	1.14689700
C	-4.70080000	-0.74241200	0.18066100
H	-4.88252900	-2.72286200	-0.60481600
H	-4.23299300	1.17405800	1.06125400
C	-6.53878900	0.61865800	-1.33856100
H	-6.12790700	0.06572300	-2.18352100
H	-7.58525800	0.86810700	-1.51660500
H	-5.96003400	1.52135500	-1.13840600
S	-6.50060400	-0.47137200	0.14340400
O	-7.14132000	-1.77973400	-0.27964200

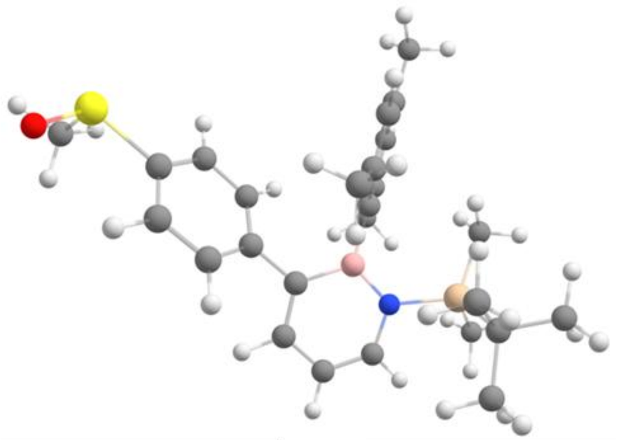

8k

C	-0.08353900	-2.73163200	-1.04208100
C	-0.37497500	-1.44263100	-0.65032600
C	2.28535900	-2.29759100	-1.10721900
C	1.24200200	-3.15327100	-1.29948300
H	-0.88533200	-3.45223300	-1.18774500
H	3.29239600	-2.64338600	-1.30237600
H	1.44355700	-4.15832000	-1.64844700
C	0.53718800	1.08985900	-0.20977000
C	0.55463400	2.00817100	-1.28389800
C	0.18003200	1.56679900	1.06944700
C	0.25707700	3.35543500	-1.06271700
C	-0.09815800	2.92346400	1.25977800
C	-0.06014400	3.83867400	0.20760000
H	0.27023500	4.04404500	-1.90350000
H	-0.36477800	3.27123100	2.25420400
C	0.04920100	0.62521800	2.24655800
H	-0.86119900	0.02268200	2.16243200
H	0.88785800	-0.07077100	2.31363800
H	-0.00644500	1.17569000	3.18838100
C	0.88356200	1.55889100	-2.69359700
H	1.94822300	1.32966400	-2.81108200
H	0.33752100	0.65256200	-2.97184600
H	0.63667200	2.33634300	-3.41993900
C	-0.33745300	5.30478300	0.43852000
H	-1.05380200	5.45235100	1.25044800
H	0.57901400	5.84040800	0.71062500
H	-0.73889500	5.78121800	-0.45923000
C	3.64323900	1.64079700	0.01645900
H	3.18475400	2.18866100	-0.80661700
H	3.08251200	1.88046600	0.92033600
H	4.66381100	2.01823000	0.13712900
C	4.80814200	-0.41347600	-1.89401500
H	5.04804400	-1.45136400	-2.13438300
H	4.29111900	0.01433600	-2.75805300
H	5.75499900	0.12328800	-1.78177300
C	4.59801700	-1.07332600	1.17585100

C	3.67605200	-1.01664000	2.41097600
H	4.18034500	-1.46251700	3.27728900
H	3.41157900	0.00915100	2.68296000
H	2.74952400	-1.57451500	2.25002100
C	4.96435400	-2.54850800	0.90656000
H	5.60616000	-2.66477600	0.02771300
H	5.51722400	-2.95142500	1.76415500
H	4.08107000	-3.17669200	0.77190800
C	5.90653300	-0.31119400	1.49824700
H	5.72277400	0.72647200	1.78730500
H	6.41712700	-0.79671500	2.33859000
H	6.60464100	-0.30944900	0.65508200
C	-1.80058800	-1.11535900	-0.37102300
C	-2.42431100	0.02856300	-0.89758800
C	-2.57387000	-1.96718100	0.43686300
C	-3.76389900	0.30143400	-0.64021800
H	-1.85732300	0.71313600	-1.51359600
C	-3.91584100	-1.70465600	0.70197200
H	-2.10753200	-2.83755300	0.88540800
C	-4.50582200	-0.57606400	0.14777600
H	-4.21781800	1.19862600	-1.04980900
H	-4.50757100	-2.34866400	1.34331200
N	2.14202600	-0.99165000	-0.68162300
B	0.79484200	-0.45036000	-0.49455900
Si	3.76329600	-0.19861300	-0.33381700
S	-6.24922300	-0.21526300	0.55220200
C	-6.96072400	-0.71494300	-1.06895900
H	-6.55130000	-0.07963400	-1.85542500
H	-6.72025900	-1.76379400	-1.24335600
H	-8.03951200	-0.57747400	-0.99124100
O	-6.69784700	-1.26185400	1.55319900

IRC 8k

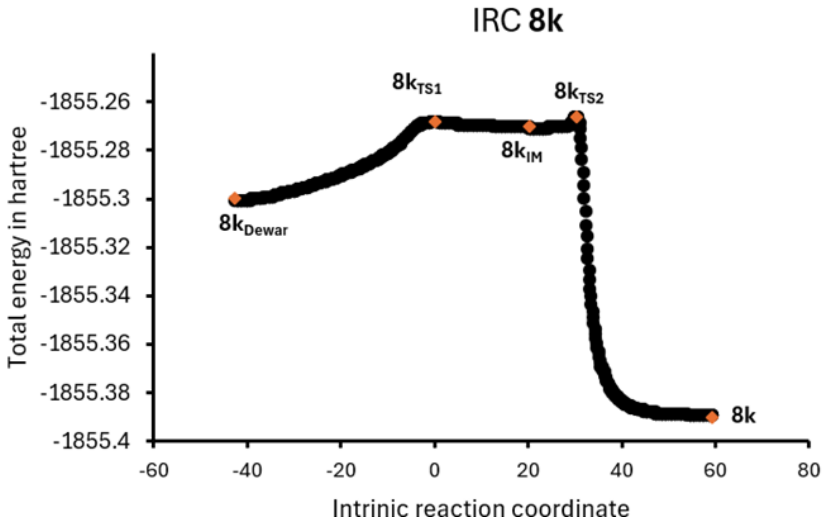

Figure S200. Intrinsic reaction coordinate of the thermal ring opening of **8k**$_{Dewar}$. The total energies of the optimized structures are shown in orange. All energies are given in hartree.

References

(1) Sheldrick, G. *Acta Crystallographica Section C* **2015**, *71*, 3-8.
(2) Dolomanov, O.; Bourhis, L.; Gildea, R.; Howard, J.; Puschmann, H. *J. Appl. Cryst. J. Appl. Cryst* **2009**, *42*, 339-341.
(3) Bourhis, L. J.; Dolomanov, O. V.; Gildea, R. J.; Howard, J. A.; Puschmann, H. *Acta Crystallogr A Found Adv* **2015**, *71*, 59-75.
(4) Richter, R. C.; Biebl, S. M.; Einholz, R.; Walz, J.; Maichle-Mössmer, C.; Ströbele, M.; Bettinger, H. F.; Fleischer, I. *Angew. Chem. Int. Ed.* **2024**, *63*, e202411078.
(5) Lamm, A. N.; Liu, S.-Y. *Mol. Biosyst.* **2009**, *5*, 1303-1305.
(6) Yang, K.; Mao, Y.; Zhang, Z.; Xu, J.; Wang, H.; He, Y.; Yu, P.; Song, Q. *Nat. Commun* **2023**, *14*, 4438.
(7) Miyaura, N.; Ishiyama, T.; Sasaki, H.; Ishikawa, M.; Sato, M.; Suzuki, A. *J. Am. Chem. Soc.* **1989**, *111*, 314-321.
(8) Kudo, N.; Perseghini, M.; Fu, G. C. *Angew. Chem. Int. Ed.* **2006**, *45*, 1282-1284.
(9) Billingsley, K. L.; Anderson, K. W.; Buchwald, S. L. *Angew. Chem. Int. Ed.* **2006**, *45*, 3484-3488.
(10) Li, C.; Xiao, G.; Zhao, Q.; Liu, H.; Wang, T.; Tang, W. *Org. Chem. Front.* **2014**, *1*, 225-229.
(11) Brown, A. N.; Li, B.; Liu, S.-Y. *J. Am. Chem. Soc.* **2015**, *137*, 8932-8935.
(12) Valencia, I.; García-García, P.; Sucunza, D.; Mendicuti, F.; Vaquero, J. J. *J. Org. Chem.* **2021**, *86*, 16259-16267.
(13) Wood, A.; Travis, E. G. *J. Am. Chem. Soc.* **1928**, *50*, 1226-1228.
(14) Brown, H. C.; Mandal, A. K. *J. Org. Chem.* **1980**, *45*, 916-917.
(15) Edel, K.; Yang, X.; Ishibashi, J. S. A.; Lamm, A. N.; Maichle-Mössmer, C.; Giustra, Z. X.; Liu, S.-Y.; Bettinger, H. F. *Angew. Chem. Int. Ed.* **2018**, *57*, 5296-5300.
(16) Hansch, C.; Leo, A.; Taft, R. W. *Chem. Rev.* **1991**, *91*, 165-195.
(17) Creary, X.; Mehrsheikh-Mohammadi, M. E.; McDonald, S. *J. Org. Chem.* **1987**, *52*, 3254-3263.
(18) Kleemiss, F.; Dolomanov, O. V.; Bodensteiner, M.; Peyerimhoff, N.; Midgley, L.; Bourhis, L. J.; Genoni, A.; Malaspina, L. A.; Jayatilaka, D.; Spencer, J. L.; et al. *Chem. Sci.* **2021**, *12*, 1675-1692.
(19) Zhao, Y.; Truhlar, D. G. *Theor. Chem. Acc.* **2008**, *120*, 215-241.
(20) Ditchfield, R.; Hehre, W. J.; Pople, J. A. *J. Chem. Phys.* **1971**, *54*, 724-728.
(21) Hehre, W. J.; Ditchfield, R.; Pople, J. A. *J. Chem. Phys.* **1972**, *56*, 2257-2261.
(22) Francl, M. M.; Pietro, W. J.; Hehre, W. J.; Binkley, J. S.; Gordon, M. S.; DeFrees, D. J.; Pople, J. A. *J. Chem. Phys.* **1982**, *77*, 3654-3665.
(23) Neese, F. *WIREs Computational Molecular Science* **2012**, *2*, 73-78.
(24) McLean, A. D.; Chandler, G. S. *J. Chem. Phys.* **1980**, *72*, 5639-5648.
(25) *Gaussian 16 Rev. C.01*; Wallingford, CT, 2016.
(26) Bauernschmitt, R.; Ahlrichs, R. *Chem. Phys. Lett.* **1996**, *256*, 454-464.
